# Interventions to reduce diabetes-related distress among adults with type 1 or type 2 diabetes: a systematic review and meta-analysis of randomised controlled trials

**DOI:** 10.1007/s00125-026-06789-0

**Published:** 2026-09-11

**Authors:** Samantha B. J. Schipper, Thomas Karagiannis, Francesco Zaccardi, Sharon Remmelzwaal, Marieke Blom, Noa Dagan, Linda J. Schoonmade, Miranda Langendam, Martin M. Jensen, Walther Jensen, Norbert Hermanns, Karin Kanc, Michelle Law, Andreia Mocan, Theresa C. Mohr, Maartje de Wit, Petra J. M. Elders, Jackie Sturt, Joline W. Beulens, Jane Speight, Richard I. G. Holt, Femke Rutters

**Affiliations:** 1 Department of Epidemiology and Data Science, Amsterdam UMC location VUmc, Amsterdam, the Netherlands; 2 Amsterdam Public Health Research Institute, Amsterdam UMC location VUmc, Amsterdam, the Netherlands; 3 Clinical Research and Evidence-Based Medicine Unit, Second Medical Department, Aristotle University of Thessaloniki, Thessaloniki, Greece; 4 Leicester Real World Evidence Unit, Leicester Diabetes Centre, University of Leicester, Leicester, UK; 5 Department of General Practice, Amsterdam UMC location VUmc, Amsterdam, the Netherlands; 6 Medical Library, Vrije Universiteit Amsterdam, Amsterdam, the Netherlands; 7 Amsterdam Public Health Research Institute, Methodology Programme, Amsterdam, the Netherlands; 8 Department of Public Health, Aarhus University, Aarhus, Denmark; 9 Public Health Research, DEFACTUM, Aarhus, Denmark; 10 European Association for the Study of Diabetes, Düsseldorf, Germany; 11 University of Bamberg and Research Institute of the Diabetes Academy Mergentheim (FIDAM), Bad Mergentheim, Germany; 12 Jazindiabetes, Private Diabetes Centre, Ljubljana, Slovenia; 13 Center for Diabetes, Nutrition and Metabolic Diseases, Emergency Clinical County Hospital, Cluj-Napoca, Romania; 14 Department of Medical Psychology, Amsterdam UMC location Vrije Universiteit Amsterdam, Amsterdam, the Netherlands; 15 Florence Nightingale Faculty of Nursing, Midwifery & Palliative Care, King’s College London, London, UK; 16 School of Psychology, Institute for Health Transformation, Deakin University, Geelong, VIC Australia; 17 The Australian Centre for Behavioural Research in Diabetes, Carlton, VIC Australia; 18 Human Development and Health, Faculty of Medicine, University of Southampton, Southampton, UK; 19 National Institute for Health Research Biomedical Research Centre, University Hospital Southampton NHS Foundation Trust, Southampton, UK

**Keywords:** Continuous glucose monitoring, Diabetes distress, Meta-analysis, Psychoeducational interventions, Psychological interventions, Randomised controlled trials, Systematic review, Type 1 diabetes, Type 2 diabetes

## Abstract

**Aims/hypothesis:**

Diabetes distress refers to the emotional and psychological burden specifically associated with living with and managing diabetes. We aimed to systematically identify, summarise and critically appraise the available evidence from RCTs assessing the efficacy of interventions for reducing diabetes distress among adults with type 1 or type 2 diabetes.

**Methods:**

We searched PubMed, the Cochrane Library, APA PsycInfo and CINAHL from inception until 23 September 2024. Studies were eligible if they were RCTs conducted in adults (aged ≥18 years) with type 1 or type 2 diabetes in which the effect of an active intervention on diabetes distress was recorded as a primary or a secondary outcome. Studies with combined populations (e.g. children plus adults) where no separate results were reported for adults were excluded. When possible, we performed a random-effects meta-analysis of the standardised mean difference (SMD) of the endpoint, diabetes distress, measured with a validated questionnaire. We conducted subgroup analyses based on baseline diabetes distress level, intervention delivery method, risk of bias assessment (using the Cochrane RoB2 tool) and trial duration. We used GRADE methodology to assess the certainty of evidence.

**Results:**

We included 248 trials, with 59 trials reporting on psychological interventions, 68 on psychoeducational interventions and 121 on miscellaneous interventions (non-psychological or non-psychoeducational, i.e. education without a psychological component, peer support without a psychological component, devices, telehealth, health behaviours and a heterogeneous group of other interventions). In total, 52 trials included participants with type 1 diabetes (including two during pregnancy), 160 included participants with type 2 diabetes, and 36 included mixed/undefined populations of adults with diabetes. The total sample size was 55,497 participants (range 12–4034). The analysed trials were judged to have either some concerns for risk of bias (94 trials) or a high risk of bias (71 trials). Compared with usual care, psychological interventions reduced diabetes distress in adults with type 1 diabetes (SMD −0.22 [95% CI −0.35, −0.08]; *N*=16; GRADE = low) and type 2 diabetes (SMD −0.31 [95% CI −0.50, −0.13]; *N*=21; GRADE = very low). Additionally, continuous glucose monitoring (CGM) or intermittently scanned glucose monitoring reduced diabetes distress in adults with type 1 diabetes, compared with standard blood glucose measurement (SMD −0.26 [95% CI −0.41, −0.11]; *N*=6; GRADE = moderate), while educational interventions reduced diabetes distress in adults with type 2 diabetes, compared with usual care (SMD −0.34 [95% CI −0.56, −0.11]; *N*=3; GRADE = low). Other comparisons, including those of psychological, psychoeducational, peer support, educational and device-based interventions with various active control interventions or usual care, did not show statistically significant effects. The results did not differ meaningfully across predefined subgroups. The certainty of evidence as assessed by GRADE methodology was moderate, low or very low in two, five and seven comparisons, respectively. No meta-analyses could be conducted for telehealth, health behaviours and the assorted mixed interventions due to heterogeneity or an insufficient number of studies.

**Conclusions/interpretation:**

Psychological interventions reduced diabetes distress among adults with type 1 diabetes and those with type 2 diabetes, while devices reduced diabetes distress in adults with type 1 diabetes and education reduced diabetes distress in adults with type 2 diabetes. Most other interventions showed little or no clear effect.

**Funding:**

European Association for the Study of Diabetes.

**Registration:**

PROSPERO registration no. CRD42024598512.

**Graphical Abstract:**

**Figure:**
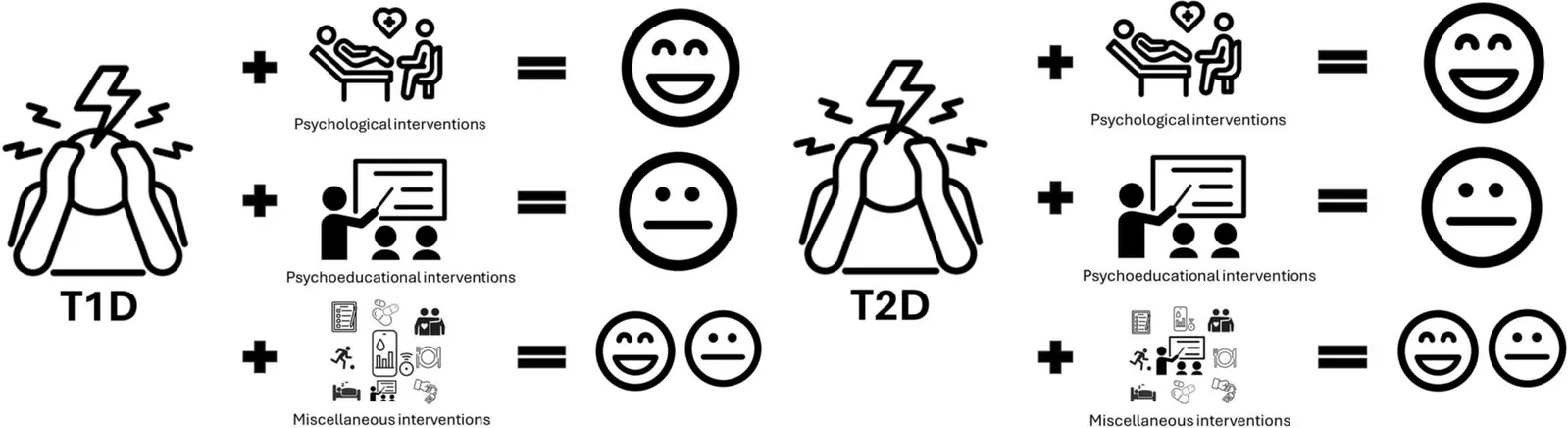

**Supplementary Information:**

The online version of this article (https://doi.org/10.1007/s00125-026-06789-0) contains peer-reviewed but unedited supplementary material.

**Figure:**
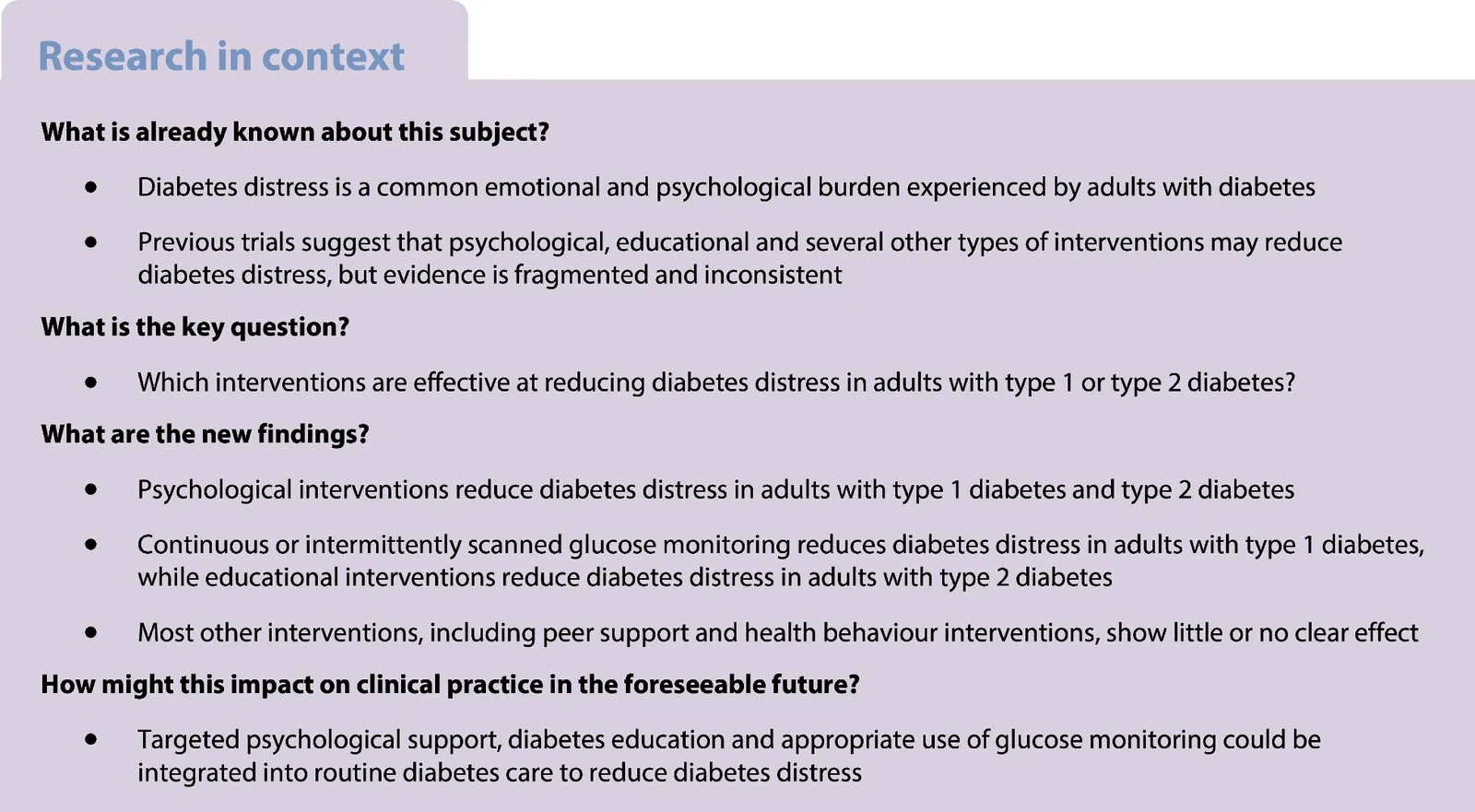

## Introduction

Beyond the physical burden associated with managing diabetes, people living with diabetes frequently experience an emotional or psychological burden. The wide range of negative feelings, including frustration, anxiety, guilt and fear, attributed to the challenges of living with and managing diabetes is often referred to as diabetes distress [1].

Factors that can contribute to diabetes distress include worries about long-term complications, frustrations over the monitoring and management of glucose levels, the burden of self-care, challenges around social interactions, the financial burden of diabetes and its management, feelings of isolation or of being overwhelmed, stigma and a perceived lack of control of the condition [1, 2]. Unlike clinical depression and anxiety, which are generic mood disorders, diabetes distress is directly related to living with and managing diabetes [3]. Although the prevalence of diabetes distress varies across populations, trial settings and the instruments used to measure it, it has been estimated that up to 80% of adults with diabetes report at least one problem area that may give rise to diabetes distress [4].

Evidence suggests a bidirectional association between diabetes distress and glycaemic indicators, whereby elevated diabetes distress is associated with higher HbA_1c_, reduced time in range and higher fasting glucose levels, and these adverse outcomes are, in turn, associated with increased diabetes distress [5]. Notably, having glucose levels within the target range does not necessarily indicate the absence of diabetes distress [6]. In addition, diabetes distress is associated with reduced engagement in glucose management and other healthy self-care behaviours. Consequently, people with diabetes distress have a higher risk of diabetes-related complications [5]. Therefore, addressing diabetes distress is beneficial not only for a person’s emotional and mental health, but also for enabling optimal diabetes management and outcomes [5].

Although diabetes distress is an important concern for people with diabetes, healthcare professionals lack detailed guidance on how to assess and manage it in clinical practice. In 2016, the ADA published a position statement on psychosocial assessment and care in people with diabetes [7]. However, these recommendations were based on commonly used clinical models, expert consensus and tested interventions, and not on a systematic review of the evidence [7]. The National Institute for Health and Care Excellence (NICE) guideline for the management of diabetes in adults in England and Wales [8, 9] makes similar recommendations, but these are also not based on systematic evidence synthesis.

The EASD has recognised the importance of diabetes distress and has commissioned an evidence-based clinical guideline to enable healthcare professionals to optimise their support of people living with diabetes and diabetes distress [10]. The purpose of this systematic review and meta-analysis was to update and expand the evidence base on the clinical management of diabetes distress by including RCTs that assess the efficacy of a wide range of interventions for diabetes distress as a primary or secondary outcome. To date, previous systematic reviews have focused only on specific types of interventions such as psychological interventions or digitally enabled peer support, or included only studies primarily designed to reduce diabetes distress [11–18]. However, interventions not specifically targeting diabetes distress may still lead to reductions as a secondary effect. Therefore, this study aimed to systematically identify, summarise and critically appraise all available evidence from RCTs that have assessed the efficacy of psychological and other interventions for the reduction of diabetes distress among adults with type 1 or type 2 diabetes.

## Methods

The protocol for this systematic review was registered in the International Prospective Register of Systematic Reviews (PROSPERO) database under number CRD42024598512. The protocol is provided in electronic supplementary material (ESM) 7. Deviations from the protocol are described in ESM 8. The systematic review was conducted in accordance with the Cochrane Handbook for Systematic Reviews of Interventions, and reported in line with the Preferred Reporting Items for Systematic Reviews and Meta-Analyses (PRISMA) guidelines [19].

### Data sources and search strategy

A comprehensive search was performed in PubMed, the Cochrane Library (Wiley), APA PsycInfo (via EBSCOhost) and CINAHL Plus (via EBSCOhost) from inception to 23 September 2024. Search terms included controlled terms (MeSH in PubMed) as well as free-text terms. Reference lists of included studies and relevant systematic reviews were searched manually for additional eligible publications. The search strategy focused on three domains: (1) diabetes, (2) distress and (3) RCTs. The three domains were included in a three-step search combining relevant search words and MeSH terms with Boolean logic operators (OR and AND). A search filter was applied to limit the results to humans. The search was performed without date or language restrictions. The full search strategy is provided in ESM 1.

### Selection of studies

Publications were included if all of the following criteria were satisfied: (1) a trial focused on adults (aged ≥18 years) with type 1 or type 2 diabetes; (2) a trial reported results on diabetes distress as a primary or secondary outcome, using data collected with the use of a validated measure such as the Diabetes Distress Scale (DDS) [2] and the Problem Areas In Diabetes (PAID) scale [1], and their subscales or derivatives (including short-form measures and versions developed for specific populations, e.g. for adults with type 1 diabetes); (3) a trial was an RCT, irrespective of its duration or the type of randomisation used (e.g. individual, cluster); and (4) a trial examined the efficacy of an active intervention, which could be either generic or diabetes specific.

There were no restrictions on delivery mode (e.g. in person, online, digital applications, or delivered by specialised mental health professionals, specialist diabetes health professionals, general health professionals or expert peers [e.g. people with lived experience of diabetes trained to deliver the intervention]), setting (individual vs groups), comparators (e.g. usual care, waiting list or active comparator), design, duration, publication date, language or status of participants (e.g. hospitalised, pregnant). We excluded studies with participants aged <18 years or with other types of diabetes (e.g. gestational diabetes), or those with combined populations (e.g. children plus adults) where no separate results were reported for adults.

The following three categories of intervention were defined:

1. Psychological interventions, which included cognition-focused, emotion-focused or emotion–cognition therapeutic interventions facilitated by a health professional trained in underpinning psychological theory. Examples included cognitive behavioural therapy, motivational interviewing, supportive counselling, mindfulness, and acceptance and commitment therapy.
2. Psychoeducational interventions, which included diabetes self-management education, peer support, health coaching or psychological interventions, all combined with a diabetes-specific educational component (e.g. carbohydrate counting, insulin dose adjustment) as well as a focus on improving self-management (e.g. cognitive reframing, goal setting, planning, problem solving) based on psychological theory or theories. Interventions in this category were typically delivered in groups or via online programmes. Examples included peer support via an online platform, motivational interviewing through an app, depression treatment via telehealth, conversation maps, and psychoeducational self-management support or nudging.
3. Miscellaneous interventions, which included interventions that could not be categorised as psychological or psychoeducational. Examples included devices (for the administration of insulin or other glucose-lowering medications or for glucose monitoring, or both); education or peer support without a clearly described psychological component (i.e. interventions with no clear description of the use of frameworks, models or techniques that have psychology or behavioural science at their core); telehealth interventions (e.g. apps, online interventions of a diverse nature); health behavioural interventions (e.g. physical activity, sleep or weight loss interventions); medication interventions (e.g. glucose-lowering injectable or oral medication); complex health service interventions (e.g. healthcare pathways, models of care); insulin titration (e.g. different protocols for insulin titration); and personalised risk assessment or use of patient-reported outcome measures (PROMs) to assess outcomes in care. The miscellaneous interventions category was further subcategorised if ten or more studies reported on the same type of intervention.

### Study screening

After deduplication, two independent reviewers screened the titles and abstracts of all studies identified in the literature search in duplicate using Covidence software [20]. Subsequently, full-text versions of the potentially eligible publications were retrieved (by contacting corresponding authors if necessary) and assessed by two independent reviewers. During the selection process, we did not mask any information about the articles, including the journal of publication, the authors or their affiliated institutions, or the magnitude and direction of the results. Discrepancies were resolved through discussion or, when necessary, by consulting a third reviewer; any remaining uncertainties were addressed in consultation with other authors.

### Data extraction

Data extraction was performed by one reviewer and checked by another reviewer, using a standardised form. Additionally, a third reviewer independently extracted the outcome data from the original articles and compared these with the data extracted by the first two reviewers. In the event of multiple publications from the same trial, we maximised the yield of information by collating all available data, and used the most complete dataset aggregated across all related publications. In general, we gave priority to the publication with the longest follow-up period. Consequently, not all publications reporting different follow-up durations from the same trials are listed in the reference list, as data were consolidated and represented by the most comprehensive publication. Extracted data included: (1) trial characteristics (first author, DOI, year of publication, setting, trial identifier, trial duration, duration of intervention, description of intervention and control condition [e.g. content, intensity, the professional involved and delivery methods], geographical location, number of participants); (2) participants’ baseline characteristics (male/female ratio, mean age, mean/median diabetes duration, mean HbA_1c_, type of diabetes); and (3) diabetes distress data for each trial arm, including measurement method and baseline distress score. For diabetes distress at follow-up, the latest available timepoint with complete data was used. For trials with several intervention groups, we included all the intervention and control groups, resulting in trials sometimes being reported more than once in any one analysis (e.g. the study by Quinn et al [21], which compared coach only vs coach with primary care provider portal vs coach with primary care provider portal with decision making vs control to test whether adding mobile application coaching and patient/provider web portals to usual care reduces HbA_1c_ in adults with type 2 diabetes). Any uncertainties identified during data extraction were resolved by consulting a third reviewer or, if necessary, by contacting the corresponding authors for clarification. Additional or missing information was also requested from the corresponding authors by email. When mean data were missing but median data were included, we imputed the means from the median data [19]. When SDs for outcomes were not reported and further information was not received from the corresponding authors, we imputed these values by assuming the SDs of the missing outcomes to be the SDs of the baseline values; when this information was also not reported (*n*=1) [22], we extrapolated SDs from trials with very similar populations and interventions, as suggested in the Cochrane Handbook [19].

Based on the extracted information, we categorised the interventions into one of the predefined categories (psychological, psychoeducational or miscellaneous [with subcategories]). This categorisation was subsequently reviewed and verified by the content and clinical experts of our author group to ensure consistency and appropriateness.

### Risk of bias assessment

Risk of bias was assessed using the Cochrane RoB2 tool for RCTs [19] for the primary outcome diabetes distress. An ‘overall risk of bias’ judgement was made, summarising the score of the five bias domains. The overall risk of bias was considered ‘low’ if an RCT was judged to be at low risk in all domains, and ‘high’ when an RCT was judged to be at high risk in at least one domain or was judged to have some concerns for multiple domains. In all remaining cases, the overall risk of bias was considered to be ‘some concerns for risk of bias’. The risk of bias assessment was performed by one independent reviewer and checked by a second reviewer (FR, SBJS). Discrepancies were resolved through discussion or through consultation with a third reviewer. The review team tested the form for the risk of bias assessment on a pilot sample of five to ten papers that spanned a range from some concerns for risk of bias to high risk of bias, to ensure that the criteria were being applied consistently and consensus could be reached. Reviewers were not blinded to author names, institutions, journal of publication or trial results when assessing methodology for risk of bias. We assessed risk of bias of studies in all three categories of interventions; however, for miscellaneous interventions, we assessed risk of bias only in the subcategories with ten or more studies that reported on the same intervention subtype, namely devices, educational interventions without a psychological component, and peer support without a psychological component. The last subcategory included fewer than ten studies (*n*=7); however, because of the strong overlap with the psychoeducational category based on content, we also assessed these studies.

### Data synthesis

Data from eligible studies were meta-analysed using random-effects models—primarily because of clinical heterogeneity (particularly in terms of the intervention used in each trial) and also because of differences in methodology across studies—when at least two studies reported results for diabetes-related distress within the same predefined intervention category. The analyses were conducted in R version 4.3.2 and the results are displayed in forest plots [23]. As different scales were used to assess diabetes distress, standardised mean differences (SMDs) were calculated. According to the Cochrane Handbook, ‘post-intervention value and change scores should not in principle be combined using standard meta-analysis approaches when the effect measure is an SMD’ [19]. A common rule of thumb is that an SMD <0.40 indicates a small effect, values between 0.40 and 0.70 indicate a moderate effect, and values >0.70 indicate a large effect, as described in the Cochrane Handbook [19]. To support interpretation of the magnitude and clinical relevance of intervention effects for diabetes distress, we defined a threshold for a minimal clinically important difference (MCID) on the SMD scale. Because diabetes distress outcomes were synthesised across multiple validated PROMs using SMDs, and no single anchor-based MCID is applicable across instruments, a pragmatic SMD-based threshold informed by the established interpretation of effect size for patient-reported outcomes and by panel consensus was adopted [24, 25]. Specifically, an SMD of 0.3 was considered to represent an MCID. Based on this framework, effects were categorised as large (SMD ≤ −0.5), moderate (−0.5 < SMD ≤ −0.3), small (−0.3 < SMD ≤ −0.2) and trivial/no effect (SMD > −0.2). This threshold was used to support the Grading of Recommendations Assessment, Development and Evaluation (GRADE) certainty of evidence assessment.

As some included trials were cluster randomised, effect estimates were adjusted for clustering when the intracluster correlation coefficient (ICC) was reported. If the ICC was not available and adjustment was not possible, these studies were excluded in a sensitivity analysis to evaluate their impact on the pooled estimates.

Where a trial reported two interventions vs a control group, we halved the sample size of the control group to avoid double counting. Because of predefined substantial clinical heterogeneity in terms of type of diabetes, we performed separate analyses for type 1 and type 2 diabetes. If a trial recruited a mixed population (both type 1 and type 2 diabetes), as a rule of thumb we included the study in a single group if ≥60% of the participants had that type of diabetes. When the proportion of participants was approximately evenly split between type 1 and type 2 diabetes (50/50) or when the proportions of diabetes types in a mixed population were undefined, we included the trial in both the type 1 and the type 2 diabetes analyses. Analyses were stratified by type of intervention (psychological, psychoeducational or subcategories of miscellaneous interventions), comparator (i.e. usual care and waiting list or active comparator) and tool used to measure diabetes distress (i.e. DDS, PAID, T1-DDS, PAID-5). In two comparisons originally labelled as having ‘miscellaneous’ comparators (i.e. interventions other than psychological or psychoeducational), we re-analysed the data to improve clinical relevance when we observed that >90% of the comparator belonged to one subcategory. We then conducted a sensitivity analysis additionally including the trials with the heterogeneous comparator types (*n*=2). These sensitivity analyses were prioritised in the guideline, while the broader analyses including all miscellaneous comparators are documented in this article for transparency. Heterogeneity between studies was assessed using the *I*^2^ statistic, which quantifies inconsistency across trials; an *I*^2^ statistic of ≥75% indicates a considerable level of heterogeneity [19].

We performed subgroup analyses based on: (1) risk of bias score; (2) intervention delivery format (i.e. one-to-one vs group based; in person vs online); (3) intervention duration (using the median duration of all included trials within each intervention category as the threshold for subgrouping); and (4) level of diabetes distress at baseline (high diabetes distress defined as DDS≥3, PAID or PAID20≥40, PAID11≥18, PAID8≥13, PAID5≥8) to assess possible explanations for heterogeneity. When three trials or more investigated a particular comparison, we used funnel plots and the Egger test to assess small study effects. If meta-analysis was not possible, we present a narrative summary of the eligible trials.

### Certainty of evidence assessment

We assessed the overall certainty of the evidence on diabetes distress according to the GRADE approach [26], which considers issues in the domains of risk of bias, inconsistency, imprecision and indirectness. Two authors (TK, FZ) independently rated the certainty of evidence using the GRADEpro online tool [27], and any discrepancies were resolved through consensus with all authors.

## Results

### Characteristics of included trials

**Fig. 1:**
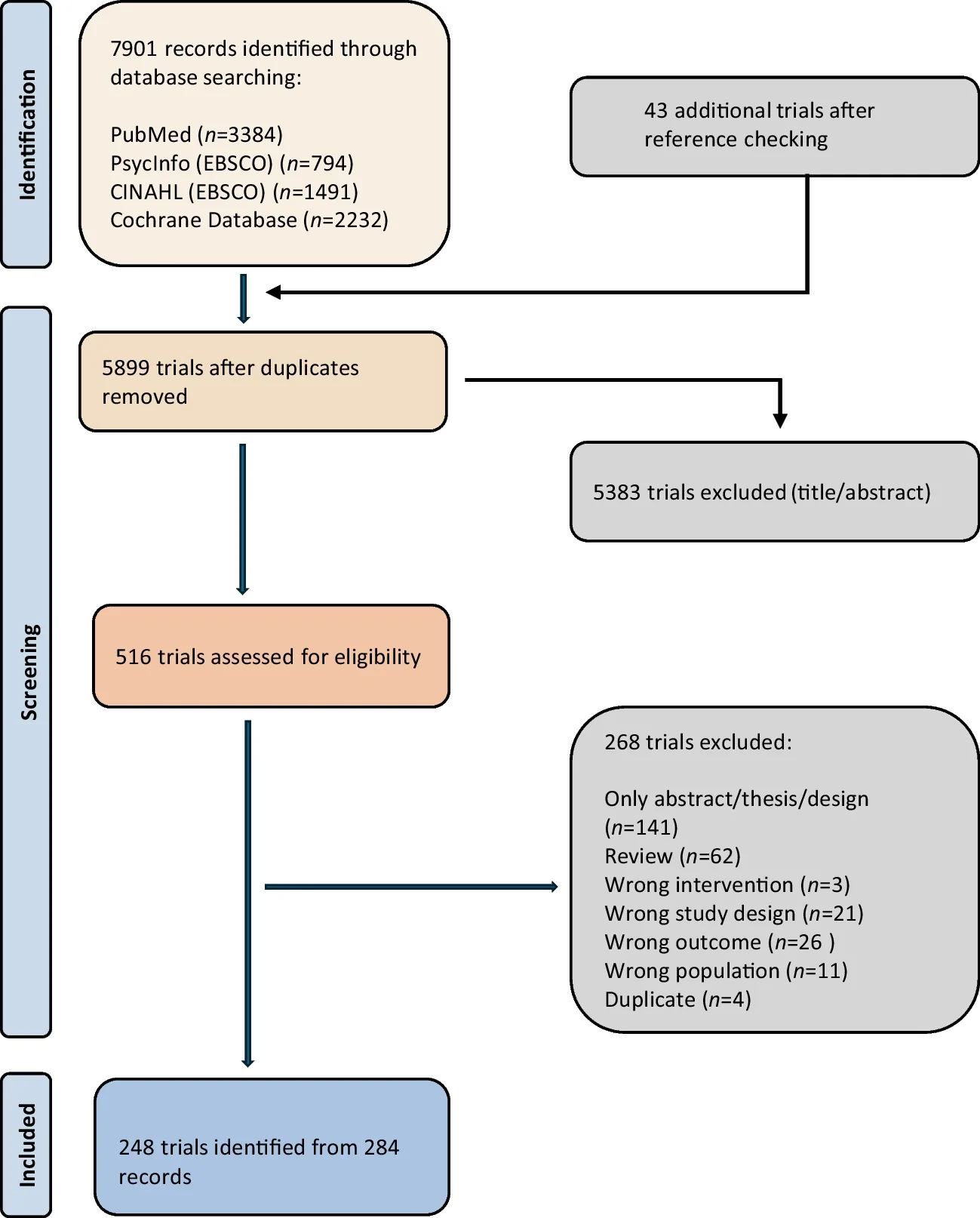
PRISMA flow diagram

As shown in the PRISMA flow diagram (Fig. 1), 284 records reporting on 248 RCTs met the inclusion criteria for the systematic review. Reasons for exclusion are provided in ESM 2. The mean kappa score for full-text screening was 0.87, which suggests almost perfect agreement. We categorised 59 trials as investigating psychological interventions, 68 as investigating psychoeducational interventions and 121 as investigating miscellaneous (neither psychological nor psychoeducational) interventions. In total, 27 trials were related to devices (for the administration of glucose-lowering medications or for glucose monitoring or for both); 15 involved peer support without a psychological component; 10 involved education without a psychological component; 23 were telehealth interventions (e.g. apps, online consultations); 21 were health behaviour interventions (e.g. targeting sleep, weight loss, diet); and 25 included a diverse selection of interventions (fewer than ten per group) that encompassed complex health service interventions (e.g. healthcare pathways, models of care), insulin titration, medication, PROMs, surgery and personalised risk assessment.

An overview of the characteristics of the included trials is shown in ESM 3. Of the 248 trials, 52 included participants with type 1 diabetes, of which two were conducted with pregnant women; 160 included participants with type 2 diabetes; and 36 included a combined population. All trials were RCTs, of which 13 were crossover RCTs and 22 were cluster RCTs. All trials measured diabetes distress with either (variations of) the PAID scale (138 trials) or (variations of) the DDS scale (107 trials) or with both (three trials). The total sample size was 55,497 participants, with the sample size in individual trials ranging from 12 to 4034.

### Risk of bias assessment

An overview of the methodological quality assessment of the included trials is presented in the RoB2 summary table in ESM 4. The methodological quality of the trials was rated as some concerns for risk of bias in 94 trials and high risk of bias in 71 trials. Most of the concerns for risk of bias were due to a high risk of selection bias and reporting bias. Risk of bias assessment was performed only for studies included in the meta-analysis and systematic review within each intervention category, and not for all identified trials.

### Meta-analysis results

Most studies did not report baseline values or change from baseline values and/or respective measures of dispersion for these values. Therefore, we were only able to conduct meta-analyses on post-intervention (endpoint) values.

#### Psychological interventions

##### Psychological interventions compared with psychoeducational interventions for type 1 diabetes

One trial compared a psychological intervention with a psychoeducational intervention in adults with type 1 diabetes [28]. Narrative analysis showed a non-significant reduction in diabetes distress in favour of cognitive behavioural therapy compared with blood glucose awareness training [28]. Detailed effect estimates for all analyses are provided in Table 1.

**Table 1:** Summary of findings

Comparison	Effect estimate, SMD (95% CI)	No. of studies	Subgroup differences
Type 1 diabetes			
Psychological vs psychoeducational interventions	X	X	X
Psychological vs educational interventions	−0.11 (−0.44, 0.23)	2	X
Psychological interventions vs usual care	−0.21 (−0.34, −0.08)*	16	No
Psychoeducational vs educational interventions	−0.02 (−0.23, 0.20)	3	No
Psychoeducational interventions vs usual care	−0.17 (−0.62, 0.28)	2	X
Educational vs peer support interventions	X	X	X
Peer support interventions vs usual care	X	X	X
Educational interventions vs usual care	X	X	X
AID systems vs no AID	−0.15 (−0.33, 0.02)	5	No
CGM/intermittently scanned glucose monitoring vs capillary glucose monitoring	−0.26 (−0.41, −0.11)*	6	No
Type 2 diabetes			
Psychological vs psychoeducational interventions	X	X	X
Psychological vs educational interventions	−0.29 (−0.98, 0.39)	10	No
Psychological interventions vs usual care	−0.31 (−0.50, −0.13)*	21	No
Psychoeducational vs educational interventions	−0.15 (−0.34, 0.03)	13	No
Psychoeducational interventions vs usual care	−0.19 (−0.40, 0.02)	23	No
Peer support vs educational interventions	0.10 (−0.08, 0.28)	3	No
Educational vs miscellaneous interventions	X	X	X
Peer support interventions vs usual care	−0.03 (−0.19, 0.13)	4	No
Educational interventions vs usual care	−0.34 (−0.56, −0.11)*	3	No
CGM vs capillary glucose monitoring	−0.35 (−0.98, 0.28)	3	No

^*^p<0.05

AID, automated insulin delivery; CGM, continuous glucose monitoring; No, subgroup analyses revealed no significant subgroup effects based on baseline diabetes distress score, trial duration, delivery format or risk of bias score; SMD, standardised mean difference; X, no meta-analysis conducted

##### Psychological interventions compared with educational interventions for type 1 diabetes

**Fig. 2:**
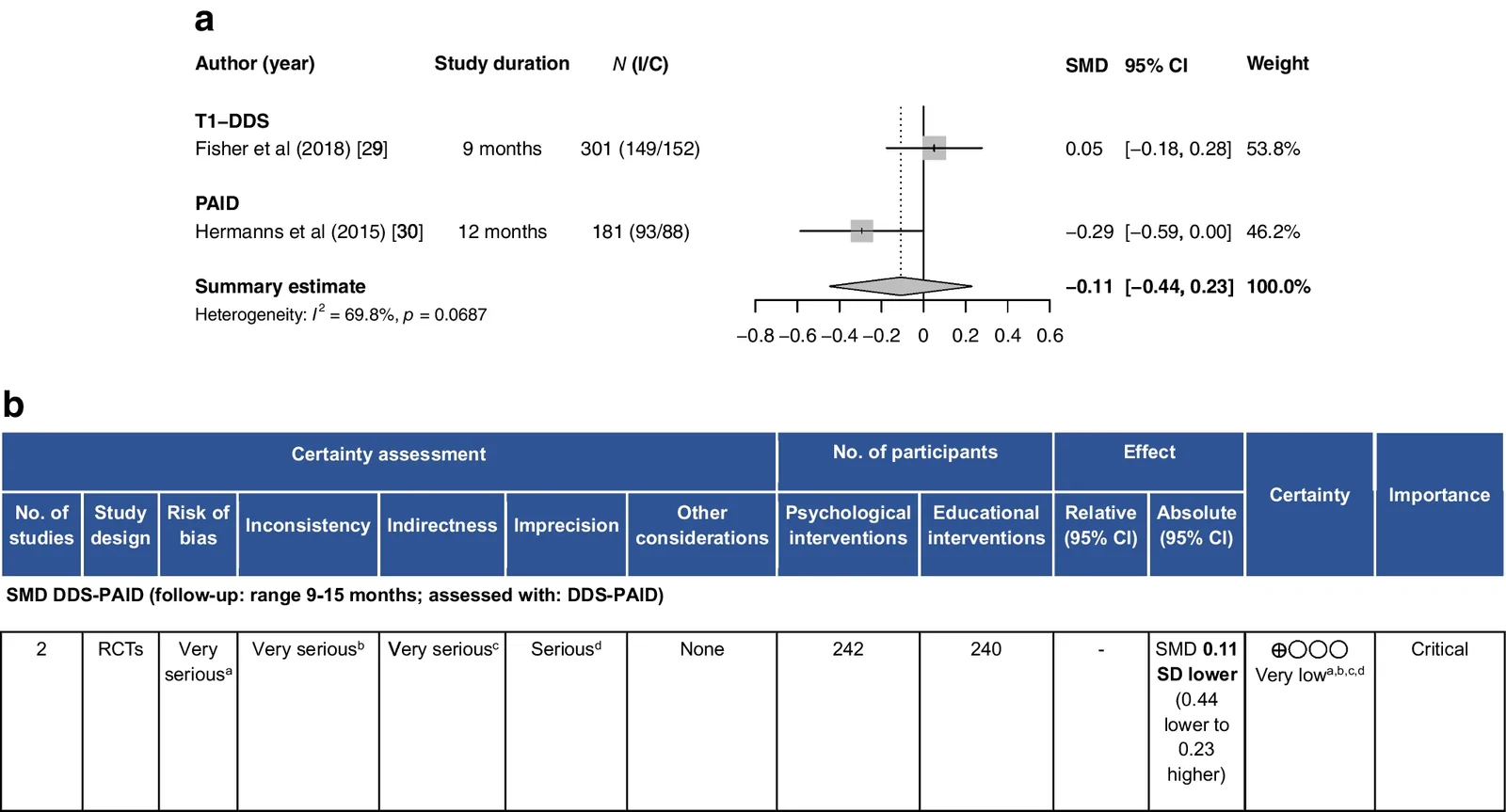
(**a**) SMD for psychological interventions compared with educational interventions without a psychological component in type 1 diabetes. C, control; I, intervention. (**b**) GRADE evidence profile for psychological interventions compared with educational interventions without a psychological component in type 1 diabetes. ^a^One study at high risk of bias and one with some concerns. ^b^*I*^2^=69.8% and effect not large. ^c^Different types of comparators. ^d^95% CI crosses both –0.3 and null but not 0.3

Two trials compared psychological interventions with educational interventions (without a psychological component) in type 1 diabetes [29, 30]. Meta-analysis showed that the difference in diabetes distress between psychological and educational interventions was statistically non-significant (−0.11 [95% CI −0.44, 0.23]) (Fig. 2a) [29, 30]. The respective GRADE evidence profile is shown in Fig. 2b. No subgroup analyses were conducted as only two trials were included in this comparison.

##### Psychological interventions compared with usual care for type 1 diabetes

**Fig. 3:**
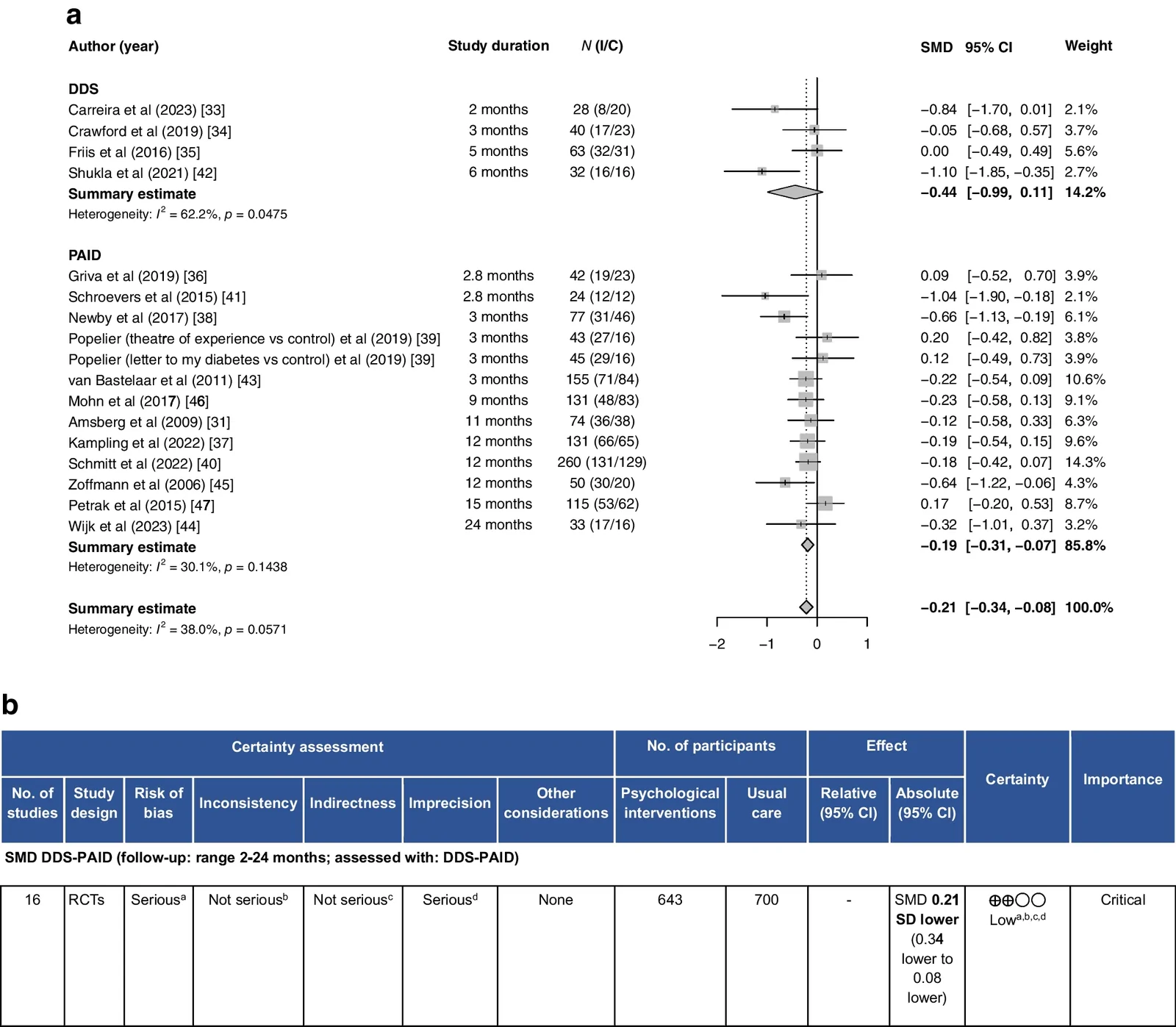
(**a**) SMD for psychological interventions compared with usual care in type 1 diabetes. C, control; I, intervention. (**b**) GRADE evidence profile for psychological interventions compared with usual care in type 1 diabetes. ^a^Most studies had some concerns. ^b^*I*^2^=38%. ^c^Variations in the types of interventions not considered important enough to downgrade. ^d^95% CI crosses –0.3 but not null

In total, 17 trials compared psychological interventions with usual care in adults with type 1 diabetes [31–47]. Meta-analysis of 16 trials (17 comparisons) showed a significant reduction in diabetes distress (−0.21 [95% CI −0.34, −0.08]) favouring psychological interventions (Fig. 3a) [31, 33–47]. The respective GRADE evidence profile is shown in Fig. 3b. Subgroup analyses revealed no significant subgroup effects by baseline diabetes distress, trial duration, intervention delivery format or risk of bias score (*p*>0.05) (ESM 5, Fig. 3c–f). Narrative analysis of the study that could not be included in the meta-analysis showed an effect consistent with the meta-analysis of psychological interventions compared with usual care for reducing diabetes distress [32].

##### Psychological interventions compared with psychoeducational interventions for type 2 diabetes

One trial compared a psychological intervention with a psychoeducational intervention in adults with type 2 diabetes [48]. Narrative analysis showed a non-significant effect in favour of a mindfulness intervention integrated into diabetes self-management education (DSME) compared with DSME alone for the reduction of diabetes distress [48].

##### Psychological interventions compared with educational interventions for type 2 diabetes

**Fig. 4:**
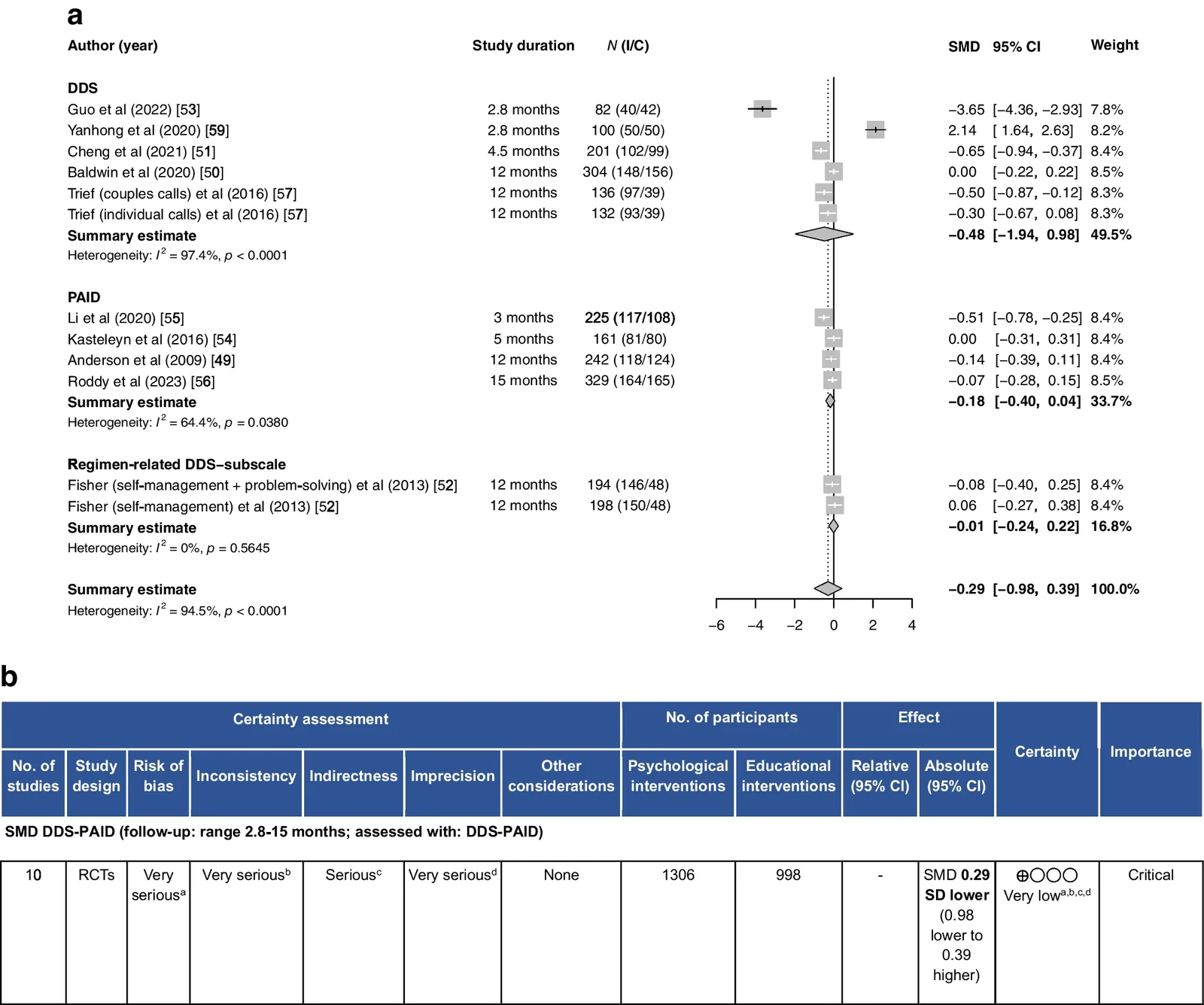
(**a**) SMD for psychological interventions compared with educational interventions in type 2 diabetes. C, control; I, intervention. (**b**) GRADE evidence profile for psychological interventions compared with educational interventions in type 2 diabetes. ^a^Most studies at high risk of bias. ^b^*I*^2^=94.5%. ^c^Different comparators; subgroup effect among outcome scales. ^d^95% CI crosses –0.3 and 0.3

Eleven trials compared psychological interventions with a set of miscellaneous interventions in adults with type 2 diabetes [49–59]. Of these, ten trials compared a psychological intervention with an educational intervention (without a psychological component); one trial that compared written emotional disclosure with neutral writing was not included in the main analyses to maintain comparability across studies [58]. Of note, a sensitivity analysis including this trial did not show significant differences (ESM 5, Fig. 4g). Meta-analysis of the ten trials (12 comparisons) showed a statistically non-significant effect on reduction of diabetes distress favouring psychological interventions compared with educational interventions (without a psychological component) (−0.29 [95% CI −0.98, 0.39]) (Fig. 4a) [49–57, 59]. The respective GRADE evidence profile is shown in Fig. 4b. Additional subgroup analyses revealed no significant subgroup effects by baseline diabetes distress, trial duration, intervention delivery format or risk of bias score (*p*>0.05) (ESM 5, Fig. 4c–f).

##### Psychological interventions compared with usual care for type 2 diabetes

**Fig. 5:**
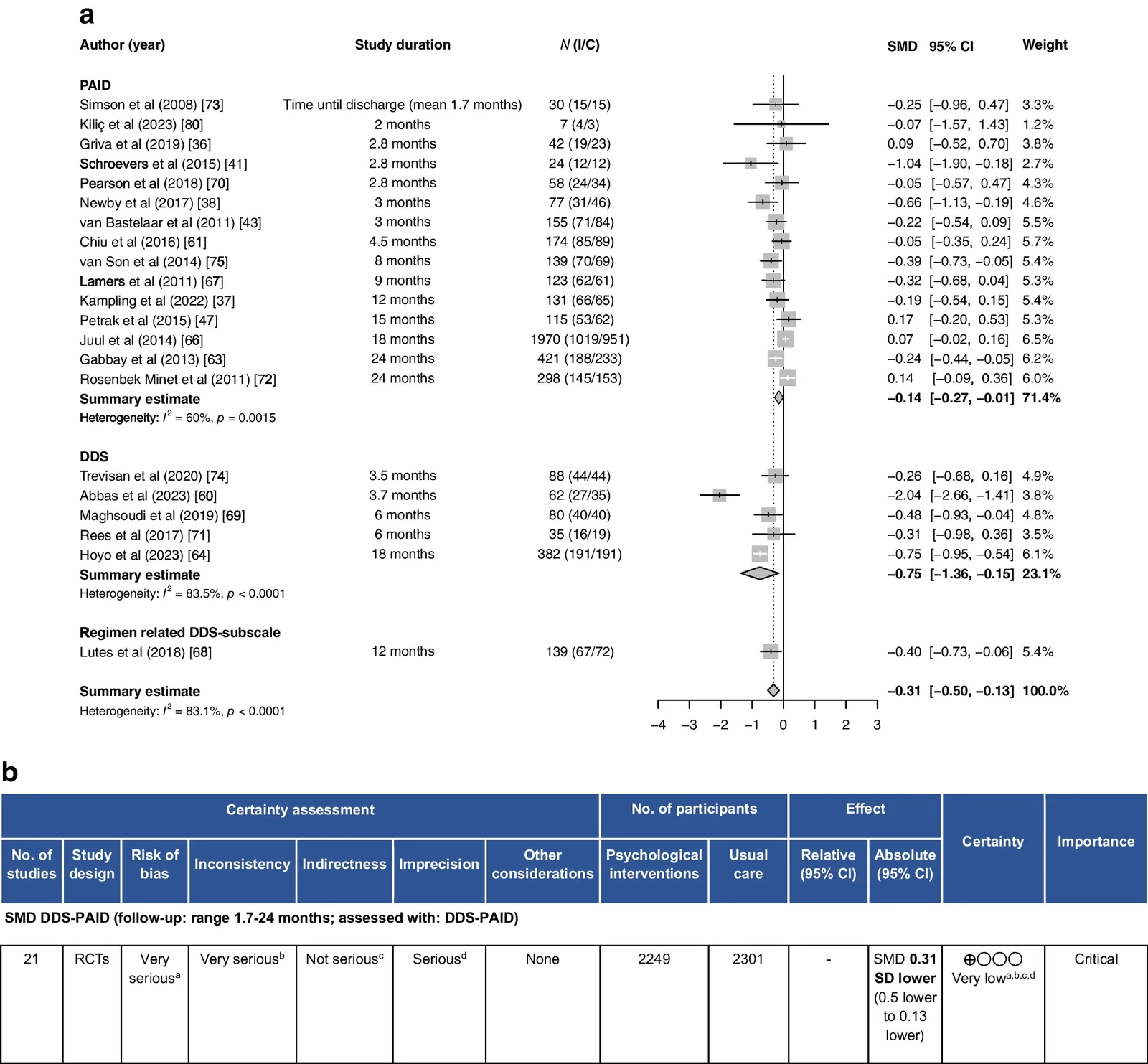
(**a**) SMD for psychological interventions compared with usual care in type 2 diabetes. C, control; I, intervention. (**b**) GRADE evidence profile for psychological interventions compared with usual care in type 2 diabetes. ^a^Many studies at high risk of bias. ^b^*I*^2^=83.1%. ^c^Variations in the types of interventions not considered important enough to downgrade. ^d^95% CI crosses 0.3 but not null

Twenty-seven trials compared psychological interventions with usual care in adults with type 2 diabetes [36–38, 41, 43, 47, 60–80]. Of these, 21 trials were included in the meta-analysis, in which a significant effect in favour of psychological interventions was found for the reduction of diabetes distress (−0.31 [95% CI −0.50, −0.13]) (Fig. 5a) [36–38, 41, 43, 47, 60, 61, 63, 64, 66–75, 80]. The respective GRADE evidence profile is shown in Fig. 5b. Subgroup analyses revealed no significant subgroup effects by baseline diabetes distress, trial duration, intervention delivery format or risk of bias score (*p*>0.05) (ESM 5, Fig. 5c–f). Narrative analyses of the six trials that could not be included in the meta-analysis showed effects of psychological interventions consistent with those observed in the meta-analysis for the reduction of diabetes distress [62, 65, 76–79].

#### Psychoeducational interventions

##### Psychoeducational interventions compared with educational interventions for type 1 diabetes

**Fig. 6:**
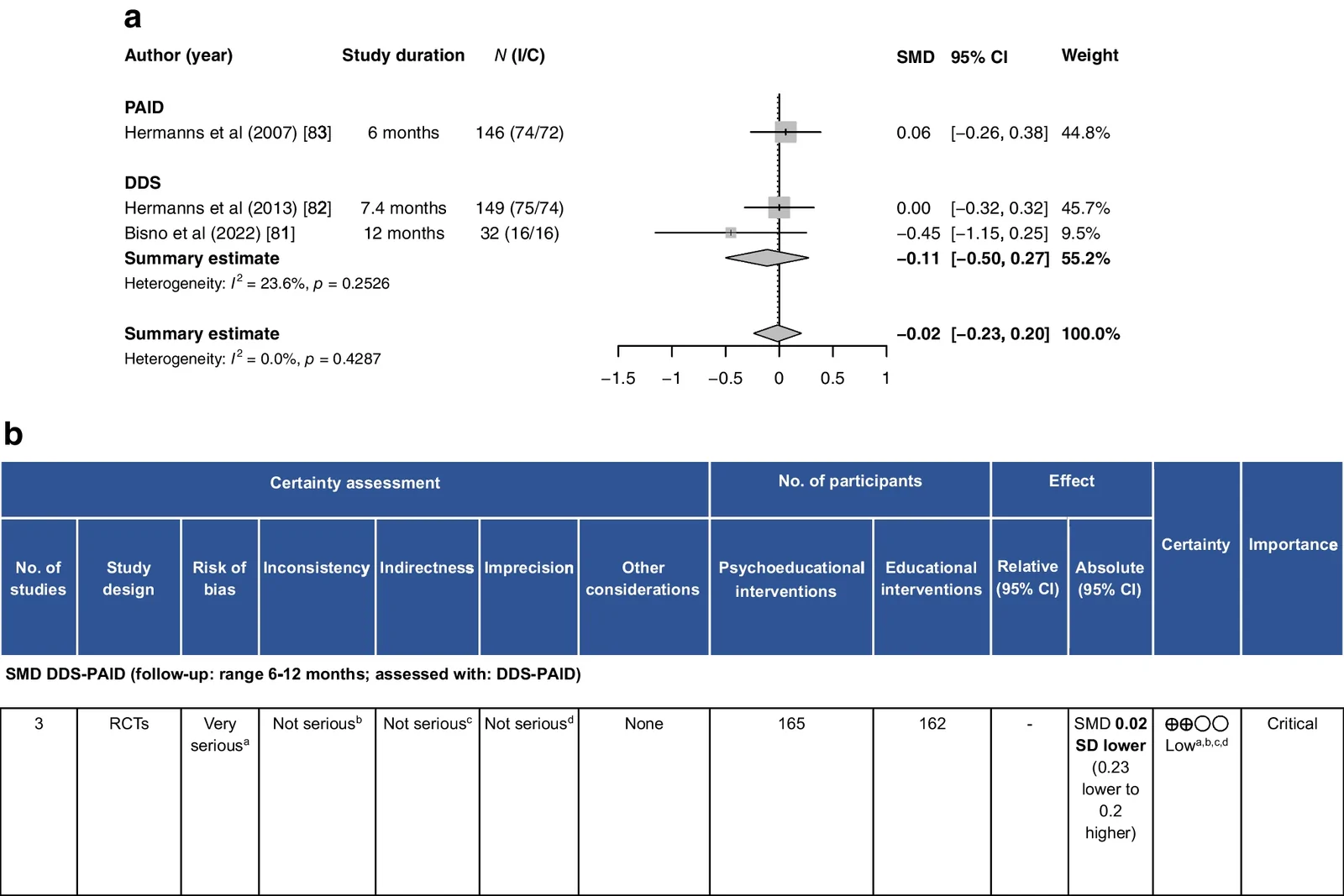
(**a**) SMD for psychoeducational interventions compared with educational interventions in type 1 diabetes. C, control; I, intervention. (**b**) GRADE evidence profile for psychoeducational interventions compared with educational interventions in type 1 diabetes. ^a^Most studies at high risk of bias. ^b^*I*^2^=0%. ^c^Different types of interventions and comparators. ^d^95% CI crosses null but not –0.3 or 0.3

Four trials compared psychoeducational interventions with educational interventions (without a psychological component) in adults with type 1 diabetes [81–84]. Meta-analysis of three trials showed a non-significant effect in favour of psychoeducational interventions (−0.02 [95% CI −0.23, 0.20]) for the reduction of diabetes distress (Fig. 6a) [81–83]. The respective GRADE evidence profile is shown in Fig. 6b. Subgroup analyses revealed no significant subgroup effects by trial duration or risk of bias score (*p*>0.05) (ESM 5, Fig. 6c, d). Narrative analysis of the trial that could not be included in the meta-analysis showed a similar effect, favouring the psychoeducational intervention [84].

##### Psychoeducational interventions compared with usual care for type 1 diabetes

**Fig. 7:**
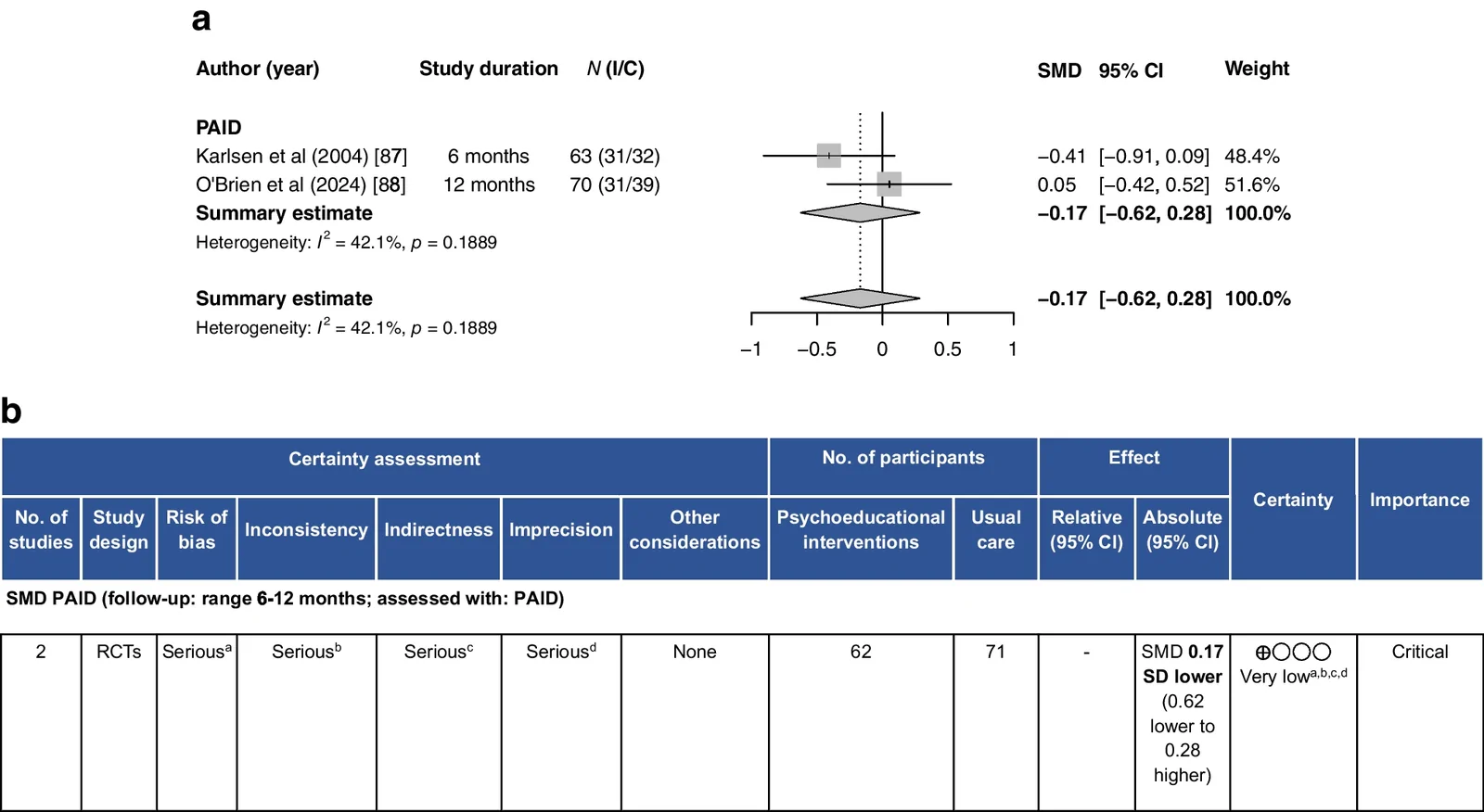
(**a**) SMD for psychoeducation interventions compared with usual care in type 1 diabetes. C, control; I, intervention. (**b**) GRADE evidence profile for psychoeducational interventions compared with usual care in type 1 diabetes. ^a^Some concerns in both studies. ^b^*I*^2^=42.1% and effect not large. ^c^Some variations in types of interventions. ^d^95% CI crosses –0.3 and null but not 0.3

Six trials compared psychoeducational interventions with usual care in adults with type 1 diabetes [85–90]. Meta-analysis of two trials showed a non-significant effect in favour of psychoeducational interventions (−0.17 [95% CI −0.62, 0.28] for the reduction of diabetes distress (Fig. 7a) [87, 88]. The respective GRADE evidence profile is shown in Fig. 7b. No subgroup analyses were conducted, as only two trials were included in the meta-analysis. Narrative analysis of the other four trials also showed a small non-significant effect in favour of psychoeducational interventions for the reduction of diabetes distress [85, 86, 89, 90].

##### Psychoeducational interventions compared with educational interventions in type 2 diabetes

**Fig. 8:**
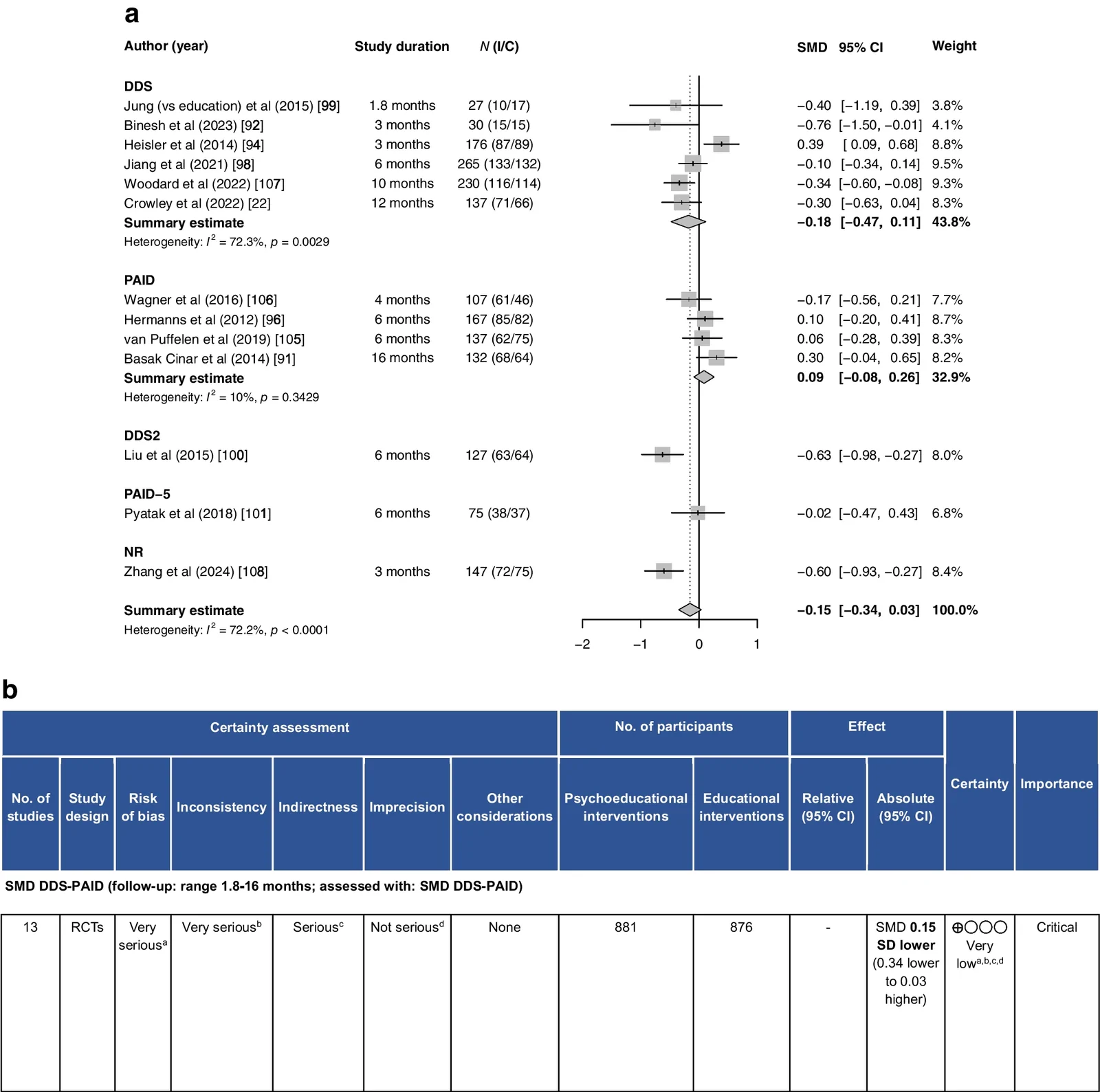
(**a**) SMD for psychoeducational interventions compared with educational interventions in type 2 diabetes. C, control; I, intervention; NR, not reported. (**b**) GRADE evidence profile for psychoeducational interventions compared with educational interventions in type 2 diabetes. ^a^Almost half of studies at high risk of bias. ^b^*I*^2^=72.2%. ^c^Different types of interventions and comparators. ^d^95% CI marginally crosses –0.3 and null

Twenty trials compared psychoeducational interventions with miscellaneous interventions in adults with type 2 diabetes [22, 84, 91–108]. Meta-analysis of 13 of these trials comparing psychoeducational interventions with educational interventions (without a psychological component) showed a marginally non-significant effect (−0.15 [95% CI −0.34, 0.03]) in favour of psychoeducational interventions for reducing diabetes distress (Fig. 8a) [22, 91, 92, 94, 96, 98–101, 105–108].The respective GRADE evidence profile is shown in Fig. 8b. A sensitivity meta-analysis of the same 13 trials including an extra comparator arm (with a health behaviour intervention, i.e. walking) in one trial showed a similar effect (ESM 5, Fig. 8g). Additionally, excluding cluster RCTs without a reported ICC also yielded similar results (ESM 5, Fig. 8h). Subgroup analyses revealed no significant subgroup effects by baseline diabetes distress, trial duration, intervention delivery format or risk of bias score (*p*>0.05) (ESM 5, Fig. 8c-f). Narrative analysis of the seven trials that could not be included in the meta-analysis showed similar effects as the meta-analysis on the reduction of diabetes distress [84, 93, 95, 97, 102–104].

##### Psychoeducational interventions compared with usual care in type 2 diabetes

**Fig. 9:**
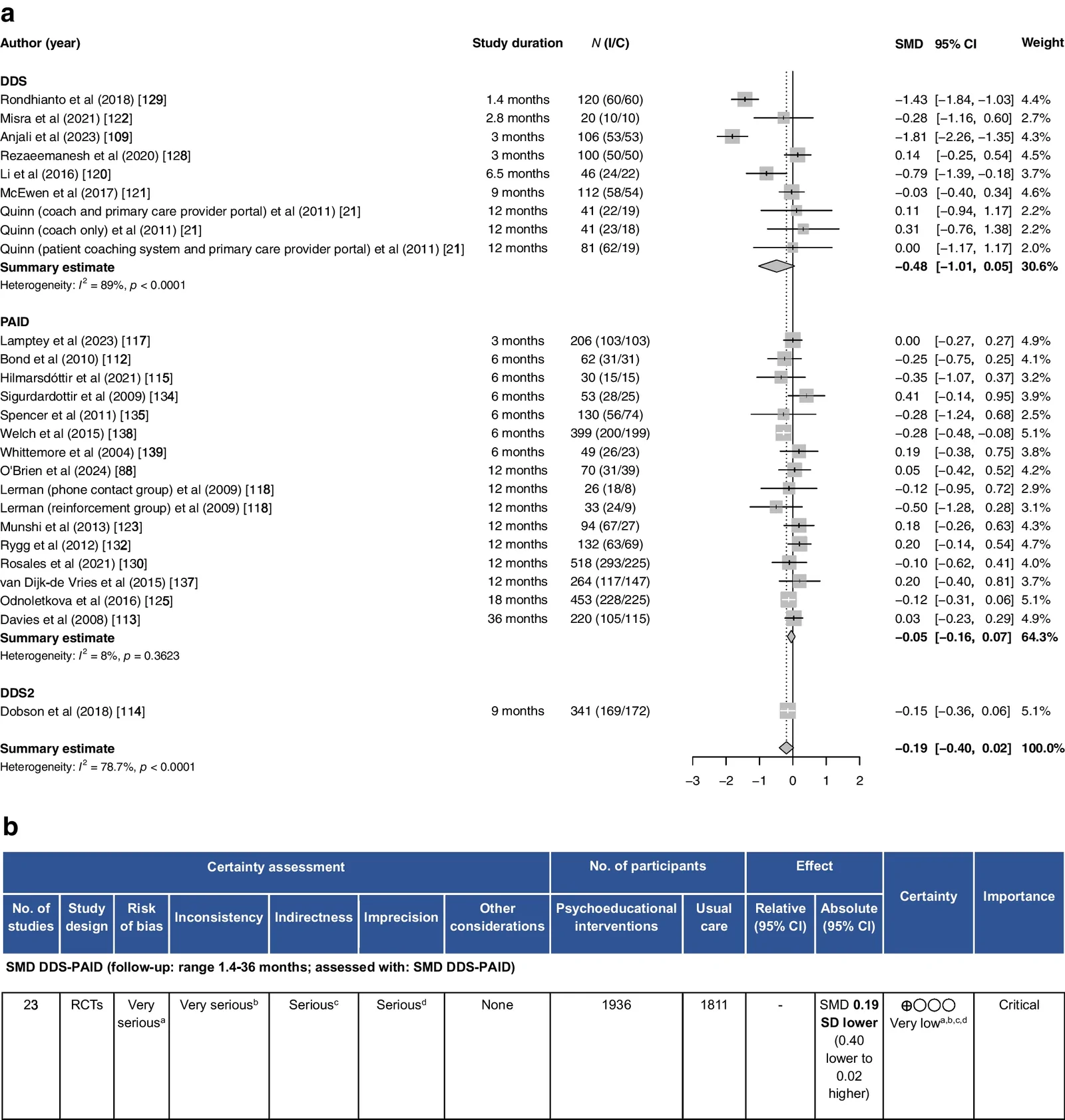
(**a**) SMD for psychoeducational interventions compared with usual care in type 2 diabetes. C, control; I, intervention. (**b**) GRADE evidence profile for psychoeducational interventions compared with usual care in type 2 diabetes. ^a^Almost half of studies were at high risk of bias. ^b^*I*^2^=78.7%. ^c^Different types of interventions. ^d^95% CI crosses –0.3 and null but not 0.03

Thirty-six trials compared psychoeducational interventions with usual care in adults with type 2 diabetes [21, 87–89, 109–140]. Of these, 23 trials were included in the meta-analysis, including 26 comparisons, with a marginally non-significant effect (−0.19 [95% CI −0.40, 0.02]) in favour of psychoeducational interventions for reducing diabetes distress (Fig. 9a) [21, 88, 109, 112–115, 117, 118, 120–123, 125, 128–130, 132, 134, 135, 137–139]. The respective GRADE evidence profile is shown in Fig. 9b. Subgroup analyses revealed no significant subgroup effects by baseline diabetes distress, trial duration, intervention delivery format or risk of bias score (*p*>0.05) (ESM 5, Fig. 9c–f). Narrative analysis of the 13 trials that could not be included in the meta-analysis showed similar effects on reduction of diabetes distress.

#### Miscellaneous interventions

Because of the high clinical heterogeneity of the miscellaneous interventions, even among the same type of subcategory, we were only able to synthesise studies that assessed devices, peer support and educational interventions.

##### Devices in type 1 diabetes

Twenty-one trials compared different methods of glucose monitoring and insulin delivery devices in adults with type 1 diabetes, including self-monitoring of blood glucose, intermittently scanned glucose monitoring, continuous glucose monitoring (CGM) and automated insulin delivery (AID). These included trials on use of AID compared with no use of AID [141–147], CGM compared with other types of CGM or intermittently scanned glucose monitoring [148–151], and CGM/intermittently scanned glucose monitoring compared with capillary glucose monitoring [152–161].

##### AID compared with no use of AID in type 1 diabetes

**Fig. 10:**
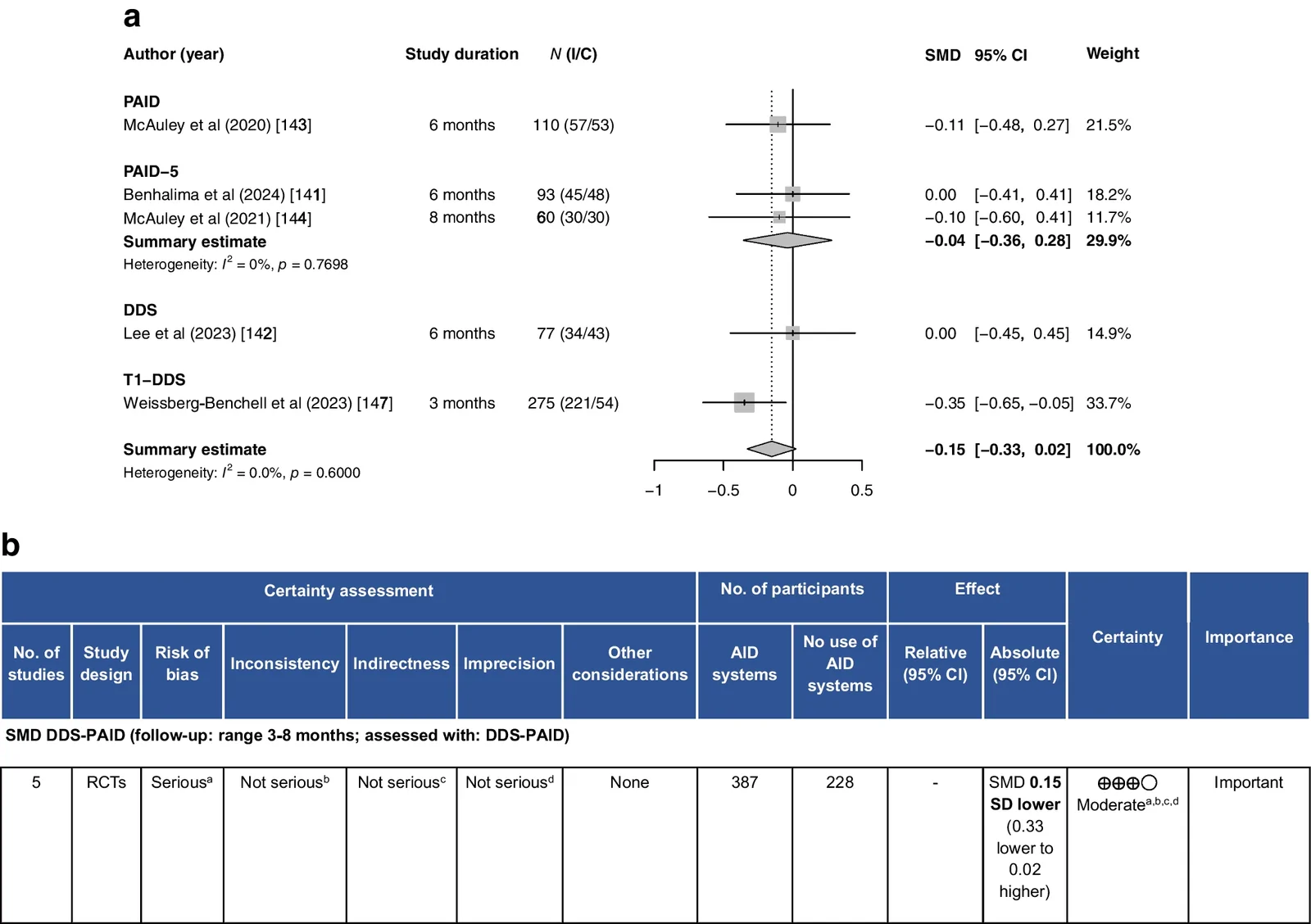
(**a**) SMD for AID compared with no use of AID in type 1 diabetes. C, control; I, intervention. (**b**) GRADE evidence profile for AID compared with no use of AID in type 1 diabetes. ^a^Studies had some concerns. ^b^*I*^2^=0.0%. ^c^Not important variations in terms of types of interventions or comparators. ^d^95% CI crosses –0.3 and marginally crosses null (0.02)

Seven trials compared AID with no use of AID in type 1 diabetes [141–147]. Meta-analysis of five of these trials showed a non-significant effect (−0.15 [95% CI −0.33, 0.02]) in favour of AID for reducing diabetes distress in type 1 diabetes (Fig. 10a) [141–144, 147]. The respective GRADE evidence profile is shown in Fig. 10b. Subgroup analyses revealed no significant subgroup effects by baseline diabetes distress or trial duration (*p*>0.05) (ESM 5, Fig. 10c, d). Narrative analysis of the other two trials showed that one trial was in favour of AID and one was in favour of no use of AID [145, 146].

##### CGM compared with other types of CGM or intermittently scanned monitoring in type 1 diabetes

Narrative analysis of four trials showed no significant differences in diabetes distress between implanted glucose sensors and wearable devices; access to live CGM data and retrospective access; CGM with hypoglycaemia alerts and CGM without hypoglycaemia alerts; or CGM and intermittently scanned glucose monitoring devices in type 1 diabetes [148–151].

##### Continuous/intermittently scanned glucose monitoring compared with standard blood glucose measurement for type 1 diabetes

**Fig. 11:**
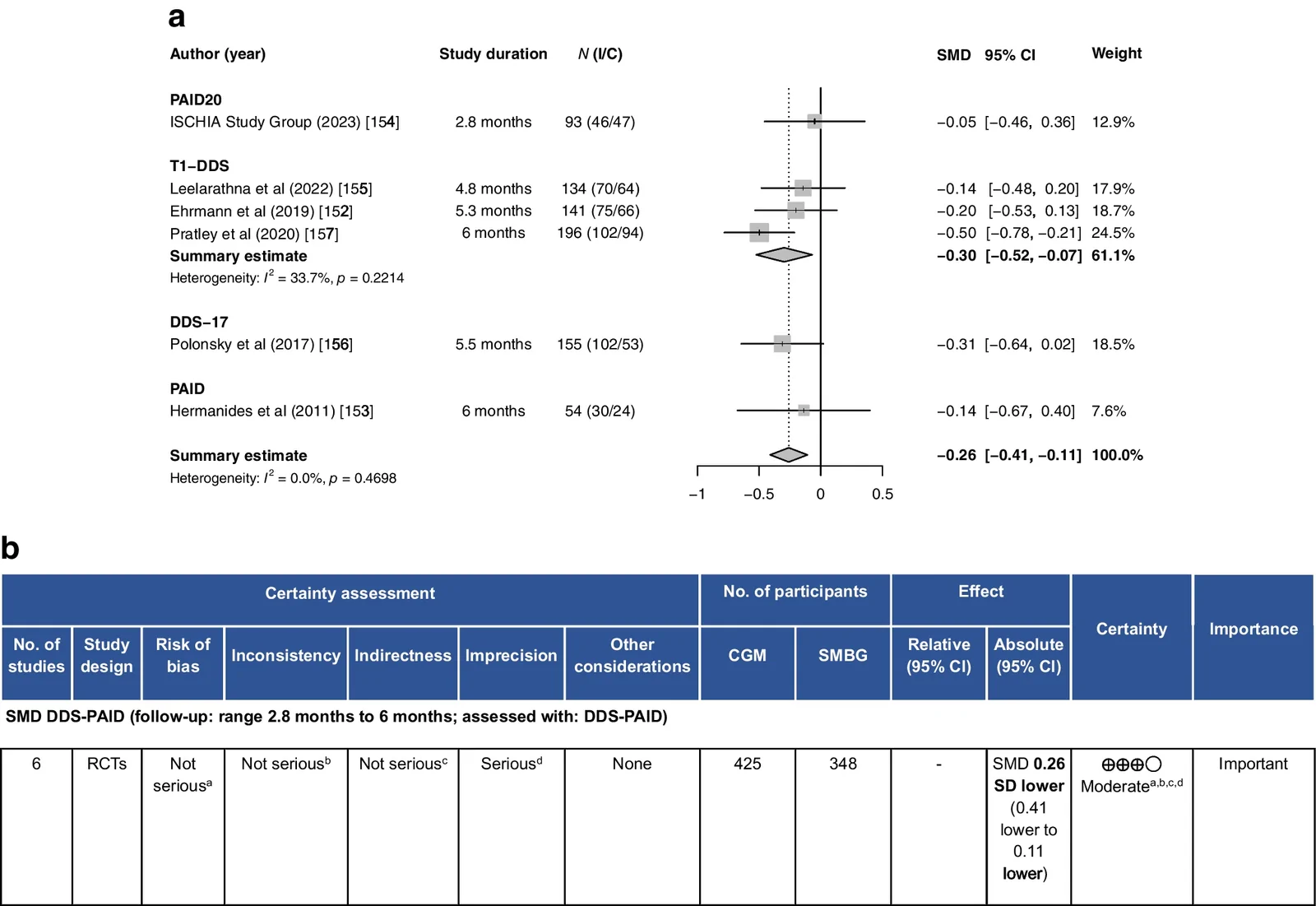
(**a**) SMD for CGM/intermittently scanned glucose monitoring compared with capillary glucose monitoring in type 1 diabetes. C, control; I, intervention. (**b**) GRADE evidence profile for CGM/intermittently scanned glucose monitoring compared with capillary glucose monitoring in type 1 diabetes. ^a^Some trials had some concerns. ^b^*I*^2^=0.0%. ^c^Not important variations in terms of types of interventions or comparators. ^d^95% CI crosses –0.3 but not null. SMBG, self-monitoring of blood glucose

Ten trials compared CGM or intermittently scanned glucose monitoring with capillary glucose monitoring [152–161]. Meta-analysis of six trials [152–157] showed a significant effect (−0.26 [95% CI −0.41, −0.11]) in favour of CGM or intermittently scanned CGM for reducing diabetes distress in type 1 diabetes (Fig. 11a) [152–157]. The respective GRADE evidence profile is shown in Fig. 11b. Subgroup analyses revealed no significant subgroup effects by baseline diabetes distress, intervention delivery format or risk of bias score for both analyses (*p*>0.05) (ESM 5, Fig. 11c–e). Narrative analysis of the four trials on CGM or intermittently scanned CGM that could not be included in the meta-analysis showed similar effects on reduction of diabetes distress [158–161].

##### Devices in type 2 diabetes

**Fig. 12:**
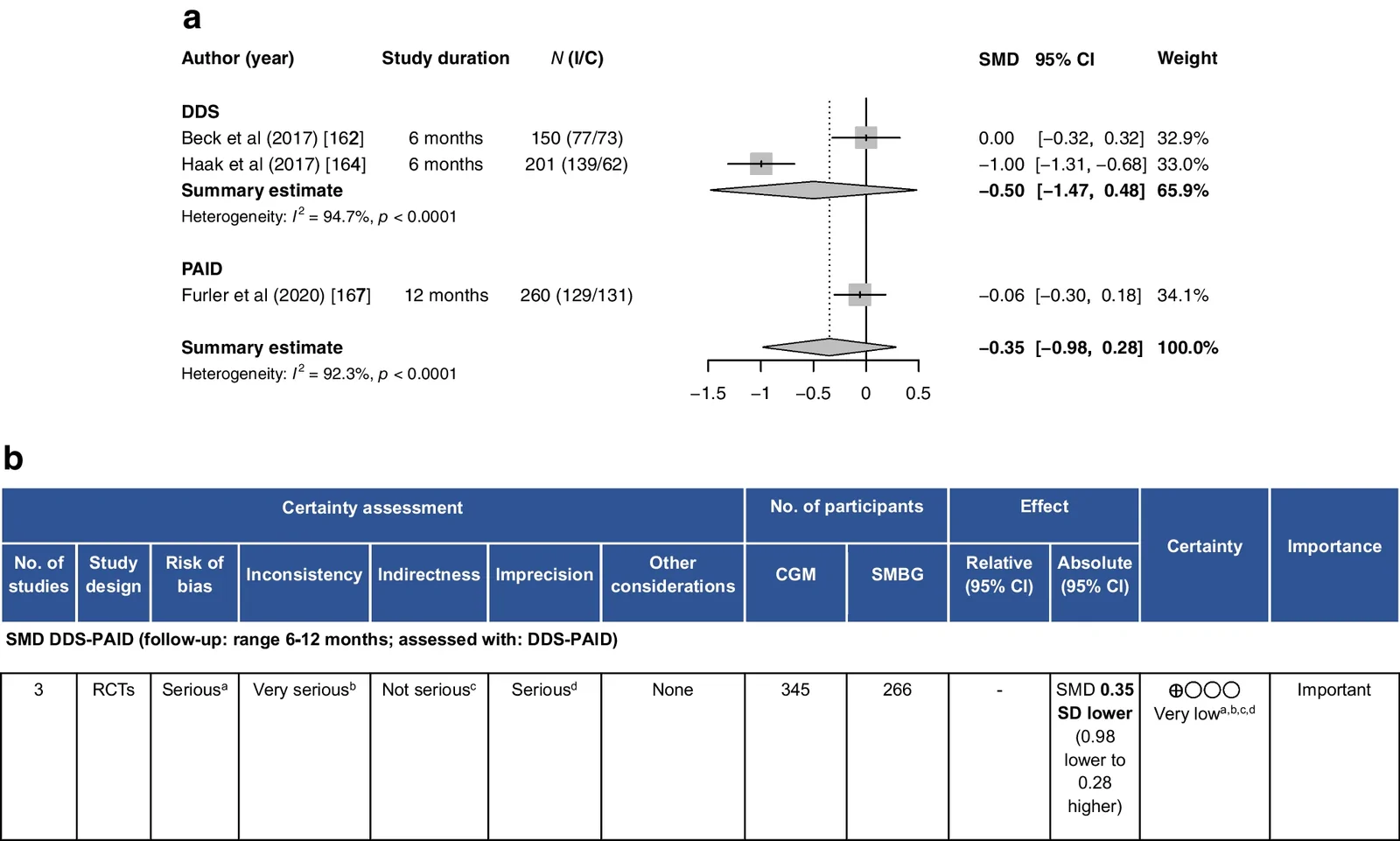
(**a**) SMD for CGM compared with capillary glucose monitoring in type 2 diabetes. C, control; I, intervention. (**b**) GRADE evidence profile for CGM compared with capillary glucose monitoring in type 2 diabetes. ^a^One trial had a high risk of bias and two had some concerns. ^b^*I*^2^=92.3%. ^c^Not important variations in terms of types of interventions or comparators. ^d^95% CI crosses –0.3 and null. SMBG, self-monitoring of blood glucose

Six trials compared devices with usual care in adults with type 2 diabetes [162–167]. Meta-analysis of three of these trials showed a non-significant effect (−0.35 [95% CI −0.98, 0.28]) for the use of CGM compared with capillary glucose monitoring for reducing diabetes distress in type 2 diabetes (Fig. 12a) [162, 164, 167]. The respective GRADE evidence profile is shown in Fig. 12b. No significant subgroup effects were found for risk of bias assessment or trial duration. Sensitivity analyses excluding cluster RCTs without a reported ICC yielded similar results. Narrative analysis of the other trials on CGM or intermittently scanned glucose monitoring that could not be included in the meta-analysis showed similar effects as the meta-analysis on the reduction of diabetes distress [163, 165, 166] (ESM 5, Fig. 12c–e).

##### Peer support interventions compared with usual care for type 1 diabetes

Two trials compared peer support (without a psychological component) with usual care in adults with type 1 diabetes [168, 169]. Narrative analysis showed no differences in the reduction of diabetes distress when reciprocal peer support was compared with nurse care management or when peer leader-led diabetes self-management support was compared with traditional self-management support [168, 169].

##### Peer support interventions compared with educational interventions for type 2 diabetes

**Fig. 13:**
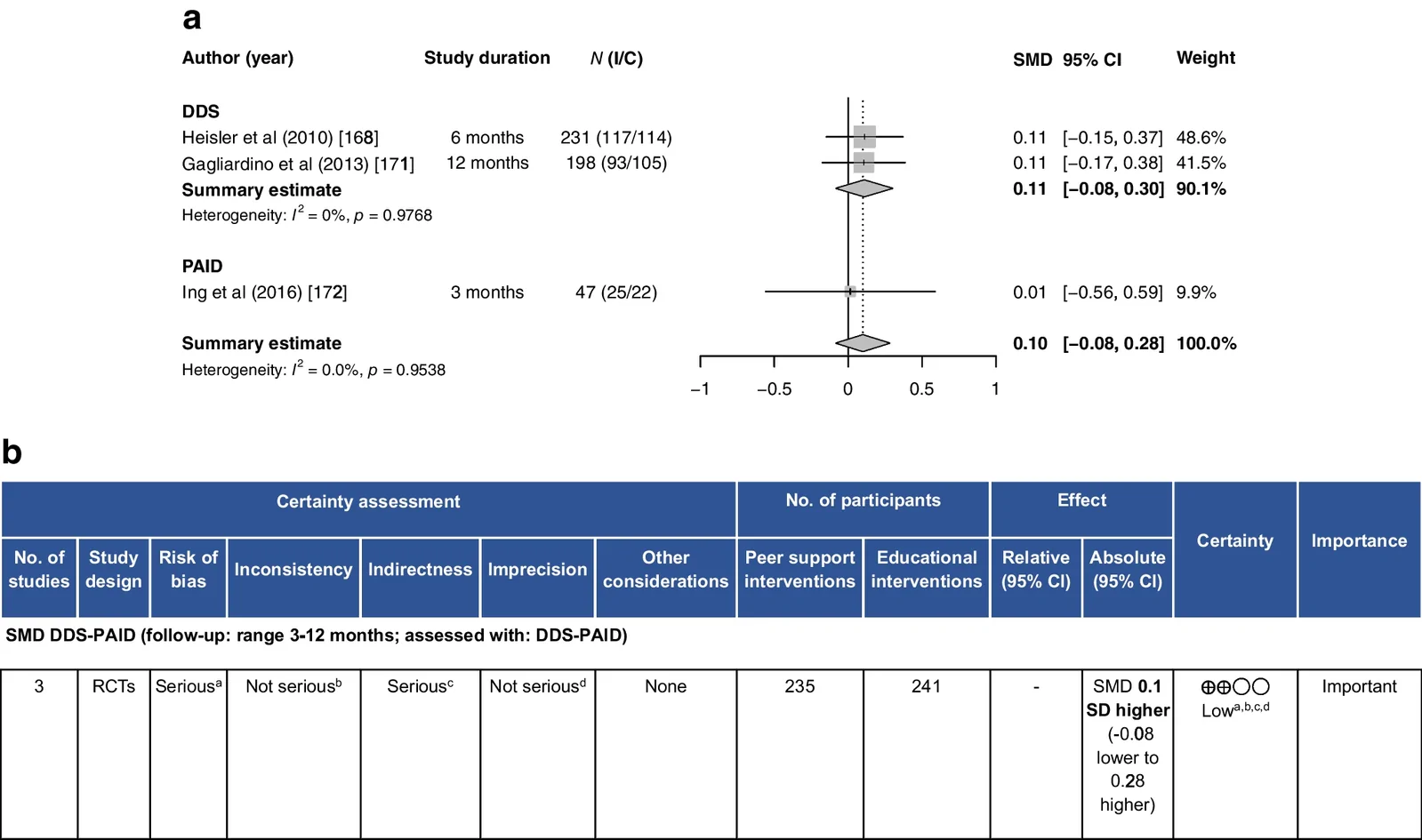
(**a**) SMD for peer support compared with educational interventions in type 2 diabetes. C, control; I, intervention. (**b**) GRADE evidence profile for peer support compared with educational interventions in type 2 diabetes. ^a^One trial had a high risk of bias and two had some concerns. ^b^*I*^2^=0.0%. ^c^Different types of interventions. ^d^95% CI crosses null but not –0.3 or 0.3

Six trials compared peer support interventions (without a psychological component) with educational interventions (without a psychological component) in adults with type 2 diabetes [168, 170–174]. Meta-analysis of three of these trials showed a non-significant effect (0.10 [95% CI −0.08, 0.28]) in favour of educational interventions for the reduction of diabetes distress (Fig. 13a) [168, 171, 172]. The respective GRADE evidence profile is shown in Fig. 13b. Subgroup analyses revealed no significant subgroup effects by baseline diabetes distress, trial duration or risk of bias score (*p*>0.05) (ESM 5, Fig. 13c-e). Narrative analysis of the trials that could not be included in the meta-analysis showed similar effects on the reduction of diabetes distress [170, 173, 174].

##### Peer support interventions compared with usual care for type 2 diabetes

**Fig. 14:**
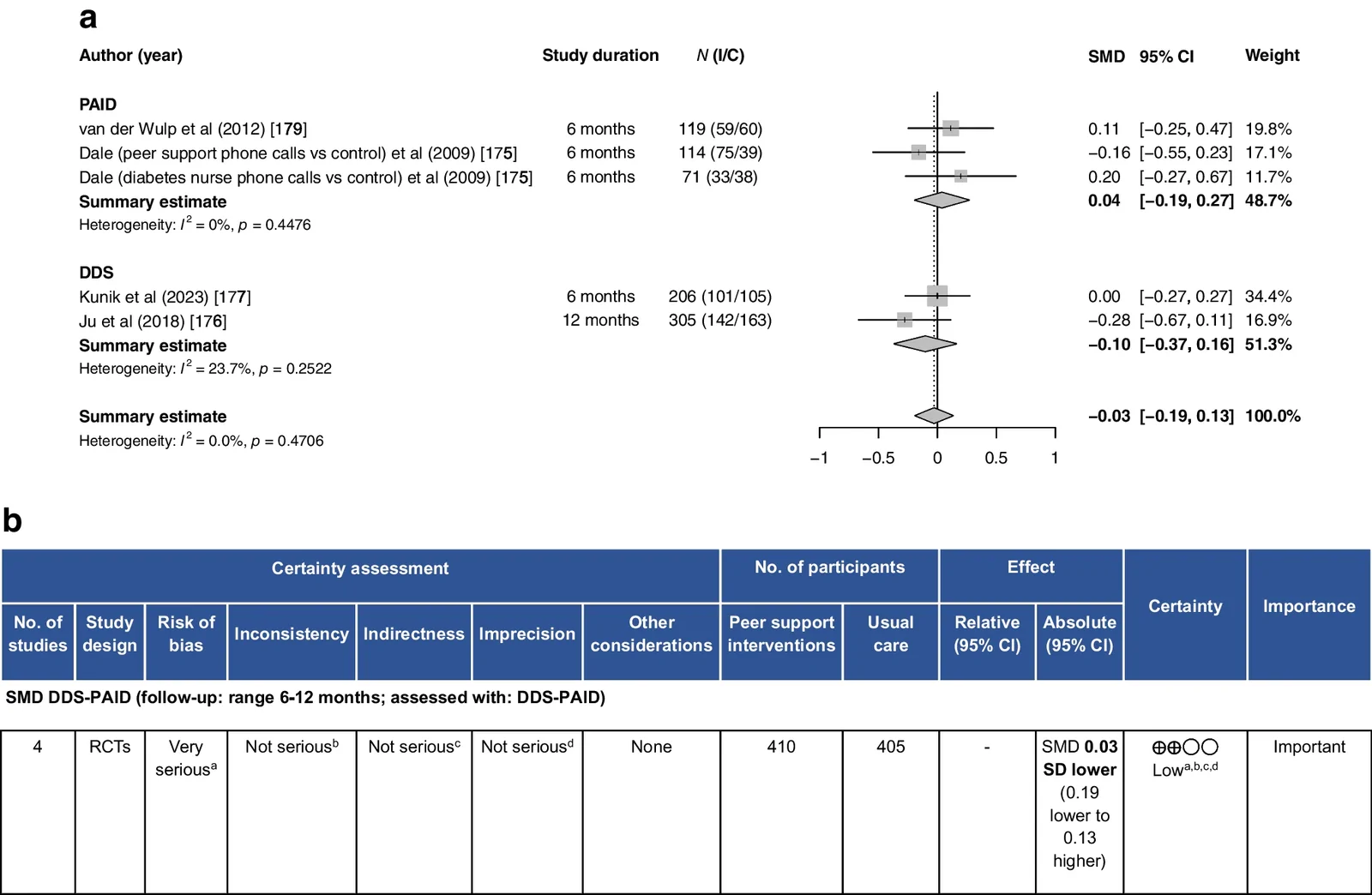
(**a**) SMD for peer support compared with usual care in type 2 diabetes. C, control; I, intervention. (**b**) GRADE evidence profile for peer support compared with usual care in type 2 diabetes. ^a^Most trials at high risk of bias. ^b^*I*^2^=0.0%. ^c^Variations in the types of interventions not considered important enough to downgrade. ^d^95% CI crosses null but not –0.3 or 0.3

Seven trials compared peer support interventions (without a psychological component) with usual care in adults with type 2 diabetes [169, 174–179]. Meta-analysis of four of these trials, including five comparisons, showed a non-significant effect of peer support (−0.03 [95% CI −0.19, 0.13]) on diabetes distress in type 2 diabetes (Fig. 14a) [175–177, 179]. The respective GRADE evidence profile is shown in Fig. 14b. Subgroup analyses revealed no significant subgroup effects by baseline diabetes distress, trial duration, intervention delivery format or risk of bias score (*p*>0.05) (ESM 5, Fig. 14c–f). Narrative analysis of the trials that could not be included in the meta-analysis showed similar effects as the meta-analysis in reducing diabetes distress [169, 174, 178].

##### Educational interventions compared with other interventions for type 2 diabetes

Four trials compared educational interventions (without a psychological component) with other miscellaneous interventions in adults with type 2 diabetes [180–183]. Meta-analysis of these trials was not possible. Narrative analysis showed a small positive effect in favour of educational interventions (without a psychological component) on reducing diabetes distress [180–183].

##### Educational interventions compared with usual care for type 2 diabetes

**Fig. 15:**
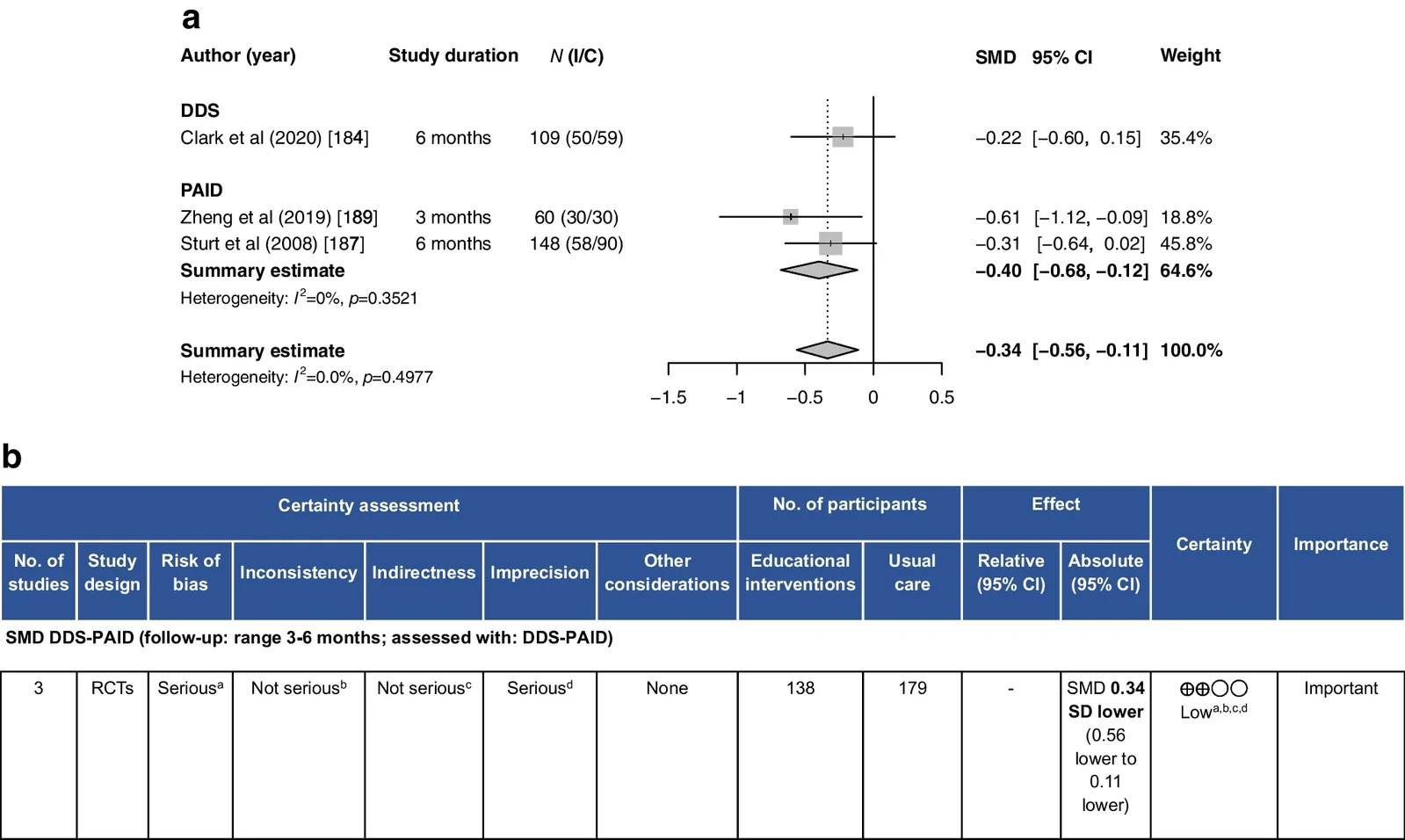
(**a**) SMD for educational interventions compared with usual care in type 2 diabetes. C, control; I, intervention. (**b**) GRADE evidence profile for educational interventions compared with usual care in type 2 diabetes. ^a^One trial at high risk of bias and two with some concerns. ^b^*I*^2^=0.0%. ^c^Variations in the types of interventions not considered important enough to downgrade. ^d^95% CI crosses –0.3 but not null

Six trials compared educational interventions (without a psychological component) with usual care in adults with type 2 diabetes [184–189]. Meta-analysis of three of these trials showed a significant effect of educational interventions (−0.34 [95% CI −0.56, −0.11]) on diabetes distress in type 2 diabetes (Fig. 15a) [184, 187, 189]. The respective GRADE evidence profile is shown in Fig. 15b. Subgroup analyses revealed no significant subgroup effects of risk of bias score or delivery format (*p*>0.05) (ESM 5, Fig. 15c, d). Narrative analysis of the trials that could not be included in the meta-analysis showed similar effects of educational interventions to those seen in the meta-analysis with regard to reduction of diabetes distress [185, 186, 188].

#### Publication bias

Funnel plots are presented in ESM 6. Egger’s tests indicated significant funnel plot asymmetry for psychological interventions compared with usual care for type 2 diabetes (*p*=0.0209) and peer support compared with educational interventions for type 2 diabetes (*p*=0.0094). However, only the former included more than ten studies, allowing a more reliable assessment of the observed asymmetry. As the latter was based on only three studies, this result should be interpreted with caution.

## Discussion

In this systematic review, we systematically assessed all available evidence from RCTs assessing the efficacy of different types of interventions for reducing diabetes distress among adults with type 1 or type 2 diabetes. This resulted in the inclusion of nearly 250 trials reporting on psychological interventions, psychoeducational interventions and miscellaneous interventions in adults with type 1 diabetes and type 2 diabetes. Compared with usual care, psychological interventions reduced diabetes distress in adults with type 1 diabetes and those with type 2 diabetes. Additionally, continuous glucose monitoring or intermittently scanned glucose monitoring reduced diabetes distress in adults with type 1 diabetes, compared with capillary glucose monitoring, while educational interventions reduced diabetes distress in adults with type 2 diabetes, compared with usual care. Other comparisons, including those of psychological, psychoeducational, peer support, educational and device-based interventions against various active controls or usual care, did not show statistically significant effects. For some comparisons, lack of statistical significance reflected estimates that were marginally non-significant rather than clearly null effects. In particular, psychoeducational interventions, compared with usual care in adults with type 2 diabetes, and AID compared with no AID in adults with type 1 diabetes, showed point estimates favouring the intervention, with confidence intervals narrowly crossing the null. As such, clinical recommendations should not be based solely on statistical significance of pooled estimates, but should consider the full GRADE evidence to decision framework [190]. The results did not differ meaningfully across predefined subgroups, indicating that the observed intervention effects were broadly consistent across these characteristics. All trials were judged to have either some concerns for risk of bias or a high risk of bias, and the certainty of evidence, as assessed by GRADE methodology, was rated as moderate, low or very low. No meta-analyses could be conducted for telehealth, health behaviour and the assorted mixed interventions because of heterogeneity or an insufficient number of trials.

Our results are in line with previous systematic reviews. For instance, a large Cochrane review specifically focused on psychological interventions for diabetes distress in adults with type 2 diabetes found a small but significant effect on diabetes distress in favour of psychological interventions compared with usual care [11]. In addition, the systematic reviews by Zu et al and Schmidt et al found positive effects of psychological interventions in adults with type 2 diabetes with elevated diabetes distress, and the systematic review by Mathiesen et al found positive effects of cognition-focused and emotion-focused interventions in vulnerable adults with type 2 diabetes [12, 15, 17]. Furthermore, several systematic reviews found motivational interviewing to have a beneficial effect on diabetes distress [12, 13, 16]. In contrast to the previous systematic reviews by Sturt et al and Perrin et al, which found a small reduction in diabetes distress with psychoeducational interventions, we did not find a significant effect of psychoeducational interventions on diabetes distress [14, 16]. Despite some overlap in included studies, our search was broader and more recent, with the additional trials causing heterogeneity and possibly diluting any potential effects. Intervention heterogeneity also made it difficult to determine which specific elements contribute to intervention effectiveness between categories. Even within the same category, for example psychological interventions, there was considerable diversity; interventions varied in focus (e.g. targeting diabetes distress vs depression, anxiety or eating behaviours), duration (ranging from brief sessions to multi-year programmes), delivery format (e.g. in person vs telehealth, individual vs group based) and provider type (e.g. physician, psychologist or multidisciplinary teams). For practical guidance on assessment and management strategies for diabetes distress, see the EASD clinical guideline on diabetes distress, which synthesises current evidence and expert consensus into clinically applicable recommendations that go beyond what can be inferred from the included studies [191].

We observed no meaningful differences across predefined subgroups. Subgroup analyses showed no significant subgroup effects in all predefined comparisons (i.e. *p*>0.05 for subgroup interaction). Several previous systematic reviews have reported similar findings. For example, Sturt et al did not find large differences between intervention delivery formats or individual vs group interventions, while Yakubu et al found only small differences related to baseline diabetes distress or trial duration [16, 18]. By contrast, Zu et al suggested that interventions delivered in groups using a technology component enhanced diabetes distress reduction [17], while Mathiesen et al reported the largest reductions in diabetes distress after individual interventions [12]. These differences may be caused by differences in (sub)categorisation, which hinders comparability. In addition, the broad scope of our systematic review resulted in the inclusion of many trials with low baseline levels of diabetes distress, which made it difficult to detect a meaningful reduction in diabetes distress.

Finally, all trials included in our systematic review were judged to be at risk of bias, and the certainty of the evidence was moderate, low or very low. The risk of bias was downgraded for all trials because of the use of PROMs as outcome measures, as this always led to ‘some concern for risk of bias’ based on the standard applications of the RoB2 assessment. Additionally, we included trials using the DDS or PAID, which are two distinct PROMs that have not been calibrated against each other, making direct comparison of their scores problematic. For this reason, we used SMDs to allow for pooled analysis across trials using different instruments and performed subgroup analyses based on PROMs. Additionally, the two PROMs may differ in specificity and psychometric behaviour; for example, they may plateau at different levels, or capture overlapping but not identical aspects of the construct of diabetes distress. Therefore, future trials should include either both PROMS or calibration should be performed. For example, within the psychological intervention category, only one trial compared a psychological intervention with a psychoeducational intervention in people with type 1 diabetes. As a result, more research is needed before firm conclusions can be made in these areas.

Our systematic review and meta-analysis provide an extensive overview on the topic of diabetes distress. We carried out a high-quality review by using validated tools such as GRADE for certainty assessment, along with categorisation of interventions, which was reviewed and verified by clinical experts. This review is the most comprehensive synthesis to date, evaluating a wide range of interventions across type 1 and type 2 diabetes. In contrast, earlier systematic reviews were narrower in scope, often being restricted to a single population or specific intervention categories. Nonetheless, our study has certain limitations.

First, we categorised trials to the best of our ability; however, in some cases, insufficient detail was provided about the underlying psychological theory or intervention content. Because of the heterogeneous nature of the interventions, some trials could reasonably have been assigned to more than one category. When mixed populations were included or when the ratio of participants with type 1 and type 2 diabetes was approximately 50/50 or undefined, we included these trials in both the type 1 diabetes and the type 2 diabetes analyses, which resulted in overlap of trials across analyses for type 1 diabetes and type 2 diabetes. However only five such trials were included in the meta-analysis, and these trials were part of outcome categories that also included a substantial number of other studies. Consequently, the relative statistical weight of the duplicate trials within each meta-analysis was limited, thereby reducing their influence on the pooled effect estimates. We therefore consider the potential bias introduced by this approach to be limited. To facilitate meta-analysis, we deliberately limited subcategorisation and combined trials wherever methodologically reasonable.

At the same time, even when interventions were classified within the same broad categories (e.g. psychological or educational), they inevitably varied in content, intensity, delivery format and contextual factors such as participant engagement, cultural appropriateness and care setting. These intervention-level and contextual characteristics were not examined systematically in the present review, and it therefore remains unclear to what extent they may have contributed to the observed effects. This heterogeneity underscores the need for future research to more precisely identify which specific intervention components, delivery characteristics and contextual factors are most likely to result in meaningful and sustained reductions in diabetes distress.

Second, in contrast to the protocol (see ESM 7 and 8), we could only use post-intervention (endpoint) values in our meta-analyses, as many trials did not report changes from baseline for diabetes distress. Additionally, although several secondary outcomes (e.g. HbA_1c_, depression, quality of life, anxiety and treatment satisfaction) were initially considered at the protocol stage, a decision was made to focus the evidence synthesis exclusively on diabetes distress. This decision was informed by both practical considerations related to the very large number of eligible trials and available resources, and methodological considerations regarding alignment with the scope and objective of the meta-analysis. In particular, the present systematic review was explicitly designed to evaluate the effects of interventions on diabetes distress, and the search strategy and eligibility criteria were therefore centred on diabetes distress-related terms. As a result, trials evaluating similar interventions with respect to other outcomes, but not assessing diabetes distress, were not systematically sought and were not eligible for inclusion. Consequently, any quantitative synthesis of secondary outcomes (such as HbA_1c_) based solely on the included studies would represent an incomplete and potentially misleading assessment of intervention effects for those outcomes, as it would exclude a substantial body of relevant evidence by design. Therefore, no additional outcomes were assessed other than diabetes distress.

Third, despite considerable efforts by the research team, including contacting trial authors at least twice, only a subset responded. As a result, a substantial proportion of data remained missing. Many trials did not report absolute numbers, or reported only means or medians without accompanying measures of variability. This issue was particularly pronounced in the ‘miscellaneous interventions’ category, where diabetes distress was often not the primary outcome. For these trials, diabetes distress was often reported only in supplementary materials and not in the abstract or the main text of the articles. As such, although we also conducted manual reference screening, we cannot rule out the possibility that additional relevant trials may have been missed, particularly for interventions that did not primarily target diabetes distress (e.g. devices). This challenge is illustrated in the systematic review by Olsen et al [192] on diabetes technology and PROMs, which helped us to identify trials of device-based interventions that reported on diabetes distress. Notably, we also identified trials that were not captured in this review, for example the study by Boscari et al [148], highlighting the challenge of identifying relevant trials when key outcomes such as diabetes distress are not explicitly mentioned in the main text, even when carrying out reference list checking and using appropriate search strategies.

Fourth, we included all types of RCT designs, including crossover and cluster trials. Each design has its own strengths and limitations, which may have influenced the results. However, the vast majority of the included RCTs used a parallel design, which likely minimised the overall impact of design heterogeneity on the findings. Moreover, cluster RCTs reporting an ICC were adjusted for clustering using the design effect [21, 130, 137, 176]. For cluster RCTs without a reported ICC, we found that the pooled effect estimates were comparable to the primary analysis estimates [99, 108, 167]. No adjustment was applied to crossover trials because only endpoint data combined across periods were reported, precluding a paired analysis. As only a small number of crossover trials were included, their potential impact on the overall results is likely to be limited [144, 154, 193]. In addition, a considerable number of included studies were pilot or feasibility studies, primarily designed to test the implementation or feasibility of interventions rather than to evaluate effectiveness. These studies generally had small sample sizes. However, as meta-analytical methods can account for small studies through appropriate weighting, we do not expect this to have significantly impacted the results.

Fifth, as outlined in the protocol (see ESM 7), we predefined the categorisation of intervention types. Nevertheless, alternative classification schemes are possible, and applying different criteria may have resulted in different groupings. We believe that the categories we used best reflect the current landscape and types of interventions in the field and that re-categorisation would have a limited effect on the results.

Sixth, we included the term ‘distress’ in the literature search strategy. However, this term was not widely adopted to describe the constructs measured by instruments such as the PAID until the publication of the DDS17 in around 2007. As a result, relying on ‘distress’ as a search term may have led to the omission of some earlier trials. However, as the search strategy also included a range of related terms (e.g. ‘psychological stress’, ‘burnout’) and specific scale names and acronyms (e.g. PAID, PAID-20, T1-DDS, T2-DDS), we believe that the risk of missing relevant trials was attenuated. Moreover, before 2007 there were no validated PROMs, so these trials could not have been included in this systematic review. This approach is consistent with previous high-quality systematic reviews on diabetes distress, including a Cochrane review [11].

Finally, although sex was reported in the different trials, methods for its ascertainment were not described and many did not perform subgroup analyses by sex, Therefore, we could not assess whether the effects of the assessed interventions may differ between women and men.

Overall, this systematic review provides an up-to-date evidence-based synthesis that reflects current practices and innovations in the management of diabetes distress. It served as the basis for the EASD evidence-based clinical guideline on diabetes distress among adults, enabling healthcare professionals to optimise their support of people living with diabetes [191]. Given the high prevalence of diabetes distress and its negative emotional impact, in congruence with ADA and NICE guidelines [7–9], we believe it is important to implement screening for diabetes distress in people with diabetes. Our findings support the use of psychological interventions, such as cognitive behavioural therapy or mindfulness, as first-line treatment for adults with type 1 diabetes and those with type 2 diabetes reporting diabetes distress. Clinicians are encouraged to integrate these interventions in their routine diabetes care or through referral to mental health professionals. Moreover, CGM should be made easily available for people with type 1 diabetes, as our findings suggest a beneficial effect on diabetes distress reduction. Among adults with type 2 diabetes, diabetes education programmes were shown to reduce diabetes distress. These programmes are likely to benefit people with diabetes by improving self-efficacy and disease understanding. Therefore, clinicians are encouraged to provide diabetes education tailored to the needs of patients. Other intervention types showed inconsistent or limited effects on diabetes distress. Therefore, firm clinical recommendations cannot be made for these categories at present, indicating that more research is needed. Finally, given the high heterogeneity across trials, our results should be interpreted with caution. We recommend that clinicians consider the evidence in this systematic review but remain aware of the specific needs of people with diabetes and refer them to mental health professionals or tailor interventions accordingly.

To conclude, this systematic review and meta-analysis found that psychological interventions consistently reduced diabetes distress among adults with type 1 diabetes and those with type 2 diabetes, while device-related interventions reduced diabetes distress in adults with type 1 diabetes and educational interventions reduced diabetes distress in adults with type 2 diabetes. Most other interventions showed little or no clear effects, and several intervention categories could not be analysed because of heterogeneity or limited data.

## Supplementary Information

Below is the link to the electronic supplementary material.

ESM (PDF 4.28 MB)

## Data Availability

The extracted data and other materials underlying the results reported in this article are available from the EASD (info@easd.org) upon reasonable request to researchers who provide a methodologically sound proposal and for the duration of the approved project. The shared materials include the published aggregate study-level data extracted for the meta-analyses; no individual participant-level data are available. Shared materials may not be used for purposes that are inconsistent with the approved proposal or otherwise misrepresent the findings of the review.

## Abbreviations

AID: Automated insulin delivery
CGM: Continuous glucose monitor
DDS: Diabetes Distress Scale
GRADE: Grading of Recommendations Assessment, Development and Evaluation
ICC: Intracluster correlation coefficient
MCID: Minimal clinically important difference
NICE: National Institute for Health and Care Excellence
PAID: Problem Areas In Diabetes
PRISMA: Preferred Reporting Items for Systematic Reviews and Meta-Analyses
PROMs: Patient-reported outcome measures
SMD: Standardised mean difference
