# Supplementary material for: Interventions to reduce diabetes-related distress among adults with type 1 or type 2 diabetes: a systematic review and meta-analysis of randomised controlled trials
Source: Diabetologia. 2026 Sep 11;69(12):3471–500. doi: 10.1007/s00125-026-06789-0 (PMC13714115; doi:10.1007/s00125-026-06789-0)
Supplement: Supplementary file 1 [file 125_2026_6789_MOESM1_ESM.pdf]

# Electronic Supplementary Material

# Contents

1. Search strategy ........................................ 3
2. Reasons for exclusion of full text papers ........................................ 8
3. Baseline table ........................................ 15
4. Risk of bias assessment ........................................ 83
5. Sensitivity and subgroup analyses ........................................ 89
6. Funnel plots ........................................ 111
7. Study protocol ........................................ 117
8. Differences between protocol and review ........................................ 132

## 1. Search strategy

Diabetes Distress, search Histories and details conducted on September 23, 2024

Results

| Database | Results | Records removed |
|---|---|---|
| PubMed | 3384 | |
| PsycInfo (Ebsco) | 794 | |
| Cinahl (Ebsco) | 1491 | |
| The Cochrane Library (Wiley) | 2232 | |
| Total | 7901 | |
| After deduplication DedupEndNote | 6015 | 1886 |
| After deduplication manually in Endnote | 5893 | 122 |

PubMed History and Search Details September 23, 2024

| Search | Query PubMed | Results |
|---|---|---|
| #1 | "Diabetes Mellitus"[Mesh:NoExp] OR "Diabetes Mellitus, Type 1" OR "Diabetes Mellitus, Type 2"[Mesh] OR diabet*[tiab] OR dm2[tiab] OR niddm[tiab] OR "dm 2"[tiab] OR t2d[tiab] OR "dm type 2"[tiab] OR "dm type II"[tiab] OR dm1[tiab] OR iddm[tiab] OR "dm 1"[tiab] OR t1d[tiab] OR "dm type 1"[tiab] OR "dm type I"[tiab] | 883,347 |
| #2 | ("Psychological Distress"[Mesh] OR "Stress, Psychological"[Mesh] OR distress*[tiab] OR "problem area*"[tiab] OR "problems area*"[tiab] OR "burnout"[tiab] OR "burn-out"[tiab] OR burned-out[tiab] OR "DDS-2"[tiab] OR "DDS-17"[tiab] OR "T1-DDS"[tiab] OR "T2-DDS"[tiab] OR "PAID"[tiab] OR "PAID-1"[tiab] OR "PAID-5"[tiab] OR "PAID-11"[tiab] OR "PAID-20"[tiab] OR "CDSS"[tiab] OR "QSD"[tiab] OR "Questionnaire on Stress in Diabet*") NOT | 352,234 |

| Search | Query PubMed | Results |
|---|---|---|
| | ("oxidative distress"[tiab] OR "oxidative stress"[tiab] OR "respiratory distress"[tiab] OR "respiratory stress"[tiab]) | |
| #3 | ("randomized controlled trial"[pt] OR "controlled clinical trial"[pt] OR "Clinical Trial" [pt] OR "clinical trials as topic"[mesh:noexp] OR "Controlled Clinical Trials as Topic"[Mesh] OR "Meta-Analysis" [pt] OR "Meta-Analysis as Topic"[Mesh] OR "Review"[pt] OR "Review Literature as Topic"[Mesh] OR trial[tiab] OR rct[tiab] OR random*[tiab] OR placebo[tiab] OR meta-analys*[tiab] OR metaanalys*[tiab] OR review[tiab] OR "literature study"[tiab]) NOT (animals[mh] NOT humans [mh]) | 6,058,038 |
| #4 | #1 AND #2 AND #3 | 3,384 |

APA PsycInfo History and Search Details September 23, 2024

| Search | APA PsycInfo (Ebsco) Query | Results |
|---|---|---|
| S1 | DE ("Diabetes" OR "Diabetes Mellitus" OR "Type 1 Diabetes" OR "Type 2 Diabetes") OR TI (diabet* OR dm2 OR niddm OR "dm 2" OR t2d OR "dm type 2" OR "dm type II" OR dm1 OR iddm OR "dm 1" OR t1d OR "dm type 1" OR "dm type I") OR AB (diabet* OR dm2 OR niddm OR "dm 2" OR t2d OR "dm type 2" OR "dm type II" OR dm1 OR iddm OR "dm 1" OR t1d OR "dm type 1" OR "dm type I") OR KW (diabet* OR dm2 OR niddm OR "dm 2" OR t2d OR "dm type 2" OR "dm type II" OR dm1 OR iddm OR "dm 1" OR t1d OR "dm type 1" OR "dm type I") | 39,526 |
| S2 | (DE ("Distress" OR "Burnout") OR TI (distress* OR "problem* area*" OR "burnout" OR "burn-out" OR "burned out" OR "DDS-2" OR "DDS-17" OR "T1-DDS" OR "T2-DDS" OR "PAID" OR "PAID-1" OR "PAID-5" OR "PAID-11" OR "PAID-20" OR "CDSS" OR "QSD" OR "Questionnaire on Stress in Diabet*") OR AB ((distress* OR "problem* area*" OR "burnout" OR "burn-out" OR "burned out" OR "DDS-2" OR "DDS-17" OR "T1-DDS" OR "T2-DDS" OR "PAID" OR "PAID-1" OR "PAID-5" OR "PAID-11" OR "PAID-20" OR "CDSS" OR | 149,584 |

| Search | APA PsycInfo (Ebsco) Query | Results |
|---|---|---|
| | "QSD" OR "Questionnaire on Stress in Diabet*") OR KW (distress* OR "problem* area*" OR "burnout" OR "burn-out" OR "burned out" OR "DDS-2" OR "DDS-17" OR "T1-DDS" OR "T2-DDS" OR "PAID" OR "PAID-1" OR "PAID-5" OR "PAID-11" OR "PAID-20" OR "CDSS" OR "QSD" OR "Questionnaire on Stress in Diabet*")) NOT (TI ((oxidative OR respiratory) N1 (distress OR stress)) OR AB ((oxidative OR respiratory) N1 (distress OR stress)) OR KW ((oxidative OR respiratory) N1 (distress OR stress))) | |
| S3 | S2 NOT (TI ((oxidative OR respiratory) N1 (distress OR stress)) OR AB ((oxidative OR respiratory) N1 (distress OR stress)) OR KW ((oxidative OR respiratory) N1 (distress OR stress))) | 148,829 |
| S4 | DE ("Meta Analysis") OR TI (trial OR rct OR control* OR random* OR review OR meta-analys* OR metaanalys*) OR AB (trial OR rct OR control* OR random* OR meta-analys* OR metaanalys* OR review) OR KW (trial OR rct OR control* OR random* OR meta-analys* OR metaanalys* OR review) OR ZC ("clinical trial" OR "meta analysis" OR "metasynthesis" OR "literature review") | 1,593,556 |
| S5 | S1 AND S3 AND S4 | 794 |

CINAHL PLUS History and Search Details September 23, 2024

| Search | CINAHL Plus (Ebsco) Query | Results |
|---|---|---|
| S1 | MH ("Diabetes Mellitus" OR "Diabetes Mellitus, Type 1" OR "Diabetes Mellitus, Type 2") OR TI (diabet* OR dm2 OR niddm OR "dm 2" OR t2d OR "dm type 2" OR "dm type II" OR dm1 OR iddm OR "dm 1" OR t1d OR "dm type 1" OR "dm type I") OR AB (diabet* OR dm2 OR niddm OR "dm 2" OR t2d OR "dm type 2" OR "dm type II" OR dm1 OR iddm OR "dm 1" OR t1d OR "dm type 1" OR "dm type I") | 281,764 |
| S2 | (MH ("Psychological Distress" OR "Symptom Distress") OR TI (distress* OR "problem* area*" OR "burnout" OR "burn-out" OR | 97,969 |

| Search | CINAHL Plus (Ebsco) Query | Results |
|---|---|---|
| | "burned out" OR "DDS-2" OR "DDS-17" OR "T1-DDS" OR "T2-DDS" OR "PAID" OR "PAID-1" OR "PAID-5" OR "PAID-11" OR "PAID-20" OR "CDSS" OR "QSD" OR "Questionnaire on Stress in Diabet*") OR AB (distress* OR "problem* area*" OR "burnout" OR "burn-out" OR "burned out" OR "DDS-2" OR "DDS-17" OR "T1-DDS" OR "T2-DDS" OR "PAID" OR "PAID-1" OR "PAID-5" OR "PAID-11" OR "PAID-20" OR "CDSS" OR "QSD" OR "Questionnaire on Stress in Diabet*")) NOT (TI ((oxidative OR respiratory) N1 (distress OR stress)) OR AB ((oxidative OR respiratory) N1 (distress OR stress))) | |
| S3 | MH ("Clinical Trials+" OR "Randomized Controlled Trials" OR "Therapeutic Trials" OR "Meta Analysis" OR "Meta Synthesis" OR "Literature Review+") OR TI (trial OR rct OR control* OR random* OR meta-analys* OR metaanalys* OR review OR "literature study") OR AB (trial OR rct OR control* OR random* OR meta-analys* OR metaanalys* OR review OR "literature study") | 1,838,828 |
| S4 | S1 AND S2 AND S3 | 1,491 |

The Cochrane Library (Wiley) History and Search Details September 23, 2024

| Search | The Cochrane Library (Wiley) Query | Results |
|---|---|---|
| #1 | ((diabet* OR dm2 OR niddm OR "dm 2" OR t2d OR "dm type 2" OR "dm type II" OR dm1 OR iddm OR "dm 1" OR t1d OR "dm type 1" OR "dm type I")) :ti,ab,kw (Word variations have been searched) | 125,957 |
| #2 | ((distress* OR problem* NEXT area* OR "burnout" OR "burn-out" OR "burned out" OR "DDS-2" OR "DDS-17" OR "T1-DDS" OR "T2-DDS" OR "PAID" OR "PAID-1" OR "PAID-5" OR "PAID-11" OR "PAID-20" OR "CDSS" OR "QSD" OR "Questionnaire on Stress" NEAR/2 Diabet*)) :ti,ab,kw NOT (((oxidative OR respiratory) NEXT (distress OR stress))):ti (Word variations have been searched) | 38,424 |

| Search | The Cochrane Library (Wiley) Query | Results |
|---|---|---|
| #3 | (oxidative OR respiratory) NEXT (distress OR stress):ti<br>(Word variations have been searched) | 6,316 |
| #4 | #1 AND (#2 NOT #3) | 2,234 |
| | Results:<br>20 Cochrane Reviews<br>2212 Trials | |

## 2. Reasons for exclusion of full text papers

| Study | Exclusion reason |
|---|---|
| Afshar 2022 | Wrong Study Design |
| Aikens 1997 | Wrong outcome |
| Alessi 2022 | Duplicate |
| Allen 2017 | Wrong outcome |
| Alloway 2001 | Wrong outcome |
| An 2023 | Review |
| Anderbro 2022 | Only abstract/thesis/design |
| Arora 2013 | Only abstract/thesis/design |
| Ayala 2022 | Wrong study design |
| Azadbakht 2023 | Wrong study design |
| Baca 2007 | Only abstract/thesis/design |
| Baig 2020 | Only abstract/thesis/design |
| Baig 2021 | Only abstract/thesis/design |
| Baig 2021 | Only abstract/thesis/design |
| Bajaj 2016 | Only abstract/thesis/design |
| Banks 2023 | Wrong study design |
| Barcena 2021 | Only abstract/thesis/design |
| Barrett 1995 | Wrong outcome |
| Bassi 2021 | Review |
| Bastian 2020 | Only abstract/thesis/design |
| Benhalima 2021 | Only abstract/thesis/design |
| Benhalima 2025 | Duplicate |
| Bergis 2019 | Wrong population |
| Bilgin 2022 | Review |
| Bisno 2019 | Only abstract/thesis/design |
| Bisno 2023 | Wrong population |
| Bogusch 2019 | Review |
| Bowyer 2014 | Only abstract/thesis/design |
| Browning 2016 | Wrong outcome |
| Burner 2015 | Only abstract/thesis/design |
| Bykowski 2017 | Only abstract/thesis/design |
| Carreira 2014 | Only abstract/thesis/design |
| Castensøe-Seidenfaden 2018 | Wrong population |
| Celik 2020 | Review |
| Cheng 2017 | Review |
| Cheng 2017 | Only abstract/thesis/design |
| Chew 2017 | Review |
| Chew 2017 | Only abstract/thesis/design |

| | |
|---|---|
| Chiu 2014 | Only abstract/thesis/design |
| Cho 2021 | Review |
| Cohen 2023 | Only abstract/thesis/design |
| Cowart 2020 | Review |
| Cox 2020 | Only abstract/thesis/design |
| Cummings 2015 | Only abstract/thesis/design |
| Cummings 2017 | Wrong Intervention |
| Cummings 2017 | Only abstract/thesis/design |
| Cummings 2018 | Only abstract/thesis/design |
| Cummings 2019 | Only abstract/thesis/design |
| Curtis 2022 | Only abstract/thesis/design |
| Dafoulas 2014 | Only abstract/thesis/design |
| Dafoulas 2014 | Only abstract/thesis/design |
| DeGroot 2017 | Only abstract/thesis/design |
| DeGroot 2018 | Only abstract/thesis/design |
| Delgado 2018 | Only abstract/thesis/design |
| Dennick 2013 | Only abstract/thesis/design |
| DeVries 2014 | Only abstract/thesis/design |
| Diribe 2024 | Review |
| Efthymiadis 2022 | Review |
| Ehrmann 2016 | Wrong study design |
| Ehrmann 2018 | Only abstract/thesis/design |
| Ehrmann 2018 | Only abstract/thesis/design |
| Ehrmann 2023 | Only abstract/thesis/design |
| Ehrmann 2023 | Only abstract/thesis/design |
| Ehrmann 2024 | Wrong population |
| Ewen 2023 | Only abstract/thesis/design |
| Farzaneh 2023 | Wrong study design |
| Fernandez-Rodrigo 2022 | Only abstract/thesis/design |
| Fernández-Rodríguez 2024 | Review |
| Fisher 2012 | Only abstract/thesis/design |
| Fisher 2015 | Only abstract/thesis/design |
| Fisher 2018 | Only abstract/thesis/design |
| Fisher 2019 | Duplicate |
| Fisher 2020 | Wrong outcome |
| Fortmann 2016 | Only abstract/thesis/design |
| Franceschi 2023 | Review |
| Freckmann 2018 | Only abstract/thesis/design |
| Glasgow 2009 | Wrong study design |
| Godoi 2023 | Review |

| | |
|---|---|
| GonçalvesdeBrito 2016 | Wrong study design |
| Gonzalez 2024 | Wrong study design |
| Gray 2021 | Wrong outcome |
| Groeneveld 2024 | Wrong outcome |
| Gulde 2009 | Review |
| Guo 2019 | Review |
| Gutierrez 2019 | Review |
| Hadjiconstantinou 2016 | Review |
| Hall 2024 | Wrong outcome |
| Halliday 2022 | Wrong population |
| Hambidge 2016 | Only abstract/thesis/design |
| Hambidge 2017 | Only abstract/thesis/design |
| Hamdy 2015 | Only abstract/thesis/design |
| Hartmann 2012 | Wrong outcome |
| Hawkins 2022 | Only abstract/thesis/design |
| Heitkemper 2017 | Only abstract/thesis/design |
| Herder 2018 | Wrong Intervention |
| Hermanns 2011 | Only abstract/thesis/design |
| Hermanns 2011 | Only abstract/thesis/design |
| Hermanns 2014 | Only abstract/thesis/design |
| Hermanns 2016 | Only abstract/thesis/design |
| Hermanns 2016 | Only abstract/thesis/design |
| Hermanns 2019 | Wrong population |
| Hermanns 2019 | Only abstract/thesis/design |
| Hessler 2020 | Only abstract/thesis/design |
| Hessler 2023 | Only abstract/thesis/design |
| Hilmarsdottircc 2020 | Only abstract/thesis/design |
| Hinsch 2015 | Only abstract/thesis/design |
| Hood 2021 | Wrong population |
| Hopkins 2012 | Wrong study design |
| Hur 2020 | Only abstract/thesis/design |
| Ismail 2004 | Review |
| Iversen 2019 | Only abstract/thesis/design |
| Jenkinson 2022 | Review |
| Johnson 2013 | Only abstract/thesis/design |
| jRct 2023 | Only abstract/thesis/design |
| Jugnee 2017 | Only abstract/thesis/design |
| Jugnee 2018 | Only abstract/thesis/design |
| Karahan 2009 | Wrong outcome |
| Kaselitz 2019 | Wrong outcome |

| | |
|---|---|
| Kasteleyn 2014 | Only abstract/thesis/design |
| Katz 2017 | Only abstract/thesis/design |
| Kelly 2023 | Only abstract/thesis/design |
| Kempf 2013 | Only abstract/thesis/design |
| Khan 2022 | Only abstract/thesis/design |
| Khunti 2010 | Only abstract/thesis/design |
| Kong 2020 | Review |
| Kopf 2014 | Wrong outcome |
| Koscielniak 2018 | Only abstract/thesis/design |
| Kotsanos 1997 | Wrong outcome |
| Kudva 2021 | Wrong population |
| Kulzer 2012 | Only abstract/thesis/design |
| Kulzer 2014 | Only abstract/thesis/design |
| Kulzer 2014 | Only abstract/thesis/design |
| Kulzer 2019 | Only abstract/thesis/design |
| Kulzer 2023 | Only abstract/thesis/design |
| Kulzer 2023 | Only abstract/thesis/design |
| Lee 2014 | Wrong study design |
| Lei 2019 | Only abstract/thesis/design |
| Li 2016 | Only abstract/thesis/design |
| Li 2017 | Review |
| Li 2022 | Review |
| Liang 2021 | Review |
| Liang 2021 | Review |
| Lim 2023 | Only abstract/thesis/design |
| Lind 2020 | Only abstract/thesis/design |
| Lind 2021 | Wrong study design |
| Litchman 2020 | Review |
| Littlewood 2018 | Only abstract/thesis/design |
| Liu 2020 | Review |
| Long 2018 | Only abstract/thesis/design |
| Long 2018 | Only abstract/thesis/design |
| Long 2021 | Review |
| LozanoDelHoyo 2022 | Only abstract/thesis/design |
| Lutes 2018 | Only abstract/thesis/design |
| Mader 2020 | Only abstract/thesis/design |
| Mahr 2011 | Wrong outcome |
| Martyn-Nemeth 2020 | Only abstract/thesis/design |
| Massey 2019 | Review |
| Mathiesen 2019 | Review |

| | |
|---|---|
| Mathiesen 2023 | Review |
| McAuley 2020 | Only abstract/thesis/design |
| McGowan 2009 | Only abstract/thesis/design |
| Mizokami-Stout 2021 | Wrong Intervention |
| Munshi 2010 | Only abstract/thesis/design |
| Munshi 2011 | Only abstract/thesis/design |
| Naik 2023 | Wrong outcome |
| New 2013 | Only abstract/thesis/design |
| Newton 2013 | Only abstract/thesis/design |
| Ngan 2021 | Review |
| Ni 2021 | Review |
| Noordali 2017 | Review |
| Nurchis 2022 | Review |
| O'Connor 2023 | Only abstract/thesis/design |
| O'Neal 2020 | Only abstract/thesis/design |
| Oyedeji 2022 | Review |
| Pang 2023 | Wrong population |
| Pereira 2018 | Only abstract/thesis/design |
| Perrin 2019 | Review |
| Pfutzner 2014 | Only abstract/thesis/design |
| Philis-Tsimikas 2023 | Only abstract/thesis/design |
| Piatt 2021 | Only abstract/thesis/design |
| Pibernik-Okanovic 2013 | Only abstract/thesis/design |
| Piette 2013 | Wrong outcome |
| Plack 2010 | Review |
| Polonsky 2010 | Only abstract/thesis/design |
| Polonsky 2017 | Only abstract/thesis/design |
| Presswala 2018 | Only abstract/thesis/design |
| Pyatak 2017 | Only abstract/thesis/design |
| Pyatak 2023 | Only abstract/thesis/design |
| Quinn 2012 | Only abstract/thesis/design |
| Racey 2022 | Review |
| Rahmani 2013 | Only abstract/thesis/design |
| Reed 2024 | Wrong study design |
| Reimer 2017 | Wrong study design |
| Rogers 2014 | Wrong outcome |
| Saha 2024 | Wrong outcome |
| Samuel-Hodge 2006 | Only abstract/thesis/design |
| Schmidt 2018 | Review |
| Schmidt 2020 | Review |

| | |
|---|---|
| Sharp 2018 | Wrong outcome |
| Siaw 2015 | Only abstract/thesis/design |
| Siaw 2015 | Only abstract/thesis/design |
| Sieber 2016 | Only abstract/thesis/design |
| Sieber 2016 | Only abstract/thesis/design |
| Simmons 2013 | Only abstract/thesis/design |
| Simonson 2014 | Only abstract/thesis/design |
| Simonson 2015 | Only abstract/thesis/design |
| Simonson 2016 | Only abstract/thesis/design |
| Simonson 2017 | Only abstract/thesis/design |
| Skelly 2005 | Wrong outcome |
| Skinner 2010 | Wrong study design |
| Spaic 2017 | Only abstract/thesis/design |
| Spaic 2019 | Wrong population |
| Speight 2023 | Review |
| Sperl-Hillen 2010 | Only abstract/thesis/design |
| Sperl-Hillen 2011 | Only abstract/thesis/design |
| Sperl-Hillen 2012 | Only abstract/thesis/design |
| Spiess 1995 | Wrong outcome |
| Sturt 2015 | Review |
| Sudhir 2017 | Review |
| Swoboda 2018 | Only abstract/thesis/design |
| Takano 2017 | Only abstract/thesis/design |
| Tang 2015 | Only abstract/thesis/design |
| Tang 2022 | Wrong study design |
| Tasya 2021 | Review |
| TavaresFranquez 2023 | Review |
| Taylor 2005 | Wrong outcome |
| Taylor 2006 | Only abstract/thesis/design |
| Tolentino 2022 | Review |
| Trief 2006 | Wrong study design |
| Trief 2011 | Wrong outcome |
| Trief 2016 | Only abstract/thesis/design |
| Trief 2017 | Only abstract/thesis/design |
| Trief 2019 | Wrong population |
| Trump 2017 | Review |
| Uchendu 2017 | Review |
| Vakharia 2022 | Only abstract/thesis/design |
| VanAlphen 2007 | Only abstract/thesis/design |
| vanBeers 2017 | Wrong outcome |

| | |
|---|---|
| vanBon 2024 | Wrong Study Design |
| VanSon 2012 | Only abstract/thesis/design |
| vanVugt 2016 | Wrong study design |
| Verma 2022 | Review |
| Vieira 2020 | Review |
| Vlachou 2022 | Review |
| Wagner 2019 | Wrong study design |
| Walker 2013 | Only abstract/thesis/design |
| Wang 2024 | Review |
| Weinger 2004 | Review |
| Wermeling 2013 | Wrong study design |
| Westall 2023 | Only abstract/thesis/design |
| Wicaksana 2024 | Review |
| Wilmoth 2019 | Only abstract/thesis/design |
| Wilmoth 2022 | Only abstract/thesis/design |
| Winkley 2006 | Review |
| Winkley 2020 | Review |
| Winkley 2022 | Only abstract/thesis/design |
| Wong 2020 | Review |
| Yang 2018 | Only abstract/thesis/design |
| Yao 2014 | Only abstract/thesis/design |
| Yap 2024 | Review |
| Yu 2017 | Wrong outcome |
| Yu 2024 | Review |
| Zagarins 2010 | Only abstract/thesis/design |
| Zenasni 2022 | Only abstract/thesis/design |
| Zhang 2022 | Review |
| Zheng 2023 | Review |
| Zoffmann 2013 | Only abstract/thesis/design |
| Yanhong 2020 | Duplicate |

## 3. Baseline table

| Main paper, publication date and country | Study design, DD outcome measures and longest follow up time | Population sample (I/C) | Sex | Age | Type of diabetes (%) | Setting | Diabetes duration | Intervention and comparison group used in meta-analysis | Mean baseline data for DD ± SD | Mean baseline $HbA_{1c}$ in mmol/mol ± SD (% ± SD) | Subcategory other | Diverse specified |
|---|---|---|---|---|---|---|---|---|---|---|---|---|
| Abbas, 2023, Pakistan (1) | RCT; DDS; 3.7 months | 90 (45/45) | 44% male | Mean age 37 years | T2D | Hospital outpatient | 4.35 years | CBT on managing stress and coping with diabetes-related challenges. in-person. 8–10 sessions total, 1 within 10–12days, 45–60minutes, over 16 weeks vs wait list/CAU | I 16.5±2.7 C 15.9±2.8 | NR | | |
| Agarwal, 2019, Canada; USA (2) | RCT; PAID; 6 months | 223 (110/113) | 52% male | Mean age 51.8 years | T2D | Hospital-based diabetes education program | NR | Individually, online, BlueStar mobile app: Transtheoretical Model of Behaviour Change-based 3-month education & Informative personalised app vs CAU and waiting list | I 29.6 ±22.9 C 25.9±20.1 | I 73.7±19.9 mmol/mol (8.89±1.82%) C 75.2±16.7 mmol/mol (9.03±1.53%) | Telehealth | |
| Albabtain, 2024, Saudi Arabia; UK; USA; Qatar (3) | RCT; DD; 6 months | 160 (80/80) | 68.1% male | Mean age 50 years | T1D/T2D (NR) | Community pharmacy | 8.3 years | Medication therapy management (MTM): individual (in-person/phone) patient-centred pharmaceutical care cognitive service given by pharmacists, every 4, 8, 16 and 20 weeks plus 3-month and 6-month visits vs CAU | I 2.68±1.2 C 2.63±1.2 | I 85.8±23.1 mmol/mol (10±2.11%) C 84.4±17.4 mmol/mol (9.87± 1.59%) | Diverse | Complex healthcare |
| Alessi, 2021, Brazil (4) | RCT; B-PAID; 3.7 months | 91 (46/45) | 35.2% male | Mean age 61.3 years | T2D | Outpatient clinics | 18.1 years | Diabetes-specific individual telehealth intervention (16 weekly doses of 5–10min) vs active control (website with weekly posts on diabetes care and mental health) | I 21.0±2.8 C 18.0±24.1 | I 69.4±18.6 mmol/mol (8.5±1.7%) C 74.9±17.5 mmol/mol (9.0±1.6%) | | |
| Alessi, 2022, Brazil (5) | RCT; B-PAID; 3.7 months | 58 (29/29) | 50% male | Mean age 43.8 years | T1D | Outpatient clinics | 25.2 years | Individually, 16 week Weekly teleintervention of min 10 minutes telephone calls by health professional | NR | I 69.4±16.4 mmol/mol (8.5±1.5%) C | | |

| | | | | | | | | | | | | |
|---|---|---|---|---|---|---|---|---|---|---|---|---|
| | | | | | | | | plus online messaging group vs CAU | | 73.8±15.3 mmol/mol (8.9±1.4%) | | |
| Amiel, 2022, UK; USA (6) | RCT; PAID; 24 months | 99 (49/50) | 44.4% male | Mean age 54.3 years | T1D | Specialised type 1 diabetes clinic | 35.9 years | Hypoglycaemia Awareness Restoration Programme (HARP) face to face psychoeducational programme (4 group sessions, 2 individual sessions) vs Blood Glucose Awareness Training (BGAT) psychoeducational programme, group-based and individual | I 33.2±21.3 C 30.6±19.1 | I 57.4±12.0 mmol/mol (7.4±1.1%) C 57.4±14.2 mmol/mol (7.4±1.3%) | | |
| Amsberg, 2009, Sweden (7) | RCT; PAID; 11 months | 94 (46/48) | 49 % male | Mean age 41.2 years | T1D | University hospitals | 21.6 years | CBT on HbA1c, self-care behaviours and psychosocial factors in poorly controlled diabetes + 2x CGM for 72 hours, 8 weekly 2-hour basic program sessions, individual maintenance program sessions at week 12 and 24, telephone maintenance sessions at week 10, 16, 20, 32, and 42 and group maintenance sessions at week 12 and 24 vs wait list/CAU | I 31.1±20.4 C 33.4±17.3 | I 69.4±9.8 mmol/mol (8.5±0.9%) C 69.4±8.7 mmol/mol (8.5±0.8%) | | |
| Anderson, 2009, USA (8) | RCT; PAID; 12 months | 310 (156/154) | 41% male | Mean age 56 years | T2D | Primary care | 8.3 years | Empowerment based Diabetes-Self management Consultant. Face to face and telephone. Monthly contacts for 24 months and yearly face to face visit. vs written metabolic assessment results/CAU | I 28.3±21.3 C 28.2±22.6 | I 60.7±23.0 mmol/mol (7.7±2.1%) C 58.5±19.7 mmol/mol (7.5±1.8%) | Diverse | Complex healthcare |
| Anjali, 2023, India (9) | RCT; DDS; 3 months | 106 (53/53) | 68.9% male | Mean age 53.4 years | T2D | Diabetes clinic | NR | Diabetes self-management education (DSME) face to face, group based (2 sessions of 60min) vs CAU | I 2.0±0.5 C 2.4±0.7 | I 74.9±24.0 mmol/mol (9.0±2.2%) C 84.7±33.9 mmol/mol (9.9±3.1%) | | |
| Aronson, 2023, Canada(10) | RCT; DDS17; 6.2 months | 116 (58/58) | 63.8% male | Mean age 58.4 years | T2D | Diabetes specialist centres | 10 years | Individually, in person 16 weeks intermittently scanned continuous glucose monitoring plus diabetes self-management education (CGM + DSME) or DSME alone | I 2.3±1.1 C 2.3±1.2 | I 69.4±10.9 mmol/mol (8.5±1.0%) C 71.6±13.1 mmol/mol (8.7±1.2%) | Devices | |

| | | | | | | | | | | | | |
|---|---|---|---|---|---|---|---|---|---|---|---|---|
| Bae, 2024, Korea (11) | RCT; DDS2; 3.7 months | 73 (39/34) | 51.3% male | Mean age 62.2 years | T2D | Public health centres | 10 years | DangDang Care application for individual-based psychological support and diabetes self-management plus usual care (daily) vs CAU | I 6.4±1.9 C 5.7±2.6 | I 50.8±6.6 mmol/mol (6.8±0.6%) C 57.4±21.9 mmol/mol (7.4±2.0%) | | |
| Baig, 2024, USA (12) | Cluster RCT; DDS; 12 months | 195 (75/120) | 39.5% male | Mean age 53 years | T2D | Community health centres | NR | Diabetes group visits, group 2–3h visits per month for 6 months plus optional text-messaging of 25-week interactive type 2 diabetes text-message program, in-person vs wait list/CAU | NR | I 79.2±17.5 mmol/mol (9.4±1.6%) C 82.5±20.8 mmol/mol (9.7±1.9%) | | |
| Bajaj, 2016, Canada (13) | RCT; DDS; 2.8 months | 139 (72/67) | NR | Mean age 56.4 years | T2D | Diabetes + Endocrinology centres | 12 years | Individually, web-based LTHome tool: Insulin self-administration instructions (bedtime only dosing) & lifestyle modification counselling by non-health professionals (visits every 4 weeks + phone calls) vs enhanced usual therapy | NR | I 97.8±65.6 mmol/mol (11.1±6.0) C 58.5±82.0 mmol/mol (12.9±7.5%) | Telehealth | |
| Baldwin, 2020, Australia (14) | RCT; DDS; 12 months | 780 (397/383) | 35.7% male | Mean age 58 years | T2D | Community-based | 11.0 year | CBT-based electronic program mental health modules and self-monitoring, access for 8 weeks, followed by a 4-week-only self-monitoring. Online. Individual. vs Healthy Lifestyles electronic modules w/o CBT | I 2.5±0.9 C 2.5±0.9 | I 45±6 mmol/mol (6.3±2.7%) C 46±6 mmol/mol (6.3±2.7%) | Lifestyle | |
| Basak Cinar, 2014, Denmark (15) | RCT; PAID; 16 months | 186 (77/109) | NR | NR | T2D | Outpatient clinics | 11.8 years | Health coaching (5–6 in-person sessions and 3–4 phone sessions over 10 months) vs formal health education, in-person and by phone, individual | I 39.8±10.5 C 38.9±13.5 | I 58.5±16.4 mmol/mol (7.5±1.5%) C 61.7±17.5 mmol/mol (7.8±1.6) | Education | |
| Beck, 2017, USA (16) | RCT; DDS17; 6 months | 158 (79/79) | 56.5% male | Mean age 60 years | T2D | Endocrinology practices | 17.5 years | Individually, in person, CGM for 24 weeks vs CAU | I 1.9±0.8 C 2.0±0.8 | I 69.4±6.6 mmol/mol (8.5±0.6%) C 69.4±7.7 mmol/mol (8.5±0.7%) | Devices | |
| Bendig, 2021, Netherlands (17) | RCT; PAID; 1.8 months | 42 (21/21) | 50% male | Mean age 46.9 years | T1D/T2D (76%/24%) | Clinical environment | NR | ACT-based internet intervention for diabetes distress. individually. one module per week for 7 weeks, about 45–60 min vs wait list /CAU | I 45.1±13.8 C 42.6±13.4 | NR | | |

| | | | | | | | | | | | | |
|---|---|---|---|---|---|---|---|---|---|---|---|---|
| Benhalima, 2024, Belgium (18) | RCT; PAID-5; 6 months | 95 (46/49) | 0% male | Mean age 30.5 years | T1D | Secondary and primary care | 17 years | Individual advanced hybrid closed loop therapy during pregnancy vs standard insulin therapy during pregnancy | I 5.8±4.4 C 5.3±4.3 | I 47.5±6.6 mmol/mol (6.5±0.6%) C 47.5±7.7 mmol/mol (6.5±0.7%) | Devices | |
| Beverly, 2013, USA (19) | RCT; PAID; 12 months | 134 (67/67) | 49 % male | Mean age 59 years | T2D | Diabetes clinics | 13 years | Group map based program US Diabetes Conversation maps (4 sessions of 1 hour), in person vs group didactic education | I 33.3±20.3 C 34.8±23.1 | I 69.4±15.3 mmol/mol (8.5±1.4%) C 67.2±10.9 mmol/mol (8.3±1.0%) | | |
| Binesh, 2023, Iran (20) | RCT; DDS; 3 months | 30 (15/15) | 56% male | Mean age 56 years | T2D | University | 11 years | Occupational Therapy Diabetes Self-Management (10 sessions of 45 - 150min) combined individual + group, in-person and remote both vs Diabetes self-management education | I 2.1±0.8 C 2.3±0.8 | I 70.5±10.9 mmol/mol (8.6±1.0%) C 71.6±9.8 mmol/mol (8.7±0.9%) | Education | |
| Bisno, 2022, USA (21) | RCT; DDS; 12 months | 58 (23/35) | 33% male | Mean age 20.4 years | T1D | Diabetes centre | 10 years | Telehealth plus virtual group appointments (individual and group-based, online, 3 telehealth visits of 30 min each, 1 in-person visit over 12 months, 4 virtual group-appointments) vs only telehealth | I 1.9±0.9 C 1.9±0.9 | I 70.5±15.3 mmol/mol (8.6±1.4%) C 70.5±14.2 mmol/mol (8.6±1.3%) | Telehealth | |
| Bock, 2019, USA (22) | RCT; PAID; 9 months | 48(24/24) | 37.5% male | Mean age 55.7 years | T2D | Hospital-based gym-type facility | NR | Group, in-person meditation, breath-work class, 2 per week × 60 minutes vs Moderate intensity group exercise class, 5-min warm-up, 50-min moderate aerobic exercise, 5-min cool-down, 2 per week × 60 minutes | I 40.6±23.5 C 34.5±23.1 | I 67.2±17.5 mmol/mol (8.3±1.6%) C 68.3±12.0 mmol/mol (8.4±1.1) | Lifestyle | |
| Bolinder, 2016, Sweden (23) | RCT; DDS17; 6 months | 241 (120/121) | 57% male | Mean age 47.5 years | T1D | 23 European diabetes centres | 20 years | Individually, in person, flash sensor-based glucose monitoring vs self-monitoring of blood glucose with capillary strips for 6 months | NR | I 50.8±5.5 mmol/mol (6.8±0.5%) C 50.8±6.6 mmol/mol (6.8±0.6%) | Devices | |
| Bond, 2010, USA (24) | RCT; PAID; 6 months | 62 (31/31) | 55% male | Mean age 68 years | T2D | Hospital and community clinics | 17 years | Web-based intervention, including online weekly discussion groups and chats with the nurse both individual and groups-based vs CAU | I 2.3±0.9 C 2.1±0.8 | NR | | |

| | | | | | | | | | | | | |
|---|---|---|---|---|---|---|---|---|---|---|---|---|
| Boscari, 2022, Italy (25) | RCT cross-over; DDS; 3 months | 16 (16/16) | 81.3% male | Mean age 48.8 years | T1D | University Hospital | 29.8 years | Implanted continuous glucose monitor (Eversense, EVS) with monthly data download + individual, in-person clinic visits for inspection vs Wearable glucose sensor (Dexcom G5, DG5), Choice of receiver or app w/ same clinic visits | I 2.41±1.29 C 2.65±1.07 | I 57.4±8.7 mmol/mol (7.4±0.8%) C 57.4±9.8 mmol/mol (7.4±0.9 %) | Devices | |
| Brennan, 2021, Australia (26) | RCT; PAID; 2.8 months | 117 (58/59) | 44% male | Mean age 45 years | T1D | University | 20 years | Type 1 TACTICS for exercise (2 sessions of 1 and 3 hours, plus peer-led Facebook group) in-person, group-based vs CAU | NR | NR | | |
| Brinkworth, 2016, Australia (27) | RCT; PAID; 12 months | 115 (58/57) | 57.4% male | Mean age 58 years | T2D | Clinical research unit | I 7; C 9 years | Individually, in person, Hypocaloric diets: Personalised meal plans (plus food vouchers) with fortnightly dietitian visits (first 12 weeks), then monthly & Supervised 3x/week 60-minute aerobic + resistance training: Low-Carb (LC) vs High-Carb (HC) | I 22.7±2.0 C 22.3±2.1 | I 56.3±12.0 mmol/mol (7.3±1.1%) C 57.4±12.0 mmol/mol (7.4±1.1%) | Lifestyle | |
| Browne, 2016, Australia (28) | RCT; PAID; 6 months | 56 (27/29) | 68% male | Mean age 56 years | T2D | Hospital-based outpatient clinics | 8 years | Community-based integrated diabetes care (IDEAS): Individual & in-person integrated healthcare in one setting by multidisciplinary team vs CAU | I 27.18±20.04 C 29.25±23.50 | I 70.6±14.9 mmol/mol (8.61±1.36%) C 81.5±22.0 mmol/mol (9.61±2.01%) | Diverse | Complex healthcare |
| Bruijnes, 2023, Netherlands (29) | RCT; DDS; 0.7 months | 156 (79/77) | 52.6% male | Mean age 38.6 years | T1D/T2D (NR) | University | NR | Interactive conversational agent online, individual (3 sessions each separated by at least one week) vs psycho-education | I 2.7±1.1 C 2.4±1.0 | NR | | |
| Burner, 2017, USA (30) | RCT; PAID; 3 months | 44 (22/22) | 43% male | NR | T1D/T2D (NR) | University hospital | NR | Individual online TExT-MED plus Family and Friends Network Support (1–2 text messages per day) vs TExT-MED plus a mailed pamphlet with the same information | I 49.0±31.5 C 45.9±31.5 | I 90.2±21.9 mmol/mol (10.4±2.0 %) C 86.9±21.9 mmol/mol (10.1±2.0%) | | |
| Callan, 2022, USA (31) | RCT; DDS+PAID; 3.7 months | 12 (3/3/3/3) | 25% male | Mean age | T2D | Internal medicine clinic | 12.33 years | Phone-delivered individual CBT (6 (Ia), 8 (Ib), or 12 (Ic) weekly sessions of 30 min) | DDS Ia 3.85±0.96 Ib 3.35±0.24 Ic | Ia 74.5±5.4 mmol/mol (9.0 ±0.5%) Ib | | |

| | | | | | | | | | | | | |
|---|---|---|---|---|---|---|---|---|---|---|---|---|
| | | | | 57.2 years | | | | augmented with a CBT skills practice smartphone application. T2DM self-management and diabetes distress. Vs. CAU (C) | 4.52±0.16 C 3.86±0.94. PAID Ia 42.50±19.5 Ib 60.42±14.5 Ic 75.42±11.2 C 51.9±18.6 | 107.3±19.6 mmol/mol (12±1.8%) Ic 89.8 ± 19.6 mmol/mol (10.4±1.8%) C 95.4 ±15.3 mmol/mol (10.9 ±1.4%) | | |
| Carreira, 2023, Spain (32) | RCT; DDS; 2 months | 65 (35/30) | 31% male | Mean age 36 years | T1D | Secondary and tertiary care | 19.9 years | CBT-based Individual online program focused on depressive symptoms. 9* 20–30 min sessions. vs wait list/CAU | I 2.61±0.94 C 2.44±0.95 | I 66.3±16.5 mmol/mol(8.2±1.5% ) C 67.3±16.2 mmol/mol (8.3±1.5%) | | |
| Chan, 2014, Hong Kong (33) | RCT; C-DDS 15; 12 months | 628 (312/316) | 56.5% male | Mean age 54.7 years | T2D | Hospital-based diabetes clinics | 9.4 years | PEARL: Individual one-on-one peer supporters providing phone calls biweekly for 3 months, then monthly next 3 months, then every other month within the initial in-person group introduction & JADE: Regular risk assessments, personalised health reports, nurse-led education, patient empowerment, peer-support training vs only JADE | I 43.1± 15.0 C 41.2±14.6 | I 66.1±18.6 mmol/mol(8.2±1.7% ) C 66.1±17.5 mmol/mol (8.2± 1.6%) | Peer support | |
| Cheng 2021, China (34) | RCT; DDS; 3 months | 242 (121/121) | 74% male | Mean age 55 years | T2D | Tertiary teaching hospitals | 7.97 years | Empowerment-based integrative education program, 6 week weekly program, face-to-face small group + four phone-based individualised consultation and maintenance sessions. Vs. attentional control care/CAU | I 2.67±0.81 C 2.87±0.84 | I 84.7±43.7 mmol/mol(9.9±4%) C 88.0±43.7 mmol/mol (10.2±4%) | Telehealth | |
| Chew, 2019, Malaysia (35) | Cluster RCT; DDS; 12 months | 295 (145/150) | 39% male | Mean age 55.7 years | T2D | Public health clinics | 7 years | Emotion focused educational program VEMOfit (4 biweekly group sessions of 2h plus booster and follow-up session) vs attention control programme (3 sessions of 2h in total) | I 3.4±0.6 C 3.1±1.0 | I 84.7±19.7 mmol/mol(9.9% ± 1.8%) C 79.2±20.8 mmol/mol (9.4±1.9%) | | |

| | | | | | | | | | | | | |
|---|---|---|---|---|---|---|---|---|---|---|---|---|
| Chiu, 2016, Taiwan (36) | RCT; PAID; 8 months | 182 (91/91) | 51.7% male | Mean age 64.6 years | T2D | Medical centre | 10.5 years | Minimal psychological intervention via telephone. Nurses called over a period of 6 weeks 3–4 phone calls lasting 30–60 minutes each time on lifestyle, routines, health. Vs. CAU | I 5.6±7.6 C 5.4 ± NR | I 59.6±14.2 mmol/mol (7.6±1.3%) C 60.7±15.3 mmol/mol (7.7±1.4%) | | |
| Clark, 2020, USA (37) | RCT; DDS; 6 months | 126 (63/63) | 25% male | Mean age 48.5 years | T2D | Neighbourhood Healthcare clinics | NR | Dulce Digital: 2–3 text messages/day (motivational, educational, call-to-action), tapering over time, if out-of-range glucose then follow-up phone calls for 6m. Vs. CAU | I 2.32±1.32 C 2.53±1.24 | NR | Education | |
| Coccaro, 2022, USA (38) | RCT; DDS-RRD; 2.8 months | 20 (10/10) | 25% male | Mean age: 54.4 years | T2D | Diabetes centre | 15.9 years | 10–12 weekly individual in-person 45-minute Emotion-Focused Behavioural Therapy sessions. CAU | DD I: 3.6±1.4 C: 3.6±1.5 | I 83.6±16.4 mmol/mol(9.8±1.5% ) C 78.1±28.4 mmol/mol (9.3±2.6%) | | |
| Cohen, 2024, Canada (39) | RCT cross-over; DDS; 19.7 months | 15 (15/15) | 40% male | Mean age 39.4 years | T1D | University Health Centre | 22 years | Individually, combined 14 days In-person (for setup) + Remote monitoring plus days 1, 3, and 8 (+/–2 days) follow-up: carbohydrate counting insulin doses using their insulin-to-carb ratios vs Meal announcement + insulin & pramlintide dose delivery based on meal size vs only meal announcement | NR | NR | Lifestyle | |
| Cohn, 2014, USA (40) | RCT; DDS; 1.8 months | 49 (29/20) | 49.1% male | Mean age 54 years | T2D | Diabetes education centre | NR | Online individual positive affect skills training program, with psychoeducational content, exercises, and activities aimed at enhancing positive affect. Self-paced over 8-week . vs CAU | NR | NR | | |
| Cornely, 2022, USA (41) | RCT; DDS; 3 months | 20 (10/10) | 21% male | Mean age 56.4 years | T2D | Family Medicine Clinic | 13.3 years | Web-based CBT motivational interviewing intervention. Six 1-h sessions, every 2 weeks for 3 months, focusing on food planning education (15 min) and culturally | I 2.57 ± NR C 2.35 ± NR | NR | | |

| | | | | | | | | | | | | |
|---|---|---|---|---|---|---|---|---|---|---|---|---|
| | | | | | | | | tailored CBT/MI presentations and interactive activities (45 min) vs in-person format | | | | |
| Cox, 2021, USA (42) | RCT; DDS; 2 months | 178 (40/39/51/48) | 62.1% male | Mean age 56 years | T2D | Clinical environment | 9.2 years | Glucose Excursion Minimisation (GEM), 4 in-person group behaviour change and self-management sessions, 1 week between sessions 1–2, 3 weeks between sessions 2–3 and 3–4: GEM-S: BG self-monitoring before/after meals & physical activity vs GEM-C: Real-time BG feedback using CGM (Dexcom G5) vs GEM-D: Educational only (no BG monitoring vs Standard behavioural weight loss program plus food logs, 6 weekly in-person group sessions (1 hour each) | I 2.71±1.23 C 2.7±1.08 | I 67.9±14.5 mmol/mol (8.4±1.3%) C 66.6±10.8 mmol/mol (8.2± 1%) | Diverse | Insulin titration |
| Crawford, 2019, Australia (43) | RCT; DDS; 3 months | 88 (37/51) | 21% male | Mean age 53.8 years | T1D/T2D (64%/36%) | Online recruitment | 17.69 years | Individual online writing platform for positive writing regarding diabetes, 15-minute online writing sessions for 3 consecutive days. vs. same protocol but writing positive feelings/thoughts | I 1.74±0.66 C 1.73±0.63 | NR | | |
| Crowley, 2022, USA (44) | RCT; DDS; 12 months | 200 (101/99) | 77.5% male | Mean age 57.8 years | T2D | Outpatient veterans | 12.1 years | Telehealth intervention aimed at poorly controlled diabetes (26 bi-weekly sessions with 5 components both in-person and application, individual vs Telemonitoring (daily data transmission and 26 scheduled meetings) | I 1.9±0.7 C 1.9±0.9 | I 86.9±13.1 mmol/mol (10.1±1.2%) C 88.0±15.3 mmol/mol (10.2±1.4%) | Telehealth | |
| D'Eramo Melkus, 2010, USA (45) | RCT; PAID; 24 months | 109 (57/52) | 0% male | Mean age 46 years | T2D | Primary care | NR | Nurse-led and culturally relevant group-based diabetes self-management training and coping skills training (11 weeks, first 6 sessions 2h duration) vs conventional group-based | I 54±31 C 60±30 | I 63.9±23.0 mmol/mol (8.0±2.1%) C 67.2±24.0 mmol/mol (8.3±2.2%) | Education | |

| | | | | | | | | | | | | |
|---|---|---|---|---|---|---|---|---|---|---|---|---|
| | | | | | | | | diabetes education and Q&A, in-person | | | | |
| Dale, 2009, UK (46) | RCT; PAID; 6 months | 231 (90/44/97) | 55.4% male | NR | T2D | General practices | NR | (Ia): one-on-one phone-call-based support by trained peer supporters scheduled at ~6 time points including motivational interviewing, tracking goals, lifestyle change vs (Ib):phone-call-based support by diabetes specialist nurses (same schedule) vs (C): CAU | Ia 14.6±12.7 Ib 22.7±18.8 C 19.8±15.5 | Ia 68.3±12.0 mmol/mol (8.4 ±1.1%) Ib 73.8±16.4 (8.9±1.5%) C 71.6±14.2 mmol/mol (8.7±1.3%) | Peer support | |
| Danne, 2018, Germany (47) | RCT; DDS2; 12 months | 782 (261/263/258) | 51.9% male | Mean age 41.2 years | T1D | International phase 3 multicentre | 18.4 years | Individually, in person oral sotagliflozin 200 mg (Ia) vs 400 mg (Ib) vs placebo (C), two tablets/day before the first meal. | Ia 5.6±2.03 Ib 5.5±2.19 C 5.3±2.11 | Ia 61.1±8.8 mmol/mol (7.7±0.8%) Ib 60.8±9.0 mmol/mol (7.7±9.0) C 61.6±9.6 (7.8±0.9%) | Diverse | Medication |
| Davies, 2008, UK (48) | RCT; PAID; 36 months | 1109 (621/488) | 55% male | Mean age 59.5 years | T2D | Primary care | NR | Group diabetes education and self-management for ongoing and newly diagnosed (DESMOND) 1×6h session vs CAU | NR | I I 86.9±13.1 mmol/mol(8.3±2.2% ) C 88.0±15.3 mmol/mol (7.9±2.0%)(8.3±2.2 %) C I 63.9±23.0 mmol/mol(8.3±2.2% ) C 67.2±24.0 mmol/mol (7.9±2.0%) (7.9±2.0%) | | |
| De Groot, 2019, USA; India; Canada (49) | RCT; DDS; 12 months | 140 (36/34/34/36) | 23.57% male | Mean age 56 years | T2D | Community organizations | NR | CBT (10 sessions occurring over 12 weeks, EXER (12 weeks of community-based exercise including six sessions with a personal trainer), CBT+EXER (concurrent over a 12-week period) vs CAU | NR | NR | | |
| Dennick, 2015, UK (50) | RCT; PAID; 3 months | 41 (23/18) | 61% male | Mean age 65.6 years | T2D | Primary care | 7 years | Written emotional disclosure (write about stressful event over the last month and feelings) vs neutral writing, both groups wrote for 20min on three days within one week (control group only neutral | I 37.1±2.5 C 34.4±2.3 | I 53.0±10.9 mmol/mol (7.0±1.0%) C 51.9±12.0 mmol/mol (6.9±1.1%) | Education | |

| | | | | | | | | | | | | |
|---|---|---|---|---|---|---|---|---|---|---|---|---|
| | | | | | | | | description of events), individual, on-paper/typing | | | | |
| DiNardo, 2022, USA (51) | RCT; PAID; 5.5 months | 132 (65/67) | 91.7% male | Mean age 60.7 years | T1D/T2D (6%/94%) | Healthcare System for veteran care | 13.0 years | Integrated mindfulness intervention, 1 face to face group session mindfulness intervention integrated into a DSMES plus one booster session + individual practice mindfulness at home +24 weeks mobile application support. vs one 3-hour comprehensive DSMES group session | I 34.5±19.3 C 39.1±20.9 | I 70.5±16.4 mmol/mol (8.6±1.5%) C 71.6±17.5 mmol/mol (8.7±1.6%) | | |
| Ding, 2015, USA (52) | RCT; PAID; 12 months | 40 (18/22) | 55% male | Mean age 51 years | T2D | Academic institutions | 9 years | Combined, in person, weight Achievement and Intensive Treatment (Why WAIT): intensive medical diabetes and weight management (IMWM; Ia), multidisciplinary team 2-hour weekly group education on lifestyle vs laparoscopic adjustable gastric band (LAGB; Ib) | Ia 44.2± 13.6 Ib 48.3±15.1 | Ia 68.3±12.0 mmol/mol (8.4±3.2%) Ib 65.0±13.1 mmol/mol (8.1±3.3%) | Lifestyle | |
| Dinneen, 2013, Ireland (53) | RCT; PAID; 18 months | 437 (216/221) | 46% male | Mean age 40.8 years | T1D | Outpatient clinics | 15.9 years | Dose Adjustment for Normal Eating (DAFNE) structured group education programme, followed by 2 (3h) booster sessions at 6 and 12 months post-DAFNE vs DAFNE followed by 2 one-to-one clinic visits | I 30±18.2 C 29.8±18.2 | I 68.3±15.3 mmol/mol (8.4± 1.4%) C 66.1±14.2 mmol/mol (8.2±1.3%) | Lifestyle | |
| Dobson, 2018, New Zealand (54) | RCT; DDS; 9 months | 366 (183/183) | 51.63% male | Mean age 47 years | T1D/T2D (35%/65%) | Primary & secondary care | 12.5 years | Individual, phone tailored text messages (9 months) to motivate and support with diabetes content (self-management and psychological support) vs CAU | I 3.37±1.5 C 3.03±1.48 | I 86.9±41.5 mmol/mol (10.1±3.8%) C 83.6±38.3 mmol/mol (9.8±3.5%) | | |
| Drion, 2015, Netherlands (55) | RCT; PAID; 3 months | 63 (31/32) | 63% male | Mean age 33 years | T1D | Hospital | 17 years | Individually, diabetes Under Control (DBEES) phone-based diabetes diary application entering diabetes-related self-care data vs standard paper diary | I 13.7±7 C 14.3± 8.5 | I 61.7±7.0 mmol/mol (7.8 ±2.8%) C 61.0 ±7.0 mmol/mol (7.7±2.8%) | Telehealth | |

| | | | | | | | | | | | | |
|---|---|---|---|---|---|---|---|---|---|---|---|---|
| Ehrmann, 2019, Germany (56) | RCT; T1-DDS; 5.3 months | 141 (75/66) | 60% male | Mean age 46 years | T1D | Hospital | 20.8 years | Individually, in-person real-time CGM for 22 weeks vs self-monitored blood glucose measurements | I 2.5±0.7 C 2.5±0.9 | I 59.6±10.9 mmol/mol (7.6±1.0%) C 57.4±10.9 mmol/mol (7.4±1.0%) | Devices | |
| Ewen, 2024, USA (57) | RCT; DDS; 2.8 months | 25 (12/13) | 100% male | Mean age 62.8 years | T2D | University | NR | Online peer-led (one certified diabetes care and education specialists and two peers) Diabetes Self-Management Education (10h over 6 weeks, online) plus Peer-Led Diabetes Self-Management Support (9hr over 6 weeks) vs enhanced usual care (six sessions of the Diabetes Self-Management Education) | NR | I 60.7±24.0 mmol/mol (7.7±2.2%) C 57.4±14.2 mmol/mol (7.4±1.3%) | | |
| Fisher, 2011, USA (58) | Cluster RCT; DDS; 12 months | 483 (256/227) | 53.2% male | Mean age 55.8 years | T2D | Primary care practice sites | 7.6 years | Individual, in person, Record a 7-point SMBG profile on each of three consecutive days prior to their quarterly physician visit vs enhance CAU | I 2.41± 0.98 C 2.25±0.88 | I 73.8±13.1 mmol/mol (8.9±1.2%) C 73.8±13.1 mmol/mol (8.9± 1.2%) | Devices | |
| Fisher, 2013, USA (59) | RCT; T1-DDS; 12 months | 392 (150/146/96) | 46.2% male | Mean age 56.11 years | T2D | Primary care and hospital clinics | 6.90 years | Computer-assisted self-management (CASM; Ia) 40-min, online + 4 phone calls at weeks 2, 4, 7, and 12 to check progress and problems + booster at 5m vs CASM + 60 in in person problem-solving therapy (CAPS; Ib) vs LEAP Ahead (Ic), a computer-administered minimal supportive intervention of 20-min, computer-delivered health risk appraisal | Ia 2.38±0.89, Ib 2.37±0.86, Ic 2.48±0.95 | Ia 56.7±17.7 mmol/mol (7.3 ±1.6%) Ib 57.9±16.7 mmol/mol (7.5±1.5%) Ic 57.9±18.7 mmol/mol (7.5±1.7%) | telehealth | |
| Fisher, 2018, USA; Canada (60) | RCT; DDS; 9 months | 345 (176/169) | 30.9% male | Mean age 45 years | T1D | Community & clinic | 24.6 years | KnowIt (Ia): an intensive education/behaviour change intervention vs OnTrack (Ib): an emotion-focused intervention. in-person & individual. Each group attended a full-day | Ia 2.87±0.63 Ib 2.90±0.60 | Ia 72.3 ±12.3 mmol/mol (8.8±1.13%) Ib 73.0 ±12.1(8.8±1.1%) | Education | |

| | | | | | | | | | | | | |
|---|---|---|---|---|---|---|---|---|---|---|---|---|
| | | | | | | | | workshop plus 4 online meetings over 3 months. | | | | |
| Frias, 2023, USA (61) | RCT; T2-DDS; 2.7 months | 192 (95/48/49) | 38% male | Mean age 54.3 years | T2D | Virtual across USA | 8.6 years | Individually, online, Fibre-enriched nutritional formula (Active) OR fibre- absent, iso-caloric, iso-protein nutritional formula (Placebo) (Nutritional formulas slowly escalated to a maximally tolerated dose of two shake packets, with at least one replacing a meal ) vs dietary advice | I 3.1± 0.9 C 3.1± 1.0 | NR | Lifestyle | |
| Friis, 2016, New Zealand (62) | RCT; DDS; 3 months | 71 (35/36) | 31.7% male | Mean age 44.37 years | T1D | Hospital | 16.84 years | 8 week mindful self-compassion program. Sessions lasted 2.5 hours and were delivered in person to groups of 8–12 people in 8 weekly sessions vs waitlist/CAU | I 3.16±0.88 C 2.35±0.63 | I 74.2±15.1 mmol/mol(8.9±1.4% ) C 64.0±13.3 mmol/mol (8.0±1.2%) | | |
| Furler, 2017, Australia (63) | Cluster RCT; PAID; 12 months | 266 (151/115) | 61.3% male | Mean age 49 years | T2D | Primary care practices | 9 years | Individually, in person, stepping Up model of care: theory based change in practice systems and reorientation roles of health professionals in the primary care diabetes team. enhancing role for the practice nurse in leading insulin initiation and mentoring by a nurse with diabetes educator credentials. vs CAU waitlist control | I 17.5±18.7 C 13.8±14.1 | I 73.0±13.1 mmol/mol(8.8±1.2% ) C 71.6±13.1 mmol/mol (8.7±1.2%) | Diverse | Complex healthcare |
| Furler, 2020, Australia (64) | RCT; PAID; 12 months | 299 (149/150) | 58.9% male | Mean age 60.1 years | T2D | General practices | 13.3 years | Flash Glucose Monitoring (FreeStyle Libre Pro) + GP Review: Individual check-ins every 3 months, In-person sensor application, GP clinic visits using “Check, Chat, Change” framework with Text/email reminders, optional online GP training. vs CAU | I 11±12 C 13±12 | I 73.8±21.9 mmol/mol(8.9± 2%) C 73.8±13.1 mmol/mol (8.9±2) | Devices | |
| Gabbay, 2006, USA (65) | RCT; PAID; 12 months | 332 (150/182) | 54.2% male | Mean age | T1D/T2D (NR) | Primary care clinics | 10.5 years | NCM: one-on-one in-person 45–60 min initial Nurse-led diabetes management visit, | NR | NR | Diverse | Complex healthcare |

| | | | | | | | | | | | | |
|---|---|---|---|---|---|---|---|---|---|---|---|---|
| | | | | 64.5 years | | | | then phone follow-up every 4 months (more often if needed) vs CAU | | | | |
| Gabbay, 2013, USA (66) | RCT; PAID; 24 months | 545 (232/313) | 42% male | Mean age 58 years | T2D | Primary care | NR | Individual motivational interviewing face to face sessions with diabetes nurse with telephone/ email support as required. Eight sessions in 24 months with empowerment change focus vs CAU | I 29±23 C 29±24 | I 72.9±26.0 mmol/mol (8.8±2.4%) C 75.4±24.8 mmol/mol (9.1% ± 2.3%) | | |
| Gagliardino, 2013, Argentina (67) | RCT; DDS; 12 months | 198 (93/105) | 50% male | Mean age 62 years | T2D | Local primary care institution | 6 years | Structured diabetes education course 4 weekly in-person group sessions (90–120 min each) + 1 reinforcement AND Peers support as relatable examples: 1-on-1 emotional and behavioural phone support weekly (first 6 months), Biweekly (months 7–9), Monthly (months 10–12), with In-person visits every 2 months vs only Structured diabetes education consists of four weekly teaching units (90–120?min each) and a reinforcement session at 6?months | I 2.3±1.2 C 2.3±1.1 | I 54.1±16.4 mmol/mol (7.1±1.5%) C 56.3±16.4 mmol/mol (7.3±1.5 %) | Peer support | |
| Gerber, 2023, USA (68) | RCT cross-over; DDS; 24 months | 221 (109/112) | 30.3% male | Mean age 55.2 years | T2D | Primary care clinics and community settings | 12.7 years | Telehealth Pharmacist, reviewing bio-parameters, individual visits every 2–3 months (30–60 min), both in-person and remote (3-way video/audio) + Health Coach Support: bi-monthly home visits & calls for monitoring, education, activity tracking vs usual care | NR | NR | Telehealth | |
| Giannoulaki, 2024, Greece (69) | RCT; DDS; 6 months | 61 (31/30) | 57% male | Mean age 48.3 years | T1D | Outpatient clinic | 25.6 years | Individually, in person, Saffron + Pro + prebiotic Supplement: 3 sachets daily vs placebo | I 3.3±0.8 C 3.2±0.7 | I 51.9±8.7 mmol/mol(6.9±0.8% ) C 54.1±8.7 mmol/mol (7.1±0.8 %) | Lifestyle | |

| | | | | | | | | | | | | |
|---|---|---|---|---|---|---|---|---|---|---|---|---|
| Glasgow, 2004, USA (70) | Cluster RCT; PAID; 12 months | 886 (469/417) | 51% male | Mean age 63 years | T2D | Primary care | NR | in-person/computerised, individual Diabetes Priority Programme (computerised assessment and development of a self-management action plan, participant received print-outs), ca. 30 minutes plus interaction with care manager vs computerised assessment also including printouts, but more general and not Physician-Recognition-Programme related | I 34.5±19.3 C 39.1±20.9 | I 70.5±16.4 mmol/mol(8.6±1.5% ) C 71.6±17.5 mmol/mol (8.7±1.6%) | | |
| Gonzalez, 2024, USA (71) | RCT; DDS; 12 months | 812 (408/404) | 43.0% male | Mean age 59.2 years | T2D | Primary care | 12.4 years | Individual, Telephonic Self-Management Support in addition to mailed printout educational material (max 6 vs max 12 phone calls depending on physiological and psychological well-being at baseline) vs Enhanced Usual Care (mailed print-out educational material) | I 1.8±0.7 C 1.8±0.8 | I 78.1±19.7 mmol/mol (9.3±1.8%) C 78.1±18.6 mmol/mol (9.3±1.7%) | | |
| de Grillo, 2016, Brazil (72) | RCT; B-PAID; 12 months | 137 (69/68) | 37% male | Mean age 62.4 years | T2D | Primary care unit | 10 years | DSME: 5 weekly in-person group sessions (2 hours each), 2 reinforcement sessions at months 4 and 8) led by nurse covering diabetes-related lifestyle, risk, complication, & foot care vs group meetings with a nurse w/o DSME | I 20±14 C 16±13 | I 72.7±20.8 mmol/mol (8.8±1.9%) C 76.0±21.9 mmol/mol (9.1±2.0%) | Education | |
| Griva, 2019, Singapore (73) | RCT; PAID; 2.8 months | 44 (20/24) | 48% male | Mean age 62.6 years | T1D/T2D (NR) | Dialysis centres | 19.03 months | bed-side 2 days group, 2 days individual sessions (30–60 min) C-DIRECT: based on Social Cognition Theory and Motivational Interviewing + haemodialysis management vs CAU | I 28.00±21.10 C 19.01±14.71 | I 81.1±15.0 mmol/mol (9.6±1.4%) C 81.6±15.3 mmol/mol ( 9.6±1.4%) | | |
| Guo, 2022, China (74) | RCT; DDS; 2.8 months | 100 (50/50) | 47% male | Mean age 62.4 years | T2D | Endocrinology ward of a tertiary hospital | NR | Mindfulness based stress reduction (MBSR), 8 daily in-person group sessions (120 min) AND 8 week online maintanance | I 3.6 ± 0.5 C 3.5±0.3 | I 63.0±18.1 mmol/mol (7.9±1.7%) C 62.8±16.2 mmol/mol (7.9±1.5%) | Education | |

| | | | | | | | | | | | | |
|---|---|---|---|---|---|---|---|---|---|---|---|---|
| | | | | | | | | (30min/d, 6 days/week) vs regular diabetes education (10–30 min/d Q&A with a diabetes educator) | | | | |
| Haak, 2017, Germany (75) | RCT; DDS; 6 months | 224 (149/75) | 66.9% male | Mean age 59 years | T2D | Diabetes centres | 17 years | Individually, in person, Flash Glucose Monitoring (Sensor-Based) vs CAU usual self-monitoring of blood glucose (SMBG) via fingerstick | NR | I 71.6±10.6 mmol/mol (8.7±1.0%) C 73.8±11.4 mmol/mol (8.9±1.0%) | Devices | |
| Halperin, 2014, USA (76) | RCT; PAID; 12 months | 43 (22/21) | 34.8% male | Mean age 51.6 years | T2D | Outpatient clinic and hospital | 10.4 years | Individually, in person, Roux-en-Y Gastric Bypass (RYGB) vs Why WAIT Program (Intensive Lifestyle & Medical Intervention), Weekly 2-hour group sessions (in-person) with Multidisciplinary team, focus on hypocaloric diet, physical activity, CBT (12-week intensive phase + 9-month maintenance) | I 52.63±16.38 C 56.18±12.59 | I 66.6±15.5 mmol/mol (8.2±1.4%) C 73.0±11.0 mmol/mol (8.8±1.0%) | Diverse | Surgery |
| Heinrich, 2010, Netherlands (77) | RCT; PAID; 24 months | 533 (274/259) | 55.1% male | Mean age 59 years | T2D | Primary care | NR | Face to face, individual motivational interviewing and supportive counselling with diabetes HCP. 8 _ 20 minutes sessions every 4 months for 2 years with a behaviour change focus vs CAR | I 16.83±13.32 C 16.98±13.92 | I 47.4±9.3 mmol/mol (6.5±0.9%) C 47.7±8.1 mmol/mol (6.5±0.7%) | | |
| Heisler, 2014, USA (78) | RCT; DDS; 3 months | 188 (93/95) | 29% male | Mean age 51.5 years | T2D | Community health centres | 9 years | Tailored, individual, interactive, web-based tablet computer iDecide tool (1–2 h session with a community health worker) vs printout material with 2 follow-up calls, in-person/telephone | I 62.7±28.3 C 68.0±26.5 | I 66.1±20.8 mmol/mol (8.2±1.9%) C 67.2±24.0 mmol/mol (8.3±2.2%) | Education | |
| Heisler, 2010, USA (79) | RCT; DDS; 6 months | 244 (125/119) | 100% male | Mean age 62.0 years | T1D/T2D (NR) | Veterans' Affairs healthcare facilities | NR | Individual and group, Motivational Interviewing (MI) and Empowerment-based techniques, 3-hour session + two 1-hour boosters with either: Nurse Care Management (NCM): 1.5-hour lab result review by a care manager vs Reciprocal Peer Support | I 26.5±16.4 C 26.4±19.8 | I 64.2±14.4 mmol/mol (8.0±1.3%) C 63.2±15.3 mmol/mol (7.9±1.4%) | Peer support | |

| | | | | | | | | | | | | |
|---|---|---|---|---|---|---|---|---|---|---|---|---|
| | | | | | | | | (RPS): 3-hour group session, age-matched peer reviewing after training in peer communication skills (with weekly calls) over 6 months | | | | |
| Hermanides, 2011, Netherlands (80) | RCT; PAID; 6 months | 83 (44/39) | 51.8% male | Mean age 38 years | T1D | Outpatient centres | 19 years | Individually, in person, for 26 weeks (Paradigm® REAL-Time) sensor-augmented insulin pump vs CAU continued with multiple daily injections | I 32.4±18.8 C 26.5±18.4 | I 69.1±10.3 mmol/mol (8.5±0.9%) C 70.9±9.4 mmol/mol (8.6±0.9%) | Devices | |
| Hermanns, 2007, Germany (81) | RCT; PAID; 6 months | 164 (84/80) | 50% male | Mean age 46 years | T1D | Outpatient centres | 21 years | Specific training programme in groups in person aimed at problems with hypoglycaemia (5 weekly sessions of 90 minutes) vs standardised, general diabetes education programme (4 weekly sessions of 90 minutes) | I 27.5±11.7 C25.5±16.6 | I 55.2±9.8 mmol/mol (7.2±0.9%) C 57.4±12.0 mmol/mol (7.4±1.1%) | | |
| Hermanns, 2012, Germany (82) | RCT; PAID; 6 months | 186 (92/94) | 55% male | Mean age 63 years | T2D | Outpatient centres | 13.5 years | MEDIAS 2 ICT education programme on lowering HbA$_{1c}$, 8 lessons of 90 min, in groups, in person vs standard education program | I 52.5±9.2 C 47.6±9.6 | I 68.3±16.4 mmol/mol(8.4±1.5) C 67.2±13.1 mmol/mol (8.3±1.2) | | |
| Hermanns, 2013, Germany (83) | RCT; DDS; 6 months | 160 (81/79) | 56% male | Mean age 45.5 years | T1D | Outpatient centres | 19.4 years | PRIMAS self-management focused education programme in type 1 diabetes 12 group lessons of 90 min in person vs established education program of 12 lessons | I 1.3±1.0 C 1.2±0.9 | I 67.2±12.0 mmol/mol (8.3±1.1%) C 65.0±10.9 mmol/mol (8.1±1.0%) | | |
| Hermanns, 2017, Germany (84) | RCT; PAID; 6 months | 182 (92/90) | 55% male | Mean age 64 years | T2D | Outpatient centres | 10 years | Education programme (MEDIAS 2 BSC) of non-intensive insulin treatment regimens 6 weekly lessons 90 min in groups in persons vs 6 lesson education program of 90 min | I 27.9±20.4 C 26.8±18 | I 63.9±14.2 mmol/mol(8.0±1.3% ) C 62.8±13.1 mmol/mol (7.9±1.2%) | | |
| Hermanns, 2009, Germany (85) | RCT cross-over; PAID; NR (monitor used twice) | 50 (50/50) | 52% male | Mean age 41.7 years | T1D | Diabetes centre | 14.75 years | Individually, in person for undetermined period CGM (Continuous Glucose Monitoring) outcome Real-Time Access (RTA) vs CGM | I 30.7±18.8 C 30.7±18.8 | I 65.0±16.4 mmol/mol (8.1±1.5%) C 65.0±16.4 mmol/mol (8.1±1.5%) | Devices | |

| | | | | | | | | | | | | |
|---|---|---|---|---|---|---|---|---|---|---|---|---|
| | | | | | | | | outcome with Retrospective Analysis (RA) | | | | |
| Hermanns, 2015, Germany (86) | RCT cross-over; PAID; 12 months | 343 (343/343) | 43% male | Mean age 43.3 years | T1D/T2D (64.5%/35.5% ) | Hospital inpatient | 14.2 years | Psycho-educational: Group based diabetes specific CBT with psychologist in 5×90 min sessions. Face to face and telephone support. Theory based with focus on mood and behaviour change vs group DSME | I 41.1±19.1 C 37.9±17.5 | I 72.7±18.6 mmol/mol (8.8±1.7%) C 71.6±18.6 mmol/mol (8.7± 1.7%) | Education | |
| Hermanns, 2015, Germany (87) | RCT; PAID; 12 months | 214 (106/108) | 43% male | Mean age 43.3 years | T1D/T2D (66%/34%) | Hospital inpatients | 14.2 years | Group based diabetes specific CBT with focus on mood and behaviour with psychologist in 5 x 90 min sessions. Face to face and telephone support. vs group DSME of five lessons (90 min each) that included topics such as healthy diet in diabetes, diabetes and exercise, and diabetes and legal issues. | I 39.7±19.6 C 37.5±17.0 | I 73.8±19.7 mmol/mol (8.9±1.8%) C 73.8±19.7 mmol/mol (8.9±1.8%) | | |
| Hernar, 2021, Norway (88) | RCT; DDS; 12 months | 79(39/40) | 49.3% male | Mean age 27.2 years | T1D | University hospital endocrinology outpatient clinic | 13.7 years | Individually, in person, PAID scale as a discussion and communication tool, two 30-minute nurse consultations within 4 weeks, second within 3 months focused on distress, self-management challenges, and goals vs CAU | I 2.1±0.69 C 2.1±0.71 | I 65.0±13.1 mmol/mol (8.1± 1.2%) C 66.1±15.3 mmol/mol (8.2± 1.4%) | Diverse | PROMS |
| Hessler, 2022, USA (89) | Cluster RCT; DDS2; 12 months | 734 (326/408) | 40% male | Mean age 54 years | T2D | Primary care | NR | Individual enhanced evidence-based SMS program for self-management support for diabetes, including health survey, goal setting +4 videos of 60 seconds + motivational interviewing in person and online vs evidence-based SMS program | I 1.4±1.1 C 1.2±1.1 | NR | Telehealth | |

| | | | | | | | | | | | | |
|---|---|---|---|---|---|---|---|---|---|---|---|---|
| Hessler, 2024, USA (90) | RCT; T1-DDS; 12 months | 296 (101/97/98) | 19% male | Mean age 47 years | T1D | University clinic for diabetes | 27 years | Three virtual group-based programs: Ia) Streamline, an educator-led education and diabetes self-management program, 3-h virtual workshop + five one-on-one personal phone calls ( 10–25 min every 2–3 weeks over a 3 months; Ib) TunedIn, a psychologist-led program focused exclusively on emotional-focused DD reduction, two 3-h virtual group workshops, four 1-h group Zoom calls, and one phone call with the group leader (10–15 min), and experience; or Ic) FixIt, an integration of Streamline and TunedIn | Ia 2.8±0.8 Ib 2.8±0.8 Ic 3.0±0.7 | Ia 66.1±9.8 mmol/mol 66.1±9.8 mmol/mol (8.2±0.9%) Ib (8.2±0.9%) Ic 67.2±12.0 mmol/mol (8.3±1.1%) | | |
| Hilmarsdöttir, 2021, Iceland (91) | RCT; PAID; 6 months | 37 (18/19) | 30% male | Mean age 51.2 years | T2D | Outpatient clinics | 6.3 years | Individual digital Lifestyle Program via app, support for 16 weeks vs CAU | I 19.5±16.5 C 17.5±16.2 | I 60.7±21.9 mmol/mol (7.7±2.0%) C 61.7±20.8 mmol/mol (7.8±1.9%) | | |
| Ho, 2020, USA (92) | Cluster RCT; DDS; 6 months | 18 (11/7) | 16.6% male | Mean age 62 years | T2D | Public Health Centre | NR | DSME with content modified by Chinese medicine principles (INC): 3 in-person weekly group sessions (2 hours), met with an acupuncturist at baseline (30 min), and provided tailored nutrition plans vs standard DSME only | I 1.8±0.3 C 2.1±0.3 | I I 65.0±13.1 mmol/mol (7.0±0.3%) C 66.1±15.3 mmol/mol (7.5±0.3%) (7.0±0.3%) C NR (7.5±0.3%) | Education | |
| Holland - Carter, 2017, USA (93) | RCT; DDS; 12 months | 563 (279/284) | 28.9% male | Mean age 55.1 years | T2D | Multiple sites ( e.g. community, primary care) | NR | Modified Weight Watchers (WW) for T2D: Group + Individual, In-person: Weekly community WW group meetings, Online: Access to WW tools and weekly T2D emails, 2 Phone/email consultations with a Certified Diabetes Educator (CDE) vs 1 in- | I 2.3±0.9 C 2.2±0.9 | I 68.3±10.9 mmol/mol (8.4±1.0%) C 67.2±10.9 mmol/mol (8.3± 1.0%) | Lifestyle | |

| | | | | | | | | | | | | |
|---|---|---|---|---|---|---|---|---|---|---|---|---|
| | | | | | | | | person nutrition counselling session at baseline w dietitian, written dietary guidance at follow-up visits, 500 kcal/day deficit hypocaloric diet & waitlist | | | | |
| Hoyo, 2022, Spain (94) | RCT; DDS; 12 months | 428 (225/203) | 28.2% male | Mean age 70 years | T2D | Population based | 10 years | Monthly telephonic interventions of 30 min delivered by specifically trained research nurses, based on psychoeducational individualised monitoring protocol including motivational interviewing and collaborative care strategies vs CAU | I 1.48±0.4 C 1.50±0.41 | I 71.6±15.3 mmol/mol (8.7±1.4%) C 71.6±16.4 mmol/mol (8.7±1.5%) | | |
| Ing, 2016, USA (95) | RCT; PAID; 6 months | 47 (25/22) | 51% male | Mean age 54 years | T2D | Community organizations | NR | Partners in Care (PIC):12 culturally adapted (NH/PI) weekly (1h) in-person group sessions, collaborative diabetes management (12 weeks) WITH SSG (Structured Support Group): 6 in-person bimonthly reinforcement group sessions (1h) by community facilitators, Health professionals vs 6 bimonthly postcards | NR | NR | Education | |
| Ishii, 2021, Japan (96) | RCT; PAID; 6 months | 120 (59/61) | 70.8% male | Mean age 62.5 years | T2D | Medical institutions | 13.45 years | Individually, in person, Insulin glargine U300 titration: Patient-led vs Physician-led | NR | NR | Diverse | Insulin titration |
| Ismail, 2018, UK (97) | RCT; DDS; 18 months | 334 (164/170) | 48.8% male | Mean age 58.9 years | T2D | Primary care | 9 years | In-person, individual motivational interviewing and CBT (Diabetes-6), 12×30min sessions over 12 months vs CAU | I 2.1 (95% CI 1.7–2.7 C 2.0 (95% CI 1.6–2.7 | I 81.4±40.4 mmol/mol (9.6±3.7%) C 80.3±42.6 mmol/mol (9.5±3.9%) | | |
| Iversen, 2020, Norway (98) | Cluster RCT; PAID; 48 months | 156 (78/78) | 73.7% male | Mean age 65.5 years | T1D/T2D (22.4%/77.6% ) | Three clinical sites | 20.8 years | Individually, telemedicine (TM): Web-based ulcer record system, remote with weekly virtual ulcer photo consultation, & tracking by specialist nurses vs CAU | I 21.6± 18.6 C 21.4± 18.3 | I 62.8±17.5 mmol/mol (7.9±1.6%) C 63.9±18.6 mmol/mol (8.0±1.7%) | telehealth | |
| Izquierdo, 2003, USA (99) | RCT; PAID; 3 months | 46 (24/22) | 46% male | Mean age 58 years | T1D/T2D (11%/89%) | Diabetes centre | 14 years | Diabetes Education Administered Through Telemedicine, consisting of | I 37.4±25.8 C 35.8±23.4 | I 71.6±23.0 mmol/mol (8.7±2.1%) C | | |

| | | | | | | | | | | | | |
|---|---|---|---|---|---|---|---|---|---|---|---|---|
| | | | | | | | | three consultative visits with diabetes nurse and nutrition educators, 30–60 min, individually vs in Person | | 70.5±17.5 mmol/mol (8.6±1.6%) | | |
| Jiang, 2021, China (100) | RCT; DDS; 12 months | 265 (133/132) | 45% male | Mean age 57 years | T2D | Hospital | 6 years | Self-efficacy-focused structured education programme | I 41.3±17.1 C 39.6±18.7 | I 70.5±13.1 mmol/mol (8.6±1.2%) C 72.7±16.4 mmol/mol (8.8±1.5%) | Education | |
| Josefsson, 2024, Sweden (101) | RCT; DDS; 2 months | 59 (28/31) | 71.1% male | Mean age 61.1 years | T2D | Healthcare centres | NR | App-based: Blood glucose, medication, and blood pressure values submitted once weekly to Sukaribit smartphone app for 2 months, physician monitored submissions and gave weekly feedback through the app vs CAU | I 2.45±0.83 C 2.00±0.73 | I 50.1±6.9 mmol/mol (6.7±2.8%) C 55.6±7.5 mmol/mol (7.2±2.8%) | telehealth | |
| Ju, 2018, China (102) | Cluster RCT; DDS; 12 months | 400 (200/200) | 34% male | Mean age 68.2 years | T2D | Community health centres | 9.45 years | Usual education: group 2 h/month of structured in-person individual diabetes education and external self-education & community focused peer-led monthly themed sessions on diabetes knowledge and self-management skills non-themed informal support via home visits, calls etc. vs CAU only usual education | I 1.3± 0.4 C 1.3±0.4 | I 50.8±16.4 mmol/mol (6.8± 1.5%) C 49.7±15.3 mmol/mol (6.7± 1.4%) | Education | |
| Jung, 2015, South Korea (103) | Cluster RCT; DDS; 1.8 months | 56 (21/18/17) | 48.2% male | Mean age 66.2 years | T2D | Outpatient clinics | 9 years | Korean mindfulness-based stress reduction in groups twice per week for 60–90 min (Ia), group walking 60 min per week + practice 4 times per week (Ib), vs patient education 1 per week for 8 weeks | Ia 54.3±12.9 Ib 48.8±15.9 C 56.8±11.6 | NR | Education | |
| Juul, 2014, Denmark (104) | RCT; PAID; 18 months | 4034 (2005/2029) | 56.5% male | Mean age 60.4 years | T2D | General practices | 8 years | In-person, individual, 6-hour Self-determination theory - based course delivered over 10 months for nurses+ visit 30 min to practice by teacher. Vs. CAU | NR | I 54.1±14.2 mmol/mol (7.1±1.3%) C 54.1±14.2 mmol/mol (7.1±1.3%) | | |

| | | | | | | | | | | | | | |
|---|---|---|---|---|---|---|---|---|---|---|---|---|---|
| Kakoschke, 2021, Australia (105) | RCT; PAID; 24 months | 115 (58/57) | 57% male | Mean age 58.5 years | T2D | Clinical research unit | 6.8 years | Individually, in person, Energy-restricted diets (500–1000 kcal/day deficit) with dietitian support (every 2 weeks for 12 weeks, then monthly) & 60 min sessions (aerobic + resistance) 3 days per week: Low-Carbohydrate (LC) Diet vs High-Carbohydrate (HC) Diet | DD I 22.67±1.70 C 22.33±1.72 | NR | Lifestyle | |
| Kampling, 2022, Germany (106) | RCT; PAID; 12 months | 178 (87/91) | 35.7% male | Mean age 48 years | T1D/T2D (48%/52%) | Clinical environment | 16.1 years | Psy-PAD is a low-threshold psycho-dynamically oriented short-term individual therapy program vs CAU | I 41.59±18.23 C 39.04±19.02 | I 72.7±13.1 mmol/mol (8.8±1.2%) C 70.5±12.0 mmol/mol (8.6±1.1%) | | |
| Karlsen, 2004, Norway (107) | RCT; PAID; 6 months | 63 (31/32) | 52% male | Mean age 49 years | T1D/T2D (30%/70%) | University | NR | Group-based counselling programme CBT approach, nine sessions of 90 min over 12months vs wait list/CAU | I 2.2±0.6 C 2.1±0.8 | I 61.7±13.1 mmol/mol (7.8±1.2%) C 68.3±14.2 mmol/mol (8.4±1.3%) | | |
| Kasteleyn, 2016, Netherlands (108) | RCT; PAID; 5 months | 201 (101/100) | 75.8% male | Mean age 65.8 years | T2D | 13 hospitals in three regions in NL | 8.0 years | 3 home visits of 60 min by diabetes nurses after first acute coronary event, motivational interviewing to increase self-efficacy regarding illness vs CAU + 1 consultation (15 min) by telephone, within 3 weeks after discharge | I 8.2±10.1 C 9.2±12.4 | I 55.2±38.3 mmol/mol (7.2±3.5%) C 50.8±33.9 mmol/mol (6.8±3.1 %) | Telehealth | |
| Katz, 2019, USA (109) | RCT; DDS; 12 months | 166 (56/56/54) | 27.7% male | Mean age 52.9 years | T2D | Outpatient clinics | 13.8 years | (Ia) Individually, Care4Life (C4L) mHealth System: cell phone-based automated messages for management-related education based on ADA guidelines (3/week), Glucose monitoring (6/week), & exercise/BP (1/week) vs (Ib) Community Health Worker (CHW) Support: One-on-one support during clinic visits, home visits, and by phone vs (Ic) C4L + CHW | Ia 16.6±5.31 Ib 16.1±5.98 Ic 16.9±6.03 | Ia 94.5±21.2 mmol/mol (10.8±1.9%) Ib 94.5 ±24.5 mmol/mol (10.8±2.2%) Ic 86.9±20.9 mmol/mol (10.1±1.9%) | Telehealth | |
| Kavin, 2010, USA (110) | RCT; PAID; 6 months | 100 (34/33/33) | NR | NR | T2D | Primary care | 9 years | Penn State Hershey Diabetes Playbook: Low-literacy book improving diabetes knowledge, | NR | NR | Education | |

| | | | | | | | | | | | | |
|---|---|---|---|---|---|---|---|---|---|---|---|---|
| | | | | | | | | emotional distress, self-care behaviour, and clinical outcomes + nurse tutorial (15–30 min) vs Penn State Hershey Diabetes Playbook only vs CAU | | | | |
| Kelly, 2023, UK (111) | RCT; DDS; NR | 98 (49/49) | 51% male | Mean age 53 years | T1D/T2D (NR) | Outpatient routine care | NR | Individually, Spotlight-AQ tool ( (?3.5 min), smart, adaptive digital assessment tool) used prior to routine appointment (in-person or remote) every 3 months & reviewed during the appointment vs CAU | NR | I 62.8 ± NR mmol/mol (7.9 ± NR%) C 66.1 ± NR mmol/mol (8.2 ± NR%) | telehealth | |
| Kempainen, 2023, USA (112) | RCT; DDS; 6 months | 214 (106/108) | NR | Mean age 55.6 years | T2D | Community | NR | FOODRx: Home-delivered food boxes every 2 weeks (12 total) & Recipes + diabetes education materials, Pre- and post-delivery phone calls for coordination vs CAU | I 2.81 ± NR C 2.59 ± NR | I 63.8 ± NR mmol/mol (8.90 ± NR%) C 62.7 ± NR mmol/mol (7.9 ± NR%) | Lifestyle | |
| Kempf, 2013, Germany (113) | RCT; PAID; 2.8 months | 220 (120/100) | 45.9% male | Mean age 61 years | T2D | Community | <5 years | Individually, online, Nintendo Wii Fit Plus: home-based individual exercise game, ?30 min/day vs CAU (waitlist) | NR | I 54.1±14.2 mmol/mol (7.1±1.3%) C 50.8±9.8 mmol/mol (6.8±0.9%) | Lifestyle | |
| Kiliç, 2023, UK; Northern Ireland (114) | RCT; PAID; 2 months | 33 (19/14) | 15.1% male | Mean age 55.85 (10.34 ) years | T2D | Online recruitment (research) | 10.24 years | Individual self-guided ACT & MSC-based five-weekly sessions delivered online (~30 min each +10Ð15 min optional daily practice) vs wait list/CAU | I 42.50±16 C 45.42±19.86 | NR | | |
| Kleinman, 2017, USA (115) | RCT; PAID; 6 months | 91 (44/47) | 86.5% male | Mean age 48.4 years | T2D | Diabetes clinic | 9 years | M-health diabetes platform, including med review and reminders individually vs wait list/CAU | I 8.7±4.1 C 11.4±6.0 | I 79.2±13.1 mmol/mol( 9.4±1.2%) C 76.0±12.0 mmol/mol (9.1±1.1 %) | | |
| Kunik, 2023, USA (116) | RCT; DDS; 6 months | 218 (110/108) | 88.5 % male | Mean age 63 years | T2D | Veterans Affairs medical centre and community agencies | NR | iNSPiRED: weekly (20–30 min) Peer-led support calls + navigation in DM, mental health, and basic needs resources for veterans vs CAU | I 3.04±1.11 C 3.06±1.07 | NR | Peer support | |
| Kwan, 2024, USA (117) | RCT cluster; DDS; 9 months | 1060 (502/558) | 41.1% male | Mean age 60.3 years | T2D | Primary care practice | NR | Group-based, in-person shared medical appointments, aka SMAs, where patient selects the topic 6 sessions (2 h), | I 2.11±0.96C 2.20±0.91 | I 67.5±22.7 mmol/mol (8.3±2.1%) C 67.0±21.4 mmol/mol (8.3± 2.0%) | Diverse | Complex healthcare |

| | | | | | | | | | | | | |
|---|---|---|---|---|---|---|---|---|---|---|---|---|
| | | | | | | | | standardised (by health educator) visits with a provider for chronic condition care (SMAs) vs Patient-selected sessions (by multidisciplinary team) SMAs | | | | |
| Lamers, 2011, Netherlands (118) | RCT; PAID; 9 months | 208 (105/103) | 49% male | Mean age 70 years | T2D | Primary care | 9 years | A nurse-administered, minimal psychological intervention, with elements of CBT and self-management, Individual, face to face, variable amount of sessions s vs CAU | I 22.6±20.5 C 23.4±20.5 | I 58.5±13.1 mmol/mol (7.5±1.2%) C 55.2±15.3 mmol/mol (7.2±1.4%) | | |
| Lamptey, 2023, Ghana (119) | RCT; PAID; 3 months | 206 (103/103) | 31% male | Mean age 58 years | T2D | public primary facilities | 5 years | Structured diabetes self-management education program, adapted from DESMOND, 1 session of 6h in person in a group in person vs CAU | I 4±1 C 5±2 | I 66.1±71.0 mmol/mol (8.2±6.5%) C 59.6±68.9 mmol/mol (7.6±6.3%) | | |
| Laurberg, 2022, Denmark (120) | RCT; PAID; 15 months | 320 (160/160) | 52.8% male | Mean age 48 years | T1D | Outpatient clinic | NR | DiabetesFlex: Patient-controlled, outcome-based telehealth (flexible, as needed) vs CAU | I 16.0 ± 13.2 C 16.3 ± 11.9 | I 57.8±11.5 mmol/mol (7.4±1.0%) C 56.9±11.3 mmol/mol (7.4±1.0%) | Telehealth | |
| Lee, 2020, South Korea (121) | RCT; PAID; 6 months | 66 (19/20/27) | 72.7% male | Mean age 51.6 years | T2D | Hospital | 10.0 years | Individually, Healthynote app + NFC-enabled glucometer (CareSens N NFC) tracking: bio-parameters, Auto-uploaded to provider-accessible server & Received 1–2 personalised messages/week from endocrinologists and a nurse & phone support vs CAU | I 49.8±15.2 C 50.6±9.8 | I 57.4±8.7 mmol/mol (7.4±0.8%) C 58.5±12.0 mmol/mol (7.5±1.1%) | Telehealth | |
| Lee, 2023, UK (122) | RCT; DDS; 6 months | 124 (61/63) | 0% male | Mean age 31.1 years | T1D | National Health Service sites | 17 years | Individual closed-loop insulin therapy during pregnancy vs standard insulin therapy during pregnancy | I 2.1±0.9 C 2.0±0.8 | NR | Devices | |

| | | | | | | | | | | | | |
|---|---|---|---|---|---|---|---|---|---|---|---|---|
| Leelarathna, 2022, UK (123) | RCT; T1-DDS; 4.8 months | 146 (78/78) | 56% male | Mean age 44 years | T1D | Seven specialist diabetes clinics and one primary care centre | 21.5 years | Individually, in person, 22 weeks intermittently scanned continuous glucose monitoring vs monitoring of blood glucose levels with fingerstick | I 1.96±0.68 C 1.92±0.65 | I 71.6±9.8 mmol/mol (8.7±0.9%) C 69.4±8.7 mmol/mol (8.5±0.8%) | Devices | |
| Lerman, 2009, Mexico (124) | RCT; PAID; 12 months | 59 (24/18/17) | 32% male | Mean age 57.4 years | T2D | Primary care | 12.3 years | (Ia) Diabetes reinforcement educational course (GCR) of group sessions of 5 hours 2 + personal motivation or (Ib) individual monthly contact by telephone with doctor (GRT; Ib) vs C: CAU | Ia 49±29 Ib 45±23 C 51±19 | Ia 67.2±18.6 mmol/mol (8.3±1.7%) Ib 69.4±15.3 mmol/mol (8.5±1.4%) C 78.1±20.8 (9.3±1.9%) | | |
| Li, 2016, China (125) | RCT; DDS; 6 months | 53 (27/26) | 53% male | Mean age 62.5 years | T2D | Hospital | 7 years | Diabetes Conversation Maps-based education of six 1h groups in 4 weeks in person vs traditional education | NR | I 61.7±27.3 mmol/mol (7.8±2.5%) C 60.7±17.5 mmol/mol (7.7±1.6%) | | |
| Li, 2017, Canada (126) | RCT; DDS2; 6 months | 140 (72/68) | 58% male | Mean age 57.4 years | T2D | Tertiary care | 10.8 years | Nurse case manager intervention 1x 60 min in person, individual meeting + follow up calls/emails every 2 weeks vs CAU | NR | NR | | |
| Li 2020, China; Hong Kong (127) | RCT; PAID; 3 months | 225 (117/108) | 50.7% male | Mean age 59.6 years | T2D | Endocrine specialist outpatient clinic; family medicine clinic; three community health centres | 7.3 years | MI & theory of empowerment-based in-person group sessions (4 within 1 month ) by trained nurses and doctors vs Traditional lecture-style health education on DM | I 20.1± 16.9 C 24.4± 22.4 | NR | Education | |
| Li, 2016, Taiwan (128) | RCT; PAID; 7 months | 122 (43/40/39) | 47.5% male | Mean age 58.6 years | T2D | Tertiary hospital centre | 9.5 years | Comparison of Different Models of Structured Self-Monitoring of Blood Glucose, (Ia) six-pair glucose test of pre- and postprandial BG per week; (Ib) three-pair glucose test of pre- and postprandial BG per week; (Ic) seven-point BG testing before and after each meal and at bedtime over a course of 3 days in 1 month | NR | Ia 71.6±14.2 mmol/mol (8.7±1.3%) Ib 70.5±14.2 mmol/mol (8.6±1.3%) Ic 73.8±16.4 mmol/mol(8.9±1.5% ) | Medication | |
| Liu, 2015, China (129) | RCT; DDS2; 6 months | 127 (63/64) | 63% male | Mean age | T2D | Hospital | 10.1 years | Education by peers for 6 months in groups, combined with at least 150 | I 3.2±0.2 C 3.1±0.9 | I 56.3±14.2 mmol/mol(7.3±1.3% ) C 57.4±12.0 | Education | |

| | | | | | | | | | | | | |
|---|---|---|---|---|---|---|---|---|---|---|---|---|
| | | | | 63.3 years | | | | min exercise per week plus once every 2 weeks contact with peer leader vs usual diabetes education | | mmol/mol (7.4±1.1%) | | |
| Lum, 2020, Singapore; USA (130) | RCT; PAID; 3 months | 97 (46/51) | 40.2% male | Mean age 59.5 years | T2D | Healthcare institutions | 11.6 years | FAST tool (structured guide for medication adjustment during fasting): support hotline for tool Qs & Self-monitoring of blood glucose (SMBG) at least 4x/day & endocrinologist-led pre-Ramadan workshop w Ramadan-specific materials vs CAU | NR | NR | telehealth | |
| Lutes, 2018, USA (131) | RCT; DDS-RRD; 12 months | 139 (67/72) | 22% male | Mean age 54 years | T2D | Primary care | 11 years | (COMRADE): individual in-person severity-tailored CBT plus standard care. Assigned to one of two levels of behavioural treatment based on baseline distress and depression, 16 sessions over 12 months, telephone follow-up vs CAU | I 2.68±1.07 C 2.30 ± .89 | I 84.7±23.0 mmol/mol (9.9±2.1%) C 79.2±19.7 mmol/mol (9.4±1.8%) | | |
| Ma, 2022, Hong Kong (132) | RCT; C-DDS 15; 12 months | 97 (214/206) | 50% male | Mean age 59.3 years | T2D | Teaching hospital | 13 years | Group, in person one personalised risk counselling incorporating clinical and genetic risk factors vs CAU | I 39.2 ± 13.8 C 39.8±12.9 | I 70.8±12.0 mmol/mol (8.6±1.1%) C 70.9±10.9 mmol/mol (8.6± 1.0%) | Diverse | Personalised risk |
| MacPhail, 2014, Australia (133) | RCT; DDS; 4 months | 77 (39/38) | NR | Mean age 68 years | T2D | Primary diabetes clinics | 10.1 years | Individually, combined Self-Directed Workbook to introduce HAPA eating interventions: 4 weekly diaries, READY: goals & outcomes, SET: Volition phase, SMART goals, implementation, GO: Action phase, Behaviour rehearsal, performance tracking, reflection AND 2 Motivational interviewing calls vs CAU | NR | NR | Lifestyle | |
| Maghsoudi, 2019, Iran (134) | RCT; DDS; 4 months | 80 (40/40) | 52.5 % male | Mean age 63.07 years | T2D | Clinical or community | 9.5 years | ACT-based in-person group sessions 8×90-min sessions, one session per week. Vs. CAU | I 52.75±13.63 C 54.7±12.11 | NR | | |

| | | | | | | | | | | | | |
|---|---|---|---|---|---|---|---|---|---|---|---|---|
| Maharaj, 2019, South Africa; Nigeria (135) | RCT; PAID; 2.8 months | 46 (23/23) | 0% male | Mean age 41.2 years | T2D | Public health diabetes clinic | 3.08 years | Individually, in person Moderate (40–65% HRR) rebound exercise (mini-trampoline) with handlebars for safety ( 15 min 3 sessions/week (alternate days)) & counselling (15 min) vs counselling only | I 43.0±20.0 C 42.3±21.3 | I 78.1±59.0 mmol/mol (9.3±5.4%) C 73.8±43.7 mmol/mol (8.9±4.0%) | Lifestyle | |
| Malanda, 2016, Netherlands (136) | RCT; PAID; 12 months | 181 (60/59/62) | 66.2% male | Mean age 61.5 years | T2D | General practice | 6 years | Individually, in person, Blood Glucose Monitoring: LifeScan OneTouch Ultra2, 3 pre-prandial + 3 postprandial per day, 2 days/week (Ia) vs Urine Glucose Monitoring: Urispec™ Plus, 1 test after dinner, 2 days/week (Ib) vs CAU (C) | Ia 14.2± 14.7 Ib 6.7± 6.5 C 7.9± 9.0 | Ia 58.5±6.6 mmol/mol (7.5± 0.6%) Ib 60.7±10.9 mmol/mol (7.7± 1.0%) C 57.4±6.6 mmol/mol (7.4± 0.6%) | Devices | |
| Martyn-Nemeth, 2023, USA (137) | RCT; DDS; 1.8 months | 14 (9/5) | 35.7% male | Mean age 29.7 years | T1D | University medical centre | 16.5 years | Sleep-Opt-In: Weekly Didactic Content (sleep importance, procrastination, diabetes & sleep, etc.) (8–10 mins) via REDCap (smartphone/tablet/desktop) & Phone Coaching (5–10 mins) & Wearable Tracker (Fitbit Charge 2®) (for engagement) vs Weekly Health Education Emails (8–10 mins to read) on general wellness (e.g. nutrition, stretching) & 5-min call with a "Healthy Living" coach | I 1.4 ± NR C 2.6± NR | 48.6 ± NR mmol/mol (6.6 ± NR%) C 59.6 ± NR mmol/mol (7.6 ± NR%) | Lifestyle | |
| McAuley, 2020, Australia (138) | RCT; PAID; 6 months | 120 (61/59) | 46.6% male | Mean age 44 years | T1D | Tertiary hospitals | 24.0 years | Individually, in person HCL: MiniMed 670G system (Medtronic), Real-time CGM + automated basal insulin adjustments & manual bolus dosing with carb entry and blood glucose input vs CAU Continued current therapy with MDI or pump & used bolus calculators only (CGM mask), for 26 weeks | I 23.8± 20 C 21.3± 17.1 | I 61.7 ± NR mmol/mol (7.8 ± NR%) C 60.7 ± NR mmol/mol (7.7 ± NR%) | Devices | |

| | | | | | | | | | | | | |
|---|---|---|---|---|---|---|---|---|---|---|---|---|
| McAuley, 2021, Australia (139) | RCT cross-over; PAID-5; 8 months | 30 (30/30) | 36.6% male | Mean age 67 years | T1D | Tertiary hospitals | 35 years | Individually, in person Closed-loop (CL) therapy: MiniMed 670G in Auto Mode with real-time CGM, Automated insulin delivery based on CGM data vs Sensor-Augmented Pump (SAP) therapy: Manual mode only, No algorithm-driven insulin delivery, CGM alerts | NR | NR | Devices | |
| McEwen, 2017, USA(140) | RCT; DDS; 6 months | 157 (83/74) | 55% male | Mean age 53.6 years | T2D | University | 11.5 years | Family-based intervention self-management intervention, 12 weeks, six 2-h group sessions + 3 2h home visits individual + 3 20 in phone calls vs wait list control/CUA | I 15.5±8.5 C 12.3±7.3 | I 84.7±17.5 mmol/mol(9.9±1.6% ) C 84.7±17.5 mmol/mol (9.9±1.6%) | | |
| McMahon, 2012, USA (141) | RCT; PAID; 12 months | 151 (49/51/51) | 94.7% male | Mean age 60.2 years | T2D | Department of Veterans Affairs Boston Healthcare System | NR | Telephone-Based Care Management in-person meeting with nurse or clinical pharmacist on ADA content, Follow-up Biweekly telephone calls to review (Ia) vs Online Care Management: in-person orientation, then log in biweekly to care management platform (Ib) vs Web Training Control: self-directed access (C) educational diabetes websites | Ia 21.8± 21.0 Ib24.5± 20.0 C 29.9± 19.6 | Ia 81.4±10.9 mmol/mol (9.6±1,0%) Ib 84.7±13.1 mmol/mol (9.9 ± 1.2%) C 86.9±15.3 mmol.mol (1.01±1.4%) | telehealth | |
| Misra, 2021, USA (142) | RCT; DDS; 2.8 months | 20 (10/10) | 30% male | Mean age 55 years | T2D | University | NR | 12-weekly 75 min group educational session on diabetes and hypertension self-management program + self-help education + PA guide + 15 min individual follow up vs wait list/CAU | I 1.7±0.8 C 1.8±0.8 | I 59.6±14.2 mmol/mol (7.6±1.3%) C 55.2±14.2 mmol/mol (7.2±1.3%) | | |
| Mitchell, 2023, USA (143) | RCT; DDS; 6 months | 309 (158/151) | 0% male | Mean age 55.4 years | T2D | Urban safety net health system and community health centre | NR | 8 in-person weekly Diabetes Medical Group Visits (DMGVs) (~120 minutes) WIC2" – adapted from Power to Prevent curriculum vs Virtual World (VW)(DMGVs): designed | I 2.72±1.4 C 2.65±1.5 | I 82.5±18.6 mmol/mol (9.7±1.7%) C 87.5±18.6 mmol/mol (10.2±1.7%) | telehealth | |

| | | | | | | | | | | | | |
|---|---|---|---|---|---|---|---|---|---|---|---|---|
| | | | | | | | | avatars, virtual dance, chat, and telehealth activities | | | | |
| Mohn, 2017, Norway (144) | RCT: PAID + DDS; 9 months | 178 (90/88) | 38.2% male | Mean age 37.1 years | T1D | Outpatient clinic | 19.8 years | 7x 2h guided self-determination group training sessions to promote patient autonomy and intrinsic motivation using reflection sheets and advanced professional communication in 14 weeks; vs CAU | I DDS 33.1±16.4 and PAID 36.8±19.3 C DDS 31.9±16.7 and PAID 35.3±18.7 | I 76.0±10.9 mmol/mol (9.1±1.0%) C 78.1±13.1 mmol/mol (9.3±1.2%) | | |
| Munshi, 2013, USA (145) | RCT; PAID; 12 months | 100 (70/30) | 46% male | Mean age 75 years | T2D | Tertiary diabetes centre | 22 years | Evaluation of barriers of self-care individually, 11 phone calls vs CAU with attention control | I 28±17.6 C 24.7±18 | I 78.1±13.1 mmol/mol (9.3±1.2%) C 74.9±8.7 mmol/mol (9.0±0.8%) | | |
| Murray, 2017, UK (146) | RCT; PAID; 12 months | 374 (185/189) | 69% male | Mean age 64.8 years | T2D | General practices; urban, suburban and rural practices | 8 years | HeLP-Diabetes program included interactive, educational, goal setting, behaviour change techniques (SCT,CBT,SDT,TPB) Online Individual (self-directed) 12 months of access to the program vs simple website-education control | I 18.1±17.1 C 19.9±19.9 | I 55.8±14.2 mmol/mol (7.3±1.3%) C 56.8±15.3 mmol/mol (7.4±1.4%) | | |
| Nayak, 2023, USA (147) | RCT; PAID-5; 8 weeks | 32 (16/16) | 40.6% male | Mean age 55.1 years | T2D | Primary care clinics | NR | VBAI (voice-based AI via Alexa) for daily insulin titration using clinician-approved protocol; automated dose adjustments based on FBG vs Standard clinician led insulin; paper logs and daily reminders | I 8.4±3.4 C 6.5±4.9 | I 84.7±17.5 mmol/mol (9.9±1.6%) C 78.1±15.3 mmol/mol (9.3±1.4%) | Diverse | Insulin titration |
| Newby, 2017, Australia (148) | RCT; PAID; 3 months | 106 (49/57) | 29% male | Mean age 46.7 years | T1D/T2D (57%/43%) | Online environment | 16.2 years | 6 automated cartoon-style Web-based individual lessons teaching CBT skills over 10 weeks, min 5 days between lessons + homework vs wait list/CAU | I 45.73±21.45 C 46.87±21.28 | I 62.5±19.6 mmol/mol(7.9±1.8% ) C 60.9±19.9 mmol/mol (7.7±1.8%) | | |
| Ngan, 2023, China (149) | RCT; CDDS-15; 1.4 months | 48 (24/24) | 60.4% male | Mean age 56.9 years | T2D | Outpatient clinic | 7 years | 5-session group-based, in-person ACT-integrated diabetes education over 6 | I 3.43±0.73 C 3.44±0.83 | I 69.7±35.3 mmol/mol (8.5±3.2%) C | | |

| | | | | | | | | | | | | |
|---|---|---|---|---|---|---|---|---|---|---|---|---|
| | | | | | | | | weeks vs (2 h) group-based diabetes education vs CAU | | 76.3±49.2 mmol/mol (9.1±4.5 %) | | |
| Nobis, 2015, Germany (150) | RCT; PAID; 6 months | 260 (130/130) | 37% male | Mean age 51 years | T1D/T2D (45%/55%) | Online | NR | GET.ON M.E.D.: systematic behavioural activation and problem solving based individual online sessions with post-session feedback, 6(+2 optional)x45 mins, 1/week by clinical psychologist supervised coaches vs unguided online psycho-education based on depression guidelines | I 10.24±4.27 10.57±4.52 | I 57.9±15.3 mmol/mol (7.5±1.4%) C 56.9±15.3 mmol/mol (7.4±1.4%) | | |
| O'Brien, 2024, Australia (151) | RCT; PAID; 12 months | 106 (55/51) | 47.1% male | Mean age 53 years | T1D/T2D (46.2%/53.8% ) | Teaching hospital | NR | Mental health IN DiabeteS Optimal Health Program (MINDS OHP): individual telephone/videocall collaborative therapy to improve day-to-day diabetes management; 9 sessions; 8×1-hour weekly individual sessions with a booster 3 months after 8 AND usual care vs CAU | I 37.65±21.85 C 25.10±19.57 | NR | | |
| Odnoletkova, 2016, Belgium (152) | RCT; PAID; 18 months | 574 (287/287) | 61.5% male | Mean age 63.1 years | T2D | Teaching hospital | 7 years | A target-driven tele-coaching intervention COACH on $HbA_{1c}$ self-monitoring of five telephone sessions of 30 (10Ð45) min, delivered interval of 5 weeks (3Ð8) motivational interviewing vs CAU | I 17±17 C 18±19 | I 53.0±12.0 mmol/mol (7.0±1.1%) C 53.0±10.9 mmol/mol (7.0±1.0%) | | |
| Overland, 2014, Australia; USA; Denmark (153) | RCT cross-over; PAID; 9 months | 35 (35/35) | 43% male | Mean age 32.7 years | T1D | Diabetes units | Median 18 years | Intervention: Accu-Chek Mobile strip-free glucose meter (3 months use), then Freestyle Optium (3 months) vs Freestyle Optium glucose meter | NR | NR | Diverse | Insulin titration |
| Pearson, 2018, Australia (154) | RCT; PAID; 2.8 months | 74 (38/36) | 53.7% male | Mean age 59.4 years | T2D | Outpatient clinic | 13.3 years | Mindfulness: self-directed audio CD of guided breath awareness, listened to individually for 8 weeks × 30 min/day. Vs. CAU | I 19.2± 14.7 C 15.2± 14.6 | I 71.7±16.4 mmol/mol (8.7±1.5%) C 63.5±15.7 mmol/mol (8.0±1.4%) | | |
| Petrak, 2015, Germany (155) | RCT; PAID; 15 months | 251 (126/125) | 37.8% male | Mean age | T1D/T2D (51%/49%) | Secondary care | 15.3 years | Diabetes education and cognitive behavioural therapy in groups in | I 47.5±18.4 C 49.1±16.5 | I 78.1±16.3 mmol/mol (9.3±1.5%) C | Diverse | Medication |

| | | | | | | | | | | | | |
|---|---|---|---|---|---|---|---|---|---|---|---|---|
| | | | | 48.5 years | | | | outpatient setting within a 12-weeks × 10 sessions (20 h) manualised semi-structured CBT. Vs. diabetes education and sertraline | | 77.0±15.7 mmol/mol (9.2±1.4%) | | |
| Peyrot, 2008, USA; Canada; Moldova (156) | RCT; DDS-17; 16 weeks | 202 (99/103) | 49% male | Mean age 59.1 years | T2D | Multicentre trial | 10.8 years | Individually, in person, Pramlintide 60–120 ?g before meals and background treatment (insulin glargine (± oral agents); titration guided by glucose targets) vs Placebo injections + CAU | I 2.2±0.8 C 2.1±0.7 | I 69.4±9.8 mmol/mol (8.5±0.9%) C 69.4±9.8 mmol/mol (8.5±0.9%) | Diverse | Medication |
| Peyrot, 2010, USA (157) | RCT; PAID; 6 months | 112 (56/56) | 63.3% male | Mean age 54.5 years | T2D | Multicentre trial | 9.5 years | Addition of mealtime pramlintide vs rapid-acting insulin analogues (RAIAs) to basal insulin therapy | NR | I 66.1±8.7 mmol/mol (8.2±0.8%) C 67.2±8.7 mmol/mol (8.3±0.8%) | Diverse | Medication |
| Piatt, 2018, USA (158) | RCT cluster; DDS-17; 12 months | 221 (119/102) | 36.2% male | Mean age 63.0 years | T1D/T2D (NR) | Primary care practices | NR | Group, in person and telephone diabetes self-management education (DSME) of 6 monthly meetings + Peer leader diabetes self-management support of 6 monthly meetings with peer leader vs CAU | NR | I 59.6 ± NR mmol/mol (7.6 ± NR%) C 58.5 ± NR mmol/mol (7.5 ± NR%) | Education | |
| Pibernik-Okanovic, 2015, Croatia (159) | RCT; PAID; 12 months | 209 (74/66/69) | 45.9% male | Mean age 58 years | T2D | University diabetes clinics | 11 years | Psycho-education: CBT-based psychologist running a 6 weekly x90 min interactive group sessions (Ia) vs 6 weekly x90 min PE education (Ib) vs enhanced CAU (C) | Ia 37.9±19.7 Ib 42.6±20.5 C 39.1±19.6 | Ia 57.4±13.1 mmol/mol (7.4±1.2%) Ib 55.2±12.0 mmol/mol (7.2±1.1%) C 55.2±12.0 mmol/mol (7.2±1.1%) | | |
| Polonsky, 2017, UK (160) | RCT; DDS-17; 5.5 months | 158 (105/53) | 55% male | Mean age 48 years | T1D | Endocrinology practices | 12 years | Individually, in preson daily use of CGM with education on calibration and glucose management for 24 weeks vs CAU Standard self-monitoring of blood glucose | I 1.8±5.7 C 1.7±0.6 | I 70.5±7.7 mmol/mol (8.6±0.7%) C 70.5±6.6 mmol/mol (8.6±0.6%) | Devices | Devices |
| Popelier, 2019, France (161) | RCT; PAID; 3 months | 87 (27/29/32) | NR | Mean age 42.5 years | T1D | Hospital clinic | 18 years | (Ia) a written workshop where type 1 diabetic patients had to write a letter to his own diabetes vs (Ib) a session of living theatre where patients texts focused on life with diabetes or not, are | Ia 39.0±12.9 Ib 35.8±12.4 C 41.7±14.8 | Ia 66.1±15.3 mmol/mol (8.2±1.4%) Ib 70.5±19.7 mmol/mol (8.6±1.8%) C 65.0±16.4 mmol/mol (8.1±1.5%) | | |

| | | | | | | | | | | | | |
|---|---|---|---|---|---|---|---|---|---|---|---|---|
| | | | | | | | | transformed into a script, played by professional actors co-directed by patients and the Director both plus a self-management support program both duration of 4.5d both group sessions vs (C) CAU | | | | |
| Pouwer, 2011, Netherlands (162) | RCT; PAID; 6 months | 223 (116/107) | 42% male | Mean age 53.6 years | T1D/T2D (36/64) | Tertiary diabetes clinics | 17 years | Depression screening via CES-D score of 16 or more followed by diagnostic interview; If diagnosis of a mood and/or an anxiety disorder, the patient, as well as diabetes specialist and general practitioner, received a letter with the DSM-IV diagnosis, and advice regarding treatment options. If no mood/anxiety disorder, the patients received a letter with the outcome of the interview and the advice to contact their physician in case symptoms vs CAU | NR | I 63.9±15.3 mmol/mol (8.0±1.4%) C 63.9±16.4 mmol/mol (8.0±1.5%) | Diverse | PROMS |
| Pranata, 2023, Indonesia (163) | RCT; DDS; 3 months | 172 (86/86) | 20.86% male | Mean age 56 years | T2D | Primary care | 3.5 years | In-person group-based self-management support (follow-ups 2xmonth for 3 months) via brief education, personalised goal-setting, peer support, and follow-ups to improve glycaemic control vs CAU | I 2.46±0.743 C 2.40±0.716 | NR | | |
| Pratley, 2020, USA (164) | RCT; T1-DDS; 6 months | 203 (103/100) | 48.2 % male | Mean age 68 years | T1D | Endocrinology practices | 37.5 years | Individually, in person, CGM using sensor daily, received general diabetes education and alerts for hypoglycaemia vs CAU Standard blood glucose monitoring with 4+ daily fingerstick for 6 months | DDI 1.5±0.4 C 1.5±0.4 | I 59.6±10.9 mmol/mol (7.6±1.0%) C 58.5±9.8 mmol/mol (7.5±0.9%) | Devices | |
| Presley, 2020, USA (165) | RCT; DDS; 6 months | 120 (70/50) | 28.9% male | Mean age 54.9 years | T2D | Public-safety net system; community outreach | 8.7 years | Community-based diabetes self-management education (DSME); group initial face-to-face visit + 6 months individual mHealth- | I 2.7±1.2 C 2.6±1.1 | I 86.9±18.6 mmol/mol (10.1±1.7%) C 83.6±18.6 mmol/mol (9.8±1.7%) | | |

| | | | | | | | | | | | | |
|---|---|---|---|---|---|---|---|---|---|---|---|---|
| | | | | | | | | enhanced peer support + 12 weekly phone calls, then 3 monthly calls from CHW, who used a novel web application to communicate with participants' healthcare teams. vs DSME alone | | | | |
| Pyatak, 2018, USA (166) | RCT; PAID-5; 6 months | 81 (41/40) | 37 % male | Mean age 22.6 years | T1D/T2D (25%/75%) | Paediatric and young adult diabetes clinic | 9.7 years | Individually, in person manualised and individualised Occupational Therapy led program (REAL Diabetes): biweekly sessions ($^3$10 hrs), flexible content modules addressing self-care, advocacy, social support, and emotional wellbeing vs Attention control: educational materials + scripted biweekly phone calls | I 9.7±5.2 , C9.4±6.2 | I 96.7±21.9 mmol/mol (11.0±2.0%) C 91.3±18.6 mmol/mol (10.5±1.7%) | Education | |
| Pylov, 2023, Sweden (167) | RCT cross-over; PAID; 18 months | 161 (161/161) | 56.1% male | Mean age 44.6 years | T1D | University | 22.1 years | Individually, in person CGM for 26 weeks vs CAU for 26 weeks | NR | I 71.7±8.7 mmol/mol (8.7±0.8%) C 71.6±9.8 mmol/mol (8.7±0.9%) | Devices | |
| Quinn, 2011, USA (168) | Cluster RCT; DDS; 12 months | 192 (80/62/28/22) | 49.2% male | Mean age 52.8 years | T2D | Primary care practice | 8.2 years | Mobile application coaching and patient/provider web portals: individual patient-coaching system & provider clinical decision-support (CPDS; Ia), real-time feedback over 1 year vs , coach only (Ib), or coach and primary care provider portal (Ic), CAU (C) | Ia 2.6±0.9 Ib 2.7±0.9 Ic 2.8±0.7 C 2.4±0.9 | Ia 84.7±23.0 mmol/mol (9.9±2.1%) Ib 78.1±19.7 mmol/mol (9.3±1.8%) Ic 74.9±19.7 mmol/mol (9.0±1.8%) C 77.0±18.6 mmol/mol (9.2±1.7%) | | |
| Ramallo-Farina, 2021, Spain (169) | Cluster RCT; DDS2; 24 months | 2334 (537/557/654/586) | 48.1% male | Mean age 55.7 years | T2D | Primary care centres | NR | Patient Training Intervention (Ia), Combined Intervention (Ib); 8 in-person group education + online monitoring & feedback + SMS support Professional Training Intervention (PFI); 2 group sessions for knowledge transfer and decision support (Ic) vs CAU (C) | I 2.8 (95% CI 2.6–2.8) Ib 2.5 (95% CI 2.3–2.6) Ic 2.7 (95% CI 2.6–2.8) C 2.6 (95% CI 2.5–2.6) | NR | | |

| | | | | | | | | | | | |
|---|---|---|---|---|---|---|---|---|---|---|---|
| Reaney, 2013, Spain (170) | RCT; PAID; 7.5 months | 681 (330/351) | 53.3% male | Mean age 62 years | T2D | Healthcare settings; multicentre | 8 years | Conversation Map[a]-education tools-based in-person group education sessions (2Ð3 hours each),1- to 2-week intervals over 6 weeks by diabetes educator vs CAU | I Germany: 19± 15.7 Spain: 35± 22.5 C Germany: 18.7± 19.4 Spain: 35.4± 22.5 | I overall 55.2±12.2 mmol/mol (7.2±1.1%) C overall 53.0±10.9 mmol/mol (7.0±1.0%) | | |
| Reddy, 2018, UK (171) | RCT; PAID20; 1.8 months | 40 (20/20) | 60% male | Mean age 49.5 years | T1D | Diabetes specialist site | 30 years | Individually, in person, 8 weeks, CGM (Dexcom G5) vs Fash glucose monitoring(Abbott Freestyle Libre) | I 31.0±23.7 C 19.0±23.7 | I 57.4±9.8 mmol/mol (7.4±0.9%) C 55.2±13.1 mmol/mol (7.2±1.2%) | Devices | |
| Rees, 2013, Australia (172) | RCT; DDS-17; 3 months | 25 (15/10) | 58% male | Mean age 67 years | T2D | Retinal clinics | 21.0 years | Individually, Receive visual feedback of their own retinal images + info on retina vs CAU | NR | I 77.0±17.5 mmol/mol (9.2±1.6%) C 72.7±9.8 mmol/mol (8.8±0.9%) | Diverse | Personalised risk |
| Rees, 2017, Australia (173) | RCT; DDS; 6 months | 40 (21/19) | 67.5% male | Mean age 59.9 years | T2D | Retinal clinics | 21.0 years | Problem solving therapy (PST-D) intervention of 8 individual sessions (45Ð60 min), and delivered via telephone or in person vs CAU | I 2.9±0.8 C 2.7±0.6 | I 65.0±13.1 mmol/mol (8.1±1.2%) C 66.1±21.9 mmol/mol (8.2±2.0%) | | |
| Rezaeemanesh, 2020, Iran (174) | RCT; DDS; 3 months | 100 (50/50) | 47% male | Mean age 54.25 years | T2D | Diabetes clinic | 10.5 years | In-person group 5×1h educational sessions; educational booklets, posters, and educational, pamphlets vs CAU | I 76.70±16.17 C 71.40±15.74 | NR | | |
| Rilstone, 2024, UK (175) | RCT cross over; DDS17; 2.4 months | 24 (24/24) | 33% male | Mean age 35.2 years | T1D | Outpatient clinics | 13.9 years | Individually, in person, 40 days, unblinded CGM with ULS alert on vs unblinded CGM with ULS alert off | I 1.8±0.7 C 1.8±0.7 | I 58.5±35.0 mmol/mol (7.5±3.2%) C 58.5±35.0 mmol/mol (7.5±3.2%) | Devices | |
| Roddy, 2023, USA (176) | RCT; PAID; 15 months | 329 (164/165) | 51.4% male | Mean age 57.0 years | T2D | UMC primary care clinics | 11.5 years | Family/friend Activation to Motivate Self-care (FAMS), monthly self-care support intervention delivered individually via mobile phones over 9 months vs enhanced CAU | I 38.8±26.9 C 38.1±24.5 | I 70.5±19.7 mmol/mol (8.6±1.8%) C 69.4±17.5 mmol/mol (8.5±1.6%) | Education | |
| Rondags, 2016, Netherlands (177) | Cluster RCT; PAID; 6 months | 137 (71/66) | 54% male | Mean age 52 years | T1D/T2D (89.1%/10.9%) | Outpatient clinic | 26 years | HypoAware: a brief, partly web-based psychoeducational group intervention for problematic hypoglycaemia, 3×2.5 h over 4 weeks, with 2 user- | NR | I 60.7±10.9 mmol/mol (7.7±1.0%) C 60.7±12.0 mmol/mol (7.7±1.1%) | | |

| | | | | | | | | | | | | |
|---|---|---|---|---|---|---|---|---|---|---|---|---|
| | | | | | | | | friendly online modules between meetings vs CAU | | | | |
| Rondhianto, 2018, Indonesia (178) | RCT; DDS; 1.4 months | 120 (60/60) | 36.67% male | Mean age 57.60 years | T2D | Community health centre | 3.8 years | Individual home visit & group-based diabetes self-management education, based on the Health Belief Model (HBM) over six sessions (~120 mins) vs CAU | I 39. 33±6.87 C 38.67±5.28 | NR | | |
| Rosales, 2021, Mexico (179) | Cluster RCT; PAID; 12 months | 464 (239/225) | 15% male | Mean age 60 years | T2D | Clinics | NR | In-person group 2-h participatory education, workshop-style sessions delivered by health professionals vs CAU | I 22.6±15.6 C 22.2±16.3 | NR | | |
| Rosenbek Minet, 2011, Denmark (180) | RCT; PAID; 24 months | 349 (173/176) | 50% male | Mean age 56.4 years | T1D/T2D (22%/78%) | Hospital clinic | 4.7 years | Motivational and goal oriented with behaviour change focus. Individually delivered face to face by multi-disciplinary team. 5 _ 35 min sessions over 12 months vs CAU | I 20±17.7 C 19.6±16.3 | I 53.0±13.1 mmol/mol (7.0±1.2%) C 53.0±13.1 mmol/mol (7.0±1.2%) | | |
| Rosland, 2022, USA (181) | RCT; PAID; 12 months | 239 (123/116) | 96.7% male | Mean age 60 years | T2D | Hospital-based; community-based | 12.5 years | Caring Others Increasing Engagement in Patient Aligned Care Teams (CO-IMPACT), one dyad coaching session (1–1.5h) then 12 months of biweekly dyadic automated telephone. Vs. CAU | I 5.6±5.2 C 6.2±5.2 | I 68.3±16.4 mmol/mol (8.4±1.5%) C 70.5±19.7 mmol/mol (8.6±1.8%) | | |
| Ruiz de Adana, 2019, Spain (182) | RCT; DDS; 6 months | 330 (167/163) | 55.2% male | Mean age 35.01 years | T1D | Diabetes units | NR | One telemedicine visit (via Diabetic platform) replaced the mid-point in-person visit; patients uploaded glucometer data and received insulin advice vs CAU | NR | I 53.0±6.6 mmol/mol (7.0±0.6%) C 54.1±7.7 mmol/mol (7.1±0.7%) | telehealth | |
| Russell-Jones, 2019, UK(183) | RCT; DDS; 24 weeks | 631 (314/317) | 50.2% male | Mean age 63.3 years | T2D | Outpatient participants research centre | 12.9 years | Self-managed titration of insulin glargine 300 U/ml (Gla-300) to target fasting glucose (4.4–7.2 mmol/l) vs Physician-managed titration of Gla-300 (same target) | I 2.04± 0.89 C 2.06±0.99 | I 68.3±9.7 mmol/mol (8.4±0.9%) C 68.6±10.1 mmol/mol (8.4±0.9%) | Diverse | Insulin titration |
| Rygg, 2012, Norway (184) | RCT; PAID; 12 months | 146 (73/73) | 55% male | Mean age 66 years | T2D | General practice | 5.8 years | Group based diabetes self-management education (DSME), in-person 15 h over | I 22.1±16.4 C 18.2±16.2 | I 54.1±15.3 mmol/mol (7.1±1.4%) C | | |

| | | | | | | | | | | | | |
|---|---|---|---|---|---|---|---|---|---|---|---|---|
| | | | | | | | | 3 sessions (1/2 weeks each session) vs CAU/wait list | | 51.9±14.2 mmol/mol (6.9±1.3%) | | |
| Safford, 2015, USA (185) | Cluster RCT; DDS; 15 months | 424 (198/226) | 24.7% male | Mean age 60.2 years | T1D/T2D (NR) | Primary care | NR | Peer-coached weekly telephone sessions for 2 months, then at monthly (8 months) AND brief education vs brief education | I 2.1±1.2 C 2.1±1.2 | I 63.9±23.0 mmol/mol (8.0±2.1%) C 62.8±20.8 mmol/mol (7.9±1.9%) | Education | |
| Sato, 2020, Japan (186) | RCT; PAID; 12 months | 30 (15/15) | 57.7% male | Mean age 52.2 years | T2D | Clinic | Newly diagnosed | Individually, in person FGM device (Freestyle Libre) for 2–4 weeks at treatment start, with education on diet and exercise; could check glucose frequently vs CAU | NR | I 77.0±25.1 mmol/mol (9.2±2.3%) C 67.2±21.9 mmol/mol (8.3±2.0%) | Devices | |
| Schmitt, 2022, Germany (187) | RCT; PAID; 12 months | 260 (131/129) | 45.3% male | Mean age 45.0 years | T1D/T2D (64%/36%) | Specialised diabetes centre | 14.4 years | Stepped-care step 1: Five group sessions (90 min each) of diabetes-specific CBT group step 2: 6–12 sessions of single CBT for depression (50 min each) via telephone step 3: outpatient depression treatment vs CAU | I 39.9±18.7 C 39.8± 18.4 | I 71.6±16.4 mmol/mol (8.7±1.5%) C 72.7±16.4 mmol/mol (8.8±1.5%) | | |
| Schneider-Utaka, 2023, UK; Austria (188) | RCT cross-over; T1D-DDS; 7 months | 37 (37/37) | 57% male | Mean age 67 years | T1D | Diabetes centres | 39 years | Individually, in person, Use of CamAPS FX hybrid closed-loop insulin delivery system (CGM + algorithm-driven pump) vs Sensor-augmented pump (SAP) therapy without automation | NR | NR | Devices | |
| Schroevers, 2015, Netherlands (189) | RCT;PAID;2.8 months | 24 (12/12) | 58% male | Mean age 55 years | T1D/T2D (46%/54%) | Outpatient clinic | 18.55 years | 8-week MBCT group protocol, eight weekly individual sessions of 60 min. asked to practice every day >30 min) the guided mindfulness exercises on CD/ informal exercises such as mindful eating. Vs. wait list /CAU | I 41.6±15.2 C 39.0±16.8 | I 66.1±13.1 mmol/mol (8.2±1.2%) C 65.0±16.4 mmol/mol (8.1±1.5%) | | |
| Secher, 2021, Denmark (190) | RCT; PAID20; 6 months | 170 (41/48/39/42) | 63% male | Mean age 47 years | T1D | Five diabetes clinics | 18 years | Individually, in person 26 weeks automated bolus calculation ABC (Ia) vs intermittently scanned continuous glucose monitoring (isCGM; Ib) or ABC+isCGM(Ic) vs CAU (C) | Ia 23±17.8 Ib21±18.5 Ic 15±14 C 25±11.1 | Ia 63.9±7.7 mmol/mol (8.0±0.7%) Ib 62.8±7.7 mmol/mol (7.9±0.7%) Ic 68.3±5.5 mmol/mol (8.4±0.5%) C | Devices | |

| | | | | | | | | | | | | |
|---|---|---|---|---|---|---|---|---|---|---|---|---|
| | | | | | | | | | | 66.1±5.5 mmol/mol (8.2±0.5%) | | |
| Seligman, 2018, USA (191) | RCT; PAID; 6 months | 568 (285/283) | 32.4% male | Mean age 54.8 years | T2D | Feeding America Food banks | NR | 6-month food bank-based support: diabetes-appropriate food (11 packages), Diabetes self-management education (2 core classes + optional drop-ins), referrals, and glucose monitoring. Delivered at pantries by trained staff/volunteers vs CAU and wait list | NR | I 83.1±19.6 mmol/mol (9.8±1.8%) C 83.0±19.2 mmol/mol (9.7±1.8%) | Lifestyle | |
| Shibayama, 2007, Japan (192) | RCT; PAID; 12 months | 134 (67/67) | 65.2% male | Mean age 61.5 years | T2D | Outpatient hospital | 11.15 years | One-on-one monthly visits by expect nurse in lifestyle counselling, management etc. 8-76 min (median, 25 min) vs CAU | I 38±17.9 C 35±18.7 | I 56.3±8.7 mmol/mol (7.3±0.8%) C 57.4±7.7 mmol/mol (7.4±0.7%) | | |
| Shukla, 2021, India (193) | RCT; DDS; 6 months | 32 (16/16) | 46.9% male | Mean age 23.8 years | T1D | Outpatient department of a tertiary care hospital | 12.7 years | Training program of breathing exercises and mindful meditation (MM), advised practice MM for min of 20 min daily, weekly telephonic calls and in-person monthly follow-ups. Vs. CAU | I 1.6±0.3 C 1.6±0.3 | I 76.0±16.4 mmol/mol (9.1±1.5%) C 78.1±25.1 mmol/mol (9.3±2.3%) | | |
| Siaw, 2017, Singapore (194) | RCT; PAID; 6 months | 411 (214 /197) | 56% male | Mean age 59.6 years | T1D | Outpatient healthcare institutions | 13.1 years | Multidisciplinary collaborative care: patients received regular follow-ups with pharmacists (every 4–6 weeks), with optional nurse/dietitian support as needed. Pharmacists used a structured protocol including motivational interviewing, medication review, insulin titration (SIGN), and patient education. Face-to-face or phone-based vs CAU | I 28.6±19.9 C 26.4±20.8 | I 70.5±16.4 mmol/mol (8.6±1.5%) C 69.4±15.3 mmol/mol (8.5±1.4%) | Diverse | Complex healthcare |
| Sigurdardottir, 2009, Iceland (195) | RCT; PAID; 6 months | 58 (30/28) | 67.9% male | Mean age 61 years | T2D | Hospital-based; primary healthcare; private | 8.5 years | A tailored care approach, one individual face-to-face session (1–2h) at baseline and telephone calls (15–20 mins) once a week for the 5 following weeks by nurses. Vs. CAU | I 24.1±14.5 C 15.8±14.5 | I 64.9±10.4 mmol/mol (8.1±1.0%) C 62.6±9.7 mmol/mol (7.9±0.9%) | | |

| | | | | | | | | | | | | |
|---|---|---|---|---|---|---|---|---|---|---|---|---|
| Siminerio, 2013, USA (196) | RCT; PAID; 6 months | 141 (38/36/35/32) | 44.0%male | Mean age 61 years | T2D | Primary care | NR | All participants first received one-on-one Diabetes Self-Management Support (DSMS) from Certified Diabetes educators (CDE). Then randomised to DSMS from either: Educators (Ia): Monthly follow-up calls (15–20 min) by CDEs; Peers: Trained peers (Ib) made monthly support calls (25–30 min); Practice Staff (Ic): Licensed nurses/MAs made brief calls (5–10 min); CAU (C): 1–2 standard follow-up calls from CDEs. Calls focused on patient-defined behavioural goals using empowerment techniques. | NR | Ia 67.2±19.7 mmol/mol (8.3±1.8%) Ib 70.5±26.2 mmol/mol (8.6±2.4%) Ic 74.9±23.0 mmol/mol (9.0±2.1%) C 71.6±20.8 mmol/mol (8.7±1.9%) | Education | |
| Simmons, 2015, UK (197) | RCT; DDS-4; 12 months | 1299 (330/325/322/322) | 60.4% male | Mean age 65.1 years | T2D | Rural communities | 6.5 years | 8–12 months Individual Peer Support (Ia): trained peer support facilitator for regular one-on-one meetings (~1 hour). Group Peer Support (Ib): small, locally organised group sessions (~1.5 hours) led by trained facilitators. Combined Peer Support (Ic)t: Participants received both 1:1 and group peer support from trained facilitators over. vs CAU (C) | Ia 6.53±4.2 Ib 6.27 ± 3.2 Ic 6.71 ± 7.3 C 6.61 ± 4.3 | Ia 57.4±14.2 mmol/mol (7.4±1.3%) Ib 58.5±14.2 mmol/mol (7.5±1.3%) Ic 56.3±14.2 mmol/mol (7.3±1.3%) C 56.3±14.2 mmol/mol (7.3±1.3%) | Peer support | |
| Simonson, 2018, USA (198) | RCT; PAID; 3 years | 38 (19/19) | 39% male | Mean age 51.6 years | T2D | Medical school | 10.4 years | Roux-en-Y gastric bypass (RYGB) Intensive multidisciplinary medical diabetes- and weight-management program (IMWM): Two-hour group sessions conducted weekly during a 12-week initiation phase; patients receive individual medication adjustments, participation in supervised group exercise and didactic | NR | I 66.6±15.5 mmol/mol (8.2±1.4%) C 72.5±11.1 mmol/mol (8.8±1.0%) | Diverse | Surgery |

| | | | | | | | | | | | | | |
|---|---|---|---|---|---|---|---|---|---|---|---|---|---|
| | | | | | | | | sessions. Moreover, weekly medication adjustments; structured modified dietary intervention with a hypocaloric (1500–1800 kcal) diet; up to 300 min/week of graded, balanced, and individualised exercise with emphasis on strength training; cognitive behavioural therapy; and group education. | | | | |
| Simson, 2008, Germany (199) | RCT; PAID; end of treatment (discharge); mean 1.7 months | 30 (15/15) | 57% male | Mean age 61 years | T1D/T2D (20%/80%) | Hospital inpatients | NR | Psychotherapy with mood focus. Individual face to face delivered by psych specialist; 5 _ 30 min sessions over 6 weeks vs CAU | I 34.6±9.4 C 30.9±17.2 | I 61.7±16.4 mmol/mol (7.8±1.5%) C 71.6±19.7 mmol/mol (8.7±1.8%) | | |
| Skelly, 2009, USA (200) | RCT; PAID; 9 months | 180 (60/60/60) | 0% male | Mean age 67 years | T2D | Healthcare centres; health department clinics; primary care | 12 years | Symptom-focused intervention: Ia) 4 in-person individual 60-minute bimonthly nurse at-home visits Ib) AND telephone calls at approximately 2–3 week intervals (15mins) vs attention control © | Ia 2.05± 0.56 Ib 2.31±0.75 C 2.44± 1.05 | I 67.2±17.9 mmol/mol (8.3±1.6%) Ib 68.7±17.8 mmol/mol (8.4±1.6%) C 65.1±17.6 mmol/mol (8.1±1.6%) | Education | |
| Snoek, 2008, Netherlands (201) | RCT; PAID; 12 months | 86(45/41) | 41.8% male | Mean age 37.8 years | T1D | Outpatient clinics | 18.3 years | Cognitive behavioural therapy (CBT) programme for adult T1D patients with prolonged self-care difficulties in six weekly in-person small group sessions. Facilitated by psychologist and diabetes nurse educator. vs six weekly blood glucose awareness training group sessions by experienced diabetes nurse educator and clinical psychologist. | I 44.4±22.4 C 49.0±17.2 | I 72.7±14.2 mmol/mol (8.8±1.3%) C 76.0±12.0 mmol/mol (9.1±1.1%) | | |
| Spencer, 2018, USA (202) | RCT; DDS; 18 months | 222 (89/60/73) | 39.2% male | Mean age 48.9 years | T2D | Community Health; Social Services Centre | NR | CHW-led, 6-month in-person group DSME AND 1) 12 months of monthly individual telephone outreach (CHW-only) OR 2) 12 months of weekly in-person group sessions by | I 2.2±1.1 C NR | | Education | |

| | | | | | | | | | | | |
|---|---|---|---|---|---|---|---|---|---|---|---|
| | | | | | | | | peer leaders with telephone outreach vs enhanced CAU | | | |
| Spencer, 2011, USA (203) | RCT; PAID; 6 months | 164 (72/92) | 29% male | Mean age 53 years | T2D | Community Health Centre | NR | Group diabetes education classes with provided by peer 11×2u, home visits 2 per month by peer 60 min in length to address participants' specific self-management goals, and 1 clinic visit with peer visit with the participant and his or her primary care provider and phone call vs wait list | I 23.8±10 C 25.9±10 | I 70.5±12.0 mmol/mol (8.6±1.1%) C 69.4±12.0 mmol/mol (8.5±1.1%) | | |
| Sperl-Hillen, 2013, USA (204) | RCT; PAID; 12 months | 623 (246/243/134) | 51% male | Mean age 62 years | T2D | Health clinics | 11.7 years | (Ia) Individual Three 1-hour personalised sessions over 3 months with certified educators using the American Association of Diabetes Education (AADE) framework. The AADE focuses on 7 self-care behaviour (Healthy eating, monitoring blood-glucose; taking medication, problem solving; risk reduction, healthy coping; being active v (Ib): Four 2-hour group education sessions vs CAU (C) | Ia: 29.81 ± NR Ib: 29.62 ± NR C: 30.52 ± NR | Ia 65.1 ± NR mmol/mol (8.1 ± NR%) Ib 64.7 ± NR mmol/mol (8.1 ± NR%) | Education | |
| Sturt, 2008, UK (205) | RCT; PAID; 6 months | 245 (114/131) | 61% male | Mean age 62 years | T2D | Primary care practices | NR | Individually, combined, Diabetes Manual Intervention underpinned by self-efficacy theory to develop confidence for self-care and reduce diabetes-distress, includes 15 minute face-to-face introduction to the 12-week Diabetes manual workbook, relaxation/audio tapes and 3 times nurse telephone support vs CAU (delayed intervention) | I 21±15 C 21±15 | I 73.8±15.3 mmol/mol (8.9±1.4%) C 71.6±15.3 mmol/mol (8.7±1.4%) | Education | |
| Sunuwar, 2023, Nepal(206) | RCT; PAID; 6 months | 156 (78/78) | 51.2% male | Mean age | T2D | Outpatient department | NR | Dietitian-led nutrition counselling + individualised | I 43.03±19.72 | I 64.2±12.1 mmol/mol | Lifestyle | |

| | | | | | | | | | | | | |
|---|---|---|---|---|---|---|---|---|---|---|---|---|
| | | | | 48.5 years | | | | diet plan and monthly follow-up vs CAI | C 49.41±17.25 | (8.0±1.1%) C 64.4±11.8 mmol/mol (8.0±1.1%) | | |
| Swoboda, 2017, USA (207) | RCT; DDS; 3.7 months | 53 (37/16) | 31.4% male | Mean age 56 years | T2D | Community organizations (e.g. libraries, medical facilities) | 7.5 years | Goal-setting by decision coaching & MI (either SG or MG) on health outcomes. Baseline in-person session, 7 biweekly calls (every 2 weeks for 14) vs same schedule attention control | I 2.29±0.66 C 2.40±1.28 | NR | | |
| Tang, 2015, USA (208) | RCT; DDS; 15 months | 106 (54/52) | 33% male | Mean age 56.3 years | T2D | Community centres | 9.2 years | 3-month group DSME (12 weekly sessions (90 min)), 12 months individual in-person peer-led self-management support (often as needed) + follow-up phone calls vs 3-month group DSME alone | NR | I 61.8±23.0 mmol/mol (7.8±2.1%) C 63.9±17.6 mmol/mol (8.0±1.6%) | | |
| Tang, 2022, Canada (209) | RCT; DDS; 12 months | 196 (98/98) | 50% male | Mean age 59.5 years | T2D | Specialty care settings | 12 years | Peer-Led, Empowerment-based, Approach, to Self-management Efforts in Diabetes (PLEASED) intervention, starts with home visit, with individually weekly telephone contacts from a peer leader in the first 3 months followed by bi-weekly telephone contacts in the last 9 months vs CAU | I 2.26±0.85 C 2.49±0.98 | I 64.3±19.9 mmol/mol (8.0±1.8%) C 65.7±17.6 mmol/mol (8.2±1.6%) | | |
| Tang, 2012, USA (210) | RCT; PAID; 12 months | 415 (202/213) | 60% male | Mean age 53.7 years | T2D | Not-for-profit healthcare organisation | NR | Comparison of online disease management of diabetes "Engaging and Motivating Patients Online With Enhanced Resources for Diabetes" (EMPOWER-D) consisting of online diabetes management consisted of glucometer uploads, dashboard, logs, messaging, nurse care manager, dietitian, educational videos, protocol-based med changes vs CAU | NR | I 77.5 ± NR mmol/mol (9.2 ±NR%) C 77.9 ± NR mmol/mol (9.3 ± NR%) | Education | |

| | | | | | | | | | | | | |
|---|---|---|---|---|---|---|---|---|---|---|---|---|
| Tang, 2014, USA (211) | RCT; DDS; 18 months | 116 (60/56) | 41.4% male | Mean age 49.3 years | T2D | Federally qualified health centre | 6.6 years | DMSE of eleven 2-h culturally tailored interactive group self-management classes, two home visits (60 min in length) per month to help patients set and follow up on specific self-care goals (action plans), and one visit with the participant and his or her primary care provider. Comparison of a peer leader (PL) + ongoing emotional and behavioural support by PLs through group-based sessions and follow-up telephone contacts. Group-based DSMS sessions were offered weekly, with participants encouraged to attend sessions as often as needed vs DSME + monthly telephone outreach initiated by a CHW over a period of 12 months | NR | I 66.1±24.0 mmol/mol (8.2±2.2%) C 61.8±18.6 mmol/mol (7.8±1.7%) | Education | |
| The ISCHIA Study Group, 2023, Japan (212) | RCT cross over; PAID-20; 2.8 months | 102 (51/51) | 47.3 % male | Mean age 51.4 years | T1D | 17 research centres | 16 years | Individually, in person 84 days intermittent-scanning continuous glucose monitoring (isCGM) device with structured education vs self-monitoring of blood glucose (SMBG) | I 31.9±18.3 C 33.4±19.2 | I 56.3±7.7 mmol/mol (7.3±0.7%) C 56.3±7.7 mmol/mol (7.3±0.7%) | Devices | |
| Tovilla-Zárate, 2019, Mexico (213) | RCT; PAID-5; 8 weeks | 21 (12/9) | 28.6% male | Mean age 48.5 years | T2D | Diabetes clinic | 11.6 years | Vortioxetine (10 mg/day) vs sertraline (75 mg/day) for 8 weeks | I 6.75±5.32 C 6.00±4.06 | I I 77.5 ± NR mmol/mol (9.2 ±NR%) C 77.9 ± NR mmol/mol (9.3 ± NR%) (60% male ± NR%) C I 66.1±24.0 mmol/mol (8.2±2.2%) C 61.8±18.6 mmol/mol (7.8±1.7%) (41.4% male ± 6.6 years%) | Diverse | Medication |

| | | | | | | | | | | | | |
|---|---|---|---|---|---|---|---|---|---|---|---|---|
| Tovote, 2015, Netherlands (214) | RCT; PAID; 9 months | 94(31/32/31) | 51% male | Mean age 53.1 years | T1D/T2D (39%/61%) | Outpatient clinic | 16.6 years | mindfulness based cognitive therapy (Ia): individual in person formal meditation, yoga exercises, and informal daily mindfulness practices 8x 45 minutes with 30 minutes of homework weekly by trained therapists ; cognitive behavioural therapy (Ib): individual in-person behavioural activation and cognitive restructuring 8x 45 minutes with 30 minutes of homework weekly by trained therapists. vs (C) waiting list | Ia 38.3±20.9 Ib 42.0±22.3 C 35.5±21.5 | Ia 63.9±9.8 mmol/mol (8.0±0.9%) Ib 67.2±15.3 mmol/mol (8.3±1.4%) C NR | | |
| Trevisan, 2020, Brazil (215) | RCT; DDS; 3.5 months | 90(45/45) | 29.2% male | Mean age 61.4 years | T2D | Primary care | 7.2 months | Implementation intention theory based action planning form filled out with the help of the main researcher (three action plans covering when, where and how they intended to take their oral anti-diabetic medication) four face-to-face meetings, took place at baseline (T0), 15 days later (T1), 60 days later (T3) and on the 105th day of monitoring (T5), and two phone reinforcements, at T2 (T0 + 30 days) and T4 (T0 + 75 days) vs CAU | I 2.4±1.0 C 2.2±0.8 | I 56.3±8.7 mmol/mol (7.3±0.8%) C 55.2±10.9 mmol/mol (7.2±1.0%) | | |
| Trief, 2007, USA (216) | Cluster RCT; DDS; 12 months | 1665 (821/844) | 37.2% male | Mean age 71 years | T2D | University medical centre | NR | Home telemedicine unit (HTU) provided Videoconference with nurse and care managers, remote glucose and blood pressure monitoring, web-based access to clinical data, and access to educational material from ADA vs CAU | NR | NR | telehealth | |

| | | | | | | | | | | | | |
|---|---|---|---|---|---|---|---|---|---|---|---|---|
| Trief, 2016, USA; Canada (217) | RCT; DDS; 12 months | 280 (82/:94/104) | 61.6% male | Mean age 56.8 years | T2D | Community | 12.4 years | Two telephone sessions (~ 75 min) of diabetes education based on social learning theory, occurred weekly for 12 weeks. Ia; 10 additional couple calls (average 57 min/call) based on Interdependence Theory in which partners were actively involved + homework, including 2 relationship focused calls. vs Ib:10 additional individual calls (average 50 min/call) which was identical to Ia except partners were not involved and the two relationship-focused calls addressed individual problem solving. vs C: no further intervention after two calls . | Ia 2.4±0.8 Ib 2.3±1.1 C 2.2±0.9 | I 73.8±14.2 mmol/mol (8.9±1.3%) Ib 78.1±18.6 mmol/mol (9.3±1.7%) C 76.0±17.5 mmol/mol (9.1±1.6%) | telehealth | |
| Uduku, 2023, UK (218) | RCT; PAID20; 2.8 months | 35 (17/18) | 56.7% male | Mean age 43 years | T1D | NHS hospitals | 26 years | Individually, in person, 12 weeks Real-time Continuous Glucose Monitoring vs CAU | I 27.0±20.74 C 26.5±20.7 | I 25.7±27.3 mmol/mol (4.5±2.5%) C 23.5±28.4 mmol/mol (4.3±2.6%) | Devices | |
| van Bastelaar, 2011, Netherlands (219) | RCT; PAID; 3 months | 255 (125/130) | 39% male | Mean age 50 years | T1D/T2D (45%/55%) | Online environment | T1D: 21; T2D: 9 years | Individual Web-based CBT (Cognitive Behavioural Therapy) with written and spoken information and videos, 8 consecutive lessons with homework facilitated by certified health psychologist. Vs. waiting list, CAU | I 42±19 C 38±19 | I 57.4±17.5 mmol/mol (7.4±1.6%) C 56.3±17.5 mmol/mol (7.3±1.6%) | | |
| Van der Wulp, 2012, Netherlands (220) | RCT; PAID; 6 months | 133 (68/65) | 54.6% male | Mean age 61 years | T2D | Primary care | 0.75 years | Peer led self-management coaching programme on self-efficacy based on Bandura's Social Cognitive Theory, 3x 1-hour individual home visit by peer in three months + phone contact within 2 weeks after each home visit vs CAU | I 16.65±18.95 ] C 14.48±15.50 | NR | Peer support | |
| van Son, 2014, Netherlands (221) | RCT; PAID; 8 months | 139 (70/69) | 50.3% male | Mean age | T1D/T2D (30%/70%) | Outpatient diabetes clinics | NR | In-person mindfulness-based cognitive therapy (MBCT) consisting of 8x 2- | I 35.5± 17.8 C 36.6± 18.9 | I 58.5±13.1 mmol/mol (7.5±1.2%) C | | |

| | | | | | | | | | | | | |
|---|---|---|---|---|---|---|---|---|---|---|---|---|
| | | | | 56.5 years | | | | hour group sessions weekly . Three months after the end of the intervention a two-hour booster session. Vs. waitlist, CAU | | 59.6±13.1 mmol/mol (7.6±1.2%) | | |
| van Beers, 2016, Netherlands(222) | RCT cross-over; PAID-5; 16 weeks | 52 (52/52) | 54% male | Mean age 48.6 years | T1D | Outpatient clinics | 30.5 years | Individually, in person, CGM using Paradigm Veo + Enlite sensor<br>vs CAU SMBG using usual home meters + masked CGM | NR | NR | Devices | |
| van den Donk, 2010, Netherlands (223) | Cluster RCT; PAID; 4.5 years | 498 (255/243) | NR | NR | T2D | Primary care practices | 0 (Screen-detected at study entry) | Intensified multi-factorial treatment: stricter targets for $HbA_{1c}$, BP, lipids; combination therapy; aspirin use encouraged vs CAU | NR | NR | Diverse | Medication |
| van Dijk-de Vries, 2015, Netherlands (224) | Cluster RCT; PAID; 12 months | 261 (116/145) | 54% male | Mean age 65 years | T2D | Family practices | 8.5 years | Biopsychosocial Self-Management Support (SMS) implemented in routine care, in-person individual 3 month detection consultation & follow-up vs CAU | I 29.9±16.9 C 28.9±19.4 | I 53.0±35.0 mmol/mol (7.0±3.2%) C 51.9±33.9 mmol/mol (6.9±3.1%) | | |
| van Puffelen, 2019, Netherlands (225) | RCT; PAID; 6 months | 209 (107/102) | 55.3% male | Mean age 63.6 years | T2D | Clinical environment | 2.4 years | Group-based self-management support program, 3 monthly 2-hour interactive sessions & one booster session 3 months after last vs single 2-hour educational lectur & CAU | I 12.6±14.3 C 13.9±16.0 | NR | Education | |
| Vaughan, 2022, USA (226) | RCT; PAID; 12 months | 225 (136/89) | 89.8% male | Mean age 61.9 years | T1D/T2D (NR) | Hospital-based primary care clinics; community-based outpatient | NR | HOPE: phone-based one-on-one collaborative, goal-setting Active intervention biweekly (30Ð40 min) (over 6 months), 1Ð3 and monthly (15 min) for sessions 4Ð6. 3×15 min maintenance during months 7Ð12 vs education enhanced usual care | I 48.2±20.9 C 49.5±17.3 | I 77.1±15.3 mmol/mol (9.2±1.4%) C 78.1±16.4 mmol/mol (9.3±1.5%) | | |
| Wagner, 2016, USA (227) | RCT; PAID; 3 months | 107 (61/46) | 27.1% male | Mean age 60 years | T2D | Outpatient clinic; community organisation | NR | 8 group in-person sessions across 8–10 weeks, community health worker (CHW) delivered stress management (SM) w/ CD at-home practice VS. CHW- | I 7.9±6.7 C 8.1±6.3 | I 69.4±15.3 mmol/mol (8.5±1.4%) C 70.5±20.8 mmol/mol (8.6±1.9%) | Education | |

| | | | | | | | | | | | | |
|---|---|---|---|---|---|---|---|---|---|---|---|---|
| | | | | | | | | delivered diabetes education | | | | |
| Watson, 2018, Australia (228) | RCT; PAID; 24 weeks | 61 (32/29) | 51.1% male | Mean age 54.9 years | T2D | General community | 7.2 years | High Protein diet (HP: 32% protein, 33% carbs) vs High carb diet (HC: 22% protein, 51% carbs); both with moderate-intensity aerobic exercise (150 min/week | I 23.9±19.7 C 25.6±18.5 | NR | Lifestyle | |
| Weinger, 2011, USA (229) | RCT; PAID; 12 months | 222 (74/75/73) | 49.5% male | Mean age 53 years | T1D/T2D (49.5%/50.5% ) | Clinical practice | 18 years | Behavioural diabetes intervention (Ia): in-person, 5 sessions over 6 weeks, manual-based, structured group diabetes education (self-care behaviours and cognitive behavioural strategies). Vs. (Ib) 5 sessions over 6 weeks, manual-based attention control group education vs (C) Individual diabetes education sessions (6 months) | Ia 34.8 ± 19.3 Ib 33.6±20.8 C 34.0 ± 21.5 | I 76.2±12.0 mmol/mol (9.1±1.1%) C 75.9±13.1 mmol/mol (9.1±1.2%) C 73.8±12.0 mmol/mol (8.9±1.1%) | | |
| Weissberg-Benchell, 2017, USA (230) | RCT cross-over; T1D-DDS; 0.8 months | 39 (39/39) | 46 % male | Mean age 33.3 years | T1D | Study centres | 17 years | Individually, in person, bionic pancreas system (BP), where Insulin and glucagon were automatically administered by a fully integrated bihormonal BP for 11 days vs CAU | I 2.37±0.84 C 1.77±0.37 | I 61.0±15.3 mmol/mol (7.7±1.4%) C 61.3±9.8 mmol/mol (7.8±0.9%) | Devices | |
| Weissberg-Benchell, 2023, USA (231) | RCT; DDS; 3 months | 275 (221/54) | 49% male | Mean age 43 years | T1D | 16 centres in the USA | 26 years | Individual, automated insulin delivery using a bionic pancreas initialised using the person's body weight for 3 months vs standard care | I 1.9±0.7 C 1.8±0.7 | NR | Devices | |
| Welch, 2011, USA (232) | RCT; PAID; 6 months | 234 (57/61/58/58) | 41% male | Mean age 55.7 years | T2D | Diabetes clinic | 8.2 years | DSME session for one hour followed by 3×30 min sessions at 1 month, 3 months, and 6 months research visits at baseline and at 6 months. AND diabetes self-management education with motivational interviewing with (Ia)/without (Ib) reporting | Ia 40.5±23.3 Ib 41.9±22.4 Ic 43.4±25.0 C 42.5±23.6 | Ia 76.0±16.4 mmol/mol (9.1±1.5%) Ib 72.7±10.9 mmol/mol (8.8±1.0%) Ic 72.7±14.2 mmol/mol (8.8±1.3%) C 73.8±13.1 mmol/mol (8.9±1.2%) | | |

| | | | | | | | | | | | | |
|---|---|---|---|---|---|---|---|---|---|---|---|---|
| | | | | | | | | web-tool in-person, individual OR diabetes self-management education with (Ic)/without reporting webtool (C) | | | | |
| Welch, 2015, USA (233) | RCT; PAID; 6 months | 399 (200/199) | 40% male | Mean age 55 years | T2D | Urban community health centres | NR | Internet-based diabetes dashboard used as a system for team care. 5 in-person, individual diabetes education with diabetes nurse/dietitian (at baseline, 2 weeks, 1 month, 3 month, 6 months) vs CAU | I 59.0± 30.5 C 51.9± 32.3 | I 73.8±15.3 mmol/mol (8.9±1.4%) C 74.9±16.4 mmol/mol (9.0±1.5%) | | |
| Welch, 2011, USA (234) | RCT; PAID; 12 months | 46 (25/21) | 35 % Male | Mean age 55.5 years | T2D | Urban community health centre | 12 years | Individually, in person, nurse-led diabetes care program (Comprehensive Diabetes Management Program, CDMP) of seven 1-hour diabetes care visits over a 12-month combined with 5 risk profile discussed with GP vs attention control of seven 1-hour visits over a 12-month period on ADA diabetes education | I 54.2±24.0 C 44.3±23.0 | I 74.9±13.1 mmol/mol (9.0±1.2%) C 69.4±10.9 mmol/mol (8.5±1.0%) | Education | |
| Wexler, 2022, USA (235) | RCT; PAID; 36 month | 211 (69/70/72) | 44.7% male | Mean age 61.7 years | T2D | Diabetes centre | 10.2 years | Adapted version of the Look AHEAD lifestyle intervention, a practice-based, three-arm, randomised comparative effectiveness trial with 1:1:1 allocation to in-person lifestyle intervention (IPLI; Ia), telephone lifestyle intervention (TLI; Ib), or referral to a dietitian for medical nutrition therapy (Ic). | Ia 39.3± 19.5 Ib 40.3±23.3 Ic 36.9±23.1 | Ia 61.8±13.1 mmol/mol (7.8±1.2%) Ib 59.6±13.1 mmol/mol (7.6±1.2%) Ic 61.8±13.1 mmol/mol (7.8±1.2%) | Lifestyle | |
| Whittemore, 2004, USA (236) | RCT; PAID; 6 months | 49(26/23) | 0% male | Mean age 57.6 years | T2D | Outpatient diabetes education centre | 2.7 years | Nurse-coaching sessions: educational, behavioural, and affective strategies. in-person individual every 2 weeks - 3 sessions, every month - 2 sessions, and, after 3 months (6 total over 6 months) + brief phone calls vs CAU | I 59.9±22 C 42.3±14 | I 60.7±10.9 mmol/mol (7.7±1.0%) C 59.6±10.9 mmol/mol (7.6±1.0%) | | |

| | | | | | | | | | | | | |
|---|---|---|---|---|---|---|---|---|---|---|---|---|
| Wijk, 2023, Sweden (237) | RCT; PAID; 24 months | 81 (43/38) | 37% male | Mean age 40.2 years | T1D | Endocrinology clinics | 23.4 years | Face-to-face ACT-based group discussions, psychoeducation, exercises, mindfulness practice, role play and value awareness. 7×2-hour sessions held every other week (14 weeks). In groups of 6–9 Vs. CAU | I 36.6±18.1 C 42.9±15.9 | I 71.6 ± NR mmol/mol (8.7 ± NR%) C 76.0 ± NR mmol/mol (9.1 ± NR%) | | |
| Winkelman, 2023, USA (238) | RCT sequential parallel comparison design; DDS; 3 months | 67 (44/23) | 34.8% male | Mean age 60.0 years | T2D | Diabetes clinic | NR | Suvorexant 10–20 mg for insomnia vs placebo | I 28.7±10.4 C 26.2±5.7 | I 57.4±10.9 mmol/mol (7.4±1.0%) C 57.4±7.7 mmol/mol (7.4±0.7%) | Diverse | Medication |
| Winkley, 2023, UK (239) | RCT; PAID; 3 months | 17 (9/8) | 41.2% male | Mean age 58.35 years | T2D | Primary care practices | 12.4 years | Diabetes insulin self-management education using motivational interviewing. face-to-face group sessions for 1Ð2 h on week 1 and week 3 3rd session ~ 6 weeks later (within 3 months) VS. standard group insulin start (follow-up via phone) | I 32.50±23.9 C 46.67±22.4 | I 86.9±40.4 mmol/mol (10.1±3.7%) C 89.1±39.3 mmol/mol (10.3±3.6%) | | |
| Witkow, 2023, Israel (240) | RCT; PAID; 6 months | 85 (44/41) | 52.9% male | Mean age 43.2 years | T1D | Tertiary hospital | 15.8 years | Individually, combined, Simplified individualised carb counting (SCC) tool with personalised insulin dosing tables vs standard/regular carb counting (RCC) using booklets/apps and insulin-to-carb ratio | I 9.3±5.4 C 12.5±5.4 | NR | Lifestyle | Lifestyle |
| Woodard, 2022, USA (241) | RCT; DDS; 10 months | 280 (140/140) | 94.3% male | Mean age 67.2 years | T2D | Veterans Affairs clinics | NR | Empowering Patients in Chronic Care (EPICC) 6 bimonthly in-person group (~1 hour), collaborative goal setting and motivational interviewing (3-month duration). Vs. CAU/enhanced education | I 2.41±1.05 C 2.45±1.02 | I 76.1±17.5 mmol/mol (9.1±1.6%) C 75.5±14.4 mmol/mol (9.1±1.3%) | Education | |
| Wycherley, 2014, Australia (242) | RCT; PAID; 16 weeks | 106 (65/41) | 55.7 % male | Mean age 56.1 years | T2D | Research clinic | NR | Calorie-restricted diet (6000–7000 kJ/day) + supervised resistance exercise (3x/ week) vs Calorie-restricted diet (6000–7000 kJ/day) | I 24 ± NR C 40 ± NR | NR | Lifestyle | |

| | | | | | | | | | | | | |
|---|---|---|---|---|---|---|---|---|---|---|---|---|
| Yanhong, 2020, China (243) | RCT; DDS; 2.8 months | 100 (50/50) | 52% male | Mean age 58.06 years | T2D | In-hospital endocrinology department | 13.68 years | Eight weekly in-person group mindfulness training based on meta-awareness by psychological counsellors and practising daily for 45 minutes every day during hospitalisation and after discharge combined with intermittent pneumatic compression in group sessions of 4–6 people, 1 hour duration, four times a week. Patients recorded experiences and communicated with each other in a WeChat group. vs Traditional comprehensive diabetes education and treatment, including diet control, exercise therapy, oral hypoglycaemic drugs and insulin application, blood sugar monitoring, psychological counselling and other education to lower blood sugar, blood pressure, lipids, improve circulation, and nourish nerves. | I 2.45±0.26 C 2.49±0.31 | I 68.1±15.7 mmol/mol (8.4±1.4%) C 68.6±14.2 mmol/mol (8.4±1.3%) | Education | |
| Yu, 2020, Canada (244) | Cluster RCT; DDS-17; 12 months | 213 (102/111) | 52.1% male | NR | T1D/T2D (NR) | Interprofessional primary care practices | NR | IMyDiabetesPlan: interactive web-based shared decision-making and goal-setting tool, provider- and patient-directed vs received standard Diabetes Canada educational resources (paper/web-based) | I 2.08 ± 1.02 C 1.93±0.83 | NR | telehealth | |
| Zhang, 2024, China (245) | Cluster RCT; PAID; 3 months | 147 (72/75) | 54% male | Mean age 52 years | T2D | Primary care practices | 7 years | Dietary education: nudge-strategy based in-person group 60–90 min 1/week sessions vs traditional dietary education intervention (same duration) | I 31± 12 C 30± 11 | I 68.9±61.9 mmol/mol (8.5±5.7%) C 61.2±21.3 mmol/mol (7.8±2.0%) | Education | |

| | | | | | | | | | | | | |
|---|---|---|---|---|---|---|---|---|---|---|---|---|
| Zheng, 2019, China (246) | RCT; PAID; 3 months | 60 (30/30) | 55% male | Mean age 55.2 years | T2D | Cardiovascular rehabilitation clinic, endocrinology clinic and geriatric clinics | 2.46 years | Regular education + a 2-session diabetes self-management education besides the regular education program. 2×45 min theory course on diabetes knowledge and self-management strategies. 2 × practical course - 1x ono-to-one nutrition guidance (40 minutes) and 1x individualised exercise guidance (personalised exercise program that should be done 3x a week for 60–90 minutes) vs CAU Regular education. | I 28.91±14.76 , C 30.72±15.86 | I 67.2±11.1 mmol/mol (8.3±1.0%) C 69.2±4.4 mmol/mol (8.5±0.4%) | Education | |
| Zoffmann, 2006, Denmark (247) | RCT; PAID; 12 months | 61 (36/25) | 48% male | Mean age 36.3 years | T1D | Hospital clinics | NR | Guided self-determination (GSD) based group training. Face to face in groups of 10 members over 8 weeks for 2h sessions. Facilitated by GSD-trained nurses. vs waiting list | I 32.0 ±(se 3.4) C 40.9 (se 4.0) | I 75.0±1.3 mmol/mol (9.0±0.1%) C 75.5±1.1 mmol/mol (9.1±0.1%) | | |
| Zoffmann, 2015, Denmark (248) | RCT; PAID; 18 months | 200 (134/66) | 50 % male | Mean age 25.7 years | T1D | Clinics | 13.7 years | Flexible version of guided self-determination (GSD) (group based/individual), theory driven. Combination of seven individual 1Ðhour daytime sessions and 2.5-hour group sessions facilitated by diabetes nurse specialists. Preferably 14 days between sessions the intervention took ~ 3.5 months (with possible extension) vs CAU | I 36.4±21.0 C 35.2±22.7 | I 80.3±14.2 mmol/mol (9.5±1.3%) C 82.5±16.4 mmol/mol (9.7±1.5%) | | |

Supplementary Table S1: Baseline characteristics. C, control arm; CAU, care as usual; CBT, cognitive behavioural therapy; C-DDS, Chinese Diabetes Distress Scale; CGM, continuous glucose monitoring; DD, diabetes distress; DDS, Diabetes Distress Scale; I, intervention arm; Ia, first intervention arm; Ib, second intervention arm; Ic, third intervention arm; NR, not reported; T1D, type 1 diabetes; T2D, type 2 diabetes

## References


1. Abbas Q, Latif S, Ayaz Habib H, Shahzad S, Sarwar U, Shahzadi M, et al. Cognitive behavior therapy for diabetes distress, depression, health anxiety, quality of life and treatment adherence among patients with type-II diabetes mellitus: a randomized control trial. BMC Psychiatry. 2023;23(1):86.
2. Agarwal P, Mukerji G, Desveaux L, Ivers NM, Bhattacharyya O, Hensel JM, et al. Mobile App for Improved Self-Management of Type 2 Diabetes: Multicenter Pragmatic Randomized Controlled Trial. JMIR Mhealth Uhealth. 2019;7(1):e10321.
3. Albabtain B, Bawazeer G, Paudyal V, Cheema E, Alqahtani A, Bahatheq A, et al. Impact of a community pharmacy-based medication therapy management program on clinical and humanistic outcomes in patients with uncontrolled diabetes: a randomised controlled trial. Sci Rep. 2024;14(1):17818.
4. Alessi J, de Oliveira GB, Franco DW, Becker AS, Knijnik CP, Kobe GL, et al. Telehealth strategy to mitigate the negative psychological impact of the COVID-19 pandemic on type 2 diabetes: A randomized controlled trial. Acta Diabetol. 2021;58(7):899–909.
5. Alessi J, Becker AS, Amaral B, de Oliveira GB, Franco DW, Knijnik CP, et al. Type 1 diabetes and the challenges of emotional support in crisis situations: results from a feasibility study of a multidisciplinary teleintervention. Sci Rep. 2022;12(1):8526.
6. Amiel SA, Potts L, Goldsmith K, Jacob P, Smith EL, Gonder-Frederick L, et al. A parallel randomised controlled trial of the Hypoglycaemia Awareness Restoration Programme for adults with type 1 diabetes and problematic hypoglycaemia despite optimised self-care (HARPdoc). Nat Commun. 2022;13(1):2229.
7. Amsberg S, Anderbro T, Wredling R, Lisspers J, Lins PE, Adamson U, et al. A cognitive behavior therapy-based intervention among poorly controlled adult type 1 diabetes patients--a randomized controlled trial. Patient Educ Couns. 2009;77(1):72–80.
8. Anderson RM, Funnell MM, Aikens JE, Krein SL, Fitzgerald JT, Nwankwo R, et al. Evaluating the Efficacy of an Empowerment-Based Self-Management Consultant Intervention: Results of a Two-Year Randomized Controlled Trial. Ther Patient Educ. 2009;1(1):3–11.
9. Anjali M, Khapre M, Kant R, Kumar S, Pandey P. Effectiveness of Diabetes Self-Management Education on Distress and HbA1C among Indian Type 2 Diabetes Mellitus Patients: A Randomized Controlled Trial. Indian J Community Med. 2023;48(5):702–8.
10. Aronson R, Brown RE, Chu L, Bajaj HS, Khandwala H, Abitbol A, et al. IMpact of flash glucose Monitoring in pEople with type 2 Diabetes Inadequately controlled with non-insulin Antihyperglycaemic ThErapy (IMMEDIATE): A randomized controlled trial. Diabetes Obes Metab. 2023;25(4):1024–31.
11. Bae JH, Park EH, Lee HK, Yoon KH, Won KC, Kim HM, et al. Enhancing Diabetes Care through a Mobile Application: A Randomized Clinical Trial on Integrating Physical and Mental Health among Disadvantaged Individuals. Diabetes Metab J. 2024;48(4):790–801.
12. Baig AA, Staab EM, Zhu M, Nunez D, Li J, Wan W, et al. Cluster Randomized Trial of Diabetes Group Visits in Community Health Centers across the Midwest. J Health Care Poor Underserved. 2024;35(3s):27–46.

13. Bajaj HS, Venn K, Ye C, Aronson R. Randomized Trial of Long-Acting Insulin Glargine Titration Web Tool (LTHome) Versus Enhanced Usual Therapy of Glargine Titration (INNOVATE Trial). Diabetes Technol Ther. 2016;18(10):610–5.
14. Baldwin PA, Sanatkar S, Clarke J, Fletcher S, Gunn J, Wilhelm K, et al. A Web-Based Mental Health Intervention to Improve Social and Occupational Functioning in Adults With Type 2 Diabetes (The Springboard Trial): 12-Month Outcomes of a Randomized Controlled Trial. J Med Internet Res. 2020;22(12):e16729.
15. Basak Cinar A, Schou L. Health promotion for patients with diabetes: health coaching or formal health education? Int Dent J. 2014;64(1):20–8.
16. Beck RW, Riddlesworth TD, Ruedy K, Ahmann A, Haller S, Kruger D, et al. Continuous Glucose Monitoring Versus Usual Care in Patients With Type 2 Diabetes Receiving Multiple Daily Insulin Injections: A Randomized Trial. Ann Intern Med. 2017;167(6):365–74.
17. Bendig E, Bauereiss N, Schmitt A, Albus P, Baumeister H. ACTonDiabetes-a guided psychological internet intervention based on Acceptance and Commitment Therapy (ACT) for adults living with type 1 or 2 diabetes: results of a randomised controlled feasibility trial. BMJ Open. 2021;11(7):e049238.
18. Benhalima K, Beunen K, Van Wilder N, Ballaux D, Vanhaverbeke G, Taes Y, et al. Comparing advanced hybrid closed loop therapy and standard insulin therapy in pregnant women with type 1 diabetes (CRISTAL): a parallel-group, open-label, randomised controlled trial. The Lancet Diabetes & Endocrinology. 2024;12(6):390–403.
19. Beverly EA, Fitzgerald SM, Brooks KM, Hultgren BA, Ganda OP, Munshi M, et al. Impact of reinforcement of diabetes self-care on poorly controlled diabetes: a randomized controlled trial. Diabetes Educ. 2013;39(4):504–14.
20. Binesh M, Shafaroodi N, Mirmohammadkhani M, Aghili R, Motaharinezhad F, Khanipour M, et al. A randomized controlled trial for evaluating an occupational therapy self management intervention in adults with type 2 diabetes. Sci Rep. 2023;13(1):10128.
21. Bisno DI, Reid MW, Fogel JL, Pyatak EA, Majidi S, Raymond JK. Virtual Group Appointments Reduce Distress and Improve Care Management in Young Adults with Type 1 Diabetes. J Diabetes Sci Technol. 2022;16(6):1419–27.
22. Bock BC, Thind H, Fava JL, Dunsiger S, Guthrie KM, Stroud L, et al. Feasibility of yoga as a complementary therapy for patients with type 2 diabetes: The Healthy Active and in Control (HA1C) study. Complement Ther Med. 2019;42:125–31.
23. Bolinder J, Antuna R, Geelhoed-Duijvestijn P, Kroger J, Weitgasser R. Novel glucose-sensing technology and hypoglycaemia in type 1 diabetes: a multicentre, non-masked, randomised controlled trial. Lancet. 2016;388(10057):2254–63.
24. Bond GE, Burr RL, Wolf FM, Feldt K. The Effects of a Web-Based Intervention on Psychosocial Well-Being Among Adults Aged 60 and Older With Diabetes. The Diabetes Educator. 2010;36(3):446–56.
25. Boscari F, Vettoretti M, Cavallin F, Amato AML, Uliana A, Vallone V, et al. Implantable and transcutaneous continuous glucose monitoring system: a randomized cross over trial comparing accuracy, efficacy and acceptance. J Endocrinol Invest. 2022;45(1):115–24.
26. Brennan MC, Albrecht MA, Brown JA, Leslie GD, Ntoumanis N. Self-Management Group Education to Reduce Fear of Hypoglycemia as a Barrier to Physical Activity in Adults Living With Type 1 Diabetes: A Pilot Randomized Controlled Trial. Can J Diabetes. 2021;45(7):619–28.

27. Brinkworth GD, Luscombe-Marsh ND, Thompson CH, Noakes M, Buckley JD, Wittert G, et al. Long-term effects of very low-carbohydrate and high-carbohydrate weight-loss diets on psychological health in obese adults with type 2 diabetes: randomized controlled trial. J Intern Med. 2016;280(4):388–97.
28. Browne JL, Speight J, Martin C, Gilfillan C. Building the evidence for integrated care for type 2 diabetes: a pilot study. Aust J Prim Health. 2016;22(5):409–15.
29. Bruijnes M, Kesteloo M, Brinkman WP. Reducing social diabetes distress with a conversational agent support system: a three-week technology feasibility evaluation. Front Digit Health. 2023;5:1149374.
30. Burner E, Lam CN, DeRoss R, Kagawa-Singer M, Menchine M, Arora S. Using Mobile Health to Improve Social Support for Low-Income Latino Patients with Diabetes: A Mixed-Methods Analysis of the Feasibility Trial of TExT-MED + FANS. Diabetes Technology & Therapeutics. 2017;20(1):39–48.
31. Callan JA, Sereika SM, Cui R, Tamres LK, Tarneja M, Greene B, et al. Cognitive Behavioral Therapy (CBT) Telehealth Augmented With a CBT Smartphone Application to Address Type 2 Diabetes Self-Management: A Randomized Pilot Trial. Sci Diabetes Self Manag Care. 2022;48(6):492–504.
32. Carreira M, Ruiz de Adana MS, Pinzón JL, Anarte-Ortiz MT. Internet-based cognitive-behavioral therapy is effective in reducing depressive symptomatology in type 1 diabetes: results of a randomized controlled trial. Front Clin Diabetes Healthc. 2023;4:1209236.
33. Chan JC, Sui Y, Oldenburg B, Zhang Y, Chung HH, Goggins W, et al. Effects of telephone-based peer support in patients with type 2 diabetes mellitus receiving integrated care: a randomized clinical trial. JAMA Intern Med. 2014;174(6):972–81.
34. Cheng L, Sit JWH, Choi KC, Chair SY, Li X, Wu Y, et al. The effects of an empowerment-based self-management intervention on empowerment level, psychological distress, and quality of life in patients with poorly controlled type 2 diabetes: A randomized controlled trial. Int J Nurs Stud. 2021;116:103407.
35. Chew BH, Vos RC, Fernandez A, Shariff Ghazali S, Shamsuddin NH, Ismail M, et al. The effectiveness of an emotion-focused educational programme in reducing diabetes distress in adults with type 2 diabetes mellitus at 12-month follow-up: a cluster randomized controlled trial. Ther Adv Endocrinol Metab. 2019;10:2042018819853761.
36. Chiu CJ, Hu YH, Wray LA, Beverly EA, Yang YC, Wu JS, et al. Dissemination of evidence-base minimal psychological intervention for diabetes management in Taiwan adults with type 2 diabetes. International journal of clinical and experimental medicine. 2016;9(7):14489–98.
37. Clark TL, Gallo L, Euyoque JA, Philis-Tsimikas A, Fortmann A. Does Diabetes Distress Influence Clinical Response to an mHealth Diabetes Self-Management Education and Support Intervention? Diabetes Educ. 2020;46(3):289–96.
38. Coccaro EF, Drossos T, Kline D, Lazarus S, Joseph JJ, de Groot M. Diabetes distress, emotional regulation, HbA(1c) in people with diabetes and A controlled pilot study of an emotion-focused behavioral therapy intervention in adults with type 2 diabetes. Prim Care Diabetes. 2022;16(3):381–6.
39. Cohen E, Tsoukas MA, Legault L, Vallis M, Von Oettingen JE, Palisaitis E, et al. Simple meal announcements and pramlintide delivery versus carbohydrate counting in type 1 diabetes with automated fast-acting insulin aspart delivery: a randomised crossover trial in Montreal, Canada. Lancet Digit Health. 2024;6(7):e489–e99.

40. Cohn MA, Pietrucha ME, Saslow LR, Hult JR, Moskowitz JT. An online positive affect skills intervention reduces depression in adults with type 2 diabetes. The Journal of Positive Psychology. 2014;9(6):523–34.
41. Cornely RM, Subramanya V, Owen A, McGee RE, Kulshreshtha A. A mixed-methods approach to understanding the perspectives, experiences, and attitudes of a culturally tailored cognitive behavioral therapy/motivational interviewing intervention for African American patients with type 2 diabetes: a randomized parallel d. Pilot Feasibility Stud. 2022;8(1):107.
42. Cox DJ, Oser T, Moncrief M, Conaway M, McCall A. Long-term follow-up of a randomized clinical trial comparing glycemic excursion minimization (GEM) to weight loss (WL) in the management of type 2 diabetes. BMJ Open Diabetes Res Care. 2021;9(2).
43. Crawford J, Wilhelm K, Proudfoot J. Web-Based Benefit-Finding Writing for Adults with Type 1 or Type 2 Diabetes: Preliminary Randomized Controlled Trial. JMIR Diabetes. 2019;4(2):e13857.
44. Crowley MJ, Tarkington PE, Bosworth HB, Jeffreys AS, Coffman CJ, Maciejewski ML, et al. Effect of a Comprehensive Telehealth Intervention vs Telemonitoring and Care Coordination in Patients With Persistently Poor Type 2 Diabetes Control: A Randomized Clinical Trial. JAMA Intern Med. 2022;182(9):943–52.
45. D'Eramo Melkus G, Chyun D, Vorderstrasse A, Newlin K, Jefferson V, Langerman S. The effect of a diabetes education, coping skills training, and care intervention on physiological and psychosocial outcomes in black women with type 2 diabetes. Biol Res Nurs. 2010;12(1):7–19.
46. Dale J, Caramlau I, Sturt J, Friede T, Walker R. Telephone peer-delivered intervention for diabetes motivation and support: The telecare exploratory RCT. Patient Education and Counseling. 2009;75(1):91–8.
47. Danne T, Cariou B, Banks P, Brandle M, Brath H, Franek E, et al. HbA(1c) and Hypoglycemia Reductions at 24 and 52 Weeks With Sotagliflozin in Combination With Insulin in Adults With Type 1 Diabetes: The European inTandem2 Study. Diabetes Care. 2018;41(9):1981–90.
48. Davies MJ, Heller S, Skinner TC, Campbell MJ, Carey ME, Cradock S, et al. Effectiveness of the diabetes education and self management for ongoing and newly diagnosed (DESMOND) programme for people with newly diagnosed type 2 diabetes: cluster randomised controlled trial. BMJ. 2008;336(7642):491–5.
49. de Groot M, Shubrook JH, Hornsby WG, Jr., Pillay Y, Mather KJ, Fitzpatrick K, et al. Program ACTIVE II: Outcomes From a Randomized, Multistate Community-Based Depression Treatment for Rural and Urban Adults With Type 2 Diabetes. Diabetes Care. 2019;42(7):1185–93.
50. Dennick K, Bridle C, Sturt J. Written emotional disclosure for adults with Type 2 diabetes: a primary care feasibility study. Prim Health Care Res Dev. 2015;16(2):179–87.
51. DiNardo MM, Greco C, Phares AD, Beyer NM, Youk AO, Obrosky DS, et al. Effects of an integrated mindfulness intervention for veterans with diabetes distress: a randomized controlled trial. BMJ Open Diabetes Res Care. 2022;10(2).
52. Ding SA, Simonson DC, Wewalka M, Halperin F, Foster K, Goebel-Fabbri A, et al. Adjustable Gastric Band Surgery or Medical Management in Patients With Type 2 Diabetes: A Randomized Clinical Trial. J Clin Endocrinol Metab. 2015;100(7):2546–56.
53. Dinneen SF, O'Hara MC, Byrne M, Smith D, Courtney CH, McGurk C, et al. Group follow-up compared to individual clinic visits after structured education for type 1 diabetes: A cluster randomised controlled trial. Diabetes Research and Clinical Practice. 2013;100(1):29–38.

54. Dobson R, Whittaker R, Jiang Y, Maddison R, Shepherd M, McNamara C, et al. Effectiveness of text message based, diabetes self management support programme (SMS4BG): two arm, parallel randomised controlled trial. Bmj. 2018;361:k1959.
55. Drion I, Pameijer LR, van Dijk PR, Groenier KH, Kleefstra N, Bilo HJ. The Effects of a Mobile Phone Application on Quality of Life in Patients With Type 1 Diabetes Mellitus: A Randomized Controlled Trial. J Diabetes Sci Technol. 2015;9(5):1086–91.
56. Ehrmann D, Heinemann L, Freckmann G, Waldenmaier D, Faber-Heinemann G, Hermanns N. The Effects and Effect Sizes of Real-Time Continuous Glucose Monitoring on Patient-Reported Outcomes: A Secondary Analysis of the HypoDE Study. Diabetes Technology & Therapeutics. 2019;21(2):86–93.
57. Ewen AM, Hawkins JM, Kloss KA, Nwankwo R, Funnell MM, Sengupta S, et al. The Michigan Men's Diabetes Project Randomized Clinical Control Trial: A Pilot/Feasibility Study of a Peer-Led Diabetes Self-Management and Support Intervention for Black Men With Type 2 Diabetes. Am J Mens Health. 2024;18(3):15579883241258318.
58. Fisher L, Polonsky W, Parkin CG, Jelsovsky Z, Amstutz L, Wagner RS. The impact of blood glucose monitoring on depression and distress in insulin-naïve patients with type 2 diabetes. Curr Med Res Opin. 2011;27 Suppl 3:39–46.
59. Fisher L, Hessler D, Glasgow RE, Arean PA, Masharani U, Naranjo D, et al. REDEEM: a pragmatic trial to reduce diabetes distress. Diabetes Care. 2013;36(9):2551–8.
60. Fisher L, Hessler D, Polonsky WH, Masharani U, Guzman S, Bowyer V, et al. T1-REDEEM: A Randomized Controlled Trial to Reduce Diabetes Distress Among Adults With Type 1 Diabetes. Diabetes Care. 2018;41(9):1862–9.
61. Frias JP, Lee ML, Carter MM, Ebel ER, Lai RH, Rikse L, et al. A microbiome-targeting fibre-enriched nutritional formula is well tolerated and improves quality of life and haemoglobin A1c in type 2 diabetes: A double-blind, randomized, placebo-controlled trial. Diabetes Obes Metab. 2023;25(5):1203–12.
62. Friis AM, Johnson MH, Cutfield RG, Consedine NS. Kindness Matters: A Randomized Controlled Trial of a Mindful Self-Compassion Intervention Improves Depression, Distress, and HbA1c Among Patients With Diabetes. Diabetes Care. 2016;39(11):1963–71.
63. Furler J, O'Neal D, Speight J, Manski-Nankervis JA, Gorelik A, Holmes-Truscott E, et al. Supporting insulin initiation in type 2 diabetes in primary care: results of the Stepping Up pragmatic cluster randomised controlled clinical trial. Bmj. 2017;356:j783.
64. Furler J, O'Neal D, Speight J, Blackberry I, Manski-Nankervis JA, Thuraisingam S, et al. Use of professional-mode flash glucose monitoring, at 3-month intervals, in adults with type 2 diabetes in general practice (GP-OSMOTIC): a pragmatic, open-label, 12-month, randomised controlled trial. Lancet Diabetes Endocrinol. 2020;8(1):17–26.
65. Gabbay RA, Lendel I, Saleem TM, Shaeffer G, Adelman AM, Mauger DT, et al. Nurse case management improves blood pressure, emotional distress and diabetes complication screening. Diabetes Res Clin Pract. 2006;71(1):28–35.
66. Gabbay RA, Añel-Tiangco RM, Dellasega C, Mauger DT, Adelman A, Van Horn DH. Diabetes nurse case management and motivational interviewing for change (DYNAMIC): results of a 2-year randomized controlled pragmatic trial. J Diabetes. 2013;5(3):349–57.

67. Gagliardino JJ, Arrechea V, Assad D, Gagliardino GG, González L, Lucero S, et al. Type 2 diabetes patients educated by other patients perform at least as well as patients trained by professionals. Diabetes/Metabolism Research and Reviews. 2013;29(2):152–60.
68. Gerber BS, Biggers A, Tilton JJ, Smith Marsh DE, Lane R, Mihailescu D, et al. Mobile Health Intervention in Patients With Type 2 Diabetes: A Randomized Clinical Trial. JAMA Netw Open. 2023;6(9):e2333629.
69. Giannoulaki P, Kotzakioulafi E, Nakas A, Kontoninas Z, Karlafti E, Evripidou P, et al. Effect of Crocus sativus Extract Supplementation in the Metabolic Control of People with Diabetes Mellitus Type 1: a Double-Blind Randomized Placebo-Controlled Trial. Nutrients. 2024;16(13).
70. Glasgow RE, Nutting PA, King DK, Nelson CC, Cutter G, Gaglio B, et al. A practical randomized trial to improve diabetes care. J Gen Intern Med. 2004;19(12):1167–74.
71. Gonzalez JS, Hoogendoorn CJ, Schechter CB, Pappalardo L, Fernandez Galvis MA, Linnell J, et al. Outcomes of New York City Care Calls: A Prospective Randomized Controlled Effectiveness Trial of Telephone-Delivered Type 2 Diabetes Self-Management Support. Sci Diabetes Self Manag Care. 2024;50(3):235–49.
72. Grillo Mde F, Neumann CR, Scain SF, Rozeno RF, Beloli L, Perinetto T, et al. Diabetes education in primary care: a randomized clinical trial. Cad Saude Publica. 2016;32(5).
73. Griva K, Rajeswari M, Nandakumar M, Khoo EYH, Lee VYW, Chua CG, et al. The combined diabetes and renal control trial (C-DIRECT) - a feasibility randomised controlled trial to evaluate outcomes in multi-morbid patients with diabetes and on dialysis using a mixed methods approach. BMC Nephrol. 2019;20(1):2.
74. Guo J, Wang H, Ge L, Valimaki M, Wiley J, Whittemore R. Effectiveness of a nurse-led mindfulness stress-reduction intervention on diabetes distress, diabetes self-management, and HbA1c levels among people with type 2 diabetes: A pilot randomized controlled trial. Res Nurs Health. 2022;45(1):46–58.
75. Haak T, Hanaire H, Ajjan R, Hermanns N, Riveline J-P, Rayman G. Flash Glucose-Sensing Technology as a Replacement for Blood Glucose Monitoring for the Management of Insulin-Treated Type 2 Diabetes: a Multicenter, Open-Label Randomized Controlled Trial. Diabetes Therapy. 2017;8(1):55–73.
76. Halperin F, Ding SA, Simonson DC, Panosian J, Goebel-Fabbri A, Wewalka M, et al. Roux-en-Y gastric bypass surgery or lifestyle with intensive medical management in patients with type 2 diabetes: feasibility and 1-year results of a randomized clinical trial. JAMA Surg. 2014;149(7):716–26.
77. Heinrich E, Candel MJJM, Schaper NC, de Vries NK. Effect evaluation of a Motivational Interviewing based counselling strategy in diabetes care. Diabetes Research and Clinical Practice. 2010;90(3):270–8.
78. Heisler M, Choi H, Palmisano G, Mase R, Richardson C, Fagerlin A, et al. Comparison of community health worker-led diabetes medication decision-making support for low-income Latino and African American adults with diabetes using e-health tools versus print materials: a randomized, controlled trial. Ann Intern Med. 2014;161(10 Suppl):S13–S22.
79. Heisler M, Vijan S, Makki F, Piette JD. Diabetes control with reciprocal peer support versus nurse care management: a randomized trial. Ann Intern Med. 2010;153(8):507–15.

80. Hermanides J, Nørgaard K, Bruttomesso D, Mathieu C, Frid A, Dayan CM, et al. Sensor-augmented pump therapy lowers HbA(1c) in suboptimally controlled Type 1 diabetes; a randomized controlled trial. Diabet Med. 2011;28(10):1158–67.
81. Hermanns N, Kulzer B, Kubiak T, Krichbaum M, Haak T. The effect of an education programme (HyPOS) to treat hypoglycaemia problems in patients with type 1 diabetes. Diabetes/Metabolism Research and Reviews. 2007;23(7):528–38.
82. Hermanns N, Kulzer B, Maier B, Mahr M, Haak T. The effect of an education programme (MEDIAS 2 ICT) involving intensive insulin treatment for people with type 2 diabetes. Patient Educ Couns. 2012;86(2):226–32.
83. Hermanns N, Kulzer B, Ehrmann D, Bergis-Jurgan N, Haak T. The effect of a diabetes education programme (PRIMAS) for people with type 1 diabetes: results of a randomized trial. Diabetes Res Clin Pract. 2013;102(3):149–57.
84. Hermanns N, Ehrmann D, Schall S, Maier B, Haak T, Kulzer B. The effect of an education programme (MEDIAS 2 BSC) of non-intensive insulin treatment regimens for people with Type 2 diabetes: a randomized, multi-centre trial. Diabet Med. 2017;34(8):1084–91.
85. Hermanns N, Kulzer B, Gulde C, Eberle H, Pradler E, Patzelt-Bath A, et al. Short-term effects on patient satisfaction of continuous glucose monitoring with the GlucoDay with real-time and retrospective access to glucose values: a crossover study. Diabetes Technol Ther. 2009;11(5):275–81.
86. Hermanns N, Kulzer B, Kohlmann T, Jacob S, Landgraf W, Theobald K, et al. Treatment satisfaction and quality-of-life between type 2 diabetes patients initiating long- vs. intermediate-acting basal insulin therapy in combination with oral hypoglycemic agents--a randomized, prospective, crossover, open clinical trial. Health Qual Life Outcomes. 2015;13:77.
87. Hermanns N, Schmitt A, Gahr A, Herder C, Nowotny B, Roden M, et al. The effect of a Diabetes-Specific Cognitive Behavioral Treatment Program (DIAMOS) for patients with diabetes and subclinical depression: results of a randomized controlled trial. Diabetes Care. 2015;38(4):551–60.
88. Hernar I, Graue M, Richards DA, Strandberg RB, Nilsen RM, Rekdal M, et al. Use of patient-reported outcome measures (PROMs) in clinical diabetes consultations: the DiaPROM randomised controlled pilot trial. BMJ Open. 2021;11(4):e042353.
89. Hessler D, Fisher L, Dickinson M, Dickinson P, Parra J, Potter MB. The impact of enhancing self-management support for diabetes in Community Health Centers through patient engagement and relationship building: a primary care pragmatic cluster-randomized trial. Transl Behav Med. 2022;12(9):909–18.
90. Hessler DM, Fisher L, Guzman S, Strycker L, Polonsky WH, Ahmann A, et al. EMBARK: A Randomized, Controlled Trial Comparing Three Approaches to Reducing Diabetes Distress and Improving HbA1c in Adults With Type 1 Diabetes. Diabetes Care. 2024;47(8):1370–8.
91. Hilmarsdóttir E, Sigurðardóttir Á K, Arnardóttir RH. A Digital Lifestyle Program in Outpatient Treatment of Type 2 Diabetes: A Randomized Controlled Study. J Diabetes Sci Technol. 2021;15(5):1134–41.
92. Ho EY, Pak S, Leung G, Xu S, Yu CK, Hecht FM, et al. Pilot Cluster Randomized Controlled Trial of Integrative Nutritional Counseling Versus Standard Diabetes Self-Management Education for Chinese Americans with Type 2 Diabetes. Health Equity. 2020;4(1):410–20.
93. Holland-Carter L, Tuerk PW, Wadden TA, Fujioka KN, Becker LE, Miller-Kovach K, et al. Impact on psychosocial outcomes of a nationally available weight management program tailored for individuals with type 2 diabetes: Results of a randomized controlled trial. J Diabetes Complications. 2017;31(5):891–7.

94. Hoyo M, Rodrigo MTF, Urcola-Pardo F, Monreal-Bartolomé A, Ruiz DCG, Borao MG, et al. The TELE-DD Randomised Controlled Trial on Treatment Adherence in Patients with Type 2 Diabetes and Comorbid Depression: Clinical Outcomes after 18-Month Follow-Up. Int J Environ Res Public Health. 2022;20(1).
95. Ing CT, Zhang G, Dillard A, Yoshimura SR, Hughes C, Palakiko DM, et al. Social Support Groups in the Maintenance of Glycemic Control after Community-Based Intervention. J Diabetes Res. 2016;2016:7913258.
96. Ishii H, Nakajima H, Kamei N, Uchida D, Suzuki D, Ono Y, et al. Comparison of Patient-Led and Physician-Led Insulin Titration in Japanese Type 2 Diabetes Mellitus Patients Based on Treatment Distress, Satisfaction, and Self-Efficacy: The COMMIT-Patient Study. Diabetes Ther. 2021;12(2):595–611.
97. Ismail K, Winkley K, de Zoysa N, Patel A, Heslin M, Graves H, et al. Nurse-led psychological intervention for type 2 diabetes: a cluster randomised controlled trial (Diabetes-6 study) in primary care. Br J Gen Pract. 2018;68(673):e531–e40.
98. Iversen MM, Igland J, Smith-Strøm H, Østbye T, Tell GS, Skeie S, et al. Effect of a telemedicine intervention for diabetes-related foot ulcers on health, well-being and quality of life: secondary outcomes from a cluster randomized controlled trial (DiaFOTo). BMC Endocr Disord. 2020;20(1):157.
99. Izquierdo RE, Knudson PE, Meyer S, Kearns J, Ploutz-Snyder R, Weinstock RS. A comparison of diabetes education administered through telemedicine versus in person. Diabetes Care. 2003;26(4):1002–7.
100. Jiang XJ, Jiang H, Chen Y, Wu XA, Yu XL, Liu L, et al. The Effectiveness of a Self-Efficacy-Focused Structured Education Program (SSEP) in Improving Metabolic Control and Psychological Outcomes of Type 2 Diabetes Patients: A 12-Month Follow-Up of a Multicenter Randomized Controlled Trial. Diabetes Metab Syndr Obes. 2021;14:305–13.
101. Josefsson C, Liljeroos T, Hellgren M, Pöder U, Hedström M, Olsson EMG. The Sukaribit Smartphone App for Better Self-Management of Type 2 Diabetes: Randomized Controlled Feasibility Study. JMIR Form Res. 2024;8:e46222.
102. Ju C, Shi R, Yao L, Ye X, Jia M, Han J, et al. Effect of peer support on diabetes distress: a cluster randomized controlled trial. Diabet Med. 2018;35(6):770–5.
103. Jung HY, Lee H, Park J. Comparison of the effects of Korean mindfulness-based stress reduction, walking, and patient education in diabetes mellitus. Nurs Health Sci. 2015;17(4):516–25.
104. Juul L, Maindal HT, Zoffmann V, Frydenberg M, Sandbaek A. Effectiveness of a training course for general practice nurses in motivation support in type 2 diabetes care: a cluster-randomised trial. PLoS One. 2014;9(5):e96683.
105. Kakoschke N, Zajac IT, Tay J, Luscombe-Marsh ND, Thompson CH, Noakes M, et al. Effects of very low-carbohydrate vs. high-carbohydrate weight loss diets on psychological health in adults with obesity and type 2 diabetes: a 2-year randomized controlled trial. Eur J Nutr. 2021;60(8):4251–62.
106. Kampling H, Köhler B, Germerott I, Haastert B, Icks A, Kulzer B, et al. An Integrated Psychosomatic Treatment Program for People with Diabetes (psy-PAD). Dtsch Arztebl Int. 2022;119(14):245–52.

107. Karlsen B, Idsoe T, Dirdal I, Rokne Hanestad B, Bru E. Effects of a group-based counselling programme on diabetes-related stress, coping, psychological well-being and metabolic control in adults with type 1 or type 2 diabetes. Patient Educ Couns. 2004;53(3):299–308.
108. Kasteleyn MJ, Vos RC, Rijken M, Schellevis FG, Rutten GE. Effectiveness of tailored support for people with Type 2 diabetes after a first acute coronary event: a multicentre randomized controlled trial (the Diacourse-ACE study). Diabet Med. 2016;33(1):125–33.
109. Katz RJ, Magee MF, Nunlee-Bland G, Johnson P, Witkin L, Nassar C, et al. PCORI Final Research Reports. Washington (DC): Patient-Centered Outcomes Research Institute (PCORI) Copyright © 2019. George Washington University. All Rights Reserved.; 2019.
110. Kavin M, Añel-Tiangco RM, Mauger DT, Gabbay RA. Development and pilot of a low-literacy diabetes education book using social marketing techniques. Diabetes Ther. 2010;1(2):93–102.
111. Kelly RC, Price H, Phiri P, Cummings M, Ali A, Patel M, et al. Delivering Biopsychosocial Health Care Within Routine Care: Spotlight-AQ Pivotal Multicenter Randomized Controlled Trial Results. J Diabetes Sci Technol. 2023:19322968231183436.
112. Kempainen S, Cutts DB, Robinson-O'Brien R, De Kesel Lofthus A, Gilbertson DT, Mino R. A Collaborative Pilot to Support Patients With Diabetes Through Tailored Food Box Home Delivery. Health Promot Pract. 2023;24(5):963–8.
113. Kempf K, Martin S. Autonomous exercise game use improves metabolic control and quality of life in type 2 diabetes patients - a randomized controlled trial. BMC Endocr Disord. 2013;13:57.
114. Kılıç A, Hudson J, Scott W, McCracken LM, Hackett RA, Hughes LD. An online acceptance, commitment, and self-compassion based treatment to decrease psychological distress in people with type 2 diabetes: A feasibility randomised-controlled trial. Internet Interv. 2023;33:100658.
115. Kleinman NJ, Shah A, Shah S, Phatak S, Viswanathan V. Improved Medication Adherence and Frequency of Blood Glucose Self-Testing Using an m-Health Platform Versus Usual Care in a Multisite Randomized Clinical Trial Among People with Type 2 Diabetes in India. Telemedicine and e-Health. 2017;23(9):733–40.
116. Kunik ME, Evans TL, Christie IC, True G, Bradford A, Vasudevan MM, et al. The impact of veteran support and resources for diabetes (iNSPiRED) on diabetes distress: Results from a randomized, parallel-group trial. Gen Hosp Psychiatry. 2023;85:55–62.
117. Kwan BM, Dickinson LM, Dailey-Vail J, Glasgow RE, Gritz RM, Gurfinkel D, et al. Comparative Effectiveness of Patient-Driven versus Standardized Diabetes Shared Medical Appointments: A Pragmatic Cluster Randomized Trial. J Gen Intern Med. 2024.
118. Lamers F, Jonkers CC, Bosma H, Knottnerus JA, van Eijk JT. Treating depression in diabetes patients: does a nurse-administered minimal psychological intervention affect diabetes-specific quality of life and glycaemic control? A randomized controlled trial. J Adv Nurs. 2011;67(4):788–99.
119. Lamptey R, Amoakoh-Coleman M, Barker MM, Iddi S, Hadjiconstantinou M, Davies M, et al. Change in glycaemic control with structured diabetes self-management education in urban low-resource settings: multicentre randomised trial of effectiveness. BMC Health Services Research. 2023;23(1):199.
120. Laurberg T, Schougaard LMV, Hjollund NHI, Lomborg KE, Hansen TK, Jensen AL. Randomized controlled study to evaluate the impact of flexible patient-controlled visits in people with type 1 diabetes: The DiabetesFlex Trial. Diabet Med. 2022;39(5):e14791.

121. Lee DY, Yoo SH, Min KP, Park CY. Effect of Voluntary Participation on Mobile Health Care in Diabetes Management: Randomized Controlled Open-Label Trial. JMIR Mhealth Uhealth. 2020;8(9):e19153.
122. Lee Tara TM, Collett C, Bergford S, Hartnell S, Scott Eleanor M, Lindsay Robert S, et al. Automated Insulin Delivery in Women with Pregnancy Complicated by Type 1 Diabetes. New England Journal of Medicine. 2023;389(17):1566–78.
123. Leelarathna L, Wilmot E, Evans M, Neupane S, Rayman G, Lumley S, et al. FLASH GLUCOSE MONITORING WITH THE FREESTYLE LIBRE 2: RESULTS FROM THE FLASH-UK RANDOMISED CONTROLLED TRIAL (ON BEHALF OF THE STUDY GROUP). Diabetes technology & therapeutics. 2022;24(SUPPL 1):A21.
124. Lerman I, López-Ponce A, Villa AR, Escobedo M, Caballero EA, Velasco ML, et al. [Pilot study of two different strategies to reinforce self care behaviors and treatment compliance among type 2 diabetes patients from low income strata]. Gac Med Mex. 2009;145(1):15–9.
125. Li F, Yao P, Hsue C, Xu J, Lou Q. Impact of "Conversation Maps" on diabetes distress and self-efficacy of Chinese adult patients with type 2 diabetes: a pilot study. Patient Prefer Adherence. 2016;10:901–8.
126. Li D, Elliott T, Klein G, Ur E, Tang TS. Diabetes Nurse Case Management in a Canadian Tertiary Care Setting: Results of a Randomized Controlled Trial. Can J Diabetes. 2017;41(3):297–304.
127. Li Z, Chen Q, Yan J, Liang W, Wong WCW. Effectiveness of motivational interviewing on improving Care for Patients with type 2 diabetes in China: A randomized controlled trial. BMC Health Serv Res. 2020;20(1):57.
128. Li CL, Wu YC, Kornelius E, Chen YJ, Lin YT, Hsiao HW, et al. Comparison of Different Models of Structured Self-Monitoring of Blood Glucose in Type 2 Diabetes. Diabetes Technol Ther. 2016;18(3):171–7.
129. Liu Y, Han Y, Shi J, Li R, Li S, Jin N, et al. Effect of peer education on self-management and psychological status in type 2 diabetes patients with emotional disorders. Journal of Diabetes Investigation. 2015;6(4):479–86.
130. Lum ZK, Khoo ZR, Toh WYS, Kamaldeen SAK, Shakoor A, Tsou KYK, et al. Efficacy and Safety of Use of the Fasting Algorithm for Singaporeans With Type 2 Diabetes (FAST) During Ramadan: A Prospective, Multicenter, Randomized Controlled Trial. Ann Fam Med. 2020;18(2):139–47.
131. Lutes LD, Cummings DM, Littlewood K, Solar C, Carraway M, Kirian K, et al. COMRADE: A randomized trial of an individually tailored integrated care intervention for uncontrolled type 2 diabetes with depression and/or distress in the rural southeastern US. Contemp Clin Trials. 2018;70:8–14.
132. Ma RCW, Xie F, Lim CKP, Lau ESH, Luk AOY, Ozaki R, et al. A randomized clinical trial of genetic testing and personalized risk counselling in patients with type 2 diabetes receiving integrated care -The genetic testing and patient empowerment (GEM) trial. Diabetes Res Clin Pract. 2022;189:109969.
133. MacPhail M, Mullan B, Sharpe L, MacCann C, Todd J. Using the health action process approach to predict and improve health outcomes in individuals with type 2 diabetes mellitus. Diabetes Metab Syndr Obes. 2014;7:469–79.
134. Maghsoudi Z, Razavi Z, Razavi M, Javadi M. Efficacy Of Acceptance And Commitment Therapy For Emotional Distress In The Elderly With Type 2 Diabetes: A Randomized Controlled Trial. Diabetes Metab Syndr Obes. 2019;12:2137–43.

135. Maharaj SS, Nuhu JM. Mini-trampoline rebound exercises: A 'self-care' initiative for glycated hemoglobin, body mass index and emotional distress for mildly obese females with non-insulin dependent type 2 diabetes. Diabetes Metab Syndr. 2019;13(2):1569–73.
136. Malanda UL, Bot SD, Kostense PJ, Snoek FJ, Dekker JM, Nijpels G. Effects of self-monitoring of glucose on distress and self-efficacy in people with non-insulin-treated Type 2 diabetes: a randomized controlled trial. Diabet Med. 2016;33(4):537–46.
137. Martyn-Nemeth P, Duffecy J, Quinn L, Steffen A, Baron K, Chapagai S, et al. Sleep-Opt-In: A Randomized Controlled Pilot Study to Improve Sleep and Glycemic Variability in Adults With Type 1 Diabetes. Sci Diabetes Self Manag Care. 2023;49(1):11–22.
138. McAuley SA, Lee MH, Paldus B, Vogrin S, de Bock MI, Abraham MB, et al. Six Months of Hybrid Closed-Loop Versus Manual Insulin Delivery With Fingerprick Blood Glucose Monitoring in Adults With Type 1 Diabetes: A Randomized, Controlled Trial. Diabetes Care. 2020;43(12):3024–33.
139. McAuley SA, Trawley S, Vogrin S, Ward GM, Fourlanos S, Grills CA, et al. Closed-Loop Insulin Delivery Versus Sensor-Augmented Pump Therapy in Older Adults With Type 1 Diabetes (ORACL): A Randomized, Crossover Trial. Diabetes Care. 2021;45(2):381–90.
140. McEwen MM, Pasvogel A, Murdaugh C, Hepworth J. Effects of a Family-based Diabetes Intervention on Behavioral and Biological Outcomes for Mexican American Adults. Diabetes Educ. 2017;43(3):272–85.
141. McMahon GT, Fonda SJ, Gomes HE, Alexis G, Conlin PR. A randomized comparison of online- and telephone-based care management with internet training alone in adult patients with poorly controlled type 2 diabetes. Diabetes Technol Ther. 2012;14(11):1060–7.
142. Misra R, Shawley-Brzoska S, Khan R, Kirk BO, Wen S, Sambamoorthi U. Addressing Diabetes Distress in Self-Management Programs: Results of a Randomized Feasibility Study. J Appalach Health. 2021;3(3):68–85.
143. Mitchell SE, Bragg A, De La Cruz BA, Winter MR, Reichert MJ, Laird LD, et al. Effectiveness of an Immersive Telemedicine Platform for Delivering Diabetes Medical Group Visits for African American, Black and Hispanic, or Latina Women With Uncontrolled Diabetes: The Women in Control 2.0 Noninferiority Randomized Clinical Trial. J Med Internet Res. 2023;25:e43669.
144. Mohn J, Graue M, Assmus J, Zoffmann V, Thordarson H, Peyrot M, et al. The effect of guided self-determination on self-management in persons with type 1 diabetes mellitus and HbA(1c) ≥64 mmol/mol: a group-based randomised controlled trial. BMJ Open. 2017;7(6):e013295.
145. Munshi MN, Segal AR, Suhl E, Ryan C, Sternthal A, Giusti J, et al. Assessment of barriers to improve diabetes management in older adults: a randomized controlled study. Diabetes Care. 2013;36(3):543–9.
146. Murray E, Sweeting M, Dack C, Pal K, Modrow K, Hudda M, et al. Web-based self-management support for people with type 2 diabetes (HeLP-Diabetes): randomised controlled trial in English primary care. BMJ Open. 2017;7(9):e016009.
147. Nayak A, Vakili S, Nayak K, Nikolov M, Chiu M, Sosseinheimer P, et al. Use of Voice-Based Conversational Artificial Intelligence for Basal Insulin Prescription Management Among Patients With Type 2 Diabetes: A Randomized Clinical Trial. JAMA Netw Open. 2023;6(12):e2340232.
148. Newby J, Robins L, Wilhelm K, Smith J, Fletcher T, Gillis I, et al. Web-Based Cognitive Behavior Therapy for Depression in People With Diabetes Mellitus: A Randomized Controlled Trial. J Med Internet Res. 2017;19(5):e157.

149. Ngan HY, Chong YY, Loo KM, Chien WT. Preliminary efficacy of an acceptance-based diabetes education (ACT-DE) programme for people with type 2 diabetes on diabetes distress and self-care behaviours: A pilot randomised controlled trial. Journal of Contextual Behavioral Science. 2023;30:50–60.
150. Nobis S, Lehr D, Ebert DD, Baumeister H, Snoek F, Riper H, et al. Efficacy of a web-based intervention with mobile phone support in treating depressive symptoms in adults with type 1 and type 2 diabetes: a randomized controlled trial. Diabetes Care. 2015;38(5):776–83.
151. O'Brien CL, Apputhurai P, Knowles SR, Jenkins ZM, Ski CF, Thompson DR, et al. Initial evaluation of the Optimal Health Program for people with diabetes: 12-month outcomes of a randomised controlled trial. Psychol Health. 2024;39(3):358–78.
152. Odnoletkova I, Goderis G, Nobels F, Fieuws S, Aertgeerts B, Annemans L, et al. Optimizing diabetes control in people with Type 2 diabetes through nurse-led telecoaching. Diabetic Medicine. 2016;33(6):777–85.
153. Overland J, Abousleiman J, Chronopoulos A, Leader N, Molyneaux L, Gilfillan C. Improving Self-Monitoring of Blood Glucose among Adults with Type 1 Diabetes: results of the Mobile™ Study. Diabetes therapy. 2014;5(2):557–65.
154. Pearson S, Wills K, Woods M, Warnecke E. Effects of mindfulness on psychological distress and HbA1c in people with diabetes. Mindfulness. 2018;9(5):1615–26.
155. Petrak F, Herpertz S, Albus C, Hermanns N, Hiemke C, Hiller W, et al. Cognitive Behavioral Therapy Versus Sertraline in Patients With Depression and Poorly Controlled Diabetes: The Diabetes and Depression (DAD) Study: A Randomized Controlled Multicenter Trial. Diabetes Care. 2015;38(5):767–75.
156. Peyrot M, Rubin RR, Polonsky WH. Diabetes distress and its association with clinical outcomes in patients with type 2 diabetes treated with pramlintide as an adjunct to insulin therapy. Diabetes Technol Ther. 2008;10(6):461–6.
157. Peyrot M, Rubin RR, Polonsky WH, Best JH. Patient reported outcomes in adults with type 2 diabetes on basal insulin randomized to addition of mealtime pramlintide or rapid-acting insulin analogs. Curr Med Res Opin. 2010;26(5):1047–54.
158. Piatt GA, Rodgers EA, Xue L, Zgibor JC. Integration and Utilization of Peer Leaders for Diabetes Self-Management Support: Results From Project SEED (Support, Education, and Evaluation in Diabetes). Diabetes Educ. 2018;44(4):373–82.
159. Pibernik-Okanović M, Hermanns N, Ajduković D, Kos J, Prašek M, Šekerija M, et al. Does treatment of subsyndromal depression improve depression-related and diabetes-related outcomes? A randomised controlled comparison of psychoeducation, physical exercise and enhanced treatment as usual. Trials. 2015;16:305.
160. Polonsky WH, Hessler D, Ruedy KJ, Beck RW. The Impact of Continuous Glucose Monitoring on Markers of Quality of Life in Adults With Type 1 Diabetes: Further Findings From the DIAMOND Randomized Clinical Trial. Diabetes Care. 2017;40(6):736–41.
161. Popelier M, Ciangura C, Flahault C, Louot C, Vanier A, Sachon C, et al. Évaluation de deux approches de médecine narrative, le théâtre du vécu et un atelier d’écriture, dans une prise en charge éducative intégrée de patients diabétiques de type 1 = Evaluation of two ways of narrative medicine, 'living theatre' and a written w. Éducation Thérapeutique du Patient / Therapeutic Patient Education. 2019;11(1):1–7.

162. Pouwer F, Tack CJ, Geelhoed-Duijvestijn PH, Bazelmans E, Beekman AT, Heine RJ, et al. Limited effect of screening for depression with written feedback in outpatients with diabetes mellitus: a randomised controlled trial. Diabetologia. 2011;54(4):741–8.
163. Pranata S, Shu-Fang Vivienne W, Tsae-Jyy Tiffany W, Shu-Yuan L, Bistara DN, Yeu-Hui C, et al. Effects of the tailored intervention program in lowering blood glucose levels and diabetes distress among patients with diabetes in Indonesia: a randomized controlled trial. Jurnal Ners. 2023;18(1):71–9.
164. Pratley RE, Kanapka LG, Rickels MR, Ahmann A, Aleppo G, Beck R, et al. Effect of Continuous Glucose Monitoring on Hypoglycemia in Older Adults With Type 1 Diabetes: A Randomized Clinical Trial. JAMA. 2020;323(23):2397–406.
165. Presley C, Agne A, Shelton T, Oster R, Cherrington A. Mobile-Enhanced Peer Support for African Americans with Type 2 Diabetes: a Randomized Controlled Trial. J Gen Intern Med. 2020;35(10):2889–96.
166. Pyatak EA, Carandang K, Vigen CLP, Blanchard J, Diaz J, Concha-Chavez A, et al. Occupational Therapy Intervention Improves Glycemic Control and Quality of Life Among Young Adults With Diabetes: the Resilient, Empowered, Active Living with Diabetes (REAL Diabetes) Randomized Controlled Trial. Diabetes Care. 2018;41(4):696–704.
167. Pylov D, Polonsky W, Imberg H, Holmer H, Hellman J, Wijkman M, et al. Treatment Satisfaction and Well-Being With CGM in People With T1D: An Analysis Based on the GOLD Randomized Trial. J Diabetes Sci Technol. 2023:19322968231183974.
168. Quinn CC, Shardell MD, Terrin ML, Barr EA, Ballew SH, Gruber-Baldini AL. Cluster-randomized trial of a mobile phone personalized behavioral intervention for blood glucose control. Diabetes Care. 2011;34(9):1934–42.
169. Ramallo-Fariña Y, Rivero-Santana A, García-Pérez L, García-Bello MA, Wägner AM, Gonzalez-Pacheco H, et al. Patient-reported outcome measures for knowledge transfer and behaviour modification interventions in type 2 diabetes-the INDICA study: a multiarm cluster randomised controlled trial. BMJ Open. 2021;11(12):e050804.
170. Reaney M, Zorzo EG, Golay A, Hermanns N, Cleall S, Petzinger U, et al. Impact of Conversation Map™ Education Tools Versus Regular Care on Diabetes-Related Knowledge of People With Type 2 Diabetes: A Randomized, Controlled Study. Diabetes Spectrum. 2013;26(4):236–45.
171. Reddy M, Jugnee N, El Laboudi A, Spanudakis E, Anantharaja S, Oliver N. A randomized controlled pilot study of continuous glucose monitoring and flash glucose monitoring in people with Type 1 diabetes and impaired awareness of hypoglycaemia. Diabet Med. 2018;35(4):483–90.
172. Rees G, Lamoureux EL, Nicolaou TE, Hodgson LA, Weinman J, Speight J. Feedback of personal retinal images appears to have a motivational impact in people with non-proliferative diabetic retinopathy and suboptimal HbA1c: findings of a pilot study. Diabet Med. 2013;30(9):1122–5.
173. Rees G, O'Hare F, Saeed M, Sudholz B, Sturrock BA, Xie J, et al. Problem-solving therapy for adults with diabetic retinopathy and diabetes-specific distress: a pilot randomized controlled trial. BMJ Open Diabetes Res Care. 2017;5(1):e000307.
174. Rezaeemanesh M, Solhi M, Fard Azar FE, Javaheri J, Alaghemand A. The effect of educational intervention based on Pender's health promotion model for distress management in patients with type 2 diabetes. Pakistan journal of medical and health sciences. 2020;14(3):969–75.

175. Rilstone S, Oliver N, Godsland I, Tanushi B, Thomas M, Hill N. A Randomized Controlled Trial Assessing the Impact of Continuous Glucose Monitoring with a Predictive Hypoglycemia Alert Function on Hypoglycemia in Physical Activity for People with Type 1 Diabetes (PACE). Diabetes Technol Ther. 2024;26(2):95–102.
176. Roddy MK, Spieker AJ, Nelson LA, Greevy RA, Jr., LeStourgeon LM, Bergner EM, et al. Well-being outcomes of a family-focused intervention for persons with type 2 diabetes and support persons: Main, mediated, and subgroup effects from the FAMS 2.0 RCT. Diabetes Res Clin Pract. 2023;204:110921.
177. Rondags SM, de Wit M, Twisk JW, Snoek FJ. Effectiveness of HypoAware, a Brief Partly Web-Based Psychoeducational Intervention for Adults With Type 1 and Insulin-Treated Type 2 Diabetes and Problematic Hypoglycemia: A Cluster Randomized Controlled Trial. Diabetes Care. 2016;39(12):2190–6.
178. Rondhianto, Kusnanto, Melaniani S. The effect of diabetes self-management education, based on the health belief model, on the psychosocial outcome of type 2 diabetic patients in Indonesia. Indian journal of public health research and development. 2018;9(11):1718–23.
179. Rosales CB, Denman CA, Bell ML, Cornejo E, Ingram M, Del Carmen Castro Vásquez M, et al. Meta Salud Diabetes for cardiovascular disease prevention in Mexico: a cluster-randomized behavioural clinical trial. Int J Epidemiol. 2021;50(4):1272–82.
180. Rosenbek Minet LK, Wagner L, Lønvig EM, Hjelmborg J, Henriksen JE. The effect of motivational interviewing on glycaemic control and perceived competence of diabetes self-management in patients with type 1 and type 2 diabetes mellitus after attending a group education programme: a randomised controlled trial. Diabetologia. 2011;54(7):1620–9.
181. Rosland AM, Piette JD, Trivedi R, Lee A, Stoll S, Youk AO, et al. Effectiveness of a Health Coaching Intervention for Patient-Family Dyads to Improve Outcomes Among Adults With Diabetes: A Randomized Clinical Trial. JAMA Netw Open. 2022;5(11):e2237960.
182. Ruiz de Adana MS, Alhambra-Expósito MR, Muñoz-Garach A, Gonzalez-Molero I, Colomo N, Torres-Barea I, et al. Randomized Study to Evaluate the Impact of Telemedicine Care in Patients With Type 1 Diabetes With Multiple Doses of Insulin and Suboptimal HbA1c in Andalusia (Spain): PLATEDIAN Study. Diabetes Care. 2019;43(2):337–42.
183. Russell-Jones D, Dauchy A, Delgado E, Dimitriadis G, Frandsen HA, Popescu L, et al. Take Control: A randomized trial evaluating the efficacy and safety of self- versus physician-managed titration of insulin glargine 300 U/mL in patients with uncontrolled type 2 diabetes. Diabetes Obes Metab. 2019;21(7):1615–24.
184. Rygg LØ, Rise MB, Grønning K, Steinsbekk A. Efficacy of ongoing group based diabetes self-management education for patients with type 2 diabetes mellitus. A randomised controlled trial. Patient Education and Counseling. 2012;86(1):98–105.
185. Safford MM, Andreae S, Cherrington AL, Martin MY, Halanych J, Lewis M, et al. Peer Coaches to Improve Diabetes Outcomes in Rural Alabama: A Cluster Randomized Trial. Ann Fam Med. 2015;13 Suppl 1(Suppl 1):S18–S26.
186. Sato S, Tajiri Y, Shimono D, Sumiyoshi S, Futata T. Changes in psychological behavior accompanied by the short-term usage of flash glucose monitoring for newly diagnosed type 2 diabetes mellitus. Therapeutic research. 2020;41(7):577–86.

187. Schmitt A, Kulzer B, Reimer A, Herder C, Roden M, Haak T, et al. Evaluation of a Stepped Care Approach to Manage Depression and Diabetes Distress in Patients with Type 1 Diabetes and Type 2 Diabetes: Results of a Randomized Controlled Trial (ECCE HOMO Study). Psychother Psychosom. 2022;91(2):107–22.
188. Schneider-Utaka AK, Hanes S, Boughton CK, Hartnell S, Thabit H, Mubita WM, et al. Patient-reported outcomes for older adults on CamAPS FX closed loop system. Diabet Med. 2023;40(9):e15126.
189. Schroevers MJ, Tovote KA, Keers JC, Links TP, Sanderman R, Fleer J. Individual mindfulness-based cognitive therapy for people with diabetes: A pilot randomized controlled trial. Mindfulness. 2015;6(1):99–110.
190. Secher AL, Pedersen-Bjergaard U, Svendsen OL, Gade-Rasmussen B, Almdal T, Raimond L, et al. Flash glucose monitoring and automated bolus calculation in type 1 diabetes treated with multiple daily insulin injections: a 26 week randomised, controlled, multicentre trial. Diabetologia. 2021;64(12):2713–24.
191. Seligman HK, Smith M, Rosenmoss S, Marshall MB, Waxman E. Comprehensive Diabetes Self-Management Support From Food Banks: A Randomized Controlled Trial. Am J Public Health. 2018;108(9):1227–34.
192. Shibayama T, Kobayashi K, Takano A, Kadowaki T, Kazuma K. Effectiveness of lifestyle counseling by certified expert nurse of Japan for non-insulin-treated diabetic outpatients: a 1-year randomized controlled trial. Diabetes Res Clin Pract. 2007;76(2):265–8.
193. Shukla R, Gupta M, Agarwal N, Bajpai A. Mindfulness Meditation as Adjunctive Therapy to Improve the Glycemic Care and Quality of Life in Patients with Type 1 Diabetes. Med Sci (Basel). 2021;9(2).
194. Siaw MYL, Ko Y, Malone DC, Tsou KYK, Lew YJ, Foo D, et al. Impact of pharmacist-involved collaborative care on the clinical, humanistic and cost outcomes of high-risk patients with type 2 diabetes (IMPACT): a randomized controlled trial. J Clin Pharm Ther. 2017;42(4):475–82.
195. Sigurdardottir AK, Benediktsson R, Jonsdottir H. Instruments to tailor care of people with type 2 diabetes. J Adv Nurs. 2009;65(10):2118–30.
196. Siminerio L, Ruppert KM, Gabbay RA. Who can provide diabetes self-management support in primary care? Findings from a randomized controlled trial. Diabetes Educ. 2013;39(5):705–13.
197. Simmons D, Prevost AT, Bunn C, Holman D, Parker RA, Cohn S, et al. Impact of community based peer support in type 2 diabetes: a cluster randomised controlled trial of individual and/or group approaches. PLoS One. 2015;10(3):e0120277.
198. Simonson DC, Halperin F, Foster K, Vernon A, Goldfine AB. Clinical and Patient-Centered Outcomes in Obese Patients With Type 2 Diabetes 3 Years After Randomization to Roux-en-Y Gastric Bypass Surgery Versus Intensive Lifestyle Management: The SLIMM-T2D Study. Diabetes Care. 2018;41(4):670–9.
199. Simson U, Nawarotzky U, Friese G, Porck W, Schottenfeld-Naor Y, Hahn S, et al. Psychotherapy intervention to reduce depressive symptoms in patients with diabetic foot syndrome. Diabet Med. 2008;25(2):206–12.
200. Skelly AH, Carlson J, Leeman J, Soward A, Burns D. Controlled trial of nursing interventions to improve health outcomes of older African American women with type 2 diabetes. Nurs Res. 2009;58(6):410–8.

201. Snoek FJ, van der Ven NC, Twisk JW, Hogenelst MH, Tromp-Wever AM, van der Ploeg HM, et al. Cognitive behavioural therapy (CBT) compared with blood glucose awareness training (BGAT) in poorly controlled Type 1 diabetic patients: long-term effects on HbA moderated by depression. A randomized controlled trial. Diabet Med. 2008;25(11):1337–42.
202. Spencer MS, Kieffer EC, Sinco B, Piatt G, Palmisano G, Hawkins J, et al. Outcomes at 18 Months From a Community Health Worker and Peer Leader Diabetes Self-Management Program for Latino Adults. Diabetes Care. 2018;41(7):1414–22.
203. Spencer M, Rosland A-M, Kieffer E, Sinco B, Valerio M, Palmisano G, et al. Effectiveness of a Community Health Worker Intervention Among African American and Latino Adults With Type 2 Diabetes: A Randomized Controlled Trial. American journal of public health. 2011;101:2253–60.
204. Sperl-Hillen J, Beaton S, Fernandes O, Von Worley A, Vazquez-Benitez G, Hanson A, et al. Are benefits from diabetes self-management education sustained? Am J Manag Care. 2013;19(2):104–12.
205. Sturt JA, Whitlock S, Fox C, Hearnshaw H, Farmer AJ, Wakelin M, et al. Effects of the Diabetes Manual 1:1 structured education in primary care. Diabet Med. 2008;25(6):722–31.
206. Sunuwar DR, Nayaju S, Dhungana RR, Karki K, Singh Pradhan PM, Poudel P, et al. Effectiveness of a dietician-led intervention in reducing glycated haemoglobin among people with type 2 diabetes in Nepal: a single centre, open-label, randomised controlled trial. Lancet Reg Health Southeast Asia. 2023;18:100285.
207. Swoboda CM, Miller CK, Wills CE. Impact of a goal setting and decision support telephone coaching intervention on diet, psychosocial, and decision outcomes among people with type 2 diabetes. Patient Educ Couns. 2017;100(7):1367–73.
208. Tang TS, Funnell MM, Sinco B, Spencer MS, Heisler M. Peer-Led, Empowerment-Based Approach to Self-Management Efforts in Diabetes (PLEASED): A Randomized Controlled Trial in an African American Community. Ann Fam Med. 2015;13 Suppl 1(Suppl 1):S27–S35.
209. Tang TS, Afshar R, Elliott T, Kong J, Gill S. From clinic to community: A randomized controlled trial of a peer support model for adults with type 2 diabetes from specialty care settings in British Columbia. Diabet Med. 2022;39(11):e14931.
210. Tang PC, Overhage JM, Chan AS, Brown NL, Aghighi B, Entwistle MP, et al. Online disease management of diabetes: Engaging and Motivating Patients Online With Enhanced Resources-Diabetes (EMPOWER-D), a randomized controlled trial. Journal of the American Medical Informatics Association. 2012;20(3):526–34.
211. Tang TS, Funnell M, Sinco B, Piatt G, Palmisano G, Spencer MS, et al. Comparative effectiveness of peer leaders and community health workers in diabetes self-management support: results of a randomized controlled trial. Diabetes Care. 2014;37(6):1525–34.
212. Ischia Study G. Prevention of hypoglycemia by intermittent-scanning continuous glucose monitoring device combined with structured education in patients with type 1 diabetes mellitus: A randomized, crossover trial. Diabetes Res Clin Pract. 2023;195:110147.
213. Tovilla-Zárate CA, Pérez-Mandujano A, Ramírez-González IR, Fresan A, Suarez-Mendez S, Martínez-Villaseñor E, et al. Vortioxetine versus sertraline in metabolic control, distress and depression in Mexican patients with type 2 diabetes. Ann Transl Med. 2019;7(22):656.

214. Tovote KA, Schroevers MJ, Snippe E, Sanderman R, Links TP, Emmelkamp PM, et al. Long-term effects of individual mindfulness-based cognitive therapy and cognitive behavior therapy for depressive symptoms in patients with diabetes: a randomized trial. Psychotherapy and psychosomatics. 2015;84(3):186–7.
215. Trevisan DD, São-João T, Cornélio M, Jannuzzi F, de Sousa MR, Rodrigues R, et al. Effect of an 'implementation intention' intervention on adherence to oral anti-diabetic medication in Brazilians with type 2 diabetes. Patient Educ Couns. 2020;103(3):582–8.
216. Trief PM, Teresi JA, Izquierdo R, Morin PC, Goland R, Field L, et al. Psychosocial outcomes of telemedicine case management for elderly patients with diabetes: the randomized IDEATel trial. Diabetes Care. 2007;30(5):1266–8.
217. Trief PM, Fisher L, Sandberg J, Cibula DA, Dimmock J, Hessler DM, et al. Health and Psychosocial Outcomes of a Telephonic Couples Behavior Change Intervention in Patients With Poorly Controlled Type 2 Diabetes: A Randomized Clinical Trial. Diabetes Care. 2016;39(12):2165–73.
218. Uduku C, Pendolino V, Jugnee N, Oliver N, Fothergill R, Reddy M. Real-time continuous glucose monitoring immediately after severe hypoglycaemia requiring emergency medical services: A randomised controlled trial. Diabet Med. 2023;40(7):e15100.
219. van Bastelaar KM, Pouwer F, Cuijpers P, Riper H, Snoek FJ. Web-based depression treatment for type 1 and type 2 diabetic patients: a randomized, controlled trial. Diabetes Care. 2011;34(2):320–5.
220. van der Wulp I, de Leeuw JR, Gorter KJ, Rutten GE. Effectiveness of peer-led self-management coaching for patients recently diagnosed with Type 2 diabetes mellitus in primary care: a randomized controlled trial. Diabet Med. 2012;29(10):e390–e7.
221. van Son J, Nyklíček I, Pop VJ, Blonk MC, Erdtsieck RJ, Pouwer F. Mindfulness-based cognitive therapy for people with diabetes and emotional problems: long-term follow-up findings from the DiaMind randomized controlled trial. J Psychosom Res. 2014;77(1):81–4.
222. van Beers CAJ, DeVries JH, Kleijer SJ, Smits MM, Geelhoed-Duijvestijn PH, Kramer MHH, et al. Continuous glucose monitoring for patients with type 1 diabetes and impaired awareness of hypoglycaemia (IN CONTROL): a randomised, open-label, crossover trial. The Lancet Diabetes & Endocrinology. 2016;4(11):893–902.
223. van den Donk M, Gorter KJ, Rutten GE. No negative effects of a multi-factorial, intensified treatment on self-reported health status, treatment satisfaction, and diabetes-related distress in screen-detected type 2 diabetes patients. The ADDITION-Netherlands study. Qual Life Res. 2010;19(4):509–13.
224. van Dijk-de Vries A, van Bokhoven MA, Winkens B, Terluin B, Knottnerus JA, van der Weijden T, et al. Lessons learnt from a cluster-randomised trial evaluating the effectiveness of Self-Management Support (SMS) delivered by practice nurses in routine diabetes care. BMJ Open. 2015;5(6):e007014.
225. van Puffelen AL, Rijken M, Heijmans M, Nijpels G, Schellevis FG. Effectiveness of a self-management support program for type 2 diabetes patients in the first years of illness: Results from a randomized controlled trial. PLoS One. 2019;14(6):e0218242.
226. Vaughan EM, Cully JA, Petersen NJ, Hundt NE, Kunik ME, Zeno DD, et al. Testing the Impact of a Collaborative, Goal-Setting, and Behavioral Telehealth Intervention on Diabetes Distress: A Randomized Clinical Trial. Telemed J E Health. 2022;28(1):84–92.

227. Wagner JA, Bermudez-Millan A, Damio G, Segura-Perez S, Chhabra J, Vergara C, et al. A randomized, controlled trial of a stress management intervention for Latinos with type 2 diabetes delivered by community health workers: Outcomes for psychological wellbeing, glycemic control, and cortisol. Diabetes Res Clin Pract. 2016;120:162–70.
228. Watson NA, Dyer KA, Buckley JD, Brinkworth GD, Coates AM, Parfitt G, et al. Comparison of two low-fat diets, differing in protein and carbohydrate, on psychological wellbeing in adults with obesity and type 2 diabetes: a randomised clinical trial. Nutr J. 2018;17(1):62.
229. Weinger K, Beverly EA, Lee Y, Sitnokov L, Ganda OP, Caballero AE. The effect of a structured behavioral intervention on poorly controlled diabetes: a randomized controlled trial. Arch Intern Med. 2011;171(22):1990–9.
230. Weissberg-Benchell J, Hessler D, Fisher L, Russell SJ, Polonsky WH. Impact of an Automated Bihormonal Delivery System on Psychosocial Outcomes in Adults with Type 1 Diabetes. Diabetes Technol Ther. 2017;19(12):723–9.
231. Weissberg-Benchell J, Vesco AT, Shapiro J, Calhoun P, Damiano ER, Russell SJ, et al. Psychosocial Impact of the Insulin-Only iLet Bionic Pancreas for Adults, Youth, and Caregivers of Youth with Type 1 Diabetes. Diabetes Technology & Therapeutics. 2023;25(10):705–17.
232. Welch G, Zagarins SE, Feinberg RG, Garb JL. Motivational interviewing delivered by diabetes educators: does it improve blood glucose control among poorly controlled type 2 diabetes patients? Diabetes Res Clin Pract. 2011;91(1):54–60.
233. Welch G, Zagarins SE, Santiago-Kelly P, Rodriguez Z, Bursell SE, Rosal MC, et al. An internet-based diabetes management platform improves team care and outcomes in an urban Latino population. Diabetes Care. 2015;38(4):561–7.
234. Welch G, Allen NA, Zagarins SE, Stamp KD, Bursell SE, Kedziora RJ. Comprehensive diabetes management program for poorly controlled Hispanic type 2 patients at a community health center. Diabetes Educ. 2011;37(5):680–8.
235. Wexler DJ, Chang Y, Levy DE, Porneala B, McCarthy J, Rodriguez Romero A, et al. Results of a 2-year lifestyle intervention for type 2 diabetes: the Reach Ahead for Lifestyle and Health-Diabetes randomized controlled trial. Obesity. 2022;30(10):1938–50.
236. Whittemore R, Melkus GD, Sullivan A, Grey M. A nurse-coaching intervention for women with type 2 diabetes. Diabetes Educ. 2004;30(5):795–804.
237. Wijk I, Amsberg S, Johansson UB, Livheim F, Toft E, Anderbro T. Impact of an Acceptance and Commitment Therapy programme on HbA1c, self-management and psychosocial factors in adults with type 1 diabetes and elevated HbA1c levels: a randomised controlled trial. BMJ Open. 2023;13(12):e072061.
238. Winkelman JW, Wipper B, Zackon J, Hoeppner BB. Lack of Efficacy of Suvorexant in People with Insomnia and Poorly Controlled Type 2 Diabetes. Nat Sci Sleep. 2023;15:1117–28.
239. Winkley K, Sorsa T, Tian Q, Reece I, Fitzgerald C, Chamley M, et al. The diabetes insulin self-management education (DIME) intervention for people with type 2 diabetes starting insulin: a pilot feasibility randomised controlled trial. Pilot Feasibility Stud. 2023;9(1):89.
240. Witkow S, Liberty IF, Goloub I, Kaminsky M, Otto O, Rabia Y, et al. Simplifying carb counting: A randomized controlled study - Feasibility and efficacy of an individualized, simple, patient-centred carb counting tool. Endocrinol Diabetes Metab. 2023;6(2):e411.

241. Woodard L, Amspoker AB, Hundt NE, Gordon HS, Hertz B, Odom E, et al. Comparison of Collaborative Goal Setting With Enhanced Education for Managing Diabetes-Associated Distress and Hemoglobin A1c Levels: A Randomized Clinical Trial. JAMA Netw Open. 2022;5(5):e229975.
242. Wycherley TP, Clifton PM, Noakes M, Brinkworth GD. Weight loss on a structured hypocaloric diet with or without exercise improves emotional distress and quality of life in overweight and obese patients with type 2 diabetes. J Diabetes Investig. 2014;5(1):94–8.
243. Yanhong GE, Lihong YU, Yue Z, Xuan LI, Lihui Z, Shuo K, et al. Curative effects of mindfulness training based on "meta-awareness" combined with intermittent pneumatic compression therapy on TcPO2,distress,mindful attenion awareness and blood glucose in diabetic patients with lower extremity arterial disease. Chinese Nursing Research. 2020;34(7):574–9.
244. Yu C, Choi D, Bruno BA, Thorpe KE, Straus SE, Cantarutti P, et al. Impact of MyDiabetesPlan, a Web-Based Patient Decision Aid on Decisional Conflict, Diabetes Distress, Quality of Life, and Chronic Illness Care in Patients With Diabetes: Cluster Randomized Controlled Trial. J Med Internet Res. 2020;22(9):e16984.
245. Zhang Q, Zhang Y, Long T, Wu Y, Li M. Effects of nudge strategy-based dietary education intervention in patients with type 2 diabetes mellitus: A cluster randomized controlled trial. Diabetes Metab. 2024;50(5):101563.
246. Zheng F, Liu S, Liu Y, Deng L. Effects of an Outpatient Diabetes Self-Management Education on Patients with Type 2 Diabetes in China: A Randomized Controlled Trial. J Diabetes Res. 2019;2019:1073131.
247. Zoffmann V, Lauritzen T. Guided self-determination improves life skills with Type 1 diabetes and A1C in randomized controlled trial. Patient Education and Counseling. 2006;64(1):78–86.
248. Zoffmann V, Vistisen D, Due-Christensen M. Flexible guided self-determination intervention for younger adults with poorly controlled Type 1 diabetes, decreased HbA1c and psychosocial distress in women but not in men: a real-life RCT. Diabet Med. 2015;32(9):1239–46.

## 4. Risk of bias assessment

**Intention-to-treat**

| Study ID | D1 | D2 | D3 | D4 | D5 | Overall |
|---|---|---|---|---|---|---|
| Abbas 2023 | + | - | + | ! | ! | - |
| Amsberg 2009 | + | + | + | ! | ! | ! |
| Anderson 2009 | + | + | - | ! | ! | - |
| Baldwin 2020 | + | + | + | ! | + | ! |
| Bendig 2021 | + | + | + | ! | ! | ! |
| Callan 2022 | ! | - | - | ! | - | - |
| Carreira 2023 | + | - | - | ! | - | - |
| Cheng 2021 | + | + | + | ! | - | - |
| Chiu 2016 | ! | - | + | ! | - | - |
| Coccaro 2022 | ! | - | ! | ! | - | - |
| Cohn 2014 | ! | ! | + | ! | - | - |
| Crawford 2019 | ! | + | + | ! | + | ! |
| de Groot 2019 | + | - | + | ! | + | - |
| Dennick 2015 | + | + | ! | ! | ! | ! |
| DiNardo 2022 | + | + | + | ! | ! | ! |
| Fisher 2013 | + | - | + | ! | - | - |
| Friis 2016 | ! | + | + | ! | ! | ! |
| Gabbay 2013 | ! | - | + | ! | ! | - |
| Griva 2019 | + | + | + | ! | ! | ! |
| Guo 2022 | + | + | + | ! | - | - |
| Heinrich 2010 | + | + | + | + | ! | ! |
| Hermanns 2015 | ! | + | ! | ! | ! | ! |
| Hoyo 2022 | + | + | + | ! | ! | ! |
| Fisher 2018 | + | - | + | ! | - | - |
| Ismail 2018 | + | + | + | ! | ! | ! |
| Juul 2014 | + | + | + | ! | + | ! |
| Kampling 2022 | + | + | + | ! | ! | ! |
| Kasteleyn 2016 | + | + | + | ! | + | ! |
| Kilic 2023 | + | - | ! | ! | - | - |
| Lamers 2011 | + | + | + | ! | ! | ! |
| Li 2020 | ! | + | - | ! | - | - |
| Lutes 2018 | ! | + | ! | ! | - | - |
| Maghsoudi 2019 | + | - | + | ! | - | - |
| Mohn 2017 | ! | - | + | ! | ! | - |
| Newby 2017 | + | + | + | ! | + | ! |
| Pearson 2018 | ! | + | + | ! | ! | ! |
| Petrak 2015 | + | + | - | ! | + | - |
| Popelier 2019 | ! | - | - | ! | ! | - |
| Rees 2017 | ! | - | - | ! | - | - |

+ Low risk
! Some concerns
- High risk

D1 Randomisation process
D2 Deviations from the intended interventions
D3 Missing outcome data
D4 Measurement of the outcome
D5 Selection of the reported result

| | | | | | | |
|---|---|---|---|---|---|---|
| Roddy 2023 | + | + | + | ! | + | ! |
| RosenbekMinet 2011 | + | + | + | ! | ! | ! |
| Schmitt 2022 | + | + | + | ! | ! | ! |
| Schroevers 2015 | + | + | + | ! | ! | ! |
| Shukla 2021 | + | - | - | ! | ! | - |
| Simson 2008 | ! | - | + | ! | ! | - |
| Snoek 2008 | + | + | + | ! | ! | ! |
| Spencer 2011 | + | ! | + | ! | + | ! |
| Trevisan 2020 | + | - | + | ! | - | - |
| Trief 2016 | + | + | + | ! | + | ! |
| van Bastelaar 2011 | + | + | + | ! | ! | ! |
| van Son 2013 | + | + | + | ! | + | ! |
| Wijk 2023 | + | + | + | ! | + | ! |
| Yahong 2020 | + | - | - | ! | - | - |
| Zoffmann 2006 | - | - | + | ! | ! | - |
| Alessi 2022 | + | + | + | ! | ! | ! |
| Anjali 2023 | ! | - | + | ! | - | - |
| Bae 2024 | ! | - | + | ! | ! | - |
| Baig 2024 | ! | + | + | ! | + | ! |
| Basak Cinar 2014 | ! | - | + | ! | ! | - |
| Binesh 2023 | + | + | + | ! | - | - |
| Bisno 2022 | ! | + | + | ! | ! | ! |
| Bond 2010 | + | + | + | ! | ! | ! |
| Brennan 2021 | + | + | + | ! | ! | ! |
| Crowley 2022 | + | + | + | ! | ! | ! |
| Davies 2008 | + | ! | + | ! | + | ! |
| D'Eramo Melkus 2010 | + | + | + | ! | ! | ! |
| Dobson 2018 | + | + | + | ! | - | - |
| Heisler 2014 | + | - | + | ! | + | - |
| Hermanns 2007 | ! | - | + | ! | ! | - |
| Hermanns 2012 | + | - | + | ! | ! | - |
| Hermanns 2013 | + | - | + | ! | ! | - |
| Hermanns 2017 | + | - | + | ! | ! | - |
| Hessler 2022 | ! | - | + | ! | ! | - |
| Hilmarsdóttir 2021 | + | + | ! | ! | ! | ! |
| Jiang 2021 | + | + | + | ! | ! | ! |
| Jung 2015 | + | - | + | ! | ! | - |
| Karlsen 2004 | ! | ! | + | ! | ! | ! |
| Kleinman 2017 | ! | ! | - | + | ! | - |
| Lamptey 2023 | + | - | + | ! | ! | - |
| Lerman 2009 | ! | - | + | ! | ! | - |

| | | | | | | |
|---|---|---|---|---|---|---|
| Li 2016 | + | - | + | ! | + | - |
| Li 2017 | + | + | ! | ! | + | ! |
| Liu 2015 | + | - | - | ! | ! | - |
| McEwen 2017 | ! | - | ! | ! | - | - |
| Misra 2021 | ! | - | - | ! | - | - |
| Munshi 2013 | + | + | + | ! | ! | ! |
| Murray 2017 | + | + | + | ! | + | ! |
| O'Brien 2024 | + | + | + | ! | ! | ! |
| Odnoletkova 2016 | + | ! | + | ! | ! | ! |
| Payatak 2018 | + | + | + | ! | + | ! |
| Pranata 2023 | + | - | ! | ! | - | - |
| Quinn 2011 | + | + | + | ! | + | ! |
| Ramallo-Farina 2021 | + | + | + | ! | ! | ! |
| Reaney 2012 | ! | + | + | ! | + | ! |
| Rezaeemanesh 2020 | ! | - | - | ! | ! | - |
| Rondags 2016 | + | + | + | ! | ! | ! |
| Rondhianto 2018 | ! | + | + | ! | ! | ! |
| Rosales 2021 | + | + | + | ! | ! | ! |
| Rosland 2022 | + | + | ! | ! | + | ! |
| Rygg 2012 | + | + | + | ! | ! | ! |
| Safford 2015 | + | ! | + | ! | ! | ! |
| Shibayama 2007 | ! | - | + | ! | ! | - |
| Sigmundottir 2009 | + | + | + | ! | - | - |
| Skelly 2009 | ! | + | + | ! | ! | ! |
| Spencer 2013 | + | ! | + | ! | + | ! |
| Spencer 2018 | + | + | + | ! | ! | ! |
| Swoboda 2017 | + | + | + | ! | ! | ! |
| Tang 2022 | + | - | + | ! | - | - |
| van Puffelen 2019 | + | + | + | ! | ! | ! |
| vanDijk-deVries 2015 | + | - | + | ! | + | - |
| Wagner 2016 | + | + | + | ! | ! | ! |
| Welch 2015 | ! | + | + | ! | ! | ! |
| Whittemore 2004 | - | + | + | ! | ! | - |
| Winkley 2023 | + | + | + | ! | ! | ! |
| Woodard 2022 | + | + | + | ! | + | ! |
| Zhang 2024 | + | + | + | ! | ! | ! |
| Boscari 2022 | + | + | + | ! | ! | ! |
| Chan 2014 | + | + | + | ! | ! | ! |
| Clark 2020 | + | + | ! | ! | ! | ! |
| Dale 2009 | ! | + | - | ! | + | - |
| Ehrmann 2016 | + | + | + | ! | ! | ! |

| | | | | | | |
|---|---|---|---|---|---|---|
| Fisher 2011 | ! | + | + | ! | - | - |
| Furler 2020 | + | + | + | ! | + | ! |
| Gagliardino 2013 | - | + | + | + | ! | - |
| GrilloMde 2016 | + | + | ! | ! | - | - |
| Haak 2016 | ! | - | ! | ! | - | - |
| Heisler 2010 | + | + | + | ! | ! | ! |
| Hermanides 2011 | + | + | + | ! | ! | ! |
| Hermanns 2009 | ! | ! | + | ! | ! | ! |
| Ho 2020 | + | ! | + | + | + | ! |
| Ing 2016 | + | + | + | ! | ! | ! |
| Ju 2018 | + | - | - | ! | ! | - |
| Kavin 2010 | + | - | + | ! | ! | - |
| Kunik 2023 | + | + | + | ! | - | - |
| Malanda 2016 | + | + | + | ! | + | ! |
| McAuley 2021 | + | ! | + | ! | + | ! |
| McAuly 2020 | + | + | + | ! | + | ! |
| Piatt 2018 | - | + | + | ! | - | - |
| Polonsky 2017 | + | + | + | ! | - | - |
| Pratley 2020 | + | + | + | ! | + | ! |
| Pylov 2023 | + | + | + | ! | ! | ! |
| Sato 2020 | + | ! | + | ! | ! | ! |
| Schneider-Utaka 2023 | ! | - | + | ! | - | - |
| Siminero 2013 | ! | + | + | ! | + | ! |
| Simmons 2015 | + | + | + | ! | ! | ! |
| Sperl-Hillen 2013 | + | + | ! | + | + | ! |
| Sturt 2008 | + | + | + | ! | ! | ! |
| Tang 2012 | + | + | + | + | - | - |
| Tang 2014 | + | + | ! | ! | ! | ! |
| van Beers 2016 | + | + | + | ! | ! | ! |
| van der Wulp 2012 | + | + | + | ! | ! | ! |
| Weissberg-Benchell 2017 | ! | - | + | ! | - | - |
| Zheng 2019 | + | - | - | ! | ! | - |
| Aronson 2023 | ! | - | + | ! | ! | - |
| Bolinder 2016 | - | - | - | ! | ! | - |
| Ischia group 2023 | ! | - | + | ! | ! | - |
| Leelarathna 2022 | + | + | + | ! | + | ! |
| Secher 2021 | + | + | + | ! | ! | ! |
| Rilstone 2024 | + | - | + | ! | ! | - |
| Reddy 2018 | + | + | + | ! | ! | ! |
| Beck 2017 | + | + | + | ! | - | - |
| Benhalima 2023 | + | + | + | ! | ! | ! |

| | | | | | | |
|---|---|---|---|---|---|---|
| Lee 2023 | + | + | + | ! | ! | ! |
| Weissberg-Benchell 2023 | + | + | + | ! | ! | ! |
| Welch 2011 | + | + | ! | ! | ! | ! |

## 5. Sensitivity and subgroup analyses

### Psychological interventions

Psychological interventions compared with usual care for type 1 diabetes

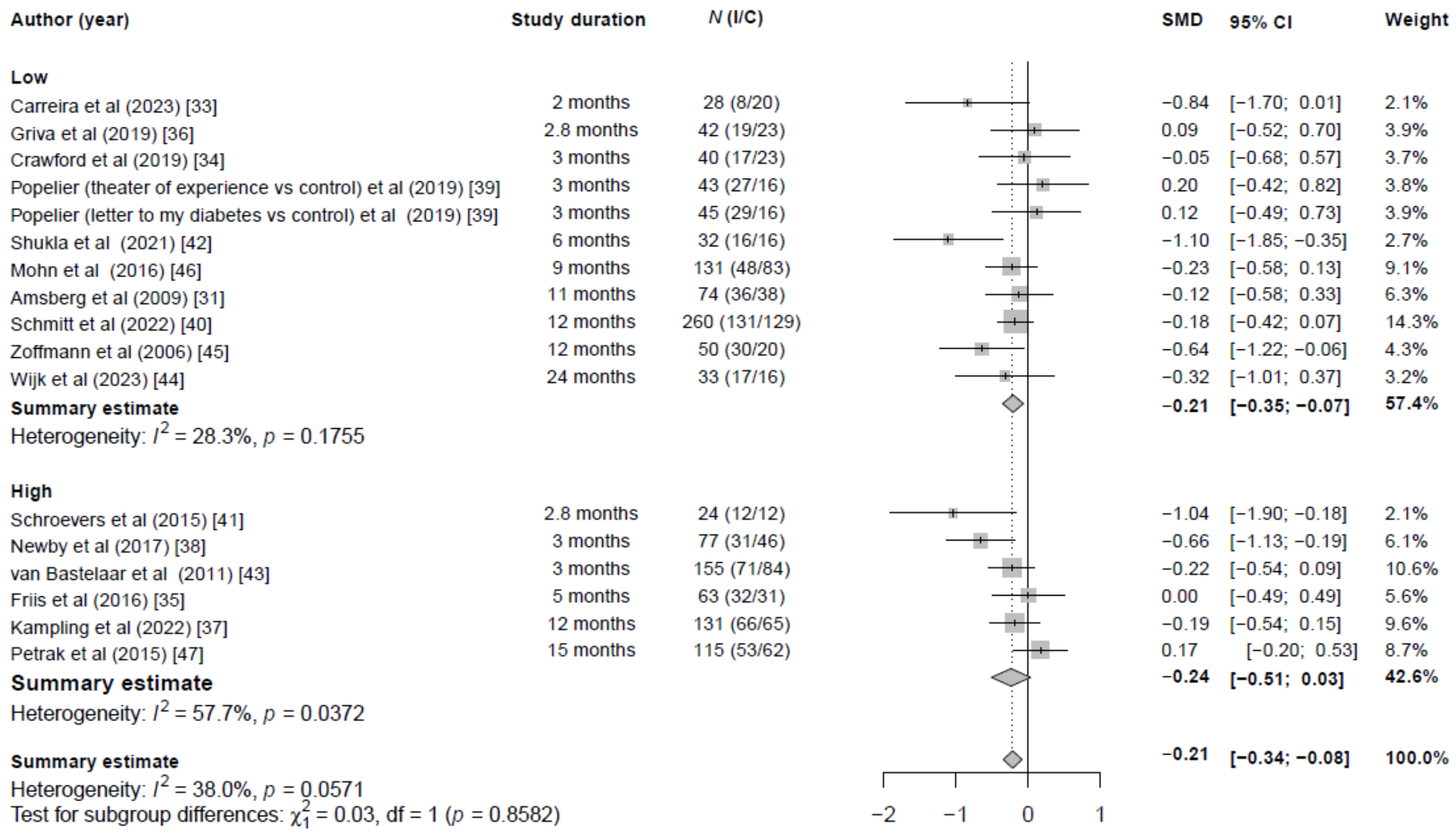


Figure 3c: SMD subgroup analysis for psychological interventions compared with usual care for type 1 diabetes stratified by baseline distress level. C, control; I, intervention

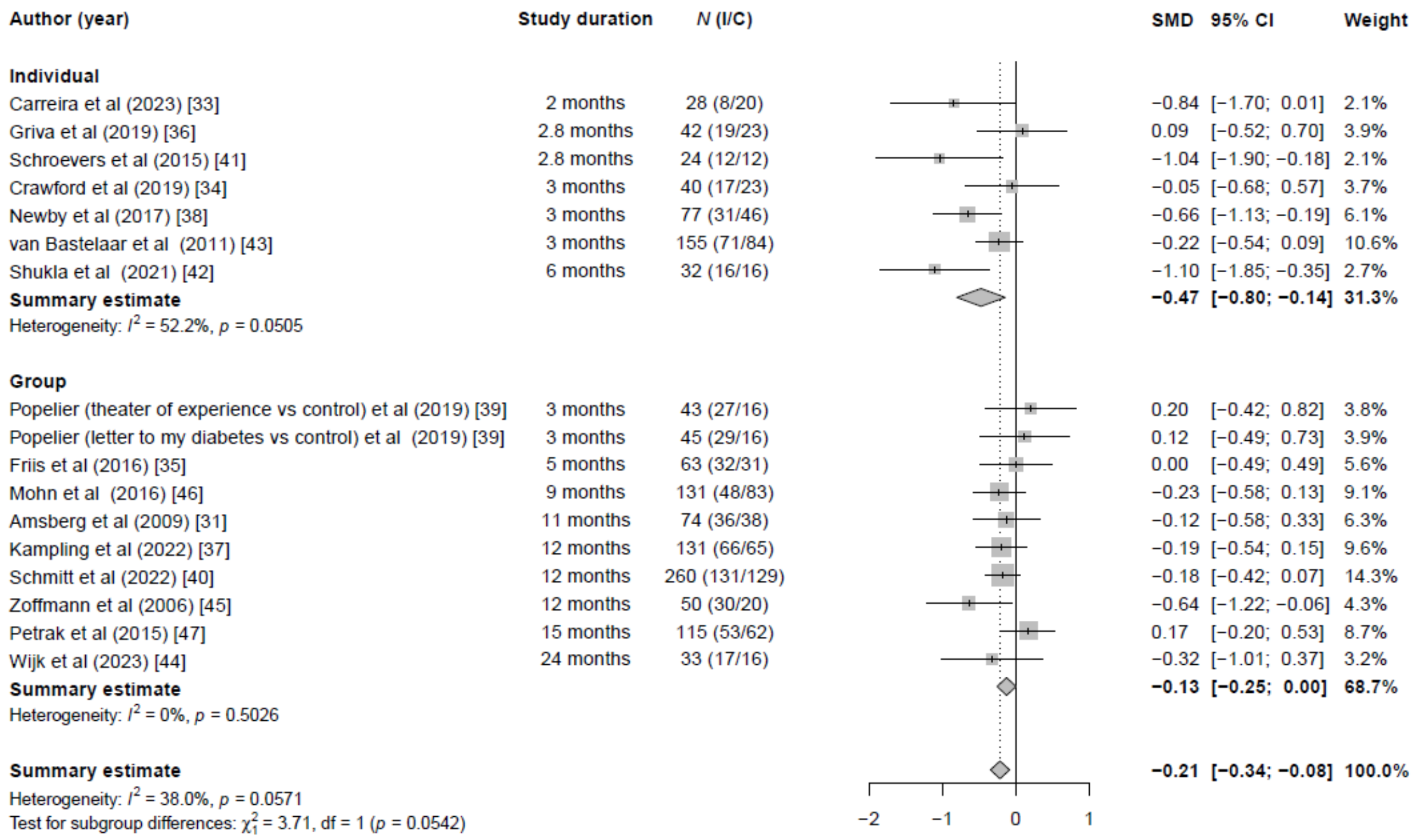

Figure 3d*:* SMD subgroup analysis for psychological interventions compared with usual care for type 1 diabetes stratified by delivery format. C, control; I, intervention

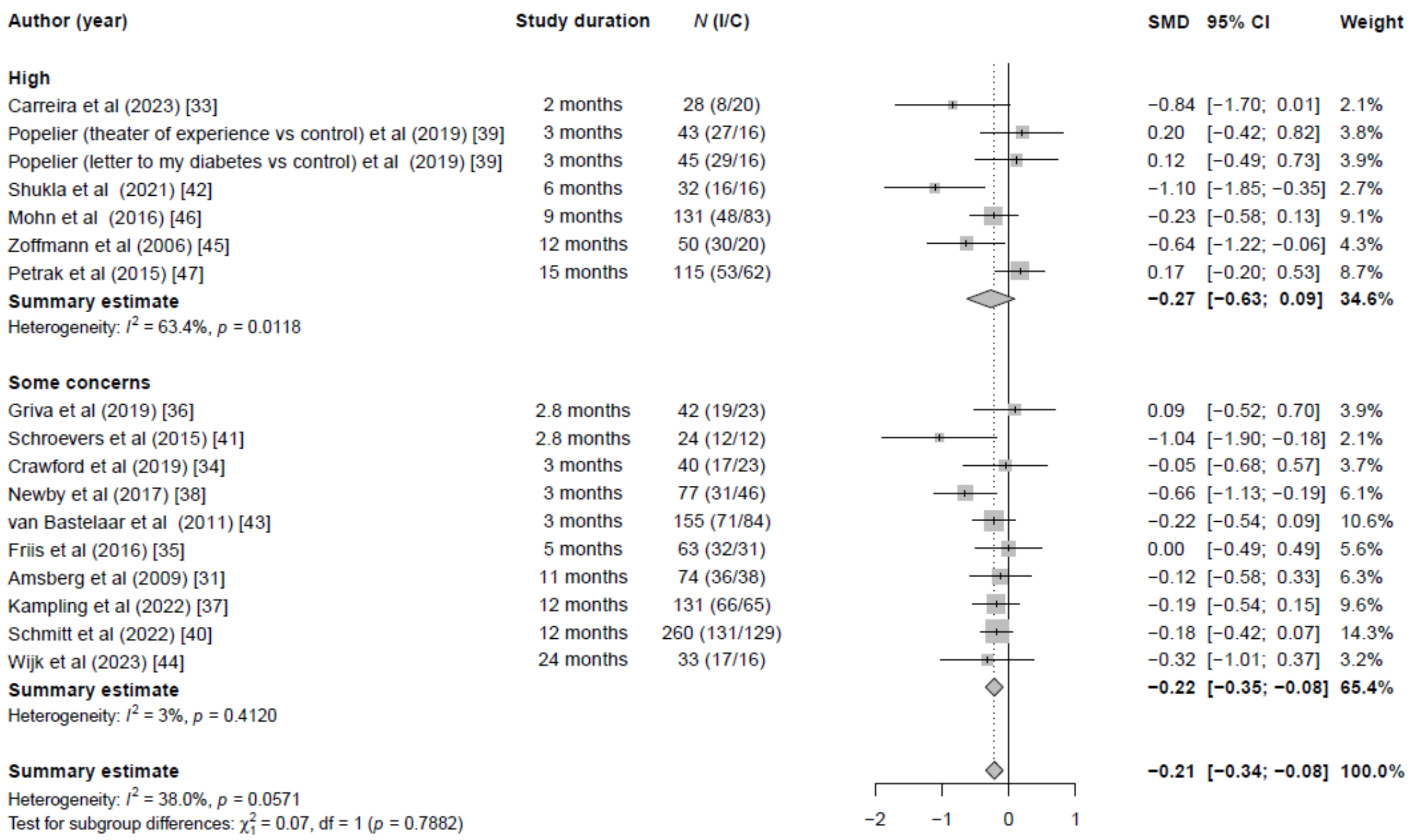


Figure 3e: SMD subgroup analysis for psychological interventions compared with usual care for type 1 diabetes stratified by risk of bias assessment. C, control; I, intervention

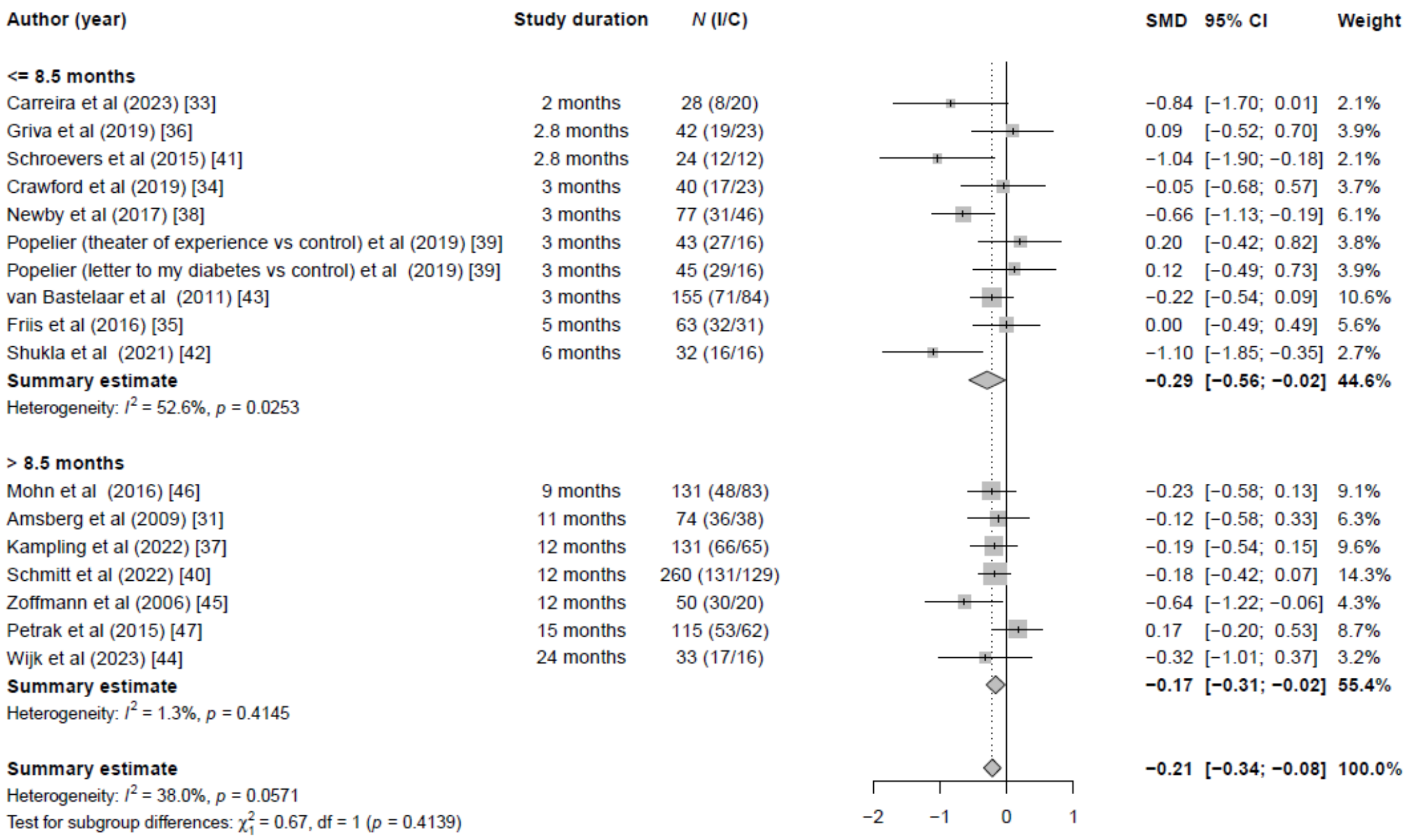

Figure 3f: SMD subgroup analysis for psychological interventions compared with usual care for type 1 diabetes stratified by trial duration. C, control; I, intervention

Psychological interventions compared with educational interventions for type 2 diabetes

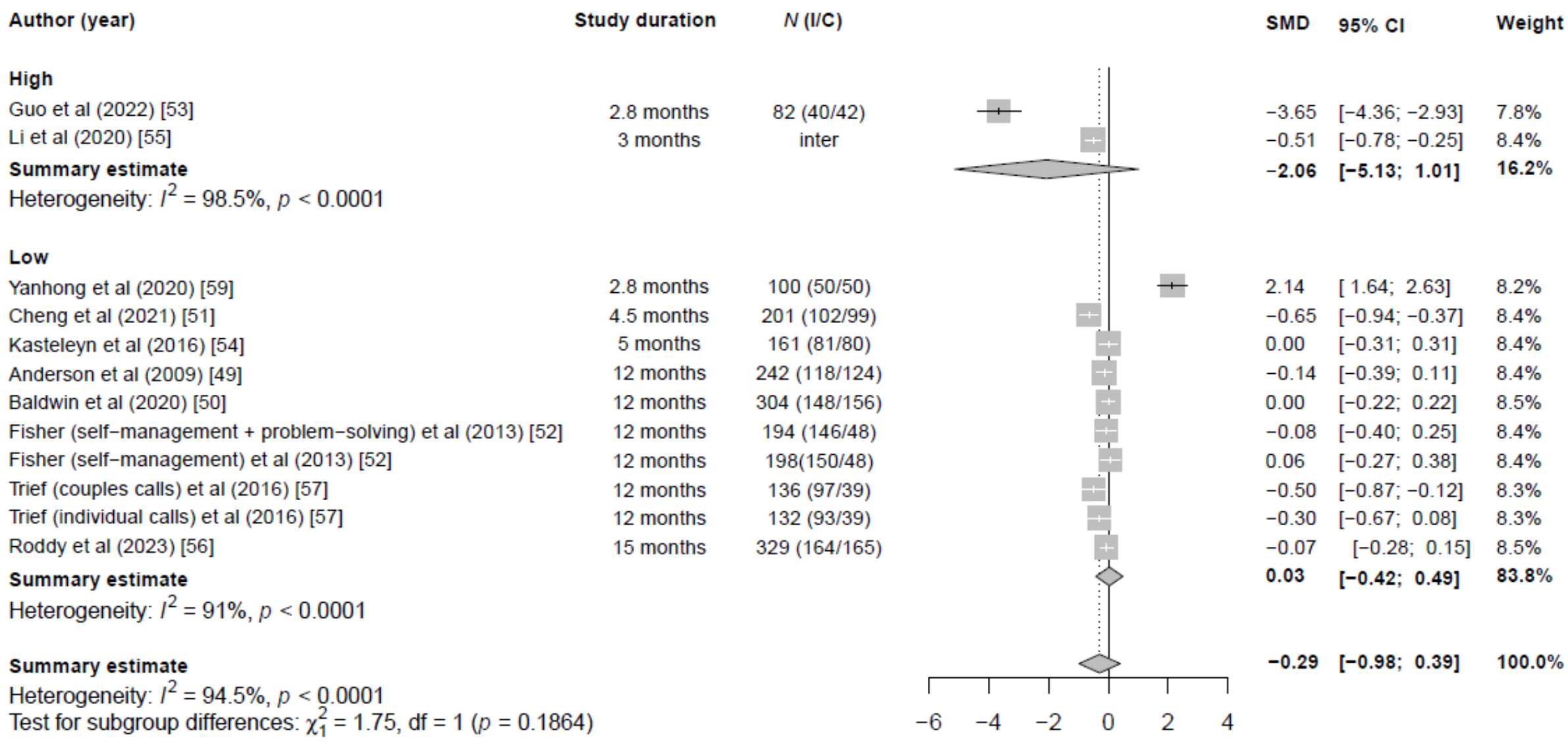


Figure 4c: SMD subgroup analysis for psychological interventions compared with educational interventions for type 2 diabetes stratified by baseline distress level. C, control; I, intervention

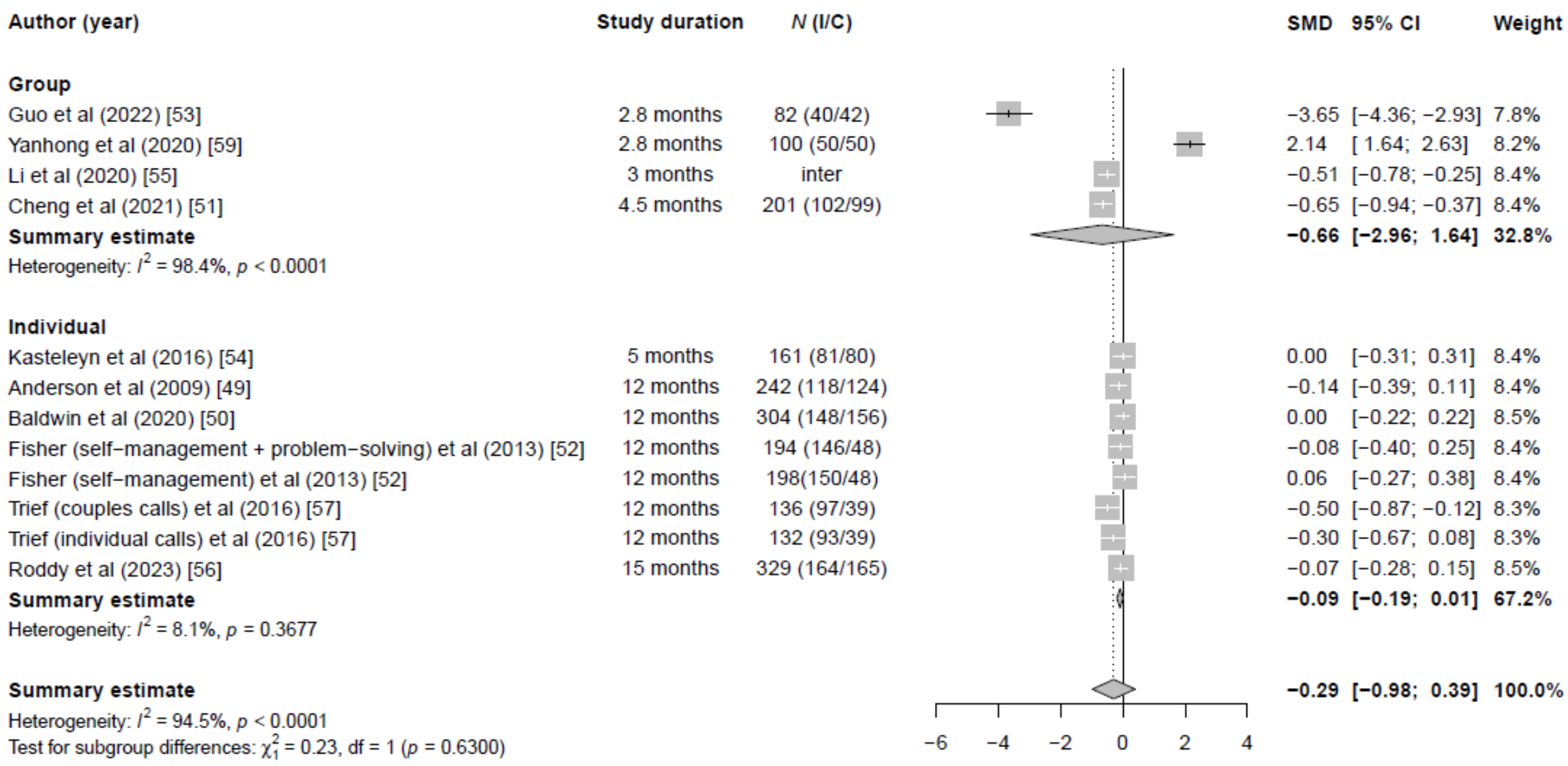


Figure 4d: SMD subgroup analysis for psychological interventions compared with educational interventions for type 2 diabetes stratified by delivery format. C, control; I, intervention

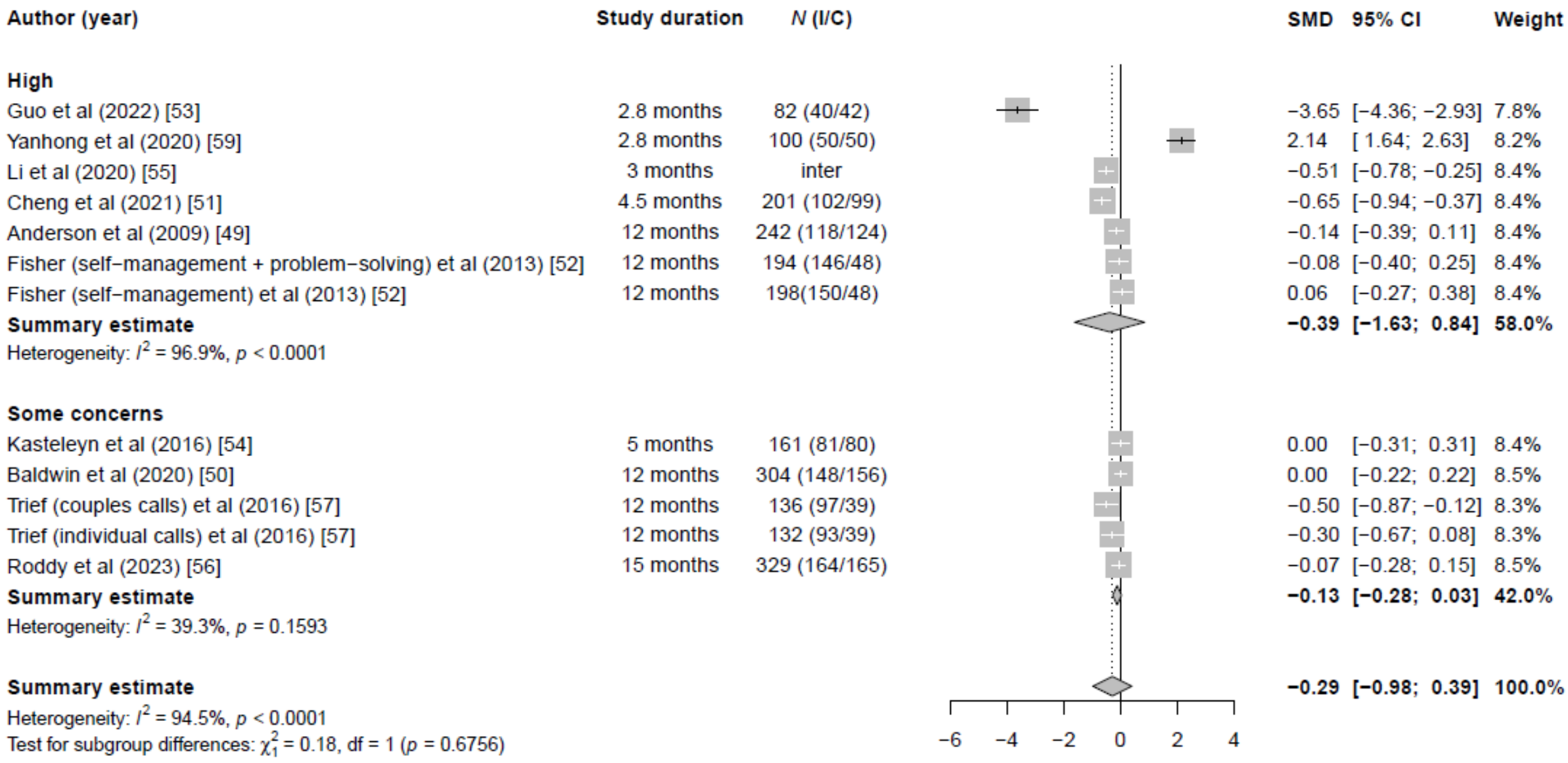


Figure 4e: SMD subgroup analysis for psychological interventions compared with educational interventions for type 2 diabetes stratified by risk of bias assessment. C, control; I, intervention

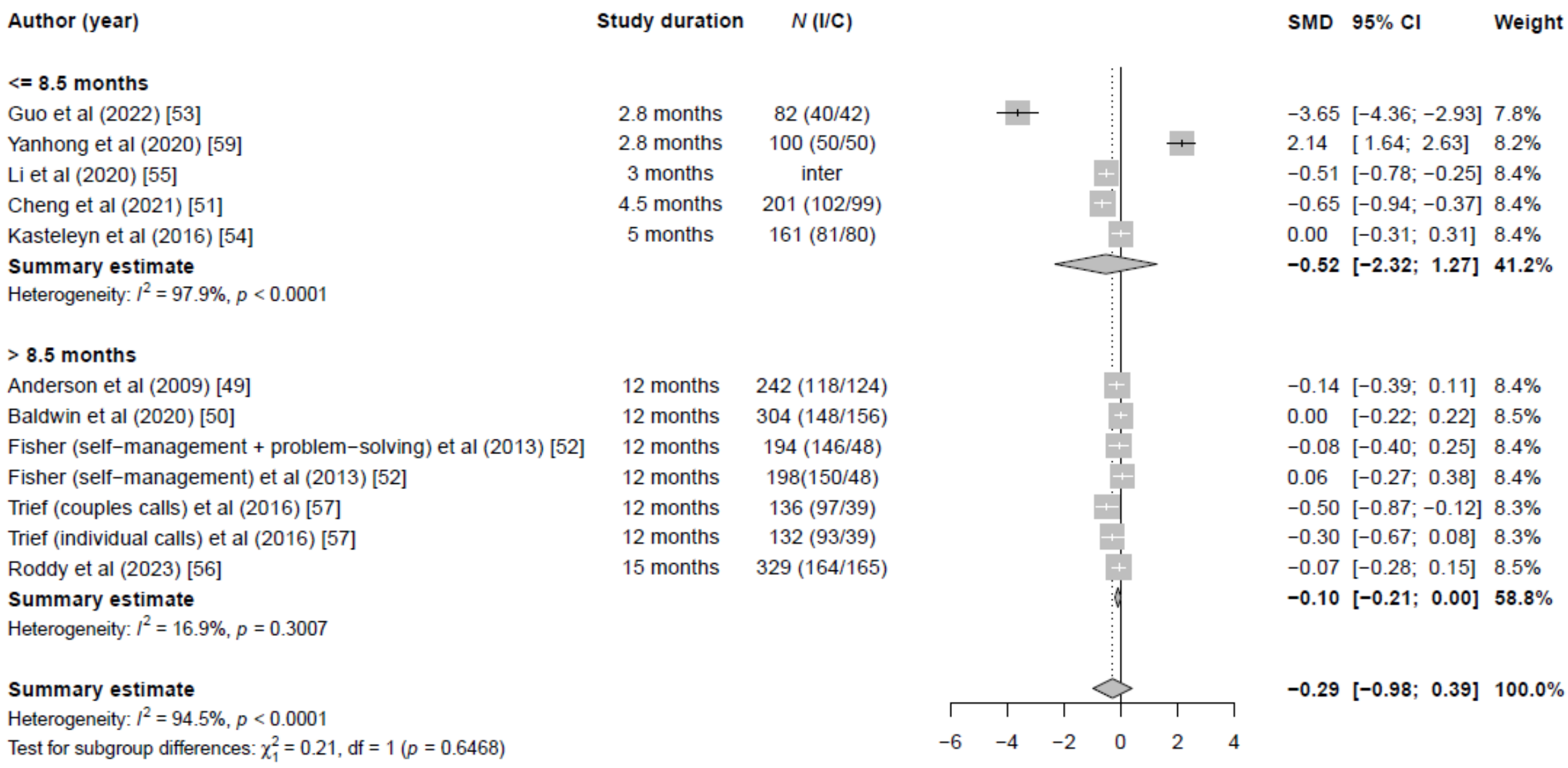


Figure 4f: SMD subgroup analysis for psychological interventions compared with educational interventions for type 2 diabetes stratified by trial duration. C, control; I, intervention

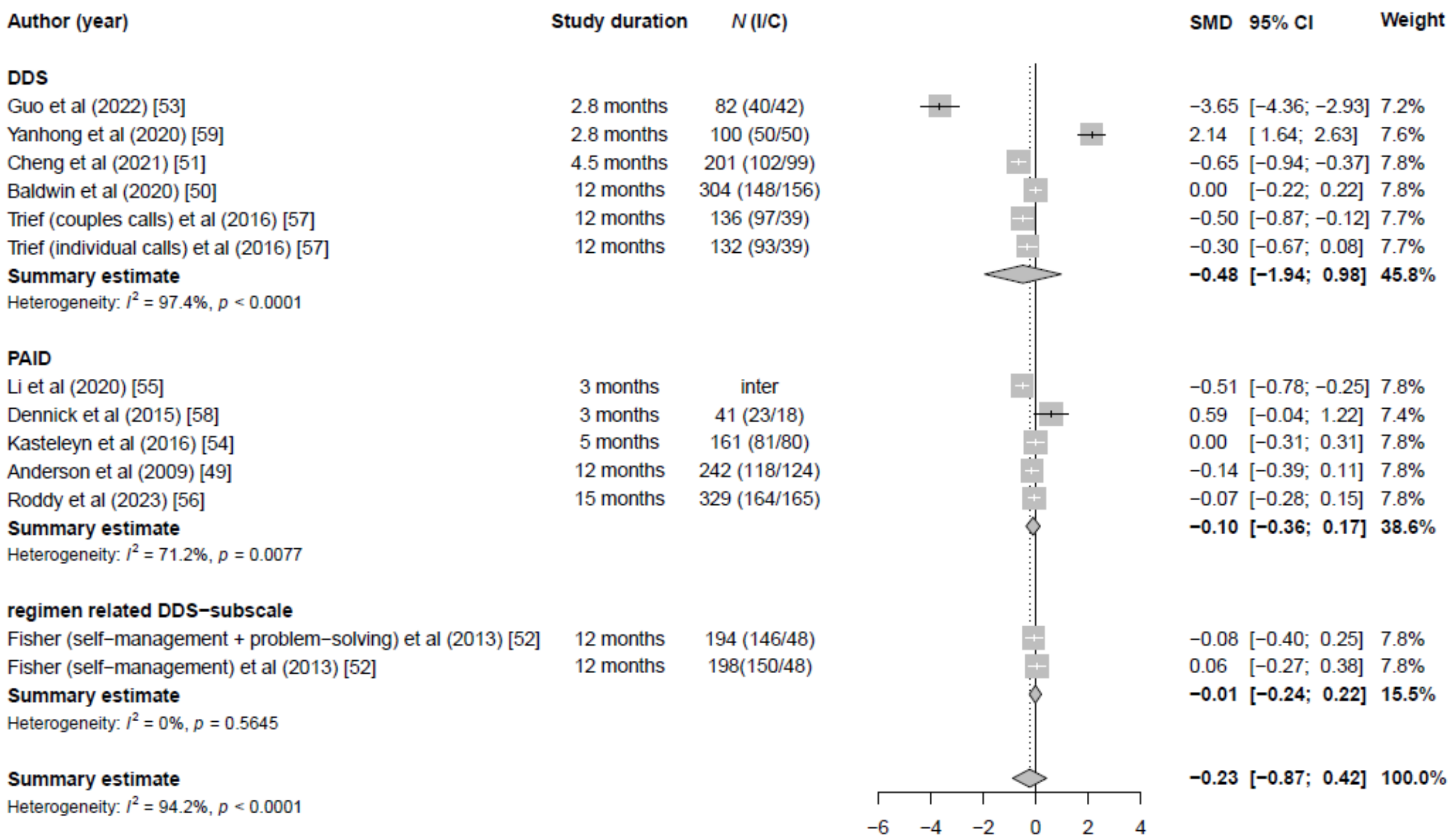


Figure 4g: SMD sensitivity analysis for psychological interventions compared with educational interventions for type 2 diabetes including the study of Dennick et al. C, control; I, intervention

<u>Psychological interventions compared with usual care for type 2 diabetes</u>

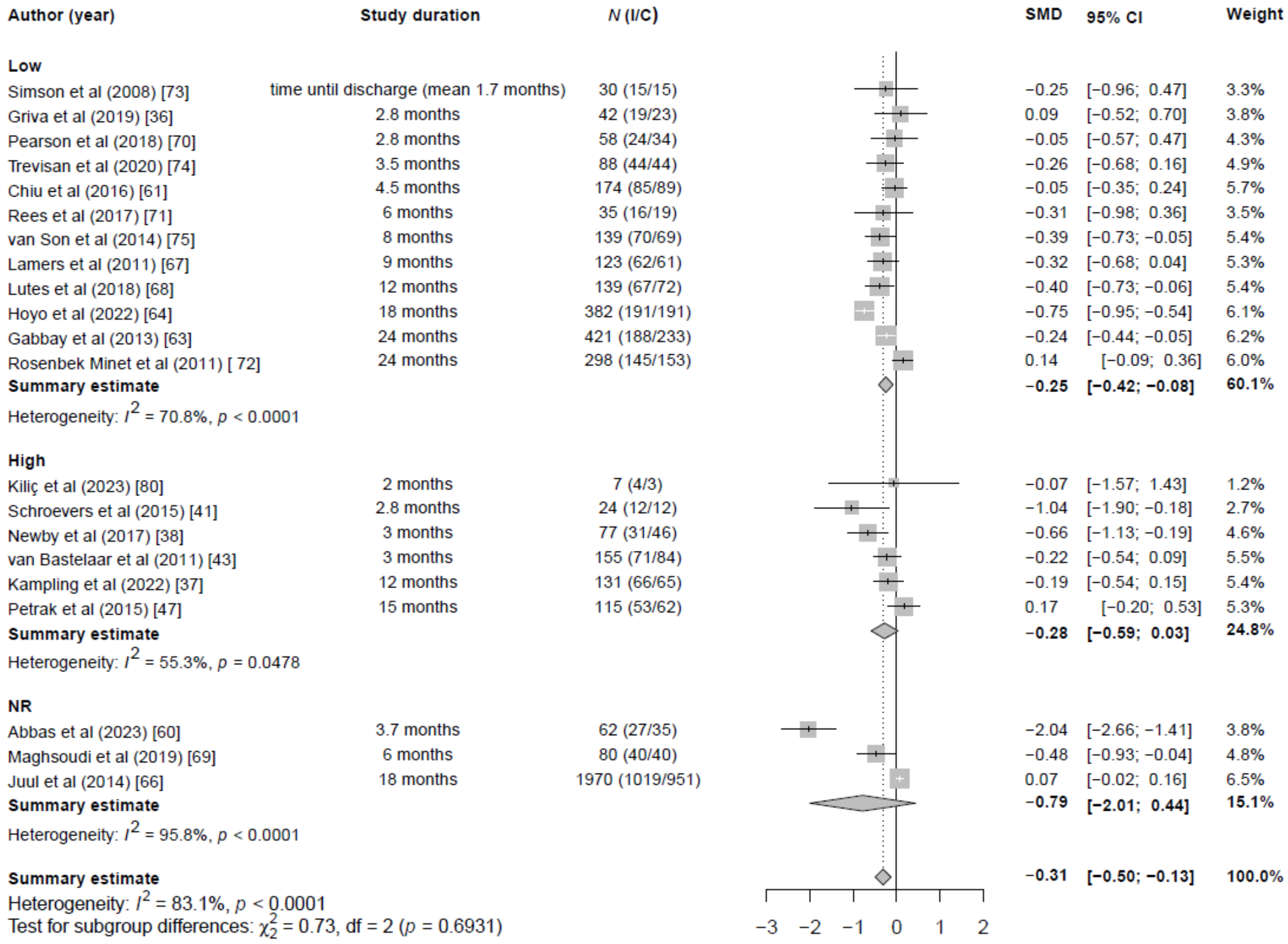


Figure 5c: SMD subgroup analysis for psychological interventions compared with usual care for type 2 diabetes stratified by baseline distress level. C, control; I, intervention

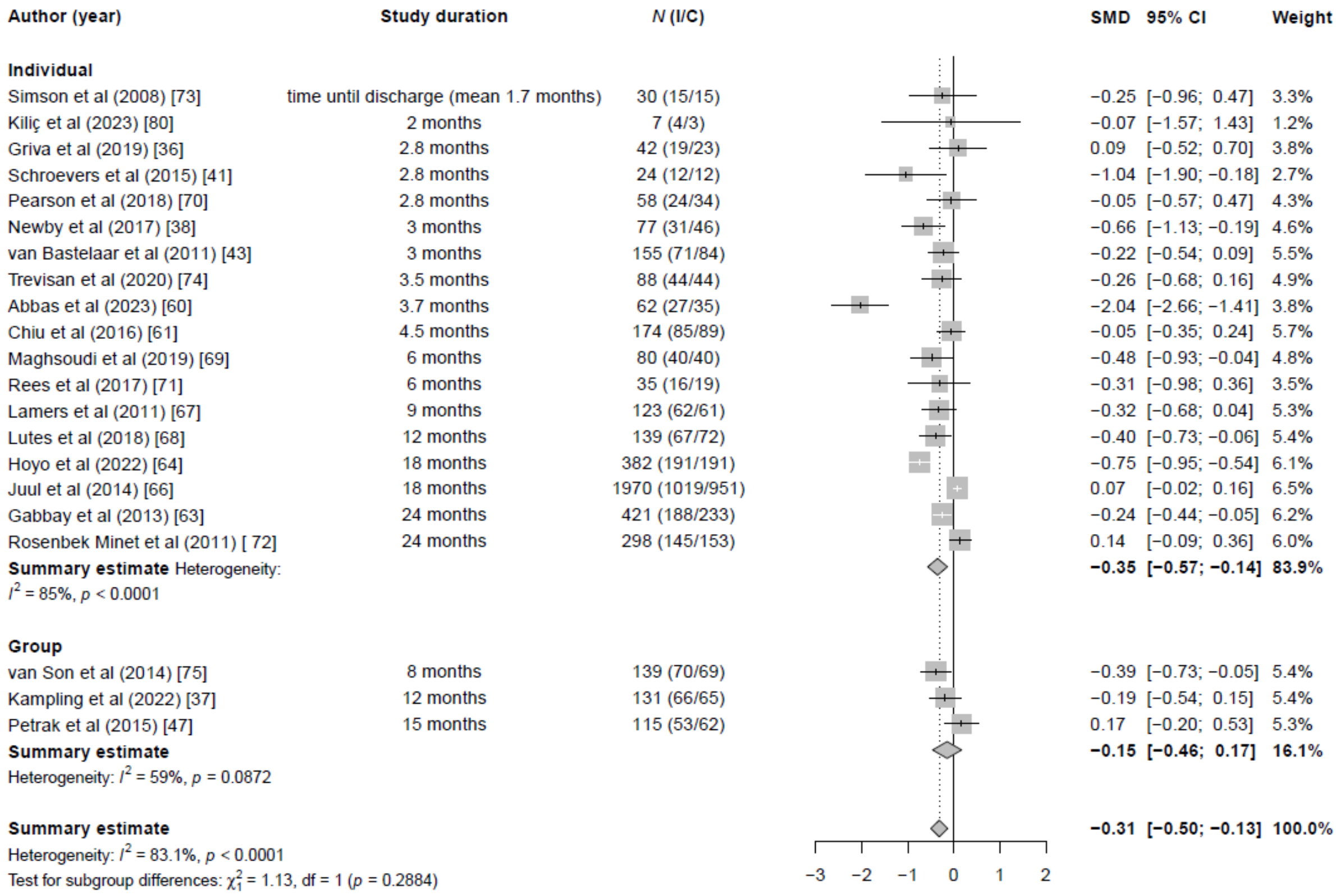


Figure 5d: SMD subgroup analysis for psychological interventions compared with usual care for type 2 diabetes stratified by risk of delivery format. C, control; I, intervention

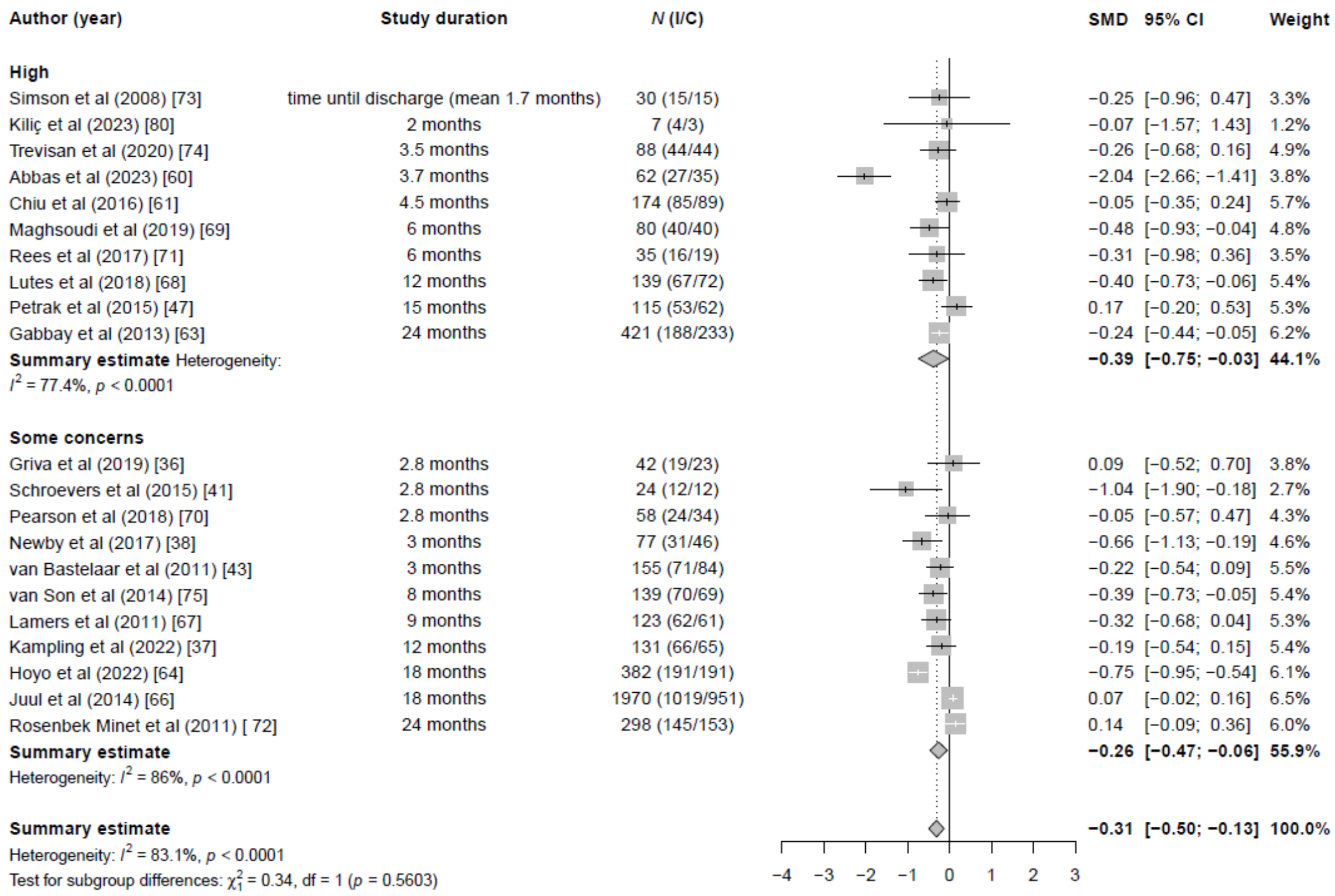

Figure 5e: SMD subgroup analysis for psychological interventions compared with usual care for type 2 diabetes stratified by risk of bias assessment. C, control; I, intervention

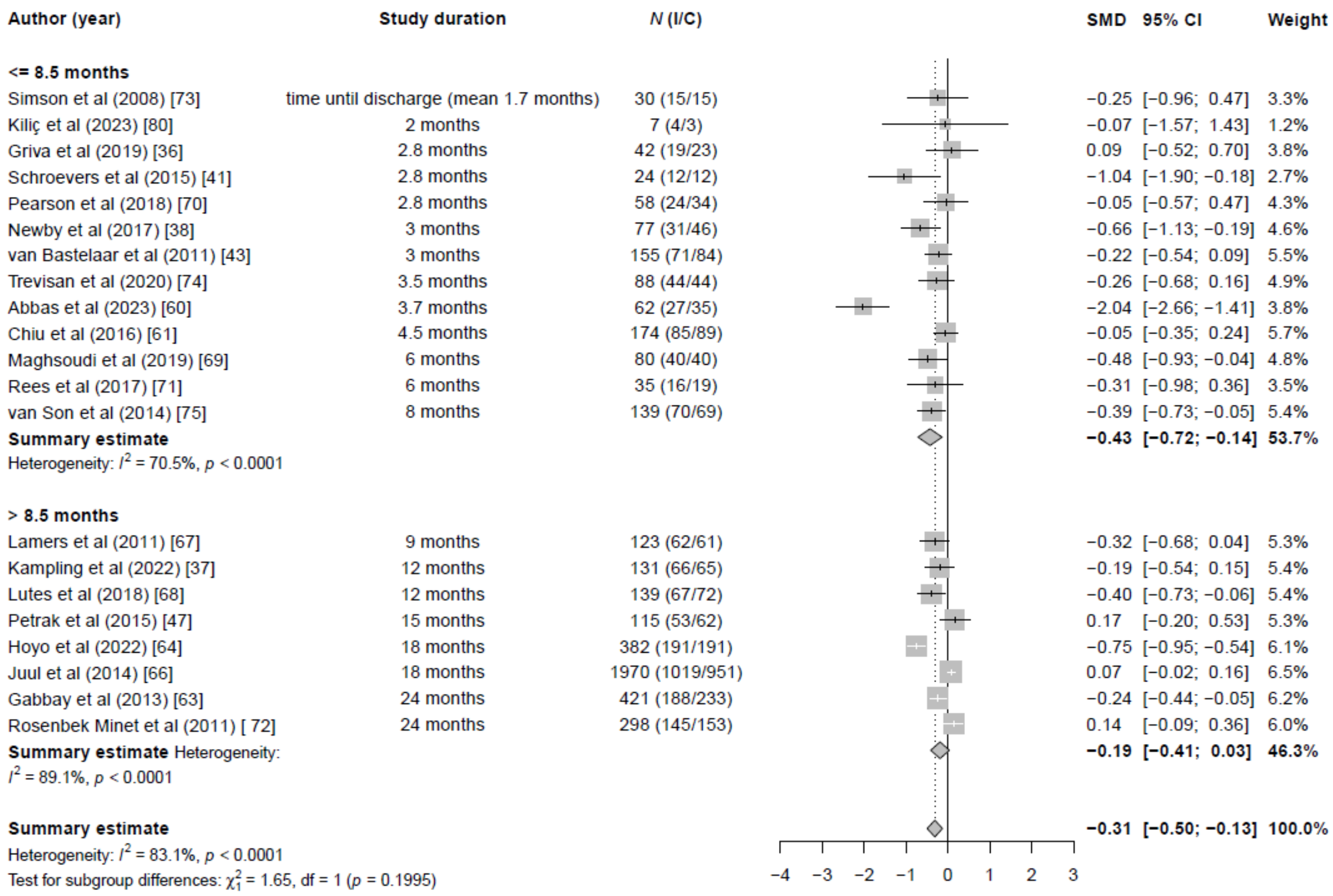


Figure 5f: SMD subgroup analysis for psychological interventions compared with usual care for type 2 diabetes stratified by trial duration. C, control; I, intervention

## Psychoeducational interventions

Psychoeducational interventions compared with educational interventions for type 1 diabetes

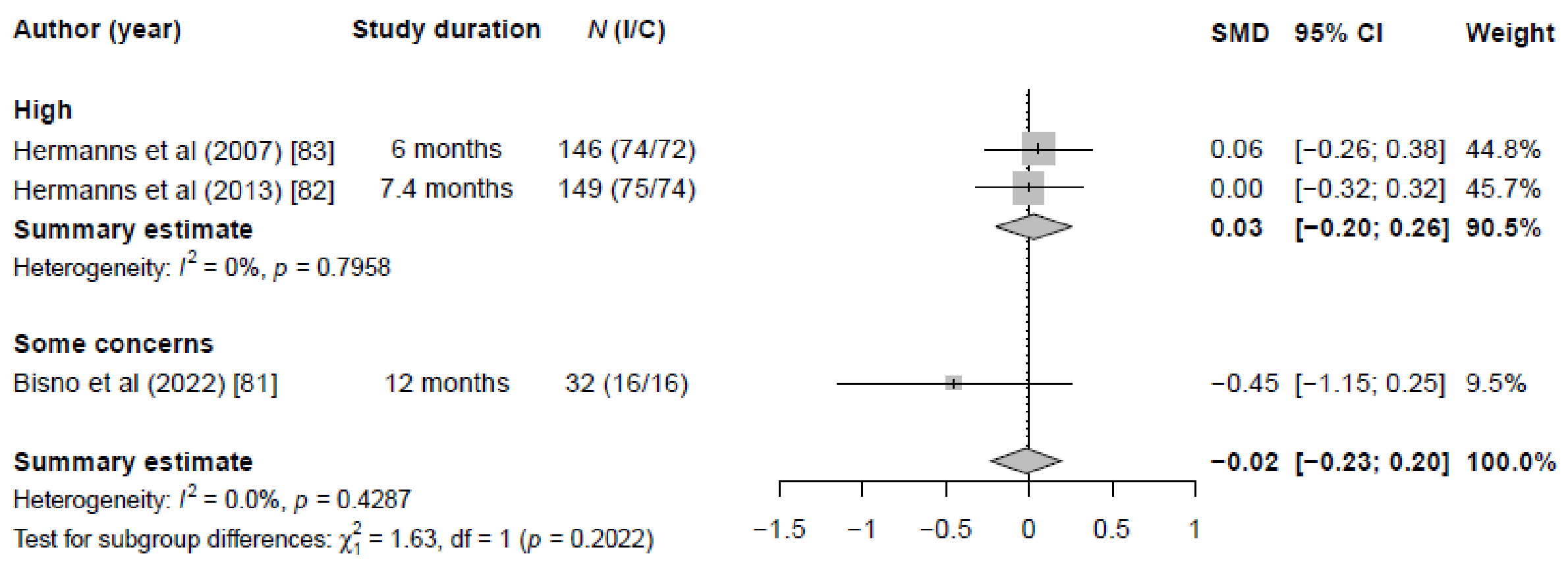


Figure 6c: SMD subgroup analysis for psychoeducational interventions compared with educational interventions for type 1 diabetes stratified by risk of bias assessment. C, control; I, intervention

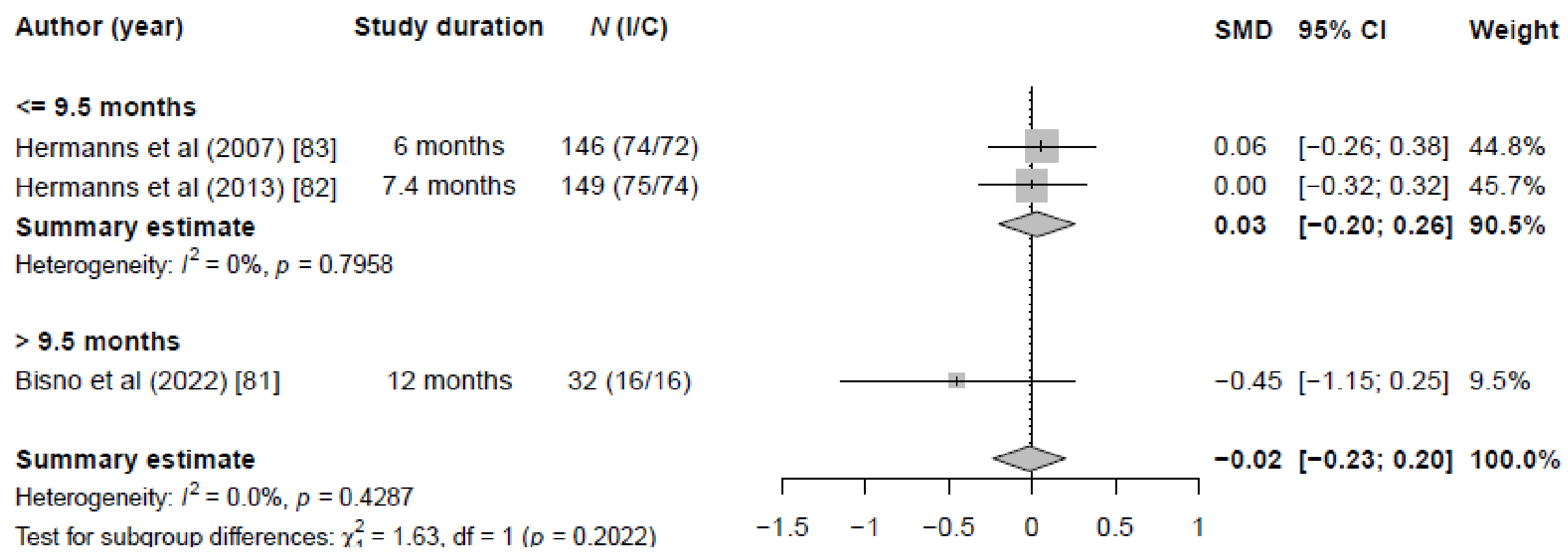


Figure 6d: SMD subgroup analysis for psychoeducational interventions compared with educational interventions for type 1 diabetes stratified by trial duration. C, control; I, intervention

Psychoeducational interventions compared with educational interventions in type 2 diabetes

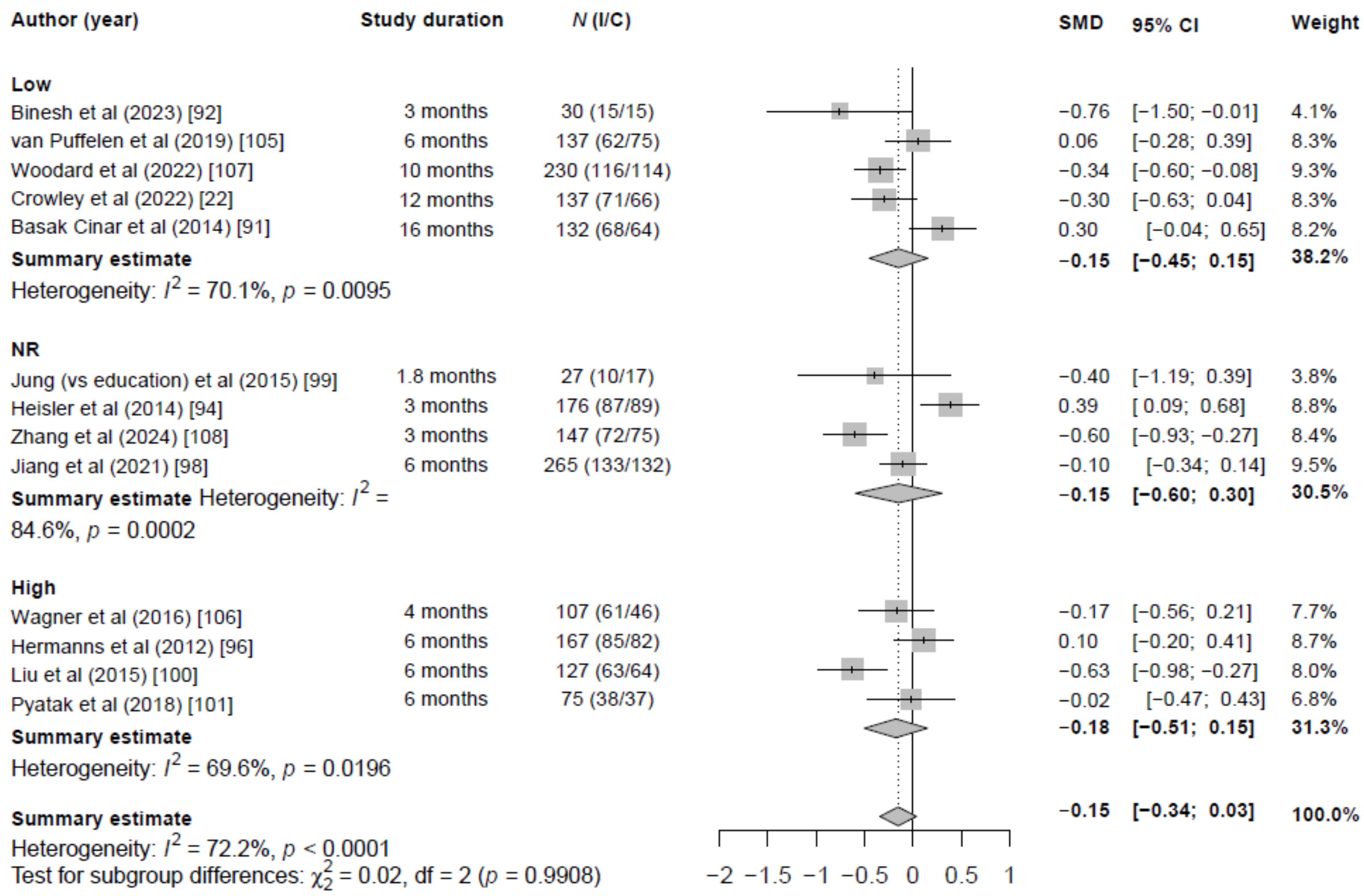


Figure 8c*:* SMD subgroup analysis for psychoeducational interventions compared with educational interventions for type 2 diabetes stratified by baseline distress level. C, control; I, intervention

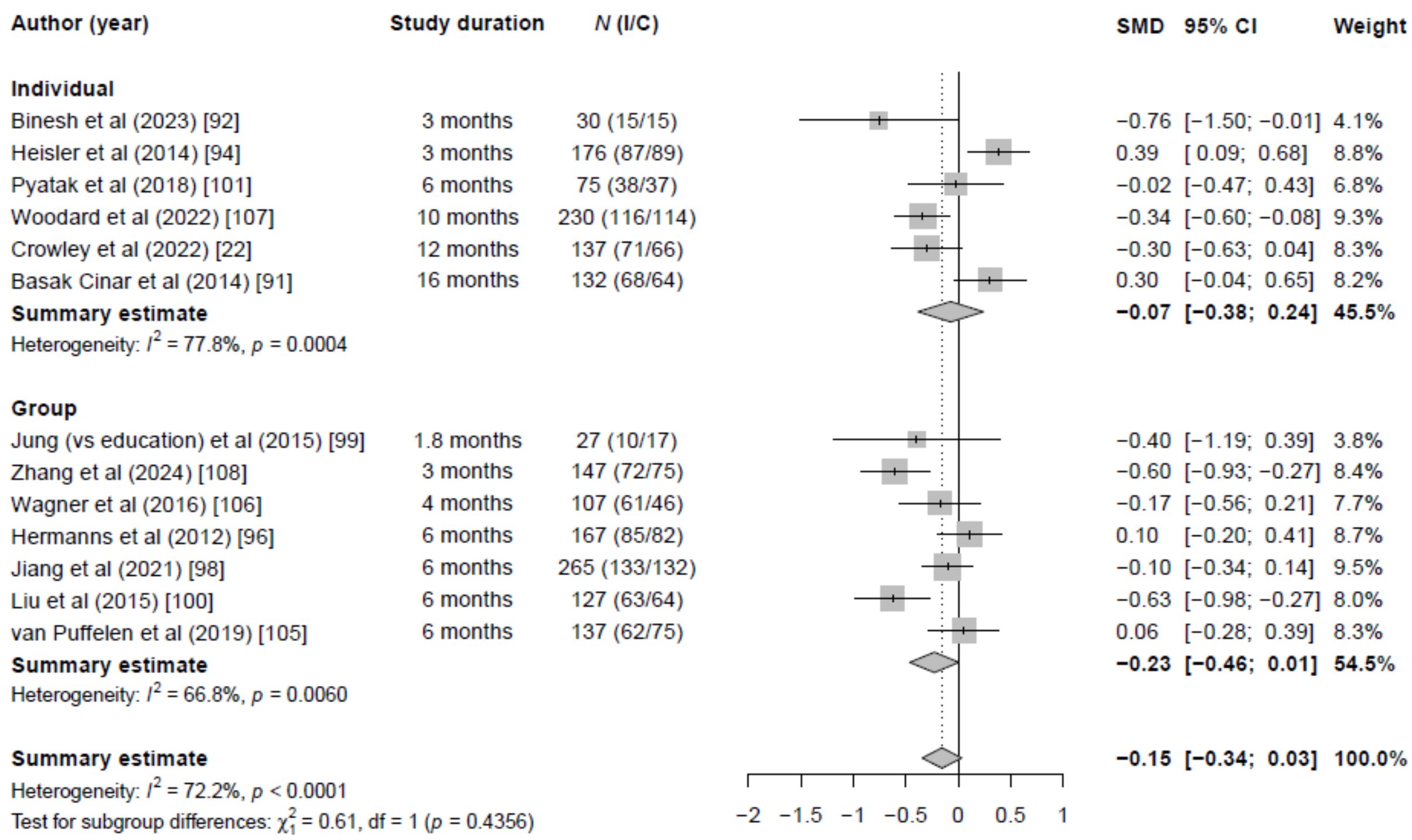


Figure 8d: SMD subgroup analysis for psychoeducational interventions compared with educational interventions for type 2 diabetes stratified by delivery format. C, control; I, intervention

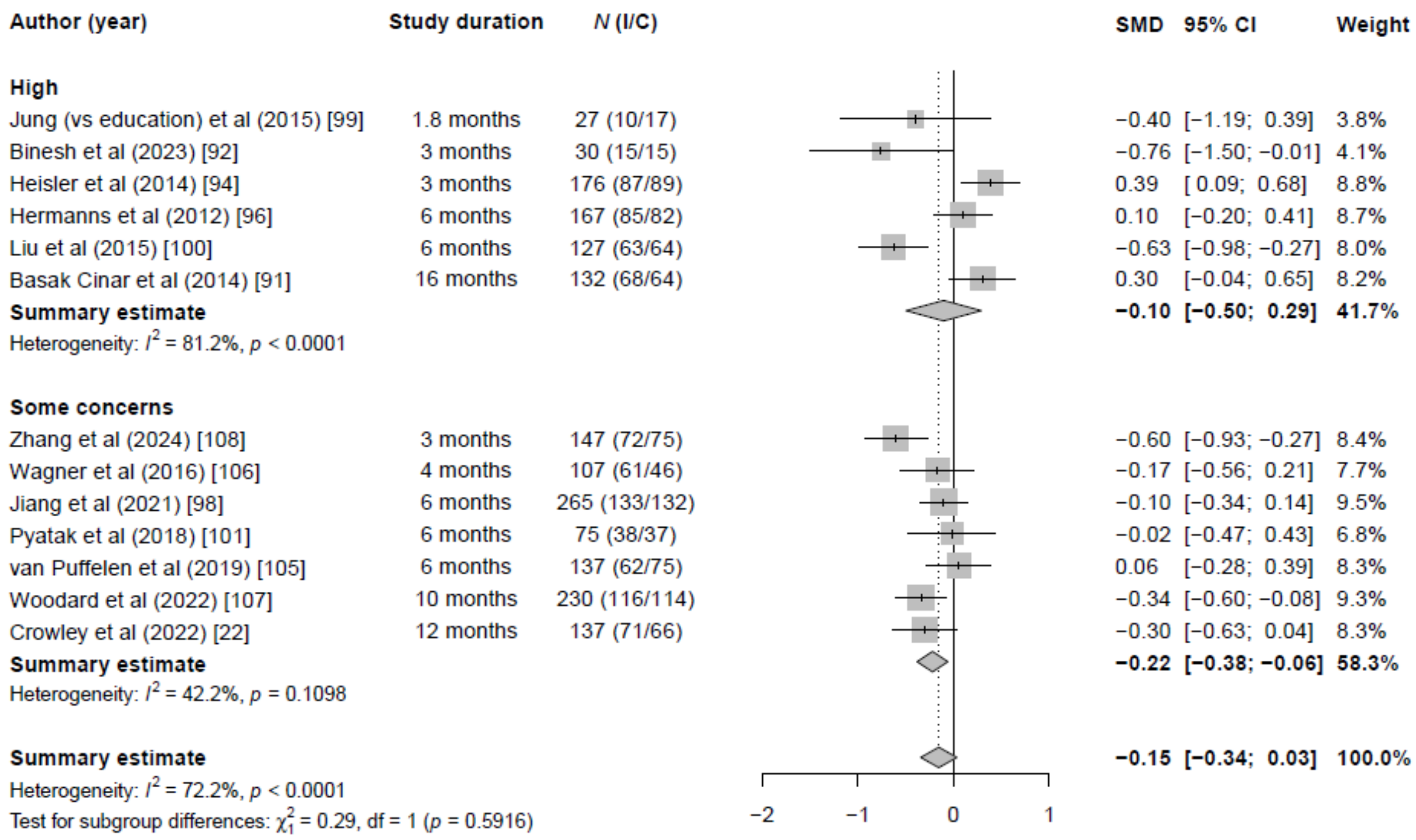


Figure 8e: SMD subgroup analysis for psychoeducational interventions compared with educational interventions for type 2 diabetes stratified by risk of bias assessment. C, control; I, intervention

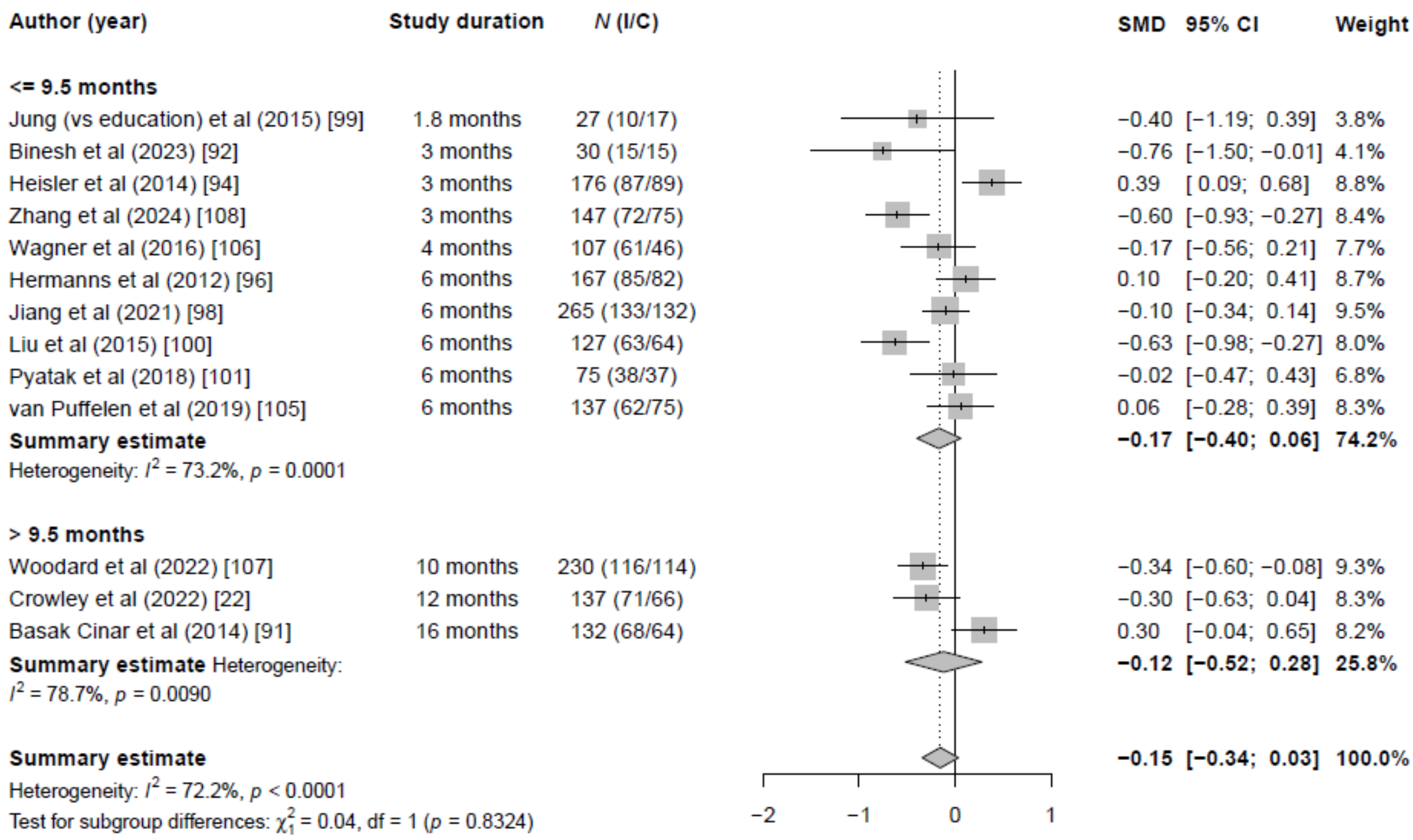


Figure 8f: SMD subgroup analysis for psychoeducational interventions compared with educational interventions for type 2 diabetes stratified by trial duration. C, control; I, intervention

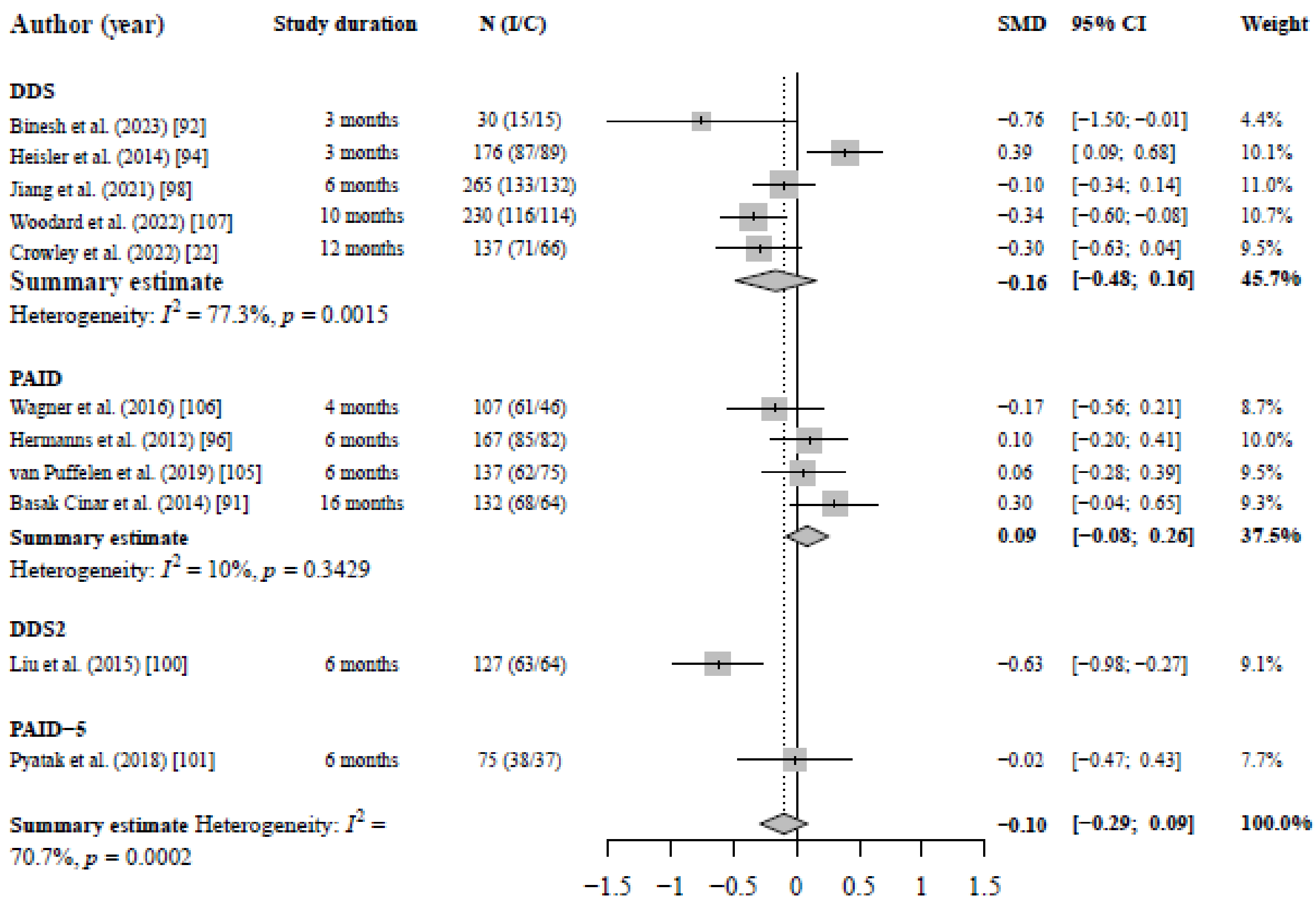


Figure 8g: SMD sensitivity analysis for psychoeducational interventions compared with educational interventions for type 2 diabetes including walking arm. C, control; I, intervention

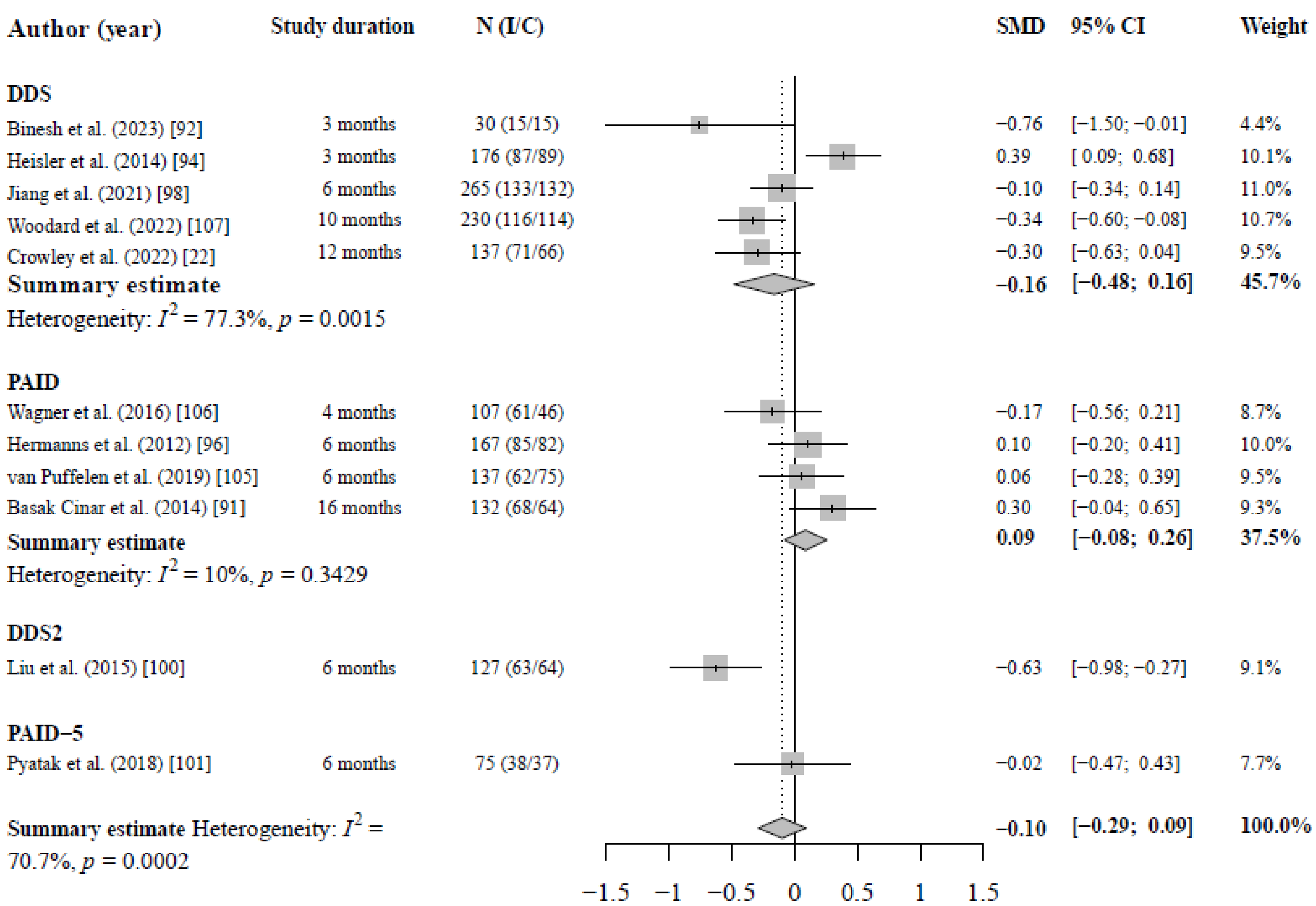

Figure 8h: SMD sensitivity analysis for psychoeducational interventions compared with educational interventions in type 2 diabetes excluding cluster randomized controlled trials without reported intraclass correlation coefficient. C, control; I, intervention

Psychoeducational interventions compared with usual care in type 2 diabetes

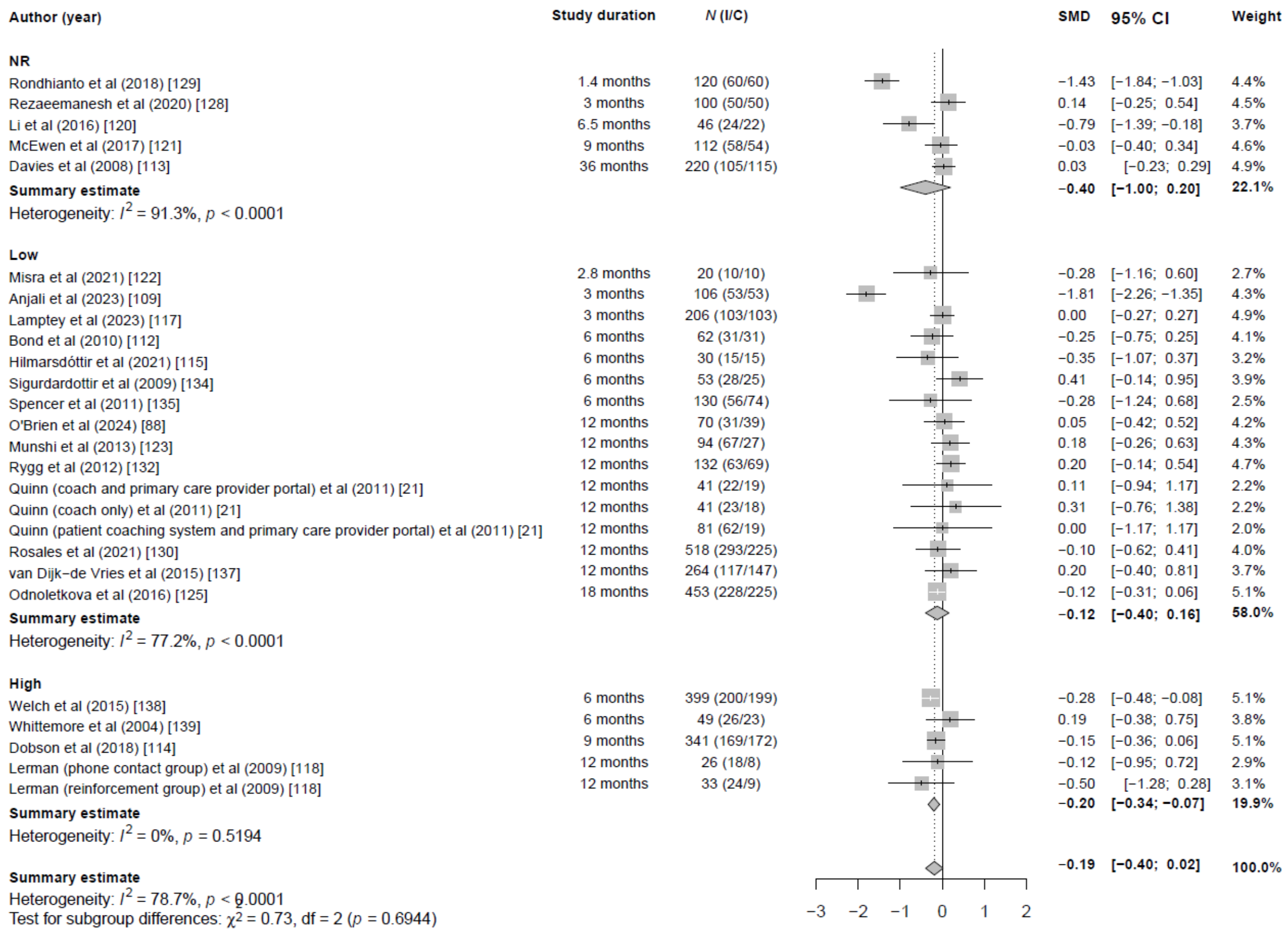


Figure 9c: SMD subgroup analysis for psychoeducational interventions compared with usual care for type 2 diabetes stratified by baseline distress level. C, control; I, intervention

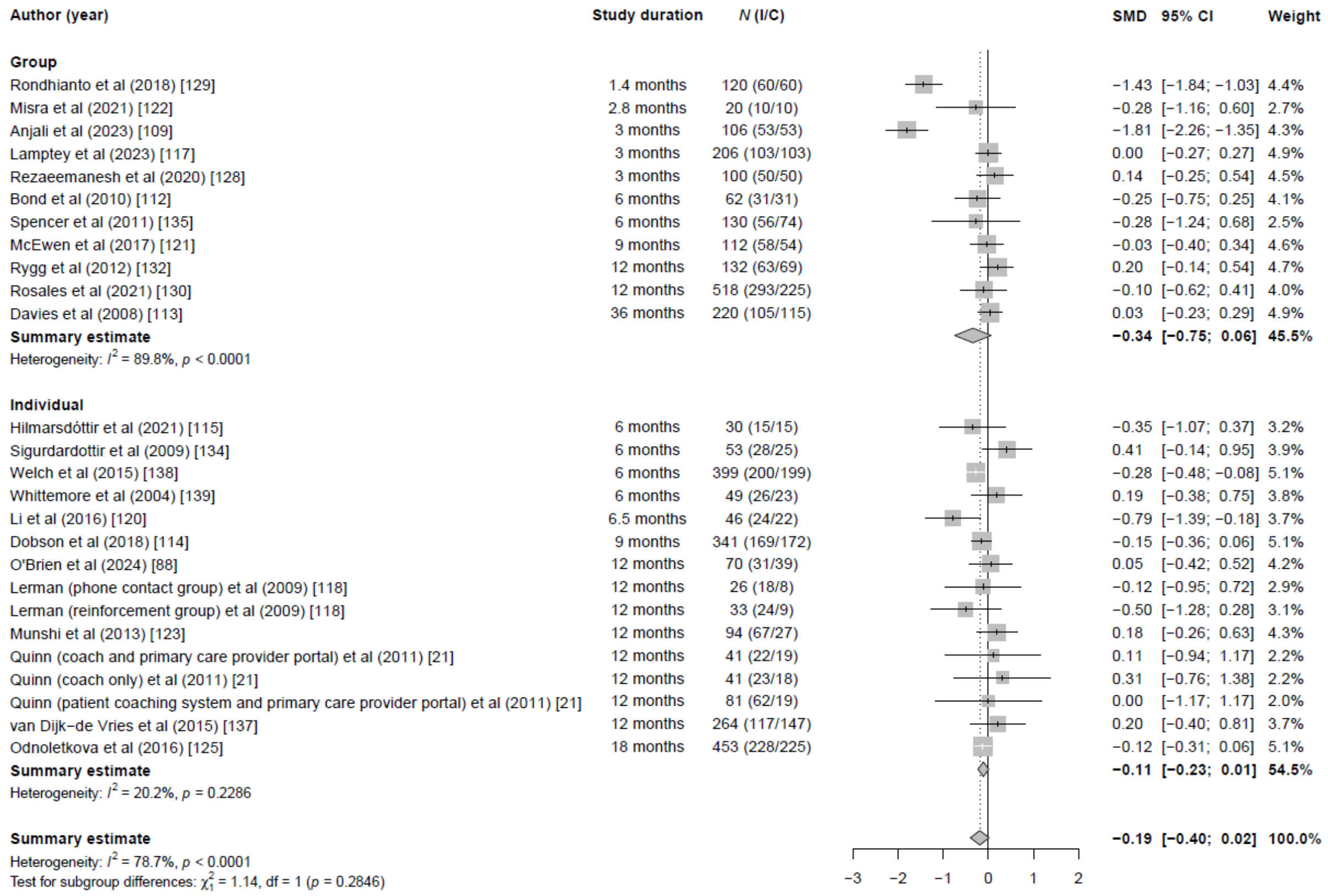


Figure 9d: SMD subgroup analysis for psychoeducational interventions compared with usual care for type 2 diabetes stratified by delivery format. C, control; I, intervention

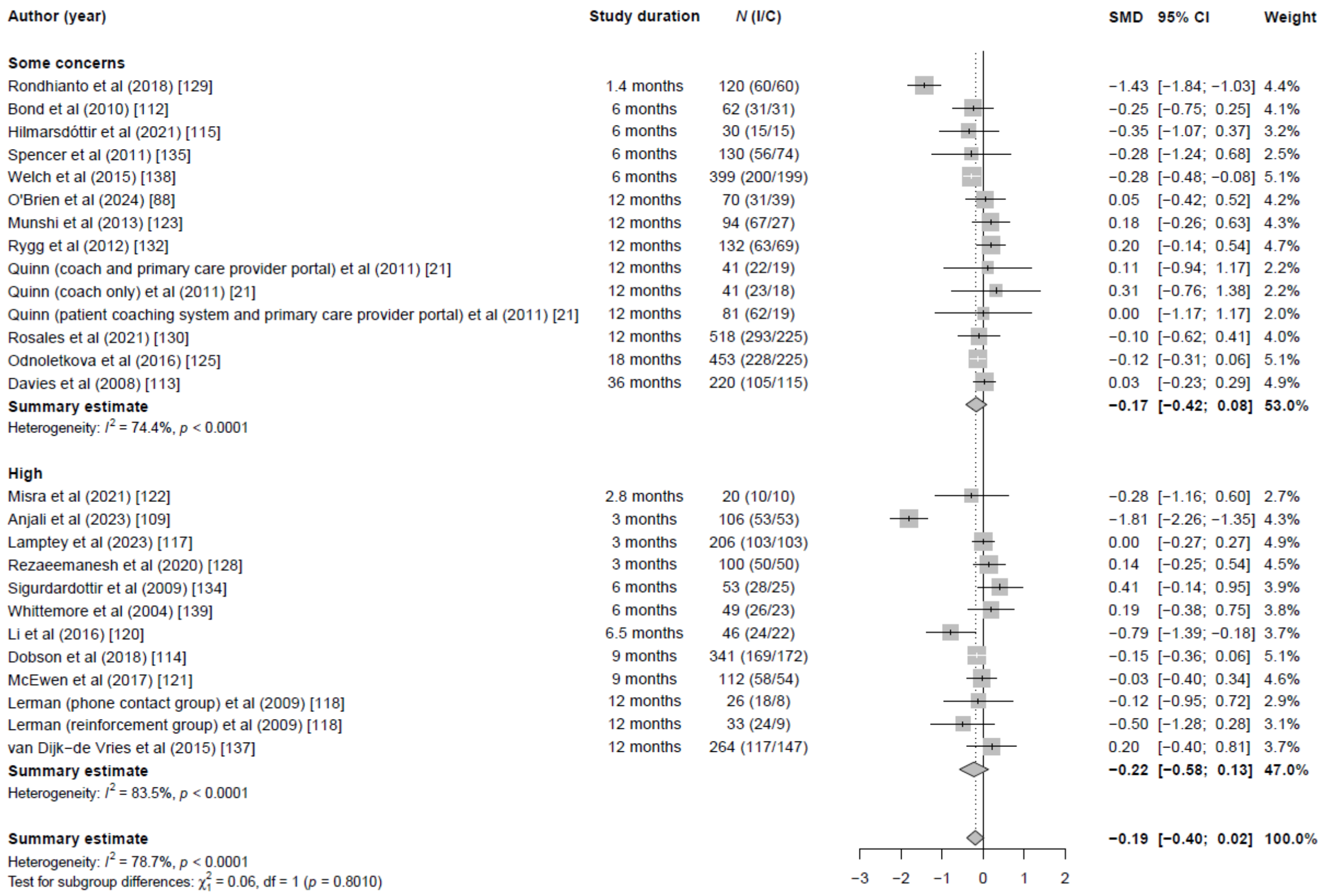

Figure 9e: SMD subgroup analysis for psychoeducational interventions compared with usual care for type 2 diabetes stratified by risk of bias assessment. C, control; I, intervention

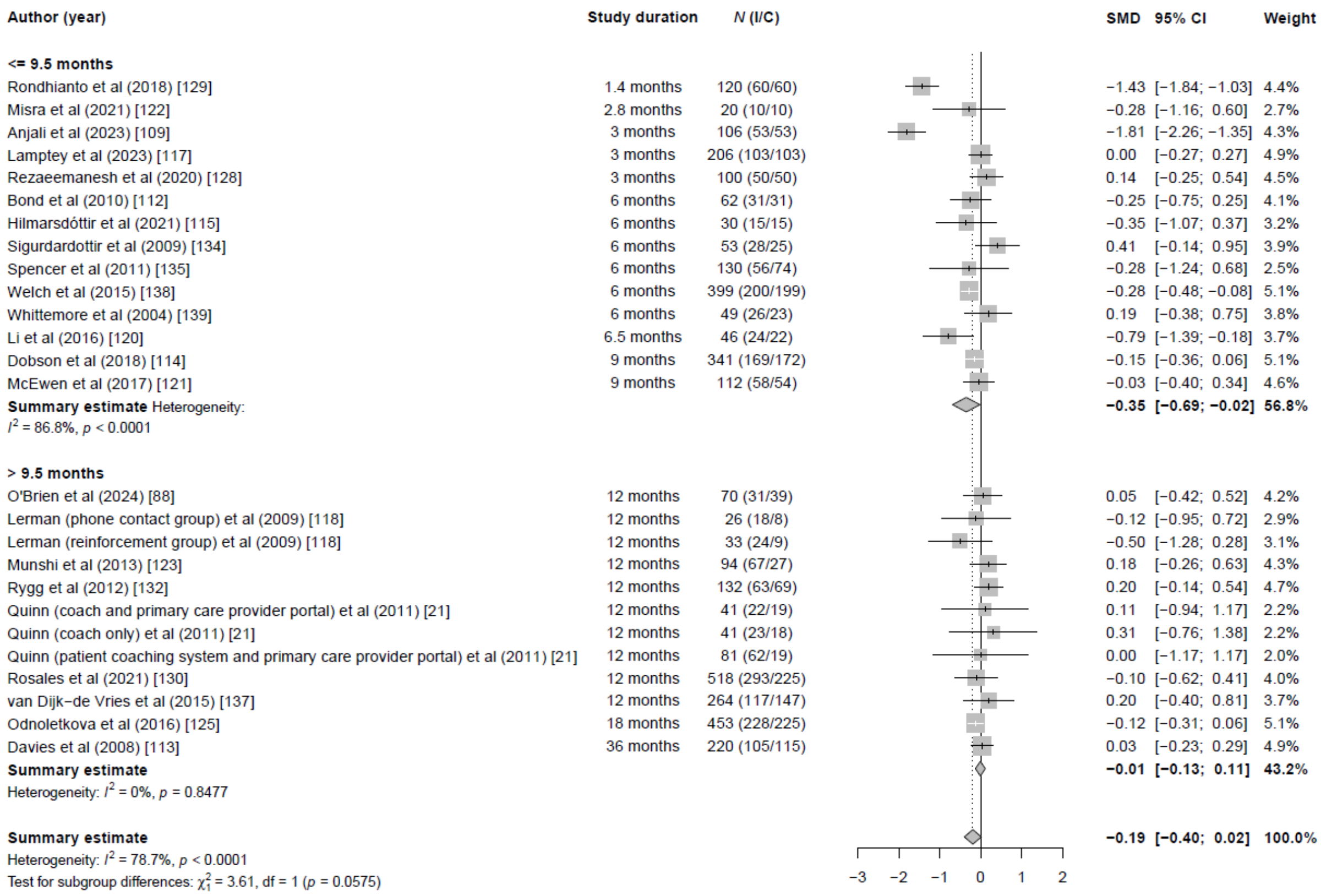


Figure 9f: SMD subgroup analysis for psychoeducational interventions compared with usual care for type 2 diabetes stratified by trial duration. C, control; I, intervention

## Miscellaneous interventions

*Devices in type 1 diabetes*

Automated insulin delivery compared with no automated insulin delivery

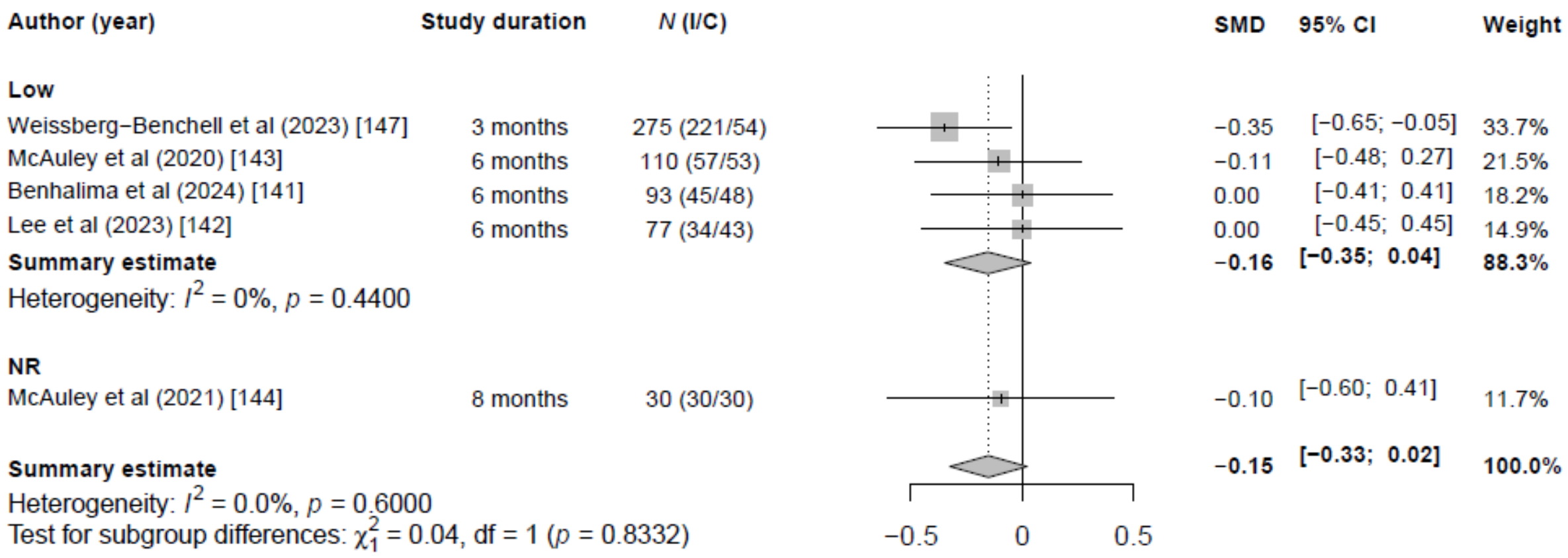


Figure 10c: SMD subgroup analysis for AID compared with no use of AID  in type 1 diabetes stratified by baseline distress level. C, control; I, intervention

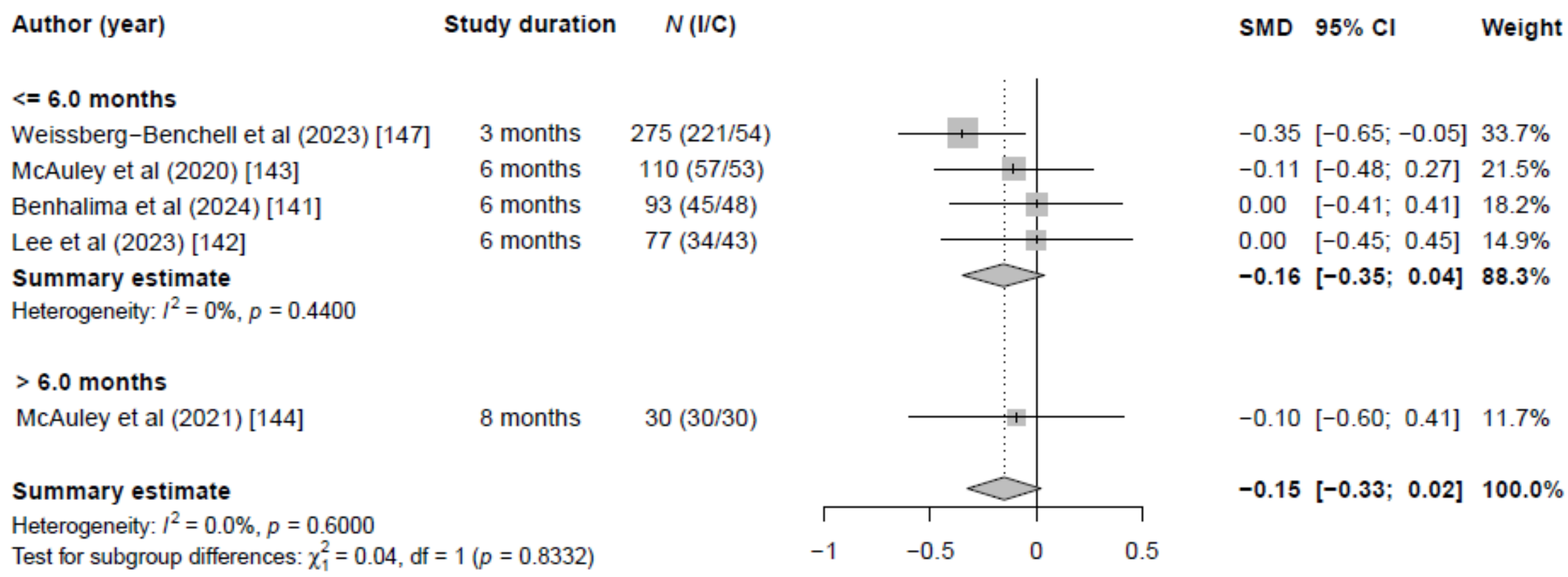


Figure 10d: Standardized mean difference subgroup analysis for AID compared with no use of AID in type 1 diabetes stratified by trial duration. C, control; I, intervention

Continuous glucose/ intermittently scanned monitoring compared with standard blood glucose measurement for type 1 diabetes

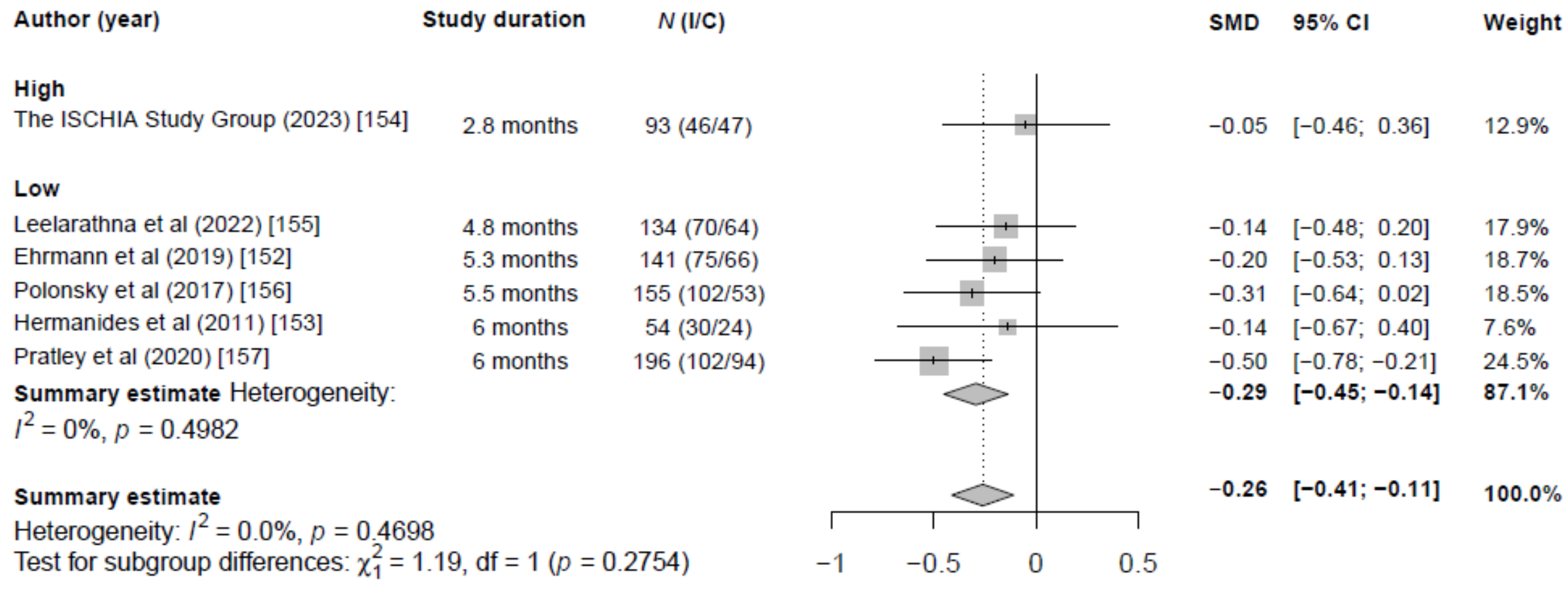


Figure 11c: SMD subgroup analysis CGM/ intermittently scanned CGM compared with capillary glucose monitoring in type 1 diabetes stratified by baseline distress level. C, control; I, intervention

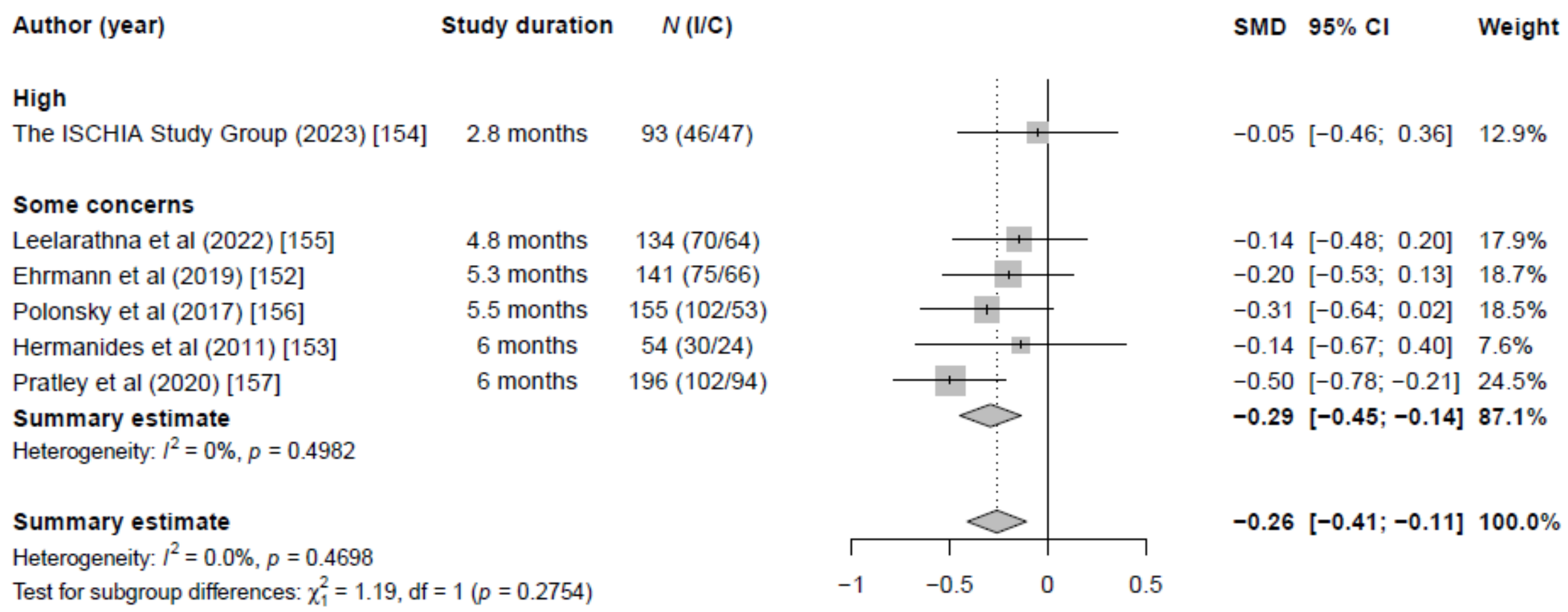


Figure 11d: SMD subgroup analysis CGM/ intermittently scanned CGM compared with capillary glucose monitoring in type 1 diabetes stratified by risk of bias assessment. C, control; I, intervention

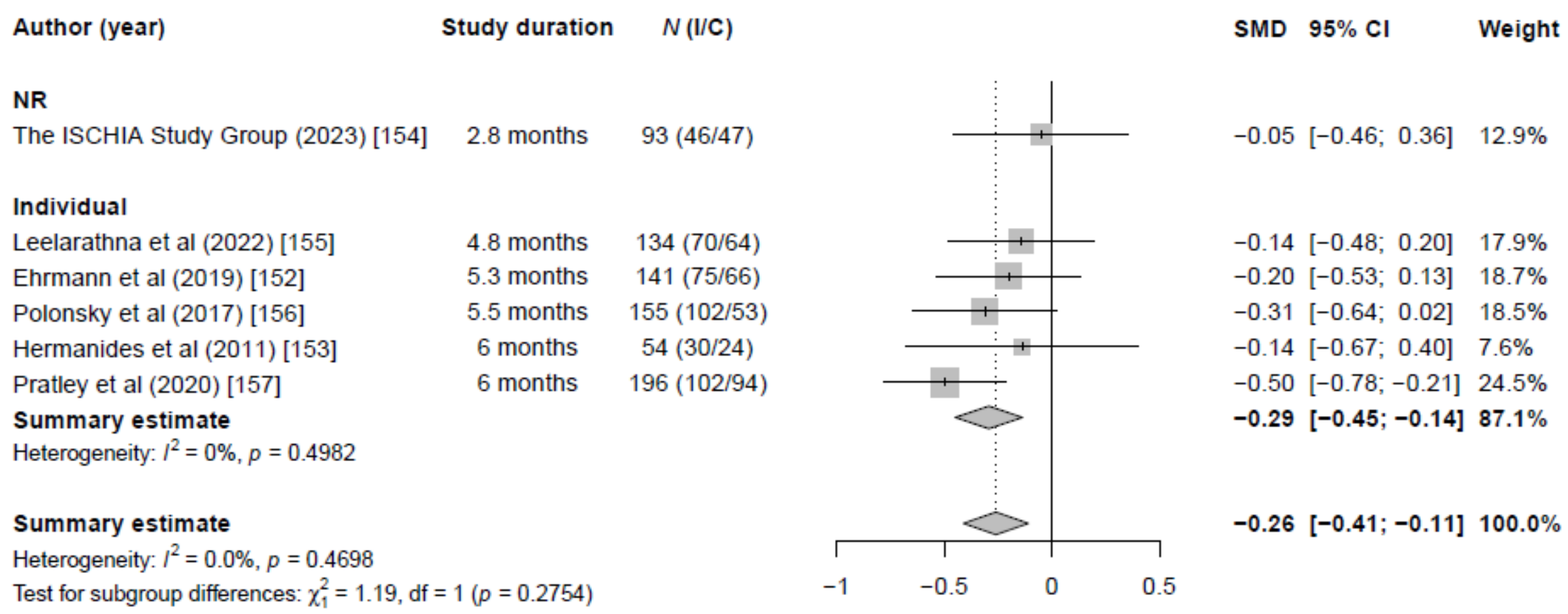

Figure 11e: SMD subgroup analysis for CGM/ intermittently scanned CGM compared with capillary glucose monitoring in type 1 diabetes stratified by delivery format. C, control; I, intervention

*Devices in type 2 diabetes*

<u>Devices compared with usual care for type 2 diabetes</u>

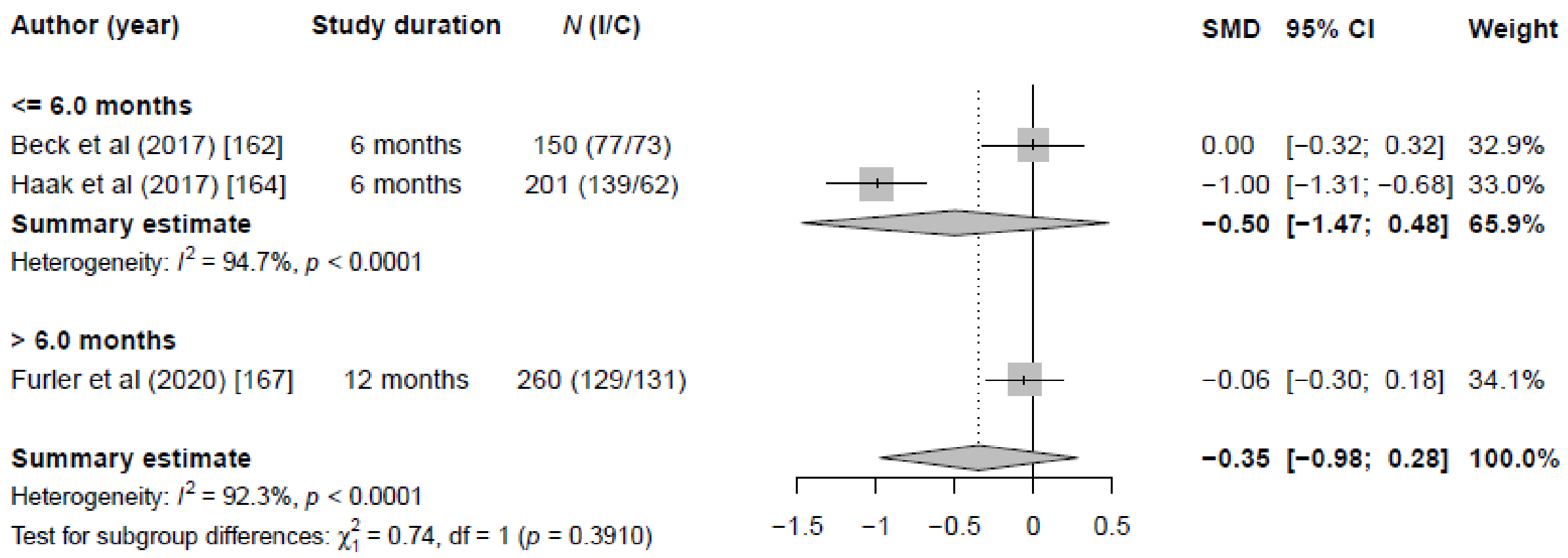


Figure 12c: SMD subgroup analysis for CGM compared with capillary glucose monitoring in type 2 diabetes stratified by risk of bias assessment. C, control; I, intervention

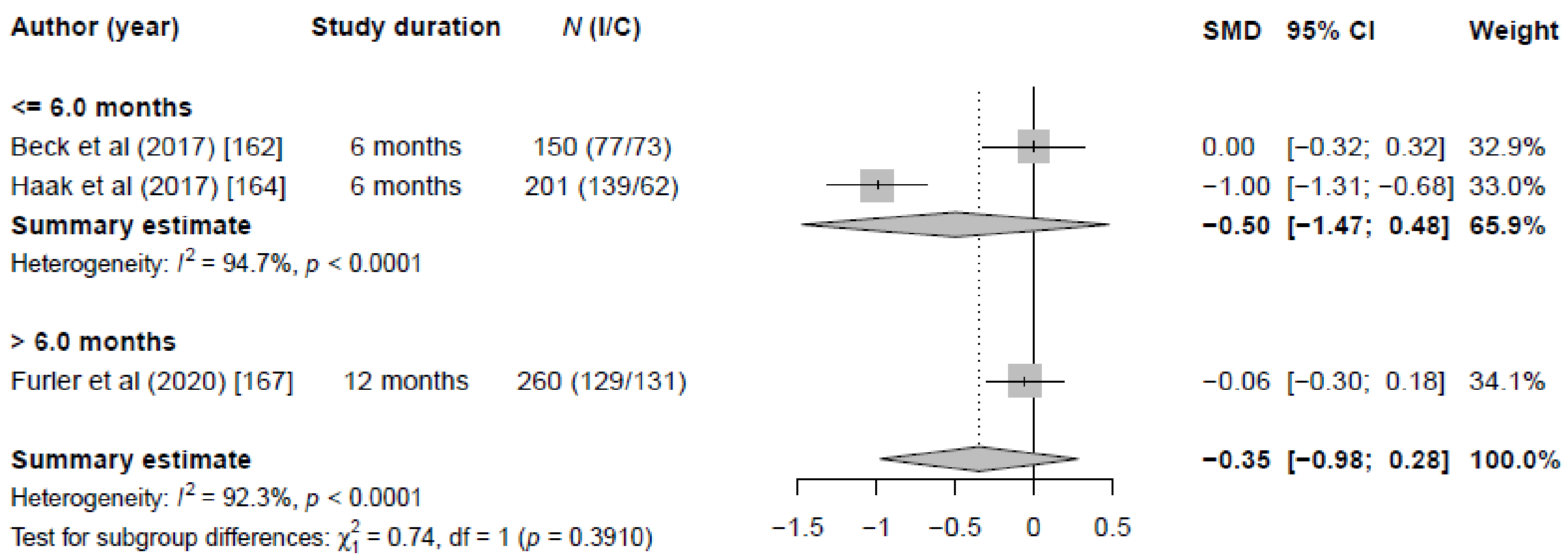


Figure 12d: SMD subgroup analysis for devices compared with usual care in type 2 diabetes stratified by trial duration. C, control; I, intervention

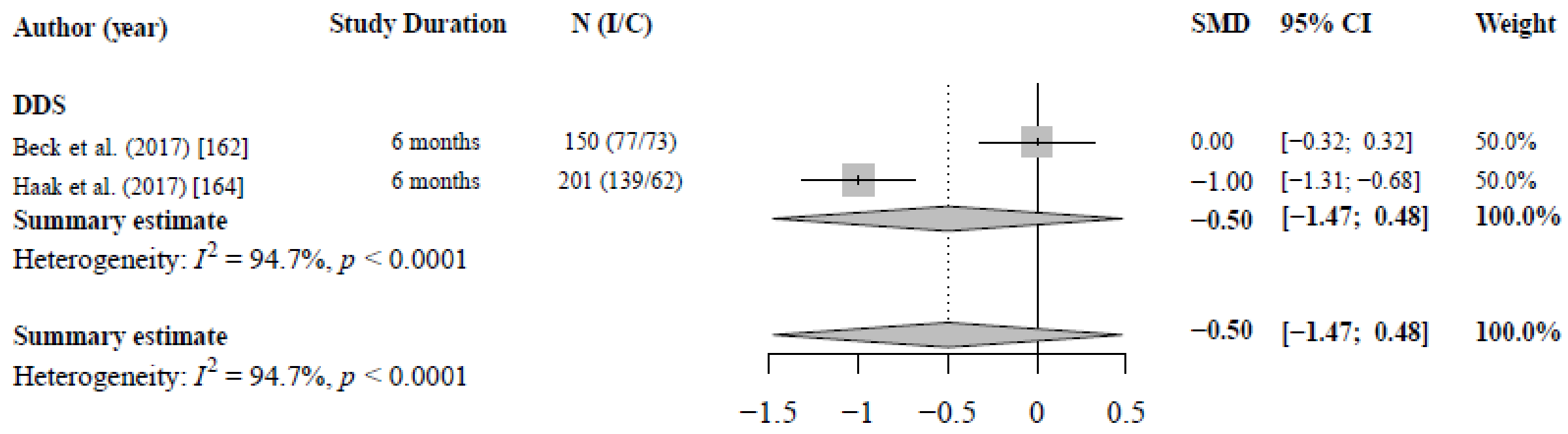


Figure 12e: SMD sensitivity analysis for devices compared with usual care in type 2 diabetes excluding cluster randomized controlled trials without reported intraclass correlation coefficient. C, control; I, intervention

Peer Support interventions compared with educational interventions for type 2 diabetes

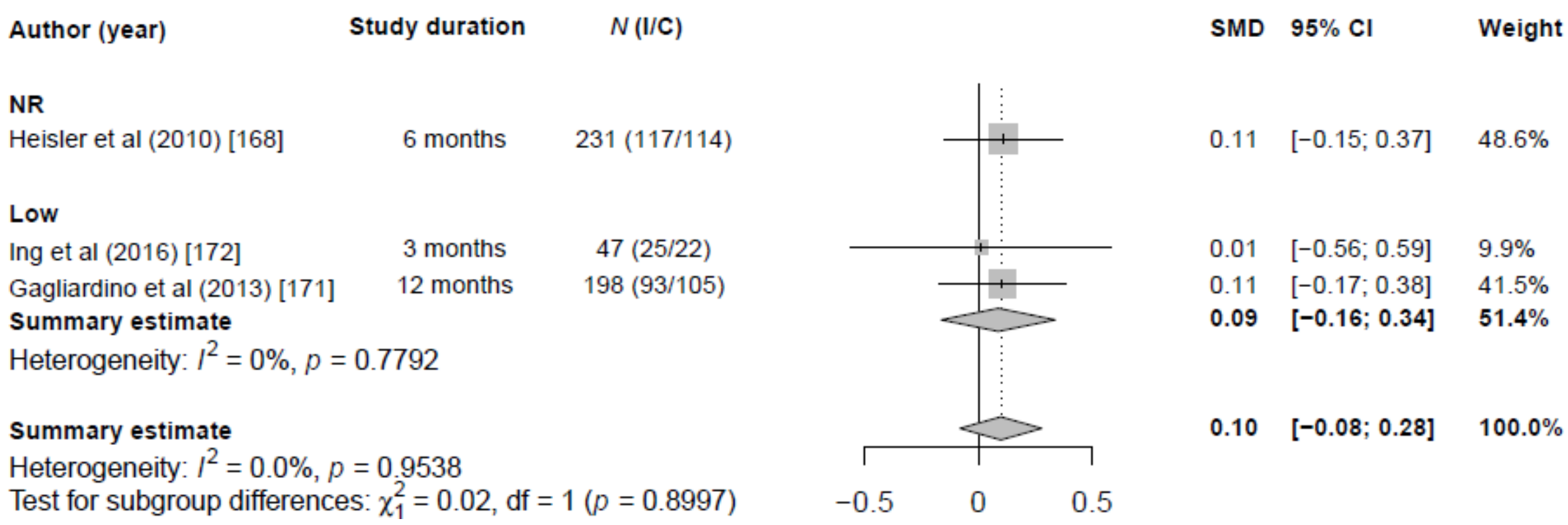


Figure 13c: SMD for peer support compared with educational interventions for type 2 diabetes stratified by baseline distress level. C, control; I, intervention

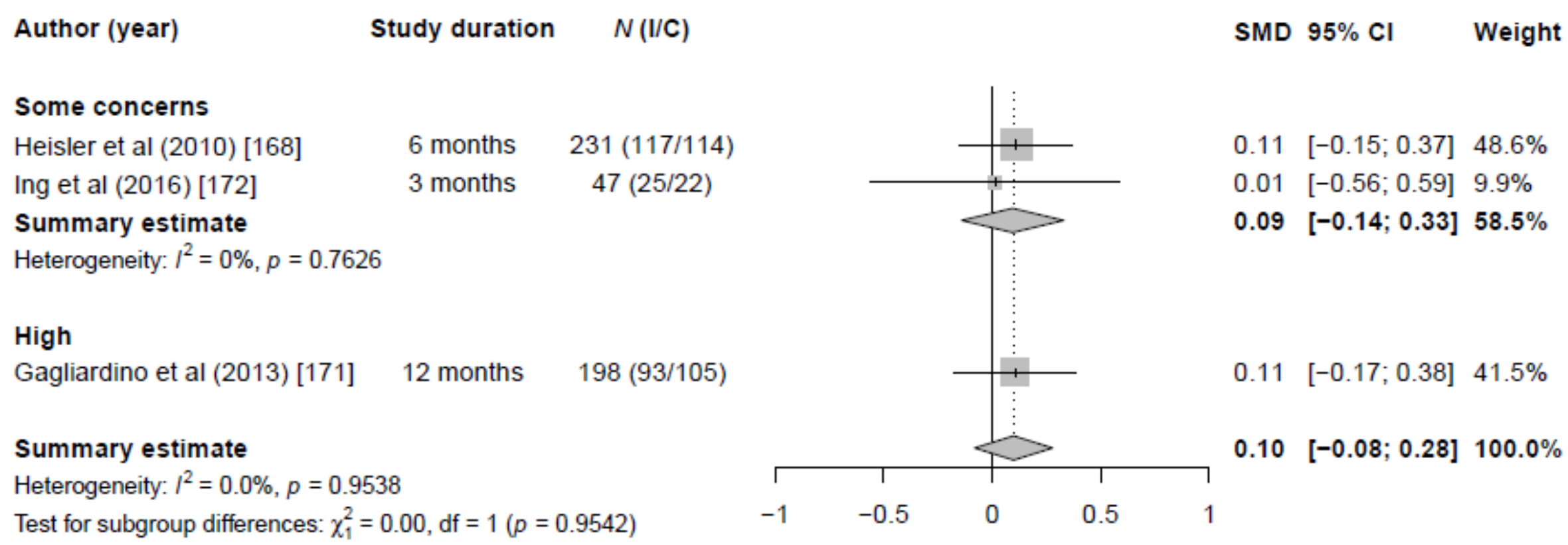


Figure 13d: SMD for peer support compared with educational interventions for type 2 diabetes stratified by risk of bias assessment. C, control; I, intervention

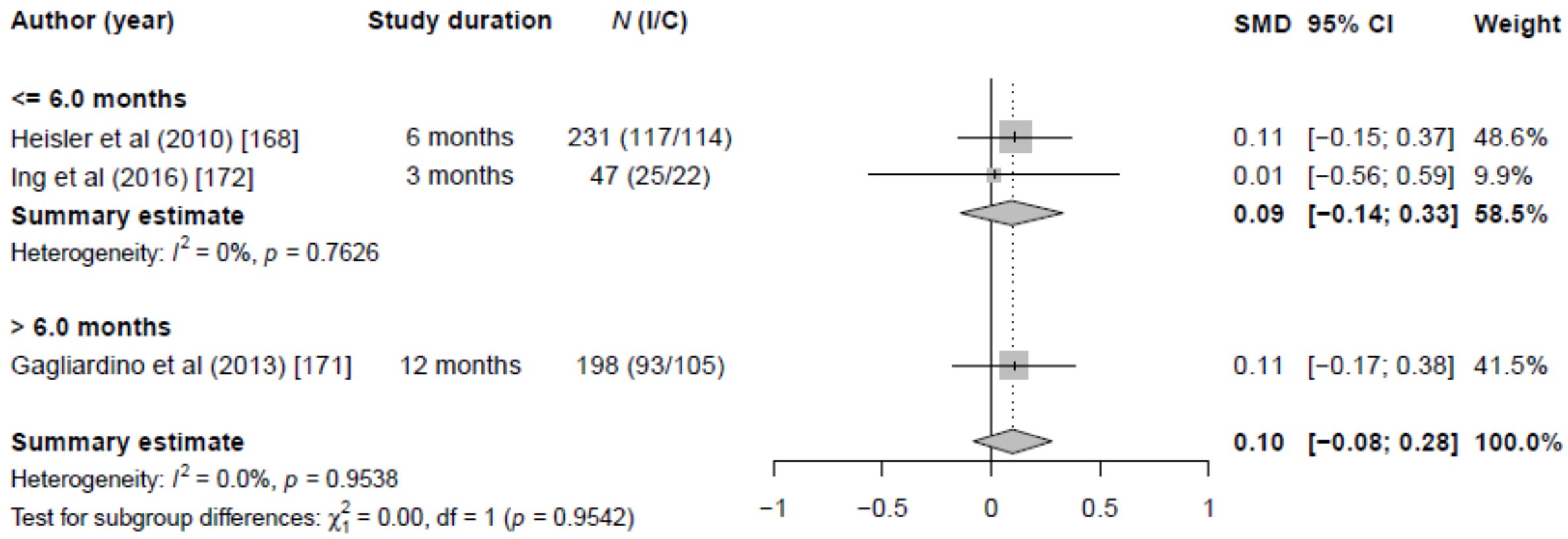


Figure 13e: SMD for peer support compared with educational interventions for type 2 diabetes stratified by trial duration. C, control; I, intervention

<u>Peer Support Interventions compared usual care for type 2 diabetes</u>

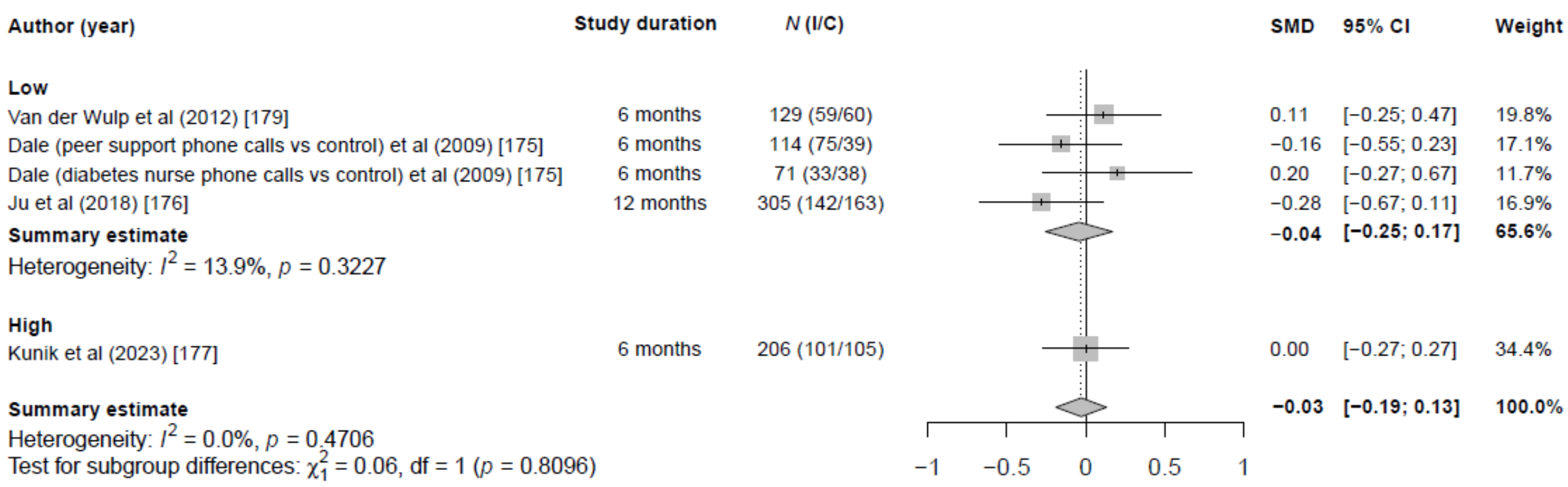


Figure 14c: SMD subgroup analysis for peer support compared with usual care for type 2 diabetes stratified by baseline distress level. C, control; I, intervention

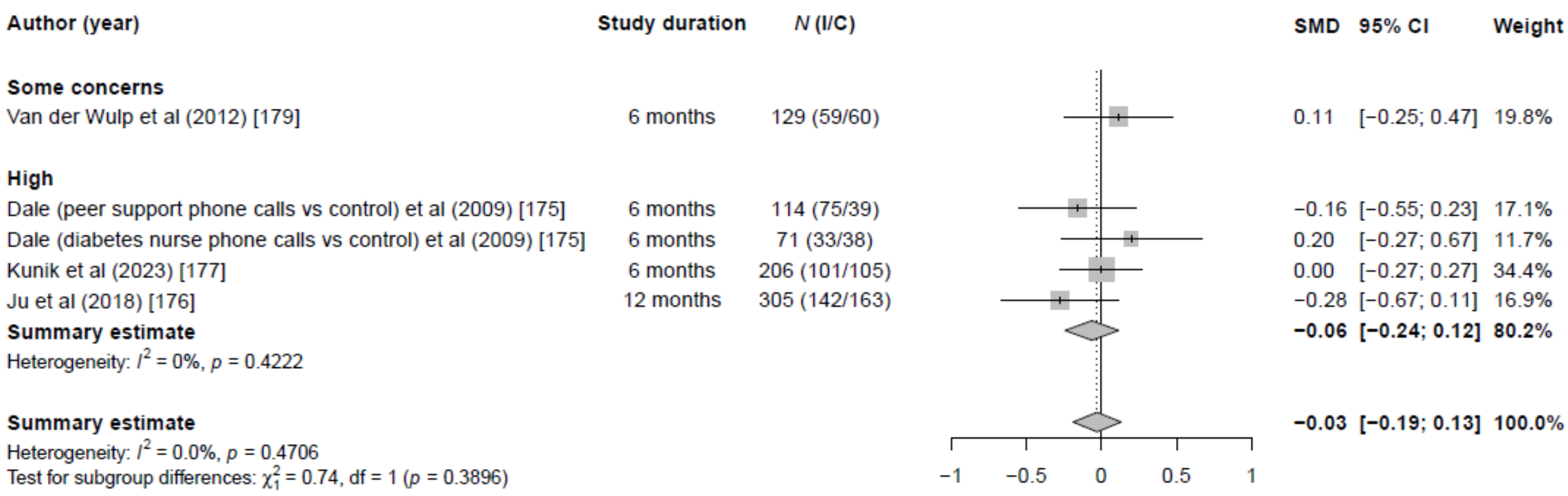


Figure 14d: SMD subgroup analysis for peer support compared with usual care for type 2 diabetes stratified by risk of bias assessment. C, control; I, intervention

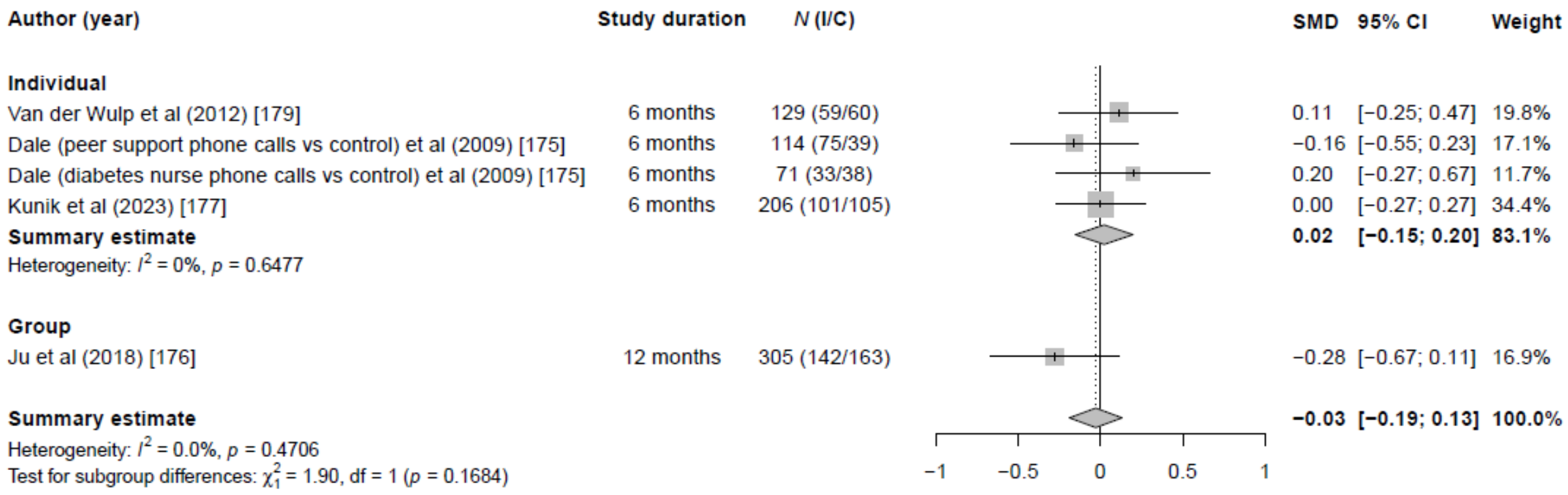


Figure 14e: SMD subgroup analysis for peer support compared with usual care for type 2 diabetes stratified by delivery format. C, control; I, intervention

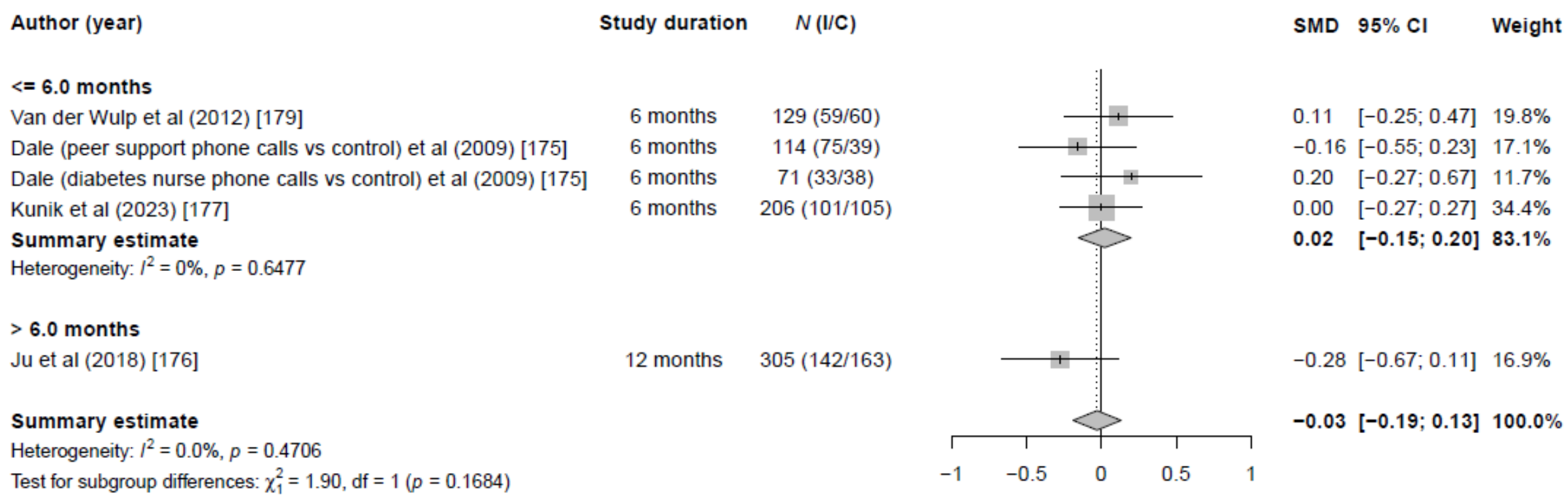


Figure 14f: SMD subgroup analysis for peer support compared with usual care for type 2 diabetes stratified by trial duration. C, control; I, intervention

<u>Educational interventions compared with usual care for type 2 diabetes</u>

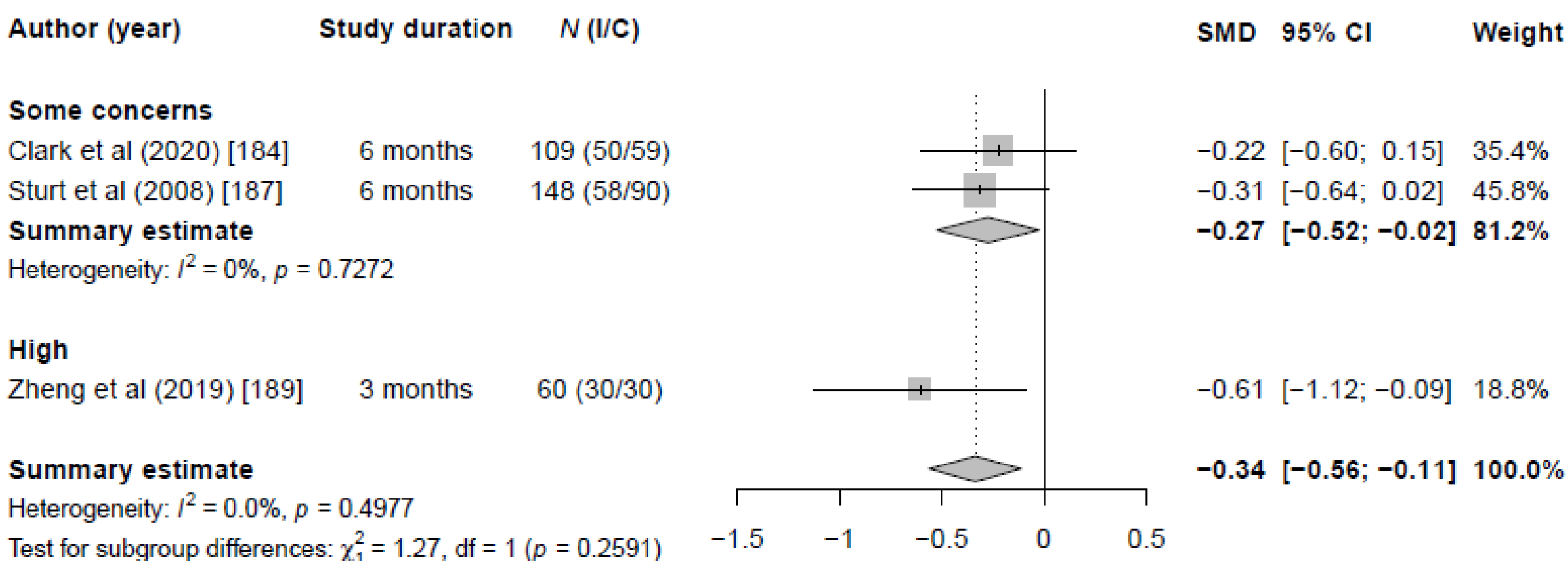

Figure 15c: SMD subgroup analysis for educational interventions compared with usual care for type 2 diabetes stratified by risk of bias assessment. C, control; I, intervention

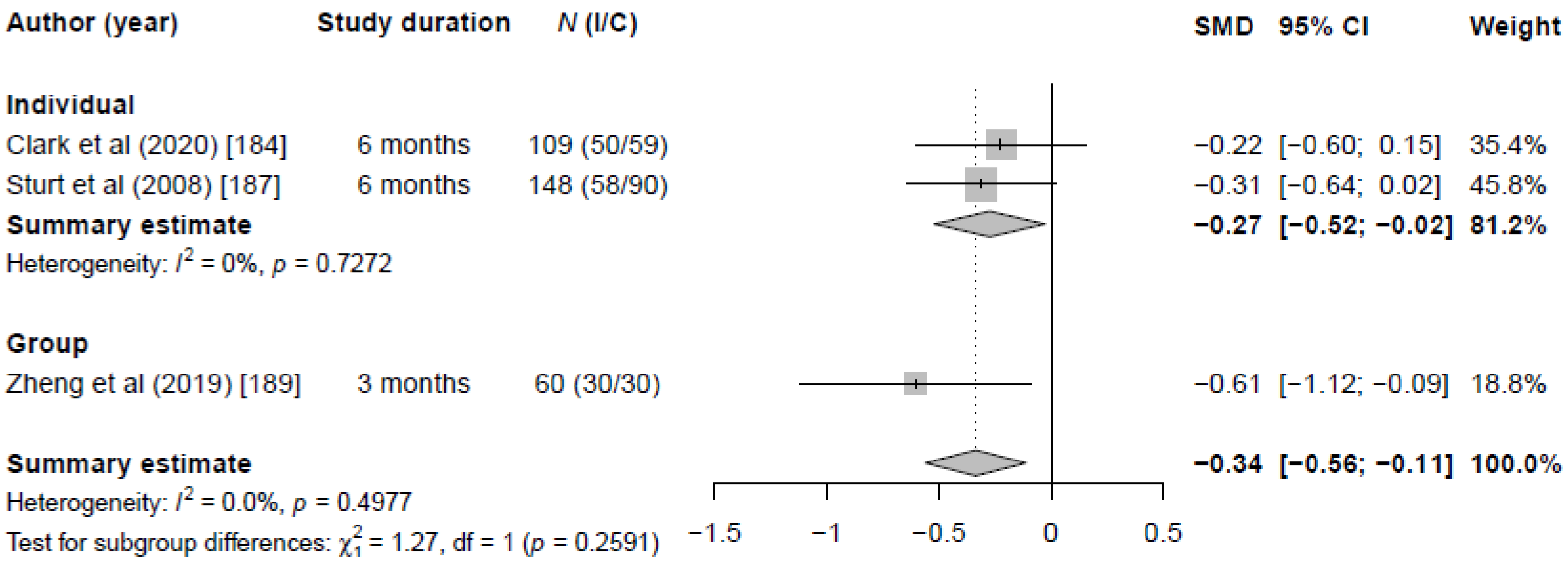


Figure 15d: SMD subgroup analysis for educational interventions compared with usual care for type 2 diabetes stratified by delivery format. C, control; I, intervention

## 6. Funnel plots

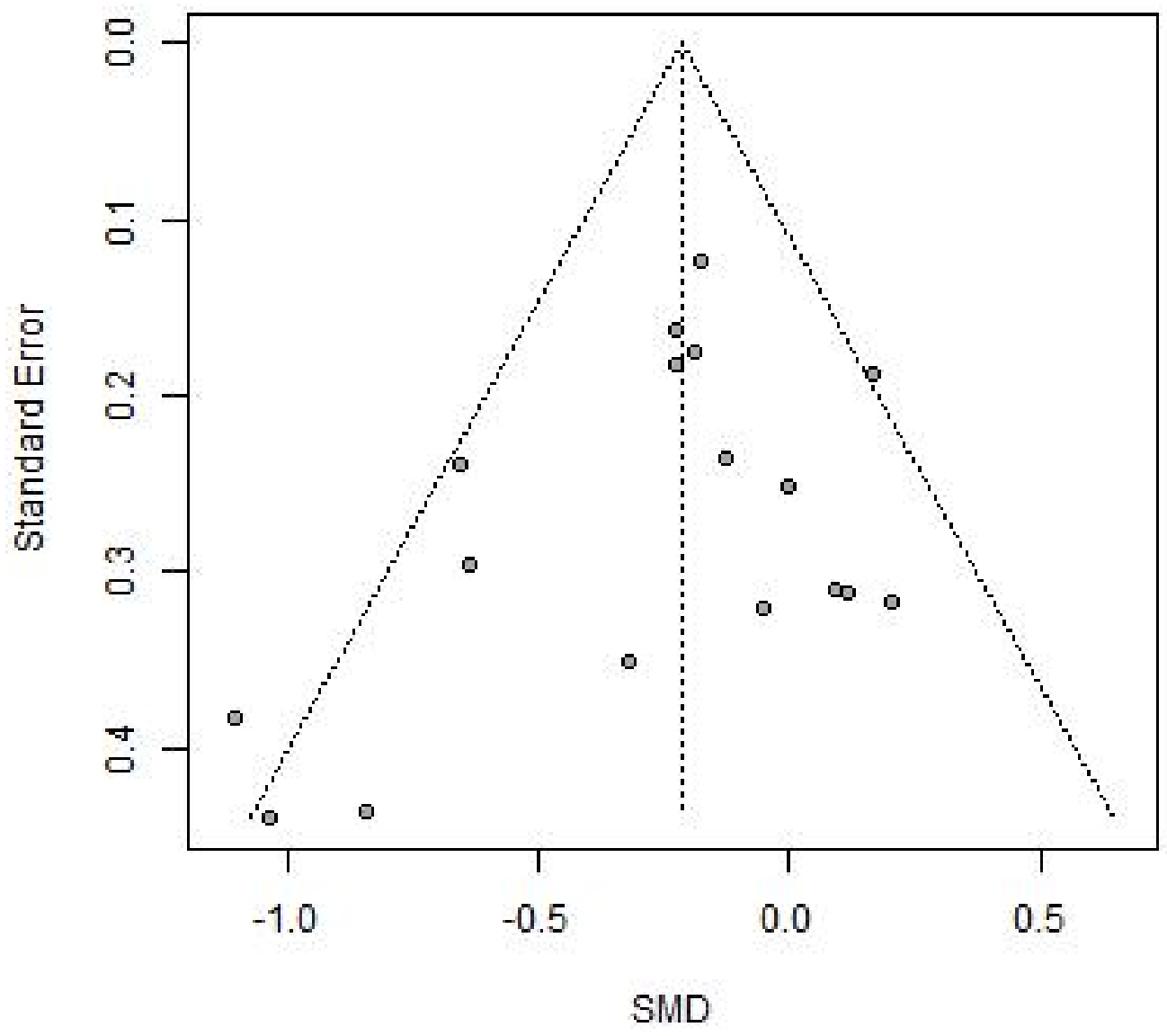


S8.1: Funnel plot psychological interventions compared with usual care for type 1 diabetes (p = 0.1782)

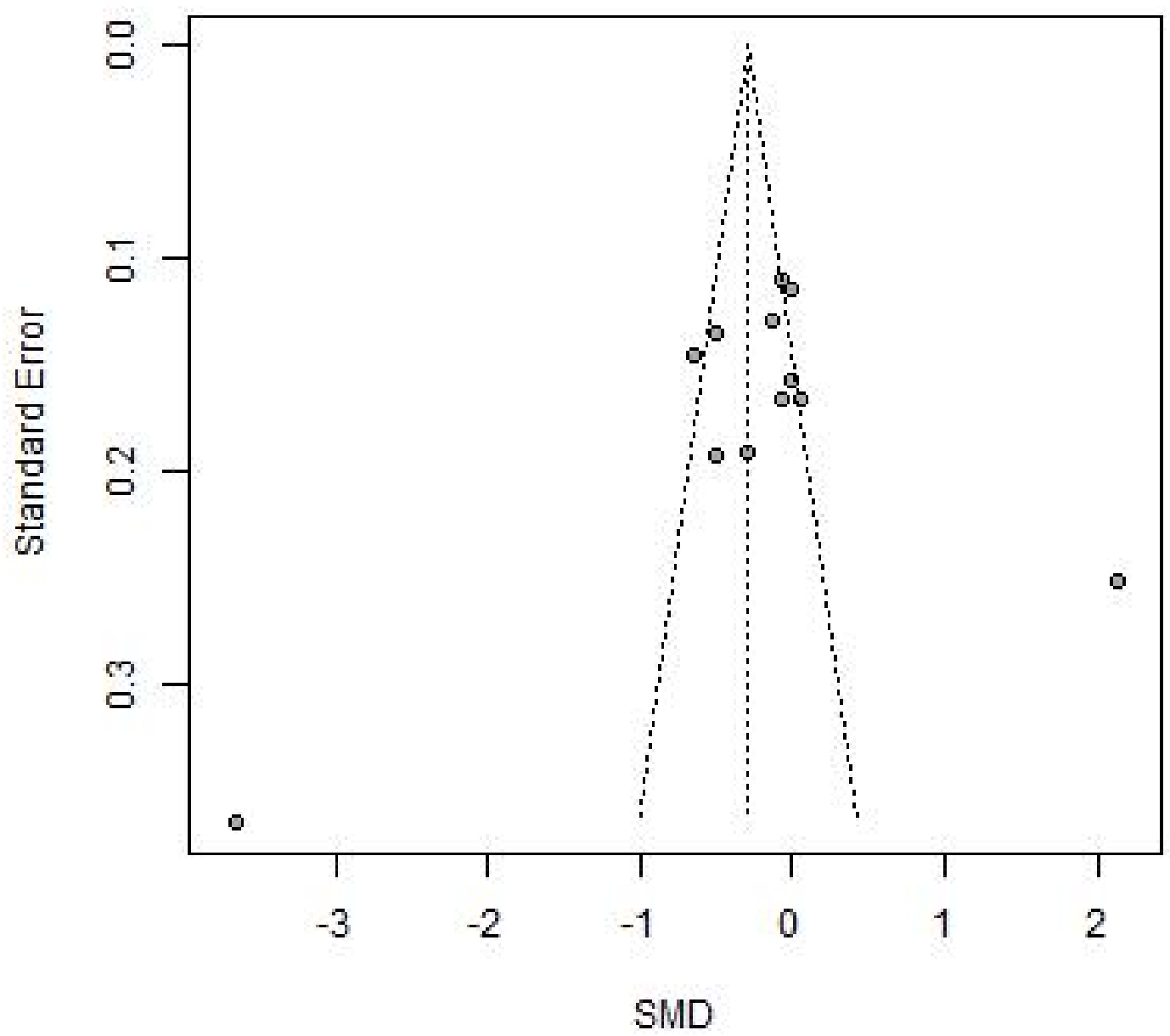


S8.2: Funnel plot psychological interventions compared with educational interventions for type 2 diabetes (p = 0.5401)

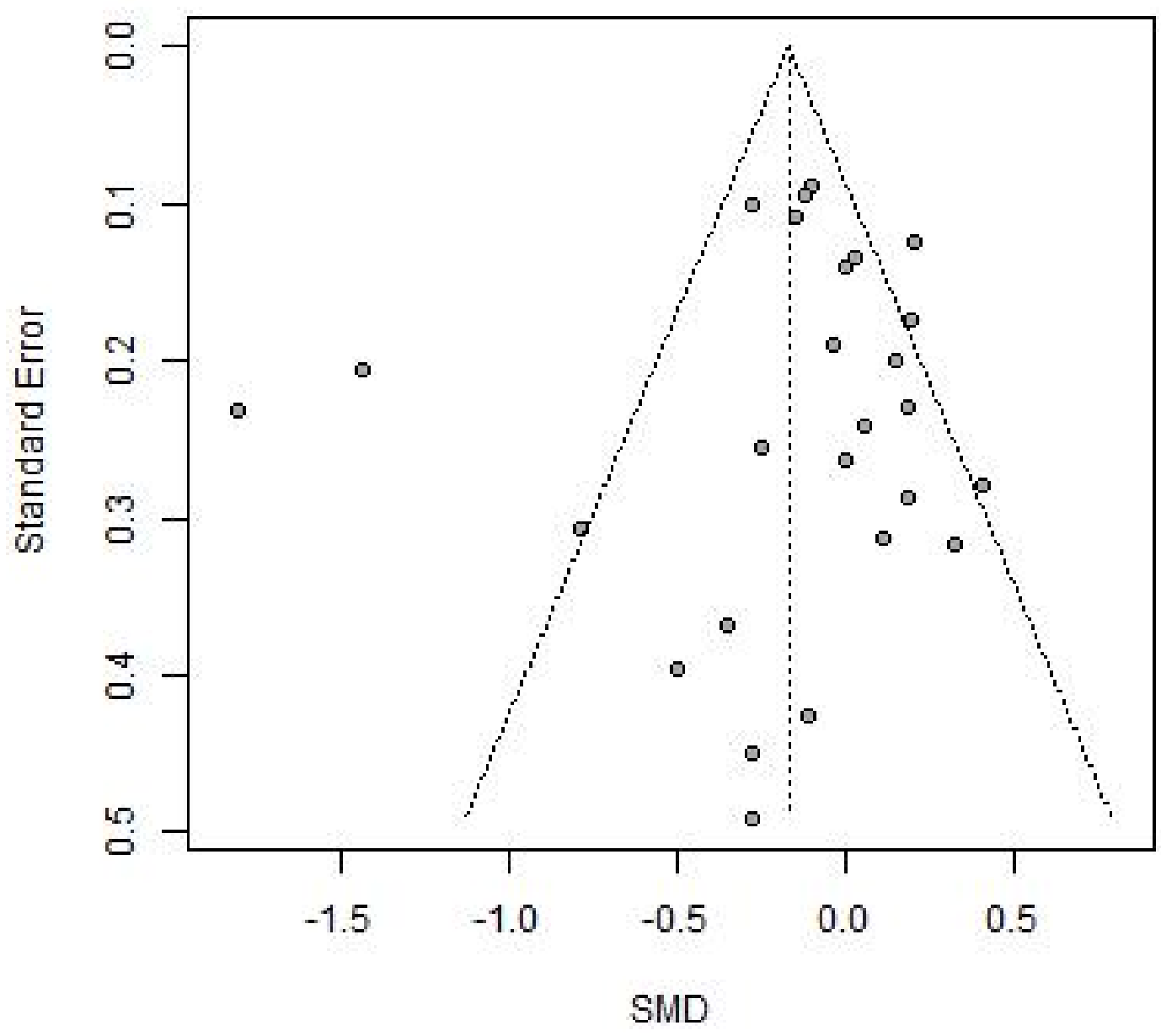


S8.3: Funnel plot psychological interventions compared with usual care for type 2 diabetes (p = 0.0209)

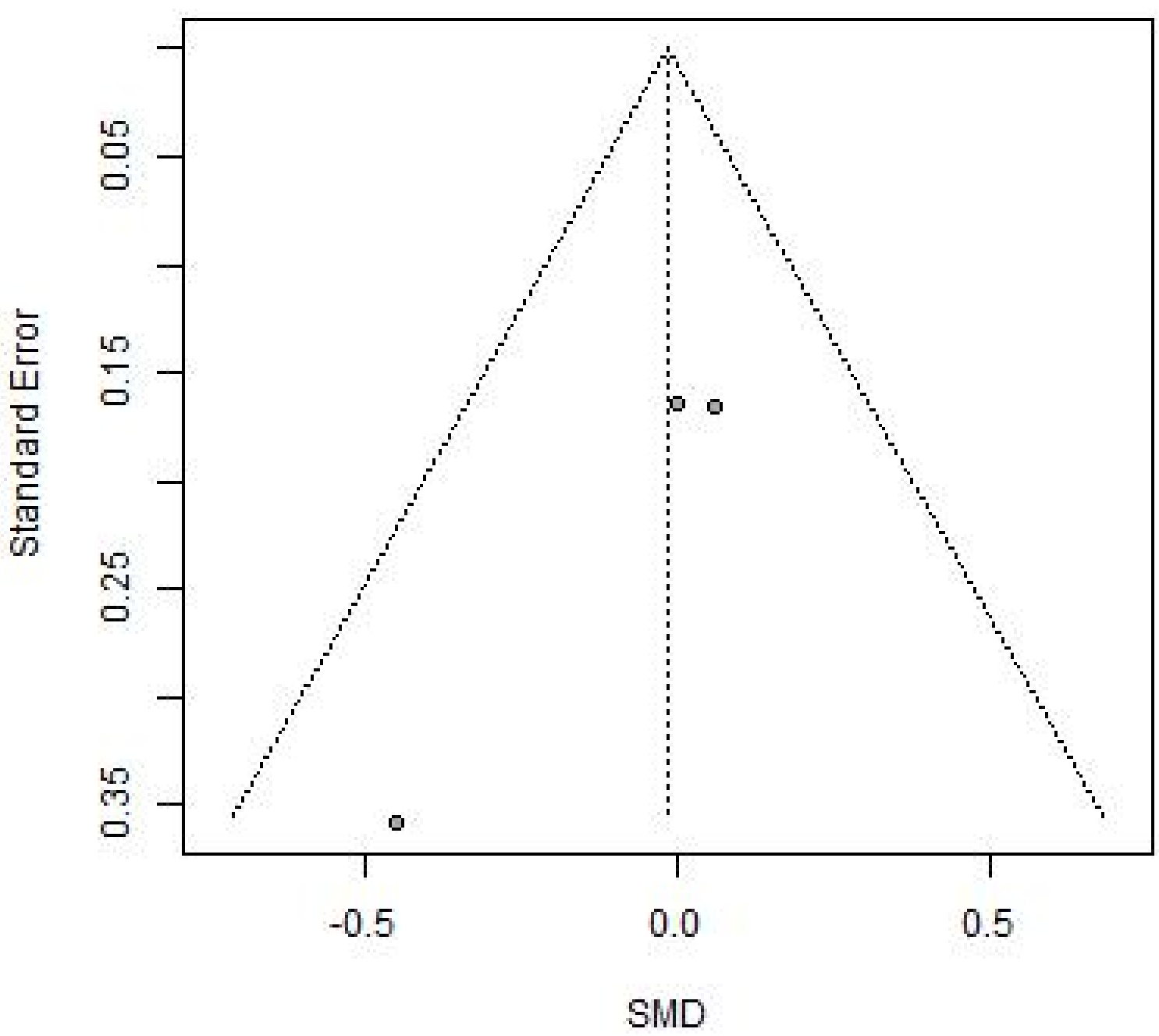


S8.4: Funnel plot psychoeducational interventions compared with educational interventions for type 1 diabetes (p = 0.1366)

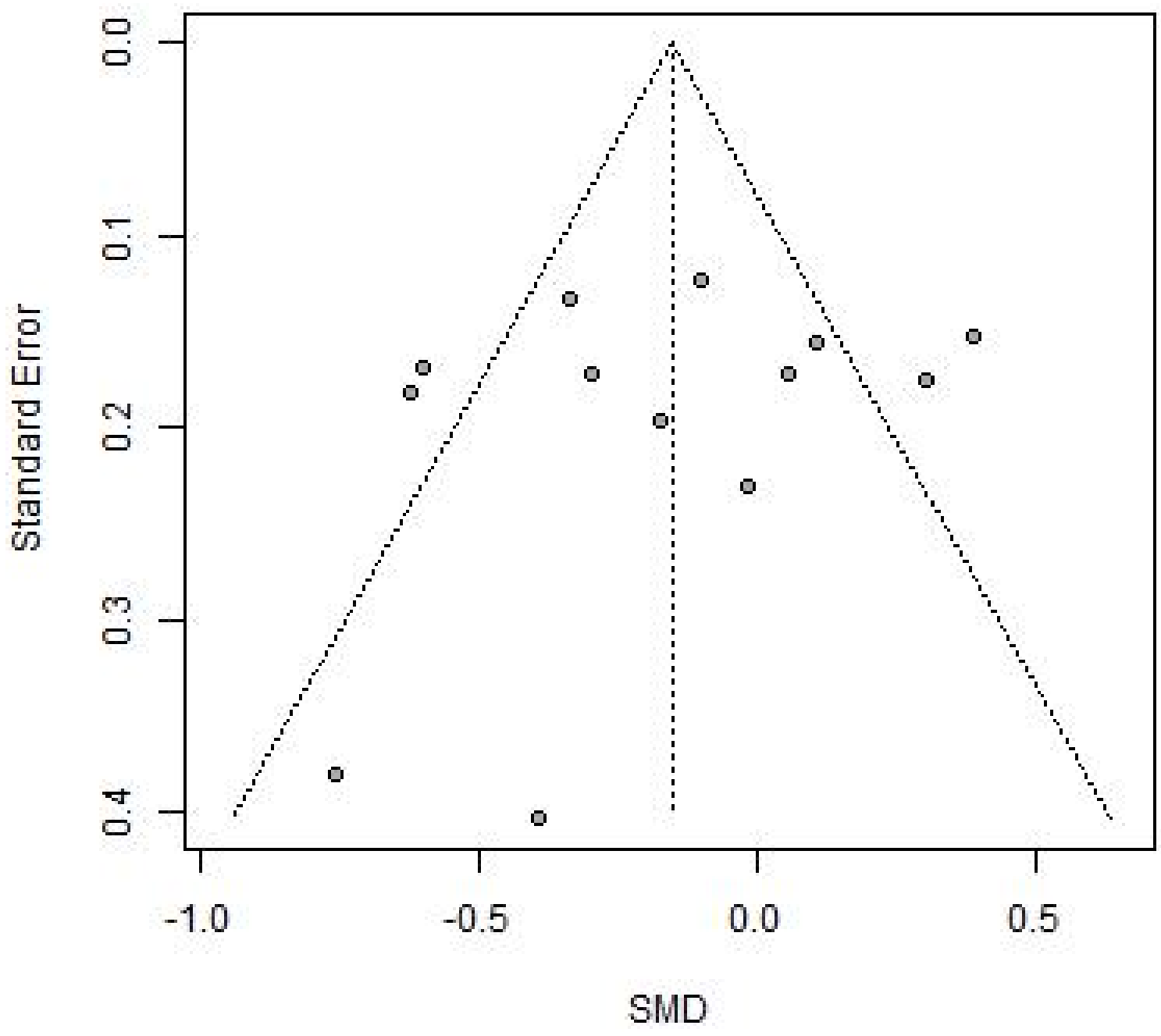


S8.5: Funnel plot psychoeducational interventions compared with educational interventions for type 2 diabetes (p = 0.4554)

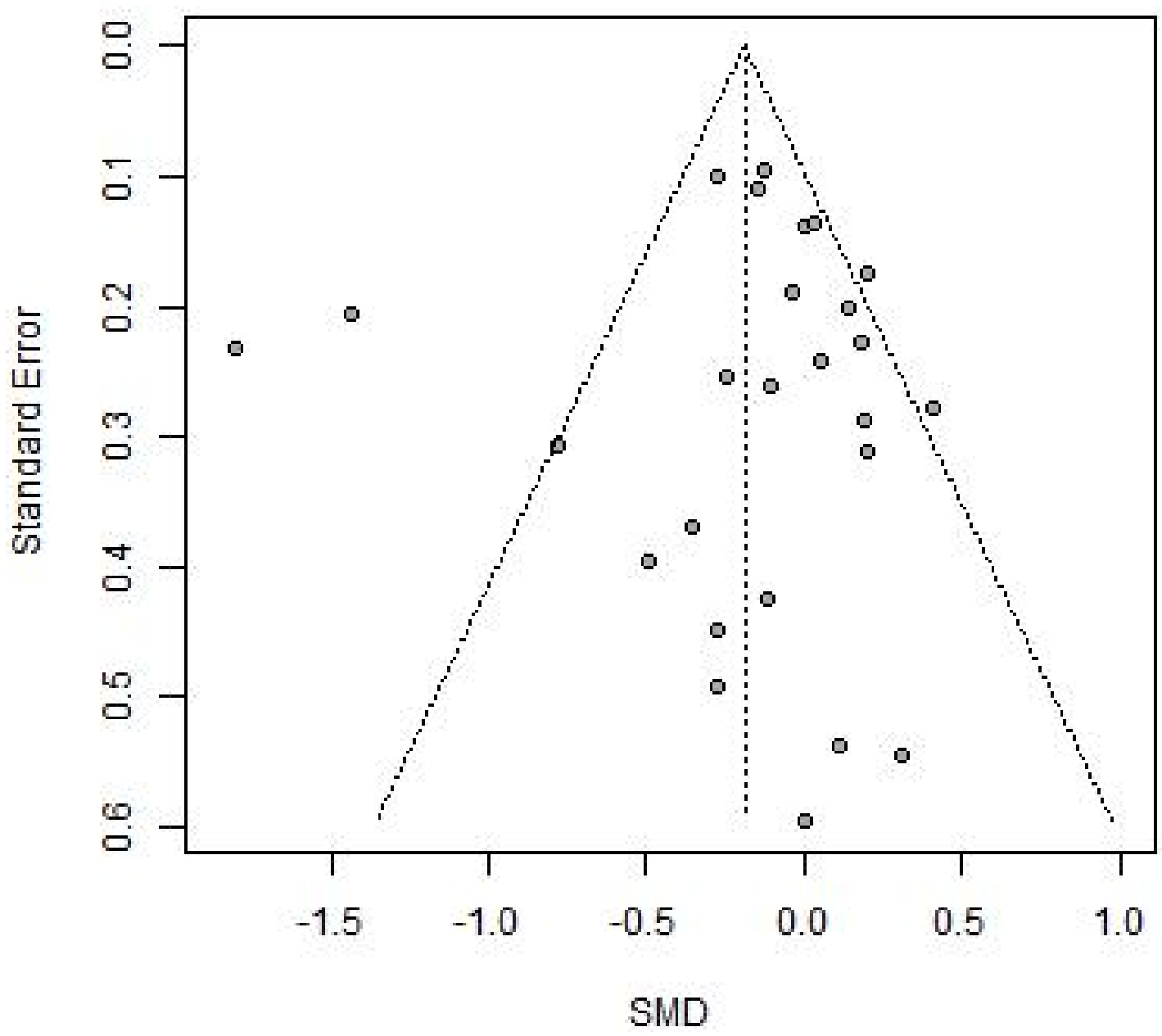


S8.6: Psychoeducational interventions compared with usual care for type 2 diabetes (p = 0.8364)

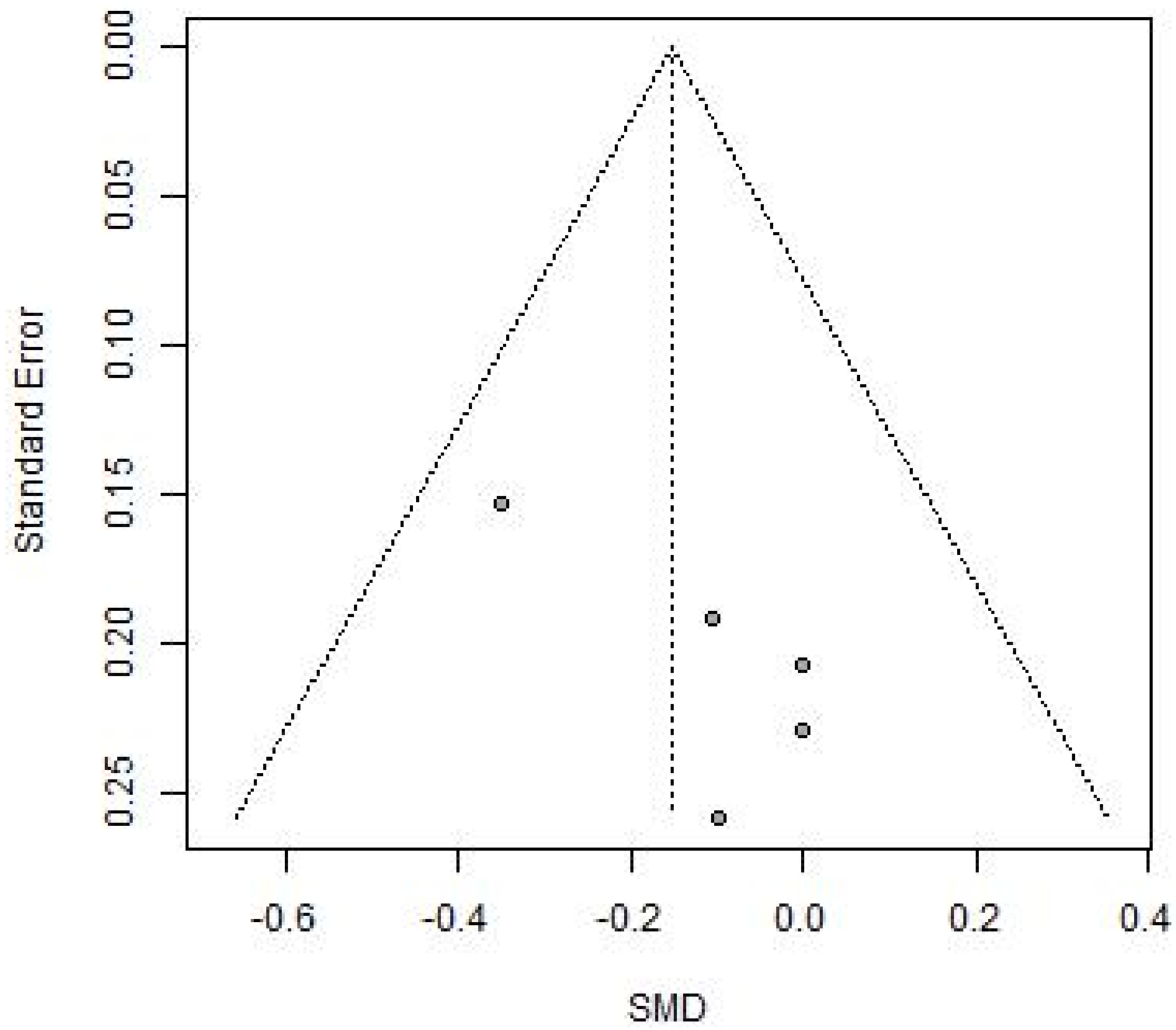


S8.7: Funnel plot AID compared with no use of AID for type 1 diabetes (p = 0.3010)

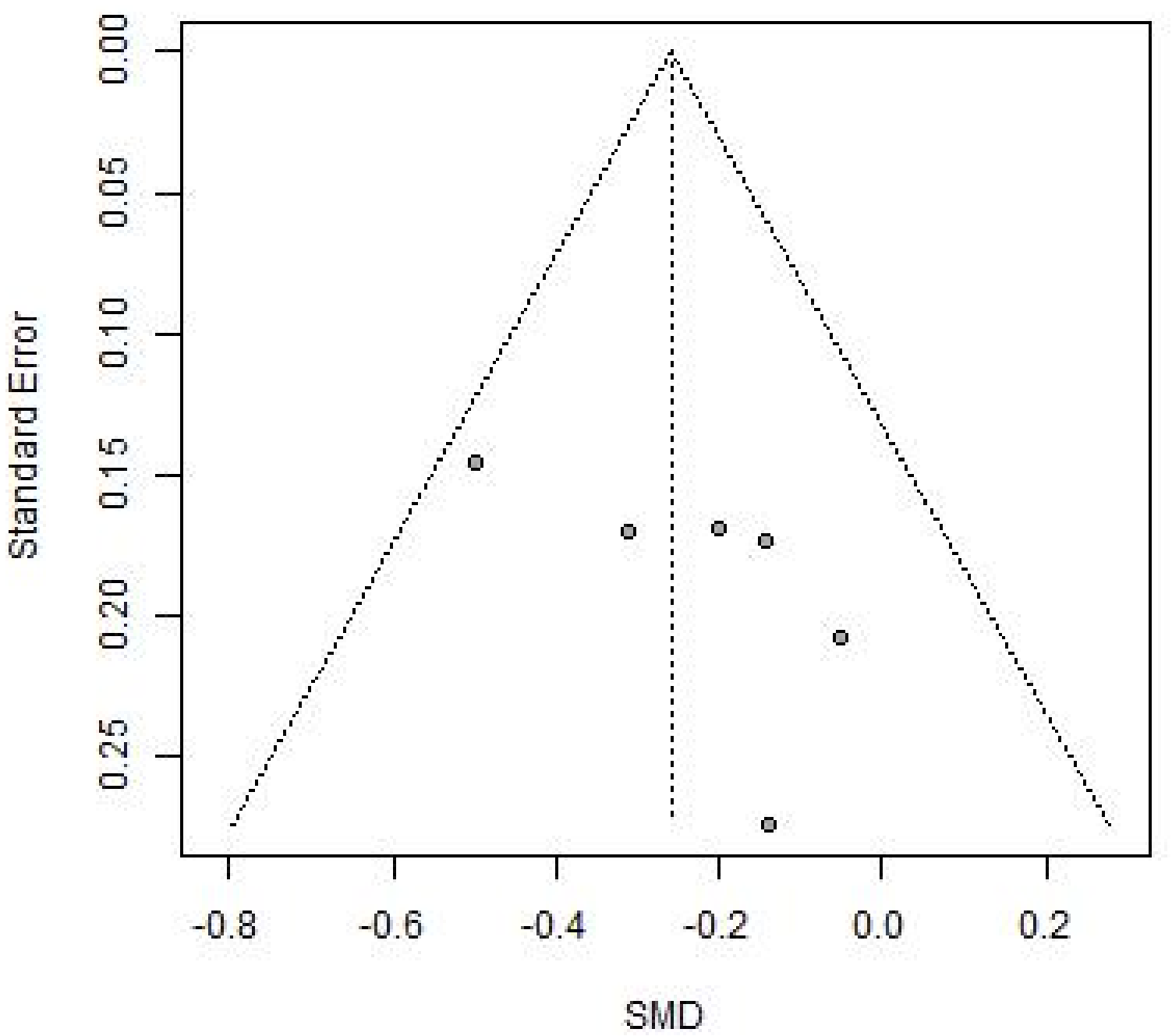


S8.8: Funnel plot CGM compared with standard blood glucose measurement for type 1 diabetes (p = 0.1396)

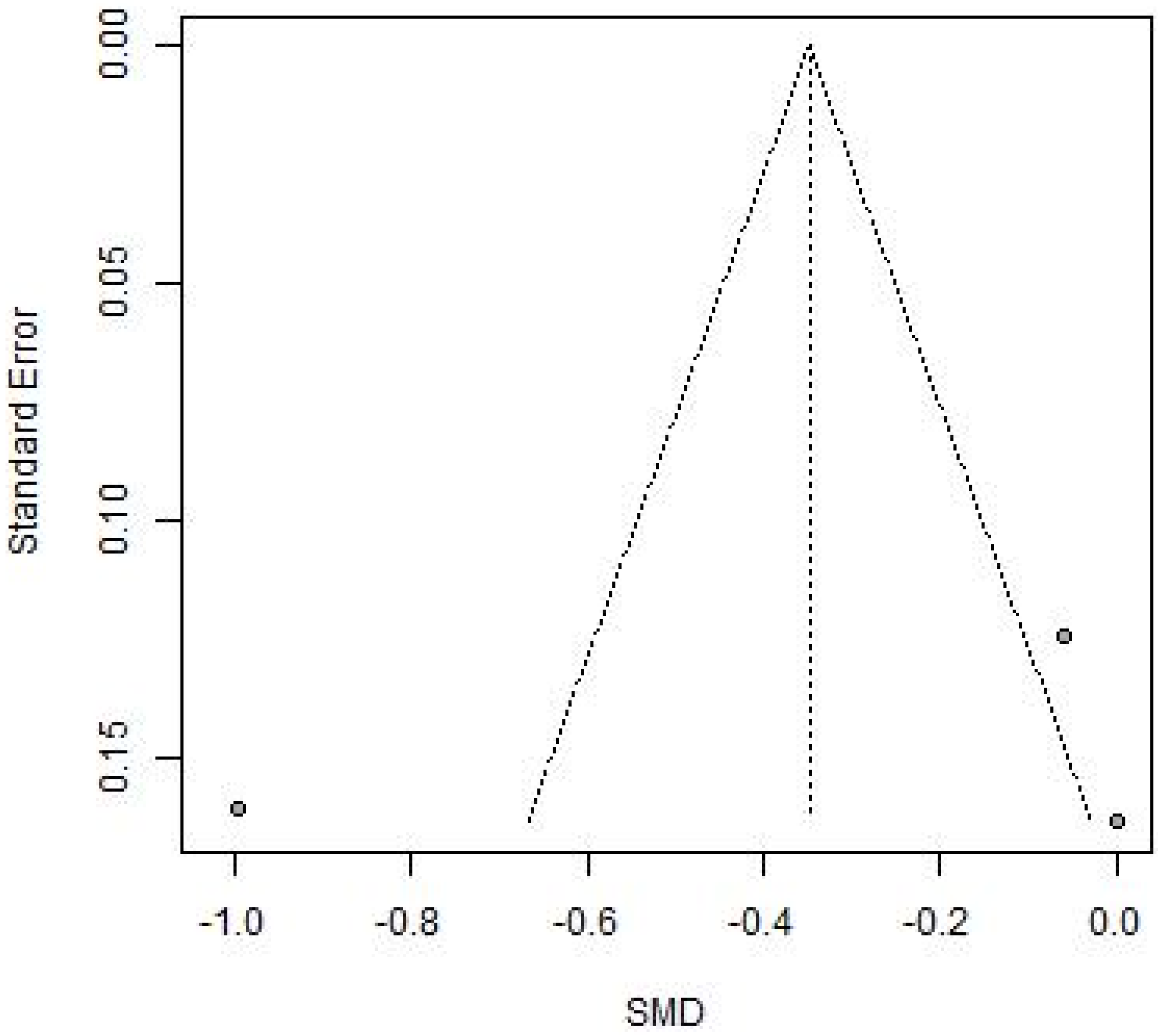


S8.9: Funnel plot devices interventions compared with usual care for type 2 diabetes (p = 0.6834)

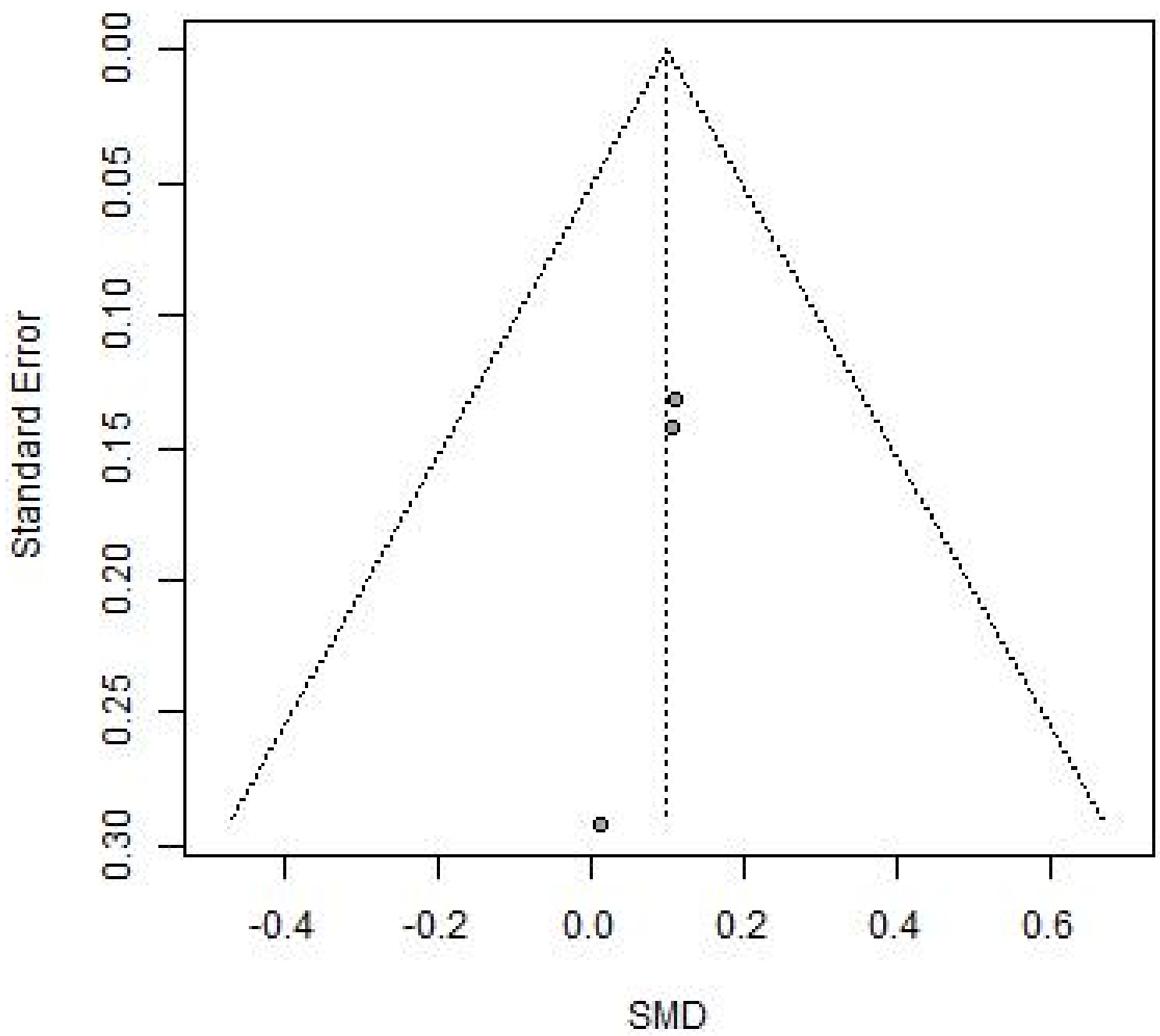


S8.10: Funnel plot peer support interventions compared with educational interventions for type 2 diabetes (p = 0.0094)

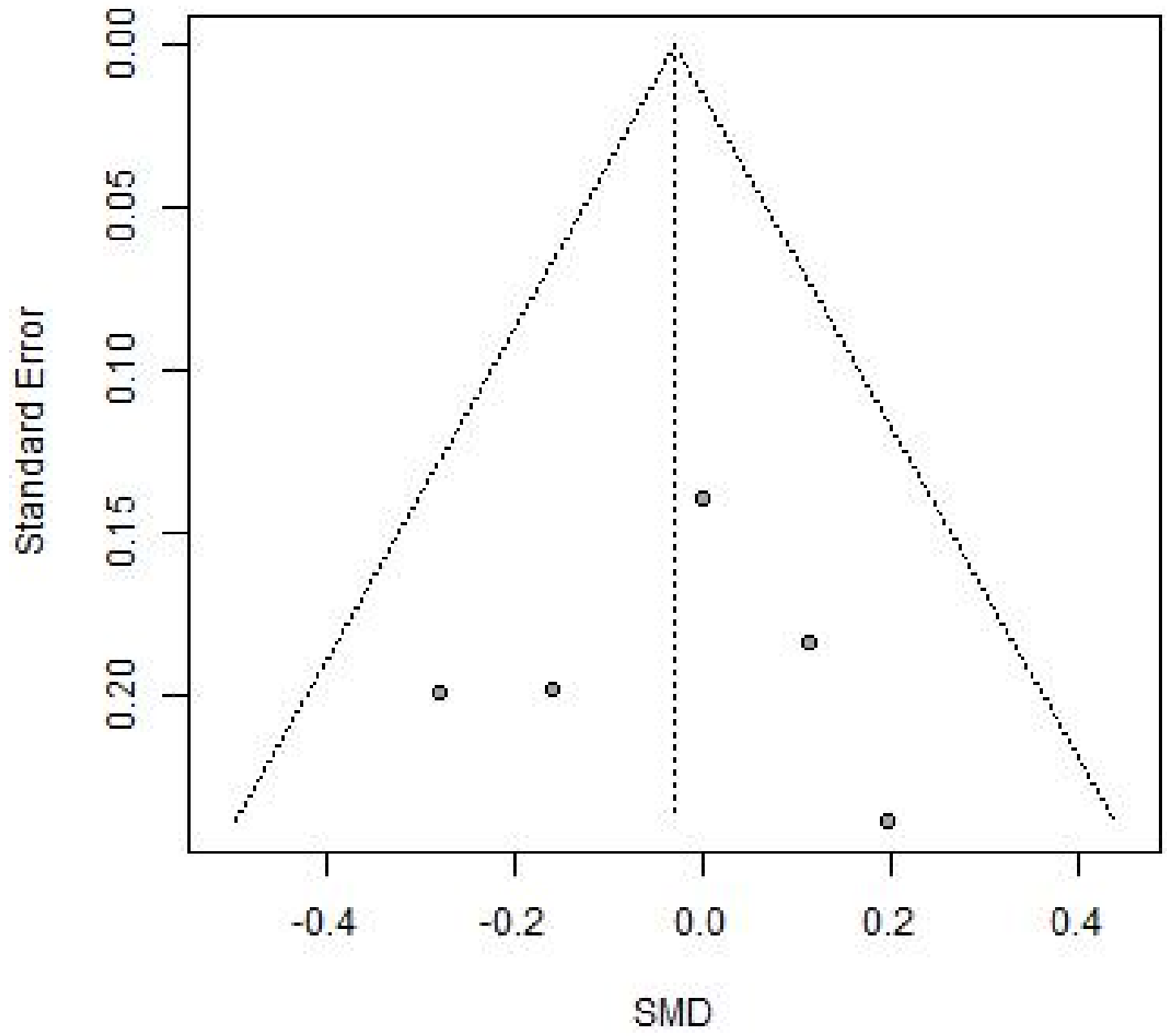


S8.11: Funnel plot peer support interventions compared with usual care for type 2 diabetes (p = 0.9819)

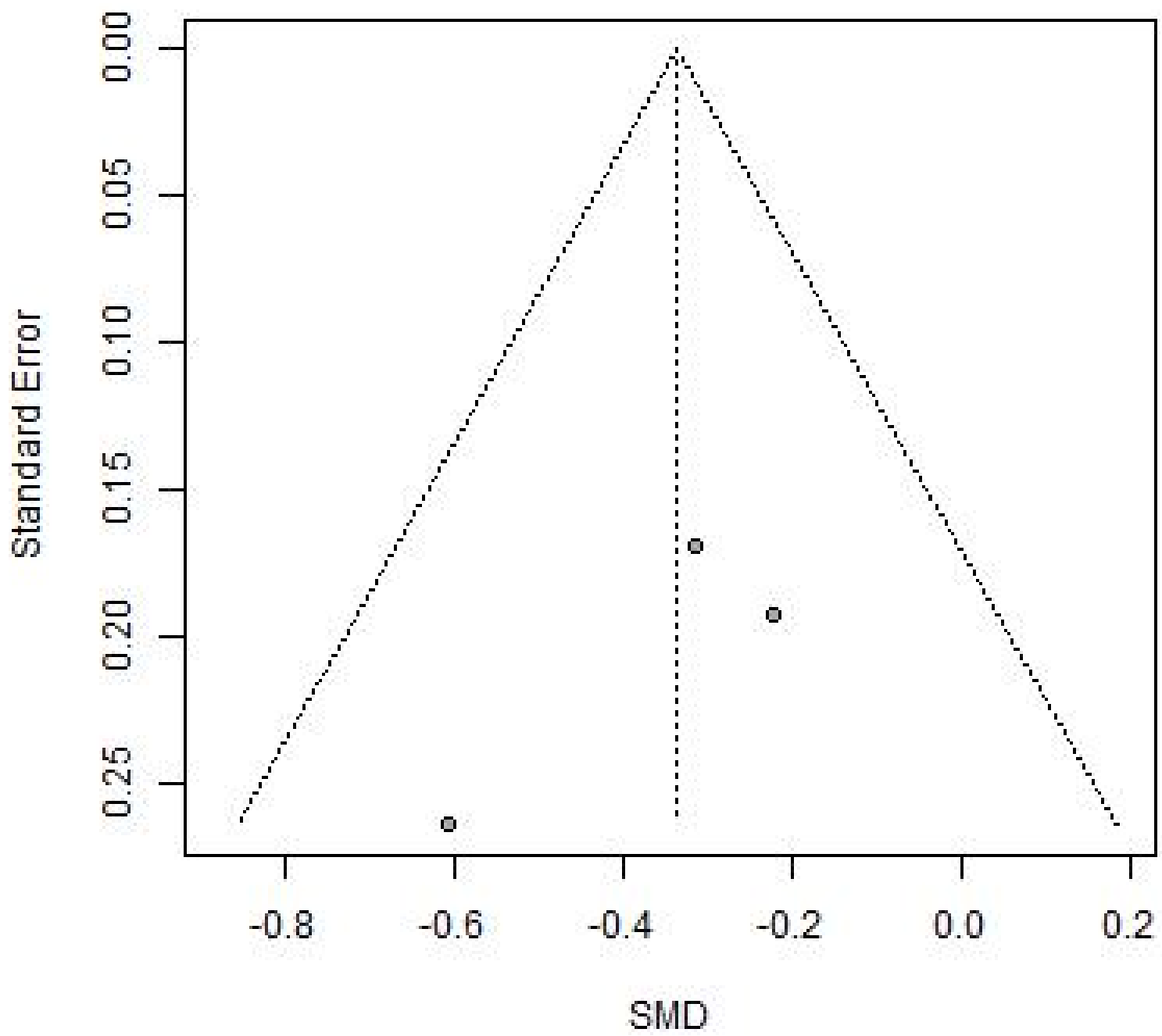


S8.12: Funnel plot educational interventions compared with usual care for type 2 diabetes (p = 0.3857)

**7. Study protocol**

# Interventions to reduce diabetes-related distress among adults with type 1 and type 2 diabetes: protocol for a systematic review and meta-analysis

Short title: **Systematic review protocol for interventions to reduce diabetes distress**

Samantha B.J. Schipper[1, 2], Sharon Remmelzwaal[1, 2], Marieke T. Blom[2, 3], Lenka Groeneveld[1, 2], Miranda Langendam[1, 4], Petra J.M. Elders[2, 3], Martin M. Jensen[5,6], Walther Jensen[7], Norbert Hermanns[8], Richard I.G. Holt[9,10], Karin Kanc[11], Thomas Karagiannis[12], Michelle Law[7], Andreia Mocan[13], Jane Speight[14,15], Jackie Sturt[16], Francesco Zaccardi[17], Joline W. Beulens[1,2] and Femke Rutters[1,2*]

*[1] Department of Epidemiology and Data Science, Amsterdam UMC, Amsterdam, the Netherlands*
*[2] Amsterdam Public Health Research Institute, Amsterdam UMC, location VUmc, Amsterdam, the Netherlands [3] Department of General Practice, Amsterdam UMC, Amsterdam, the Netherlands*
*[4] Amsterdam Public Health Research Institute, Methodology programme, Amsterdam, the Netherlands*
*[5] Department of Public health Aarhus University, Aarhus, Denmark*
*[6] Public Health Research, DEFACTUM, Aarhus, Denmark*
*[7] European Association for the Study of Diabetes, Dusseldorf, Germany*
*[8] University of Bamberg and Research Institute of the Diabetes Academy Mergentheim (FIDAM), Bad Mergentheim, Germany*
*[9] Human Development and Health, Faculty of Medicine , University of Southampton, Southampton, UK*
*[10] National Institute for Health Research Biomedical Research Centre, University Hospital Southampton NHS Foundation Trust, Southampton, UK*
*[11] Jazindiabetes (Diabetes & Me), Diabetes Centre, ,Ljubljana Slovenia*
*[12] Clinical Research and Evidence-Based Medicine Unit, Second Medical Department, Aristotle University of Thessaloniki, Thessaloniki, Greece*
*[13] Center for Diabetes, Nutrition and Metabolic Diseases, Emergency Clinical County Hospital, Cluj-Napoca, Romania*
*[14] School of Psychology, Institute for Health Transformation, Deakin University, Geelong, VIC, Australia*
*[15] The Australian Centre for Behavioural Research in Diabetes, Carlton, VIC, Australia*
*[16] Florence Nightingale Faculty of Nursing, Midwifery & Palliative Care, King's College London, London, UK*
*[17] Leicester Real World Evidence Unit, Leicester Diabetes Centre, University of Leicester, Leicester, UK*

* Corresponding author: Femke Rutters, Amsterdam UMC, Department of Epidemiology and Data Science, Amsterdam, the Netherlands

f.rutters@amsterdamumc.nl

ORCID identifier per author

Samantha B.J. Schipper: 0009-0008-6432-3497

Sharon Remmelzwaal: 0000-0003-3399-5836

Marieke Blom: 0000-0002-8088-1105

Lenka Groeneveld: 0000-0002-2901-6677

Mariska Langendam: 0000-0001-9038-3209

Petra J.M. Elders: 0000-0002-5907-7219

Martin M. Jensen: 0000-0001-7603-2007

Walter Jensen: 0009-0008-3974-3051

Norbert Hermanns: 0000-0002-2903-2677

Richard Holt: 0000-0001-8911-6744

Karin Kanc: 0000-0002-7237-6782

Thomas Karagiannis: 0000-0001-5242-0574

Michelle Law: 0009-0007-1469-0320

Andreia Mocan: 0000-0002-7678-1048

Jane Speight: 0000-0002-1204-6896

Jackie Sturt: 0000-0003-1281-1401

Francesco Zaccardi: 0000-0002-2636-6487

Joline W. Beulens: 0000-0002-4181-0937

Femke Rutters: 0000-0002-1008-678X

Publication note:
This protocol was previously published in *Systematic Reviews (2026)*. DOI: 10.1186/s13643-026-03292-w

Word count: 4094 including abstract

**Abstract**

**Background** Diabetes distress (DD) refers to the emotional and psychological burden experienced by individuals living with diabetes. Randomized controlled trials (RCTs) have investigated the efficacy of interventions on DD among adults with type 1 diabetes or type 2 diabetes. We aimed to systematically identify, summarize and critically appraise all available evidence from RCTs assessing the efficacy of interventions for reducing DD (either as primary or secondary outcome) among adults with type 1 diabetes or type 2 diabetes.

**Methods:** Four electronic databases (PubMed, Cochrane Database of Systematic Reviews, PsycINFO and CINAHL) were searched from inception until 23 September 2024. Retrieved papers were screened for eligibility by two independent reviewers, who also screened the reference lists of all included publications. Studies were included if the study was an RCT performed in an adult population (≥18 years), in which the efficacy of an active intervention on DD as primary or secondary outcome was described. Data were extracted using an a priori developed form. Two independent reviewers assessed the risk of bias of included studies using the Cochrane RoB 2 tool for RCTs. For each type of intervention, a narrative data synthesis was conducted, and, if possible, meta-analyses were conducted when two or more studies reported on the same outcome. Heterogeneity between studies was assessed using the $I^2$ statistic and a certainty of evidence assessment (GRADE) was conducted. In case of substantial heterogeneity, subgroup analysis was conducted.

**Discussion:** Data regarding the efficacy of interventions on DD was summarized and discussed. Future implications and recommendations for clinical care was presented. This systematic review and meta-analysis served as the basis for a European Association for the Study of Diabetes (EASD) guideline on the management of DD.

**Registration** This protocol was submitted for registration with the International Prospective Register of Systematic Reviews (PROSPERO) on 10 October 2024 (registration number CRD42024598512).

**Keywords** Diabetes distress, type 1 diabetes, type 2 diabetes, randomized controlled trials, systematic review, meta-analysis

## Introduction

Diabetes distress (DD) refers to the emotional and psychological burden experienced by individuals living with diabetes [1]. It encompasses a wide range of negative feelings, including frustration, anxiety, guilt, and fear attributed to the challenges of living with and managing diabetes. DD is distinct from clinical depression and anxiety, which are generic mood disorders [2]. Factors that contribute to DD can include worries about acute and long-term complications, frustrations with managing blood glucose, the burden of self-care, social interactions (including with health professionals), financial burden of diabetes and its management, feelings of isolation or overwhelm, stigma, and not feeling 'in control' of the condition [3]. Qualitative studies indicate that experiences of DD may be caused by threatened autonomy, helplessness and negative sense of self [4].

The concept of DD was introduced in 1995, with the development and publication of the 20-item Problem Areas In Diabetes (PAID) scale [1]. Since then, several other validated questionnaires have been developed to assess DD, such as the 17-item Diabetes Distress Scale (DDS-17) [5], and various derivatives now exist, including short-form measures (e.g. PAID-5, PAID-11, DDS-2) and versions developed for specific populations, e.g. for adults with type 1 diabetes [6-8]. Each questionnaire has a slightly different focus [9]. The prevalence of DD varies greatly across populations (e.g. by age, diabetes type, diabetes duration, treatment type, presence of complications), by study setting, and by the instrument used. Reviews estimate that 20-36% of all people with diabetes experience elevated DD [10-12].

Research indicates a bidirectional association between DD and glycaemic indicators, which suggests that greater DD is associated with higher HbA1c (a measure of average blood glucose), less time in range (TIR), higher fasting glucose and vice-versa [13], although glucose in target does not necessarily indicate the absence of DD [14]. In addition, DD is associated with less engagement in managing glucose (medications, monitoring) and healthy self-care behaviours. Consequently, people with DD have greater risk of diabetes-related complications [13]. Thus, addressing DD is not only beneficial for the person's emotional and mental health, but there is compelling evidence that it is crucial for enabling optimal diabetes management and physical health [13].

While DD is a significant concern for people with diabetes, their healthcare professionals lack detailed guidance on the best management strategies and interventions for use in clinical practice. To date, previous systematic reviews have focused on specific types of interventions or included only studies primarily designed to reduce DD [15-20]. Several interventions (varying in type, structure,

format, duration, etc.) have demonstrated efficacy for reducing DD. These include cognitive-behavioural therapy, mindfulness-based stress reduction, structured diabetes education, peer support programs and the introduction of new treatments and technologies [15-20]. However, evidence suggests that interventions not specifically targeting DD may reduce DD as a by-product. For instance, a randomized controlled trial (RCT) assessing the efficacy of an implantable sensor in people with type 1 diabetes resulted in lower DD, compared to a transcutaneous sensor [21]. Moreover, among adults with type 2 diabetes, an RCT showed that gastric band surgery is associated with equivalent improvement in DD to medical management [22].

In 2016, the American Diabetes Association (ADA) provided an evidence-based guideline for psychosocial assessment and care in people with diabetes, focussing on the most common psychological factors affecting those with diabetes, including DD and psychological comorbidities. The recommendations were to 1) to routinely monitor people with diabetes for DD particularly when glycaemic treatment targets are not met and/or at the onset of diabetes complications, using appropriate validated measures; and 2) if DD is identified, the person should be referred for diabetes education to address areas of diabetes self-care that are most relevant to the person with diabetes and have the most impact on diabetes outcomes. People whose self-care remains impaired after tailored diabetes education should be referred by their care team to a behavioural health specialist for evaluation and support. These recommendations were based on commonly used clinical models, expert consensus and tested interventions, but not on a systematic review of the evidence [23].

The European Association for the Study of Diabetes (EASD) recognized the importance of DD and commissioned an evidence-based clinical guideline to enable healthcare professionals to optimise their support of people living with diabetes. The EASD Guideline Oversight Committee commissioned this systematic review and meta-analysis to inform the development process according to the EASD standard operating procedure [24].

The aim of the systematic review was to update and expand the evidence base on the clinical management of DD, by including RCTs that assess the efficacy of a wide range of interventions on DD as primary or secondary outcome. We systematically identified, summarized and critically appraised all available evidence from RCTs assessing the efficacy of interventions for reducing DD (either as primary or secondary outcome) among adults with type 1 diabetes or type 2 diabetes. This resulted in an up-to-date evidence-based overview that reflected current practices and innovations in the management of DD [25]. This paper describes the protocol for the systematic review to provide a

transparent record of the original planned methods and allow readers to systematically compare the original protocol with the completed review.

**Methods**

*Protocol and registration*

This protocol was developed in collaboration with the EASD Guideline Development Panel (GDP) for DD, which included two people with lived experience of diabetes, who ensured that the clinical questions, interventions and outcomes were of relevance to people living with diabetes. This protocol adhered to the Preferred Reported Items for Systematic Reviews and Meta-analysis Protocols (PRISMA-P) and was registered in PROSPERO, the International Prospective Register of Systematic Reviews (registration number CRD42024598512) [26].

*Eligibility criteria*

Studies were included if all the following criteria were satisfied:

1) The study focussed on adults (≥18 years) with type 1 diabetes or type 2 diabetes.
2) The study examined the efficacy of an active intervention, which could be classified as one of three types:
    a) Psychological therapeutic interventions, which could be cognition-focused, emotion-focused, emotion-cognition-focused therapeutic interventions, or interventions facilitated by a health professional trained in psychology. Examples included, but were not limited to: cognitive behavioural therapy, motivational interviewing, supportive counselling, psychotherapy, mindfulness, acceptance and commitment therapy. The interventions were typically delivered one-to-one (though not exclusively);
    b) Diabetes self-management education, peer support with a psychological component, health coaching or psycho-education interventions, which included a diabetes-related educational component (e.g., carbohydrate counting, insulin dose adjustment) as well as focussing on improving self-management (e.g., cognitive reframing, goal setting, planning, problem solving) based on psychological theory or theories. This category typically included interventions delivered at greater scale, e.g. in groups or via online programs, and included interventions such as, for example (but not limited to): peer support, motivational interviewing, conversation maps, depression treatment, psycho-education, self-management support and nudging;
    c) Other types (miscellaneous), which included interventions that could not be categorized as A or B and examples included, but were not limited to:

- devices (for the administration of insulin or other glucose-lowering medications, or for glucose monitoring, or both); education or peer support without a clearly described psychological component (i.e. interventions with no clear description of the use of frameworks, models or techniques that have psychology or behavioural science at their core);
- telehealth interventions (e.g. apps, online interventions of a diverse nature);
- health behavioural interventions (e.g. physical activity, sleep or, weight loss interventions);
- medication interventions (e.g. glucose-lowering injectable or oral medication);
- complex health service interventions (e.g. healthcare pathways, models of care);
- insulin titration (e.g. different protocols for insulin titration);
- and personalised risk assessment or use of Patient- Reported Outcome Measures (PROMs) to assess outcomes in care.

The categories were described if ten or more studies reported on the same type of intervention. Regardless of classification, eligible interventions could be generic or diabetes-specific, delivered to individuals (one-to-one) or in groups, delivered in-person, online, via applications, or in combination of different methods, facilitated by specialist mental health professionals, specialist diabetes health professionals, general health professionals, or expert peers (i.e., people with lived experience of diabetes who are trained to deliver the intervention). Eligible comparators for these interventions included usual care, waiting list, placebo, or any other active intervention as indicated above. These factors, intervention characteristics and comparators were explored as possible explanations for heterogeneity.

3. The study reported results on DD as a primary or secondary outcome, using data collected with the use of a validated measure. Such measures included, but were not limited to: the Diabetes Distress Scale (DDS) [5], the Problem Areas In Diabetes (PAID) scale [1], the Questionnaire on Stress in Diabetic Patients (QSD) [27] and their subscales or derivatives (including short-form measures and versions developed for specific populations, e.g. for adults with type 1 diabetes).
4. The study was an RCT, irrespective of its duration or the type of randomization used (e.g. individual, cluster).

There were no language limitations and no exclusion criteria related to publication date. Non-English papers were translated using the help of native speaking colleagues.

*Information sources and search strategy*

We performed a systematic search in four electronic databases: PubMed, the Cochrane Library (Wiley), APA PsycInfo (via EBSCOhost) and CINAHL Plus (via EBSCOhost) from inception to 23 September 2024. Trial registries (ClinicalTrials.gov and WHO ICTRP) were searched via the Cochrane Library. Furthermore, the reference lists of all included publications were screened. The search was devised in collaboration with a librarian. Three domains were used in the following search: (1) diabetes, (2) distress, and (3) RCT. The three domains were included in a three-step search combining relevant search words and MeSH term with Boolean logic operators (OR and AND). The search strategy was initially targeted to PubMed and subsequently adapted to other databases. Supplemental file 1 shows the complete search string that was used.

*Data management*

We utilized the Covidence online platform (Covidence systematic review software, Veritas Health Innovation, Melbourne, Australia; www.covidence.org) for importing search results, removing duplicates, screening articles, and extracting data. EndNote was used as reference manager [28].

*Selection process*

After removing duplicates, two independent reviewers screened titles and abstracts to identify potentially eligible papers. The full text versions of potentially eligible records were also assessed, independently, by two reviewers. When papers reported on the same study, we used the most recent, complete paper. Reasons for exclusion were recorded. Discrepancies during abstract screening and full text screening were resolved through discussion within the team. Reference lists of the included publications were screened manually with the aim to identify additional eligible publications. A PRISMA flowchart was created to describe the selection process [29].

*Data extraction*

Data from eligible studies was extracted using a form that was developed a priori and pilot tested. From each of the eligible studies, the following information was extracted:

1) Study characteristics (e.g., first author, publication DOI, year of publication, type of study, trial identifier (i.e., National Clinical Trial number), trial duration, duration of intervention, definition of intervention and comparator, geographical location, number of participants with type 1 or type 2 diabetes).
2) Participants' baseline characteristics (e.g., demographics), relevant clinical characteristics (e.g. duration of diabetes, HbA1c).
3) Outcome-related data for each trial arm, including measurement method and relevant metrics (baseline and end-of-study mean, change from baseline value, 95% confidence

interval / standard deviation / standard error, and number of participants analyzed for continuous outcomes, as well as number of participants with outcome and number analyzed for binary outcomes, and time-point of outcome assessment for all outcomes). The primary outcome (DD) was assessed as a continuous outcome (i.e., calculated as between-arm difference at a given time-point; or change from baseline [follow-up minus baseline]; or post-intervention score) and/or a binary outcome (i.e., number/proportion of participants with a score (post-intervention minus pre-intervention or post-intervention) above or below a pre-specified threshold value deemed indicative of presence/absence of DD).

Data extraction was performed by one independent reviewer and was completely checked by a second reviewer. Discrepancies were resolved through discussion or by consultation with a third reviewer.

*Risk of bias in individual studies*

Risk of bias assessment was completed at study level. using the Cochrane RoB 2 tool for RCTs [30]. The five bias domains of this tool include:

1. Domain 1 assesses bias arising from the randomization process, i.e.. lower rating if information on randomization was missing or baseline differences in the outcome variables suggest a problem with randomization;
2. Domain 2 assesses bias due to deviations from the intended interventions, i.e.. lower ratings if there were deviations from the intended intervention that arose because of the trial context or analyses were not conducted based on intention to treat analysis;
3. Domain 3 assesses bias due to missing outcome data, i.e. lower ratings if more than 5% of the primary outcome data were missing and/or no analysis was conducted to assess if missing data influenced the outcome;
4. Domain 4 assesses bias in measurement of the outcome, with in this case lower ratings for all studies, while an inclusion criteria for our review was using validated Patient Reported Outcome Measures;
5. Domain 5 assesses bias in selection of the reported result, i.e. lower ratings when analyses were not conducted in concurrence with the analysis plan or when there were multiple analysis of the outcome; and
6. The ‘Overall risk of bias’ judgement, is the summary score of the five bias domains. Overall risk of bias is considered ‘low’ if the RCT is judged to be low risk in all domains, and ‘high’ when the trial is judged to be high risk in at least one domain or the trial is judged to have

some concerns for multiple domains. The overall risk of bias is considered as 'some concerns' in all other cases.

Risk of bias will be performed by one independent reviewer and will be checked by a second reviewer. Discrepancies will be resolved through discussion or the consultation of a third reviewer.

*Certainty of evidence assessment*

Certainty (quality) of meta-analysis findings were assessed based on the GRADE methodology [31]. Based on the GRADE framework we rated our findings for each comparison as high, moderate, low, or very low, based on factors including risk of bias, inconsistency, indirectness, imprecision, and publication (small study effect) bias. Publication bias was examined graphically (i.e., funnel plot) and statistically (i.e., Egger's test) [32, 33].

*Data synthesis*

Statistical analyses, where applicable, were conducted using R (version 4.3.2) [34]. Quantitative data synthesis were conducted using random effects meta-analysis, if two or more studies reported on the same outcome [35]. Effect sizes (mean differences between intervention and comparator in terms of change from baseline value or post-intervention value; odds ratio's (OR) or relative risks (RR) between intervention and comparator) alongside 95% confidence intervals were calculated. In case of different scales used for the assessment of DD, standardized mean differences (SMDs) were used. In case of reported RR or OR accompanied by an uncertainty measure (standard error, 95% confidence interval or exact p-value), data were log-transformed and analysed using the generic inverse variance method. Regarding the assessment as a continuous outcome, minimal clinically important differences (MCIDs) were defined based on relevant existing literature or, in the absence of such literature, GDP expert opinion. These MCIDs were used for the Certainty of Evidence Assessment through the Grading of Recommendations Assessment, Development and Evaluation (GRADE) approach. If we included trials that were cluster randomised, effect estimates were adjusted for clustering when the intracluster correlation coefficient (ICC) was reported. If the ICC was not available and adjustment was not possible, these studies were excluded in a sensitivity analysis to evaluate their impact on the pooled estimates.

We performed all analysis stratified for type of diabetes for both the primary and secondary outcomes. Additionally, we stratified analyses for types of comparator (i.e., usual care and waiting list; active comparators) for the primary outcome. Heterogeneity between studies were assessed using the $I^2$ statistic (with 95% confidence interval). In case of substantial heterogeneity for the primary outcome, we performed sensitivity analyses based on 1) risk of bias score, 2) intervention

delivery format (i.e., one-to-one vs group-based; in-person vs online vs application), 3) intervention duration, 4) study type (e.g., RCT versus pilot RCT), 5) sample size (e.g. <100 vs ≥ 100 [36]) and possibly other relevant subgroups based on study and participants' baseline characteristics (e.g., duration of diabetes, age, sex).

**Changes made from the original protocol**

Initially, the systematic review protocol (as registered in PROSPERO CRD42024598512) included additional secondary outcomes, such as HbA1c, anxiety, depression, self-management, and quality of life. However, we decided to narrow our focus to include only DD as the primary outcome and HbA1c as the sole secondary outcome due to the large number of papers that were identified. By narrowing the scope to DD while retaining HbA1c as the sole secondary outcome, we were able to provide a clearer and more relevant perspective on interventions targeting DD, while retaining clinically relevant information. This decision also reduced the risk of bias, as many studies only reported on DD and not on other outcomes, or on a random selection of other outcomes. Had we attempted to synthesize all these additional outcomes, we would likely have captured only a subset of the available data, resulting in a less comprehensive and potentially biased synthesis of the evidence.

A second change from the original protocol is that we were unable to conduct an analysis on DD as a binary outcome, as very few trials reported binary outcomes (e.g. high versus low DD) and those that did also reported DD as a continuous outcome. A third change was the fact that after data extraction, we were only able to meta-analyse post-intervention (endpoint) values, as many trials did not report baseline values or change-from-baseline scores for DD. In this instance, the Cochrane Handbook advises that post-intervention values and change scores should not in principle be combined using standard meta-analysis approaches when the effect measure is a standardized mean difference (SMD). Finally, we conducted an additional subgroup analysis based on baseline DD scores (low vs high). This subgroup was specified after data extraction, following discussions with the Guideline Development Panel, who considered that the large heterogeneity across trials might be explained in part by the inclusion of many participants with low baseline DD, which could attenuate observed effect sizes (i.e. participants with low baseline DD being less likely to show substantial improvement than those with higher baseline DD).

**Discussion**

We have described the protocol used for the systematic review that informed the EASD Clinical Practice Guideline on DD [25]. This review systematically identified, summarized and critically appraised all available evidence from RCTs assessing the efficacy of interventions for reducing DD (either as primary or secondary outcome) among adults with type 1 diabetes or type 2 diabetes. The review has been finished and the paper describing the systematic review [25] as well as the guideline [37] are accepted to be published. Publication of the original protocol and subsequent changes will facilitate reader transparency and accessibility to the published products.

In addition to the section on changes to protocol described above, we received several comments during the peer review process of this paper. Unfortunately, these were communicated too late to influence the conduct of the systematic review, but nevertheless warrant consideration. These mainly related to the search strategy scope, database selection, outcome handling, and analytical decisions.

First, although we used the PubMed, Cochrane Database of Systematic Reviews, PsycINFO, and CINAHL databases for our search, other biomedical and multidisciplinary databases such as EMBASE, Web of Science, and Scopus were not included. Given the wide range of interventions, it is possible that reliance on a limited set of databases may have increased the risk of incomplete retrieval, particularly for device- and technology-based RCTs, health service interventions and trials published in non-nursing or non-psychology journals. However, we believe that our current search strategy represents an optimal and efficient choice, given that the Cochrane Library search also draws on data from Embase as well as from ClinicalTrials.gov and WHO ICTRP. In addition, our rigorous reference searching identified 41 studies, which suggests that this approach functioned as an effective safety net for possible other studies. This was strengthen by the fact that public review of the result paper of the guideline [37] and journal review of the systematic review [25] did not identify any additional studies.

Second, the search was planned from inception to 23 September 2024, with no decision to update the review prior to completion of the guideline. For a review intended to inform clinical guidelines, the search may have become outdated as trials in psychosocial and digital interventions are constantly emerging. As is customary in the field an update of the review is planned to coincide with any scheduled update of the guideline in the future.

Third, although including trials where DD was a secondary outcome is a strength of the review, it raises analytical challenges. Although we already included a large number of subgroups and sensitivity analysis, we could have added more. The primary versus secondary outcome bias will be reflected by the subgroups we made in advance as a large part of the psychological therapeutic interventions will have been aimed at lowering DD, while most of the miscellaneous studies will not.

Fourth, our protocol allowed for DD to be analyzed as post-intervention score, change from baseline, binary threshold outcomes and multiple time points. The lack of a clearly defined primary time point may result in selective analysis. However, as many studies did not report baseline (SD) scores, this prevented us from using change scores. Furthermore as time to follow up was very heterogeneous, it was not feasible to report outcomes at a set timepoint (e.g. six months). We therefore prioritized the last follow-up time point and conducted a sensitivity analysis for study duration. As a result it turned out to be the right decision to not specify in the protocol time points as if we would have chosen one (e.g change score) we would not have been able to conduct that analysis or in a very small selected sample of studies, leading to bias.

Overall, this protocol for our review provided a comprehensive, up-to-date synthesis of the available RCT evidence of the impact of a wide range of interventions for reducing DD among adults with type 1 diabetes or type 2 diabetes [25]. The findings from this systematic review and meta-analysis formed the evidence base for the EASD *Evidence-Based Clinical Practice Guideline for Assessing and Managing Diabetes Distress Among Adults with Type 1 Diabetes or Type 2 Diabetes* [37], developed in accordance with the EASD Guidelines Standard Operating Procedures [24]. The overarching goal of the guideline is to provide clear evidence-based recommendations regarding whether interventions should be used to manage DD in adults diabetes and, if so, which interventions should be considered. The systematic review enabled the Guideline Development Panel to address this clinical question rigorously and transparently.

**Supplementary information**

Additional file 1

**Abbreviations**

| | |
|---|---|
| ADA | American Diabetes Association |
| DDS | Diabetes Distress Scale |
| EASD | European Association for the study of Diabetes |
| HbA1c | Hemoglobin A1c (a measure of average blood glucose over the past 8-12 weeks) |
| GRADE | Grading of Recommendations Assessment, Development and Evaluation |
| PAID | Problem Areas In Diabetes |
| PRISMA-P | Preferred Reporting Items for Systematic Reviews and Meta-Analyses Protocols |
| QSD | Questionnaire on Stress in Diabetic Patients |
| RCT | Randomized controlled trial |
| SMDs | standardized mean differences |
| TIR | Time in range |

**Declarations**

a. Ethics approval and consent to participate

Not applicable

b. Consent for publication

Not applicable

c. Availability of data and material

Not applicable

d. Competing interests

SBJS, SR, MB, LG, PJME, JWB, FR declared no conflict of interest

Mariska Langendam declares no conflict of interest

Martin M. Jensen declares no conflict of interest

Walther Jensen declares no conflict of interest

Norbert Hermanns declares Advisory Board member fees from Abbott Diabetes Care, Dexcom and Insulet as well as honoraria from lecturs from Sanofi Germany, Roche Diabetes Carte, and Abbott Diabetes Care, Germay

Richard Holt declares that he has received honoraria for speaker engagements from Boehringer-Ingelheim, Eli Lilly, Enore, Liberum, Novo Nordisk and ROVI Pharma and funding for conference attendance from NOVO Nordisk and Eli Lilly

Karin Kanc declares no conflict of interest

Thomas Karagiannis declares no conflict of interest.

Michelle Law declares declares no conflict of interest

Andreia Mocan declares no conflict of interest

Jane Speight declares no conflict of interest

Jackie Sturt declares membership of the Novo Nordisk Foundation Global Diabetes Centre Forum

Francesco Zaccardi declares no conflict of interest

e. Funding

The EASD has provided financial support to enable SS, FR, TK, and FZ to work on the present protocol and the systematic review. The EASD did not contribute to the development of the protocol or to the manuscript. The professional members of the Guideline Development Panel were not funded by the EASD. JSp is supported by core funding to the Australian Centre for Behavioural Research in Diabetes provided by the collaboration between Diabetes Victoria and Deakin University

f. Authors' contributions

All authors have contributed in a meaningful way. RIGH and JSp conceived the idea for the guideline, and systematic review, which was operationalized by the Guideline Development Panel, comprising

WJ, NH, RIGH, KK, TK, ML, AM, JSp, JSt and FZ. TK, FZ, SS and FR made the first draft of the protocol. All authors have reviewed and commented on the protocol. All authors have read and approved the final manuscript.

g. Acknowledgements

We want to thank the administrative staff of the EASD as well as Linda Schoonmade (librarian, Amsterdam UMC) for their support during the systematic review.

h. Authors information

NA

**References**


1. Polonsky, W.H., et al., *Assessment of diabetes-related distress.* Diabetes Care, 1995. **18**(6): p. 754–60.
2. Fisher, L., R.E. Glasgow, and L.A. Strycker, *The relationship between diabetes distress and clinical depression with glycemic control among patients with type 2 diabetes.* Diabetes Care, 2010. **33**(5): p. 1034–6.
3. Fisher, L., et al., *When is diabetes distress clinically meaningful?: establishing cut points for the Diabetes Distress Scale.* Diabetes Care, 2012. **35**(2): p. 259–64.
4. Morales-Brown, L.A., G. Perez Algorta, and Y. Salifu, *Understanding Experiences of Diabetes Distress: A Systematic Review and Thematic Synthesis.* Journal of Diabetes Research, 2024. **2024**(1): p. 3946553.
5. Polonsky, W.H., et al., *Assessing psychosocial distress in diabetes: development of the diabetes distress scale.* Diabetes Care, 2005. **28**(3): p. 626–31.
6. McGuire, B.E., et al., *Short-form measures of diabetes-related emotional distress: the Problem Areas in Diabetes Scale (PAID)-5 and PAID-1.* Diabetologia, 2010. **53**(1): p. 66–9.
7. Stanulewicz, N., et al., *PAID-11: A brief measure of diabetes distress validated in adults with type 1 diabetes.* Diabetes Res Clin Pract, 2019. **149**: p. 27–38.
8. Fisher, L., et al., *Development of a brief diabetes distress screening instrument.* Ann Fam Med, 2008. **6**(3): p. 246–52.
9. Dennick, K., J. Sturt, and J. Speight, *What is diabetes distress and how can we measure it? A narrative review and conceptual model.* J Diabetes Complications, 2017. **31**(5): p. 898–911.
10. Dennick, K., et al., *High rates of elevated diabetes distress in research populations: A systematic review and meta-analysis.* International Diabetes Nursing, 2015. **12**(3): p. 93–107.
11. Perrin, N.E., et al., *The prevalence of diabetes-specific emotional distress in people with Type 2 diabetes: a systematic review and meta-analysis.* Diabetic Medicine, 2017. **34**(11): p. 1508–1520.
12. Skinner, T.C., L. Joensen, and T. Parkin, *Twenty-five years of diabetes distress research.* Diabet Med, 2020. **37**(3): p. 393–400.
13. Aikens, J.E., *Prospective associations between emotional distress and poor outcomes in type 2 diabetes.* Diabetes Care, 2012. **35**(12): p. 2472–8.
14. Todd, P.J., et al., *Evaluating the relationships of hypoglycaemia and HbA1c with screening-detected diabetes distress in type 1 diabetes.* Endocrinology, Diabetes & Metabolism, 2018. **1**(1): p. e00003.
15. Chew, B.H., et al., *Psychological interventions for diabetes-related distress in adults with type 2 diabetes mellitus.* Cochrane Database Syst Rev, 2017. **9**(9): p. CD011469.
16. Mathiesen, A.S., et al., *Psychosocial interventions for reducing diabetes distress in vulnerable people with type 2 diabetes mellitus: a systematic review and meta-analysis.* Diabetes Metab Syndr Obes, 2019. **12**: p. 19–33.
17. Ngan, H.Y., Y.Y. Chong, and W.T. Chien, *Effects of mindfulness- and acceptance-based interventions on diabetes distress and glycaemic level in people with type 2 diabetes: Systematic review and meta-analysis.* Diabet Med, 2021. **38**(4): p. e14525.
18. Perrin, N., et al., *Effectiveness of psychoeducational interventions for the treatment of diabetes-specific emotional distress and glycaemic control in people with type 2 diabetes: A systematic review and meta-analysis.* Prim Care Diabetes, 2019. **13**(6): p. 556–567.
19. Schmidt, C.B., et al., *Systematic review and meta-analysis of psychological interventions in people with diabetes and elevated diabetes-distress.* Diabet Med, 2018.
20. Sturt, J., et al., *Effective interventions for reducing diabetes distress: systematic review and meta-analysis.* International Diabetes Nursing, 2015. **12**(2): p. 40–55.
21. Boscari, F., et al., *Implantable and transcutaneous continuous glucose monitoring system: a randomized cross over trial comparing accuracy, efficacy and acceptance.* J Endocrinol Invest, 2022. **45**(1): p. 115–124.

22. Ding, S.A., et al., *Adjustable Gastric Band Surgery or Medical Management in Patients With Type 2 Diabetes: A Randomized Clinical Trial.* J Clin Endocrinol Metab, 2015. **100**(7): p. 2546–56.
23. Young-Hyman, D., et al., *Psychosocial Care for People With Diabetes: A Position Statement of the American Diabetes Association.* Diabetes Care, 2016. **39**(12): p. 2126–2140.
24. Karagiannis T, Advani A, Davies MJ, Del Prato S, Dinneen SF, Kuss O, Lorenzoni V, Mathieu C, Mauricio D, Tankova T, Zaccardi F, Holt RIG, Tsapas A; EASD Guidelines Oversight Committee. European Association for the Study of Diabetes (EASD) Standard Operating Procedure for the development of guidelines. Diabetologia. 2025;68(8):1600-1615. doi:10.1007/s00125-025-06370-1.
25. Schipper SBJ. et al. Interventions to reduce diabetes-related distress among adults with type 1 diabetes or type 2 diabetes: systematic review and meta-analyses of randomised controlled trials. Diabetologia (2026). https://doi.org/10.1007/s00125-026-06789-0
26. Shamseer, L., et al., *Preferred reporting items for systematic review and meta-analysis protocols (PRISMA-P) 2015: elaboration and explanation.* BMJ : British Medical Journal, 2015. **349**: p. g7647.
27. Duran, G., et al., *Assessing daily problems with diabetes: a subject-oriented approach to compliance.* Psychol Rep, 1995. **76**(2): p. 515–21.
28. The EndNote Team. EndNote 21. Philadelphia, PA: Clarivate Analytics; 2023
29. Page, M.J., et al., *The PRISMA 2020 statement: an updated guideline for reporting systematic reviews.* BMJ, 2021. **372**: p. n71.
30. Higgins, J.P., et al., *Assessing risk of bias in a randomized trial*, in *Cochrane Handbook for Systematic Reviews of Interventions*. 2019. p. 205–228.
31. Balshem, H., et al., *GRADE guidelines: 3. Rating the quality of evidence.* Journal of Clinical Epidemiology, 2011. **64**(4): p. 401–406.
32. Egger, M., et al., *Bias in meta-analysis detected by a simple, graphical test.* Bmj, 1997. **315**(7109): p. 629–34.
33. Light, R.J.P., D.B., *Summing up: The Science of Reviewing Research*. 1984, Cambridge, Massachusetts: Harvard University Press.
34. R Core Team. R: A language and environment for statistical computing. Vienna, Austria: R Foundation for Statistical Computing; 2023.
35. Riley, R.D., J.P.T. Higgins, and J.J. Deeks, *Interpretation of random effects meta-analyses.* BMJ, 2011. **342**: p. d549.
36. Beets, M.W., et al., *Influence of pilot and small trials in meta-analyses of behavioral interventions: a meta-epidemiological study.* Systematic Reviews, 2023. **12**(1): p. 21.
37. Speight J, Hermanns N, Jensen W, Kanc K, Karagiannis T, Law M, Mocan A, Rutters F, Sturt J, Zaccardi F, Holt RIG. 2026 EASD evidence based clinical practice guideline for assessing and managing diabetes distress among adults with type 1 diabetes or type 2 diabetes. Diabetologia (2026). https://doi.org/10.1007/s00125-026-06840-0

## 8. Differences between protocol and review

In the section we discuss the deviations from the protocol, as the protocol is presented on Prospero and as it has been submitted to the journal Trials (currently under review).

1) We were unable to conduct an analysis on diabetes distress as a binary outcome, as hardly any trials report binary outcomes (e.g. high versus low DD) and those that did also reported on diabetes distress as a continuous outcome, resulting in a decision of the evidence synthesis team to include the latter.
2) No additional outcomes were extracted (depression, anxiety, general health status, treatment satisfaction and diabetes self-management). Together with the Guideline Development Panel, we decided to focus the evidence synthesis exclusively on diabetes distress. This reflects both practical considerations related to the high volume of eligible trials and methodological considerations regarding alignment with the scope and objective of the guideline. Because the search strategy and eligibility criteria were centred on diabetes distress, trials evaluating similar interventions but not assessing diabetes distress were not systematically sought. Consequently, any quantitative synthesis of secondary outcomes (such as HbA1c) based solely on the included studies would be incomplete and potentially misleading.
3) After data-extraction, we were able to only meta-analyze post-intervention (endpoint) values, as too many trials did not report on baseline values of diabetes distress or reported change from baseline for diabetes distress. As the Cochrane handbook advices that 'post-intervention value and change scores should not in principle be combined using standard meta-analysis approaches when the effect measure is an SMD' (Cochrane handbook).
4) We conducted an additional subgroup analysis, namely based on baseline score diabetes distress (low vs high). This was done after the data extraction had been completed, because the Guideline Development Panel considered that the large heterogeneity across trials may have been due to many trials including participants with low level of baseline diabetes distress, which in turn might influence the effect size in reduction of diabetes distress (i.e. participants with low baseline levels of distress being less likely to improve compared with those with higher baseline levels of distress).
